# NanoMnT: an STR analysis tool for Oxford Nanopore sequencing data driven by a comprehensive analysis of error profile in STR regions

**DOI:** 10.1093/gigascience/giaf013

**Published:** 2025-03-17

**Authors:** Gyumin Park, Hyunsu An, Han Luo, Jihwan Park

**Affiliations:** School of Life Sciences, Gwangju Institute of Science and Technology (GIST), Gwangju 61005, Republic of Korea; School of Life Sciences, Gwangju Institute of Science and Technology (GIST), Gwangju 61005, Republic of Korea; Department of Thyroid and Parathyroid Surgery, Laboratory of thyroid and parathyroid disease, Frontiers Science Center for Disease-related Molecular Network, West China Hospital, Sichuan University, Chengdu, Sichuan 61005, China; School of Life Sciences, Gwangju Institute of Science and Technology (GIST), Gwangju 61005, Republic of Korea

**Keywords:** Oxford Nanopore, long-read sequencing, short tandem repeats, microsatellite, error profile, bioinformatics

## Abstract

Oxford Nanopore Technology (ONT) sequencing is a third-generation sequencing technology that enables cost-effective long-read sequencing, with broad applications in biological research. However, its high sequencing error rate in low-complexity regions hampers its applications in short tandem repeat (STR)–related research. To address this, we generated a comprehensive STR error profile of ONT by analyzing publicly available Nanopore sequencing datasets. We show that the sequencing error rate is influenced not only by STR length but also by the repeat unit and the flanking sequences of STR regions. Interestingly, certain flanking sequences were associated with higher sequencing accuracy, suggesting that certain STR loci are more suitable for Nanopore sequencing compared to other loci. While base quality scores of substitution errors within the STR regions were lower than those of correctly sequenced bases, such patterns were not observed for indel errors. Furthermore, choosing the most recent basecaller version and using the super accuracy model significantly improved STR sequencing accuracy. Finally, we present NanoMnT, a lightweight Python tool that corrects STR sequencing errors in sequencing data and estimates STR allele sizes. NanoMnT leverages the characteristics of ONT when estimating STR allele size and exhibits superior results for 1-bp- and 2-bp repeat STR compared to existing tools. By integrating our findings, we improved STR allele estimation accuracy for Ax10 repeats from 55% to 78% and up to 85% when excluding loci with unfavorable flanking sequences. Using NanoMnT, we present the utility of our findings by identifying microsatellite instability status in cancer sequencing data. NanoMnT is publicly available at https://github.com/18parkky/NanoMnT.

## Introduction

Short tandem repeats (STRs), also known as microsatellites, are regions of DNA consisting of a repeated sequence of 1–6 bp [1] and are routinely employed as “genetic fingerprints” in a variety of fields, such as forensics [2] and population genetics [3]. Previous research has shown that STRs are also involved in gene regulation by modulating DNA methylation and providing binding sites for transcription factors [4–6]. Moreover, owing to their high mutation rate [7], STRs play critical roles in the pathogenesis of numerous degenerative diseases, including fragile X syndrome, spinal and bulbar muscular atrophy (SBMA), and Huntington disease [8]. The major pathomechanisms of repeat expansions are diverse: loss of function via transcriptional repression, gain of function of canonically translated repeat-containing proteins, and RNA-mediated gelation and sequestration of RNA-binding proteins, reflecting the functional importance of STR [8]. In addition, the genomic instability of STRs in mismatch repair-deficient (dMMR) cancers manifests as a molecular phenotype known as microsatellite instability (MSI), which is generally associated with a favorable response to cancer immunotherapy due to abundant generation of neoantigens [9, 10]. The allele sizes of STRs, defined by the number of repeats, are crucial for understanding their roles in these biological contexts. Consequently, accurate quantification of STR allele size is essential for assessing their functional roles and advancing our understanding of their contributions to health and disease.

Although conventional PCR-based methods for analyzing STRs have proven effective, they are limited by the number of STR regions that can be analyzed simultaneously [11]. However, advancements in next-generation sequencing (NGS) have significantly expanded the number of analyzable STR regions, to the point where whole-genome sequencing (WGS) with sufficient sequencing depth can enable the analysis of most STR regions [12]. Long-read sequencing technologies are generally more advantageous than short-read sequencing for characterizing STR regions because they can span not only the STR sequences but also their flanking sequences. This capability enables robust read alignments of STR regions, which is particularly valuable for analyzing STR with complex structures, such as compound and imperfect STRs [13]. In contrast, short-read sequencing often fails to sequence the flanking sequences of STR regions, which may result in producing misalignments. Furthermore, the lengths of some STR alleles exceed the read lengths of short-read sequencing, requiring longer reads for accurate STR allele identification [14, 15].

PacBio sequencing and Oxford Nanopore sequencing (also referred to as Oxford Nanopore Technologies or ONT) are 2 of the most widely used long-read sequencing technologies, with extensive applications in various research fields [16, 17]. Each technology has distinct advantages. PacBio HiFi sequencing achieves impressively high sequencing accuracy (99.8%, >Q30), comparable to that of Illumina sequencing (99.9%), and provides an average read length of 13.5 kb (although this varies depending on the fragmentation step) [18]. In contrast, ONT enables real-time analysis, portability, cost-effectiveness, and “ultra-long” reads exceeding 100 kb [19]. However, many studies have reported that ONT exhibits higher sequencing error rates than other sequencing technologies [20, 21].

A previous comprehensive analysis reported that approximately half of all sequencing errors occur in STR regions [22], which complicates downstream analyses that require accurate assessments of STR lengths (i.e., number of repeats). For example, indel errors in homopolymer regions can critically affect gene calling and protein prediction by introducing false frameshift events in coding sequences [23]. In an effort to improve sequencing accuracy, ONT has progressively updated flowcells, sequencing kits, and basecalling programs. One of the most notable improvements was seen after upgrading from the R9.4.1 flowcell (read accuracy Q17) to the R10.4.1 flowcell (read accuracy Q21), which greatly reduced the number of errors [24, 25]. Furthermore, the recent introduction of the ligation sequencing kit V14, designed to achieve single-pass modal accuracy of >Q20, has further improved ONT sequencing accuracy [25]. Nevertheless, the high sequencing error rate of ONT in low-complexity regions remains a significant challenge, and a comprehensive study of ONT error profiles in STR regions is still lacking.

Despite these challenges, numerous bioinformatic tools have been developed to analyze STR regions from ONT data. These tools can be grouped into 2 categories based on the type of input file: electric signal-based tools, which analyze STRs directly from the raw electric current data (e.g., FAST5 or POD5 files), and sequence-based tools, which analyze STRs from the basecalled data (e.g., FASTQ or BAM files) [26]. Although each group of tools has been successfully applied to its respective areas of interest, many are no longer maintained or have certain limitations. For example, electric signal-based tools such as WarpSTR [27], DeepRepeat [28], STRique [29], and NanoSatellite [30] require FAST5 files, which are often unavailable in public datasets. Additionally, many electric signal-based tools are incompatible with the newly introduced POD5 format, which is the current output format of ONT. In contrast, while sequence-based tools are more versatile in this aspect, many rely on discontinued dependencies. For instance, NanoSTR [31] requires Porechop (RRID:SCR_016967), which was officially discontinued as of October 2018, and PacmonSTR [32] requires BLASR (RRID:SCR_000764), which is also no longer maintained. Furthermore, many sequence-based tools do not support multithreading, severely limiting their scalability. Notably, none of these tools, including well-maintained tools such as Straglr [33], NanoRepeat [34], and tandem genotypes [35], have been tested for estimating mononucleotide repeats, which are the most error-prone regions in ONT [22].

In this study, we performed a comprehensive analysis of ONT error profiles in STR regions using 3 publicly available ONT sequencing datasets (Supplementary Table S1). We centered our analysis on the T2T-CHM13 dataset (generated using R9.4.1 flowcells) because of its high sequencing depth (∼120×) and the availability of the T2T-CHM13 reference genome, which serves as the ground truth. The near homozygosity of the CHM13 cell line removes the need to consider biallelic STR signals, enabling a straightforward analysis. To validate our findings and translate them to the current R10.4.1 flowcell version, we incorporated 2 additional datasets of the HG002 genome, one generated using the R9.4.1 flowcell and another using the R10.4.1 flowcell, both publicly available through EPI2ME. These HG002 datasets are referred to as the “HG002 R9.4.1 dataset” and the “HG002 R10.4.1 dataset” throughout the article. The HG002 R10.4.1 dataset was generated using the V14 kit (SQK-LSK114), allowing us to explore the error profile of the most up-to-date ONT configuration. Unless otherwise specified, (i) analyses of the R9.4.1 STR error profile were performed using the CHM13 dataset, and (ii) the R9.4.1 data presented in this article were basecalled with the Guppy v6.5.7 high-accuracy (HAC) model, whereas the R10.4.1 data were basecalled with the Dorado v8.1.0 HAC model. Finally, we present NanoMnT (RRID:SCR_026210), a lightweight Python-based tool that performs error correction for ONT reads in STR regions and estimates STR allele size. We demonstrate the utility of our findings by identifying MSI status of 4 cancer cell lines from the Singapore Nanopore Expression Project (SG-NEx) dataset [36] and 15 colorectal cancer (CRC) organoids [37] using NanoMnT.

## Methods

### Identification of STR regions

We employed Krait (v1.3.3, default settings, except the minimum repeat length requirement for 1-bp repeat STRs has been lowered to 10 bp) [38], an ultrafast bioinformatic program designed to identify STRs from genomes via brute-force search algorithm 2 described by Sokol et al. [39], to search for STR regions within the T2T-CHM13 (v2.0) genome and the HG002 (maternal genome, v1.0.1) genome.

1. T2T-CHM13
  Running Krait on the T2T-CHM13 genome (v2.0) resulted in an initial set of 2,103,586 STR regions. Because many 1-bp/2-bp/3-bp repeat STR regions were flanked by low-complex flanking sequences that closely resembled the STR sequences, 1-bp, 2-bp, and 3-bp repeat STR regions whose flanking sequences had excessively low *k*-mer diversity (see “Calculation of *k*-mer diversity” in Methods) were filtered out (*k*-mer $\le 2.5$ for 1-bp repeat, $\le 2.0$ for 2-bp repeat, $\le 5.0$ for 3-bp repeat), as they may introduce ambiguity when measuring STR repeat sizes. This resulted in 1,288,130 STR regions being left. Subsequently, regions with a read orientation–specific coverage of at least 20 (i.e., at least 20 forward strand reads or 20 reverse strand reads) were selected, resulting in the final set of 762,311 STR regions.
2. HG002 (Maternal genome)
  First, 1-bp, 2-bp, and 3-bp repeat STR regions were identified from the HG002 maternal assembly using Krait, resulting in an initial set of 1,316,436 STR regions. Unlike the CHM13 genome (which is haploid), the HG002 genome contains a considerable number of biallelic STR regions, which complicate the analysis of the ONT error profile. Therefore, we decided to exclusively use monoallelic STR regions in our analysis by employing LiftOff (v1.6.3, default parameters) to convert genomic coordinates from HG002 paternal assembly to HG002 maternal assembly [40]. During this process, 72,651 STR regions could not be converted, resulting in the remaining 1,243,785 regions, of which 493,804 were confirmed to be monoallelic. Finally, STR regions whose flanking sequences with low *k*-mer diversity (same thresholds applied to CHM13 STR regions) and regions with coverage lower than 5 (the coverage threshold was reduced to account for the lower coverage of the HG002 dataset compared to the CHM13 dataset) were filtered out, resulting in the final set of 186,237 STR regions.

### Data processing and visualization

After downloading FAST5 files and POD5 files from sources specified by the authors, FASTQ files were obtained by employing the appropriate basecaller for each dataset (Supplementary Table S1). We aligned the FASTQ files to the reference genomes using minimap2 (v2.24-r1122) [41] with -ax map-ont parameters for all data except for SG-NEx data, where -ax splice was used instead. To reduce misalignments, we filtered out supplementary reads and reads with a mapping quality score below 60. We validated the effectiveness of this filtering step by realigning reads mapped to chromosome 21 back to the reference genome (Supplementary Fig. S1). Approximately 85% of the reads realigned to chromosome 21, while the remaining reads aligned elsewhere with markedly low mapping quality scores, close to 0. Filtering reads with mapping quality score below 60 resulted in 99.4% of accurately mapped reads. Subsequent data analysis and visualization were performed using Seaborn (0.13.0), Matplotlib (3.7.1), Pandas (2.0.0), and Numpy (1.22.4). All datasets analyzed in this study were PCR-free, ensuring the absence of PCR stutters. Moreover, all major datasets—CHM13 data, HG002, data and SG-NEx data—provided raw FAST5/POD5 files, allowing us to compare the influence of basecalling programs and their configurations on STR sequencing accuracy.

### Calculation of *k*-mer diversity

First, the frequency of each *k*-mer within the given DNA sequence was counted. The counting process involved sliding a window across the DNA sequence by 1 nucleotide at a time, extracting all possible *k*-mers and storing their frequency in a dictionary data structure. For example, if the DNA sequence is ATCGC, the 2-mer counting process produces the following Python dictionary: {AT: 1, TC:1, CG:1, GC:1}.

Then, the *k*-mer diversity was calculated using the following expression:


\begin{eqnarray*}
k - mer\,\,\textit{diversity} = \frac{{L - \left( {k - 1} \right)}}{{\mathop \sum \limits_{i = 1}^{L - 1} F_i^2}}
\end{eqnarray*}

where *L* is the length of the given DNA sequence ($L - ( {k - 1} )$ equals the maximum number of *k*-mers that can be found in the DNA sequence), and ${ {{F_1},\,\,{F_2},{F_3},\,\,{F_4}\,\,\ldots }}$ represents the frequency of each found *k*-mer.

### CNN prediction of sequencing accuracy using flanking sequences

The flanking sequences (6 nucleotides in each direction of the STR, resulting in 12 nucleotides) were one-hot encoded and converted into a Numpy array. STR loci with coverage below 40 were discarded, and a training/validation ratio of 9:1 was used with the remaining loci. Briefly, we used TensorFlow [42] to implement a sequential neural network featuring a 1-dimensional convolutional layer with 48 filters and a kernel size of 2, followed by a flattening layer and 2 dense layers with 120 and 40 nodes, respectively, both using ReLU activation. The output layer consisted of a single node with a sigmoid activation function. The model used the Adam optimizer and mean absolute error (MAE) as the loss function. Training was performed with 20 epochs with a batch size of 400, with data shuffled at the start of each epoch, ensuring that the model encounters a random order of data. Finally, we enabled early stoppage by monitoring validation loss with a patience of 10 epochs to prevent overfitting. Using this model, we predicted the sequencing accuracy of STR regions using the one-hot encoded flanking sequences as inputs. Linear regression was performed to assess the prediction results and visualized using Seaborn’s regplot function, while Pearson correlation values were calculated using SciPy (1.7.1).

### UMAP projection of STR regions

We considered a STR region’s sequencing accuracy to be well predicted if it satisfied the following expression:


\begin{eqnarray*}
\mu - \,\,\frac{1}{2}\sigma \le \left( {\textit{predicted}\,\,\textit{accuracy} - \textit{actual}\,\,\textit{accuracy}} \right) \le \mu + \,\,\frac{1}{2}\sigma
\end{eqnarray*}

where $\mu $ is the mean of differences between predicted and actual accuracies, and $\sigma $ is the standard deviation of these differences. The flanking sequences of these well-predicted STR regions were one-hot encoded and converted into an Anndata (v0.10.6) and subjected to UMAP visualization [43]. The following functions and parameters of Scanpy (1.10.0) were used: sc.pp.neighbors(adata, n_neighbors=15, n_pcs=18) and sc.tl.umap(adata, spread=1) [44].

### Identification of “top” and “worst” 20 flanking sequences of A-repeat STR loci

Confirming the association of flanking sequences of A-repeat STR loci and sequencing accuracy through CNN prediction and UMAP visualization, we identified “top” (i.e., demonstrating the best sequencing accuracy) 20 and “worst” (i.e., demonstrating the worst sequencing accuracy) 20 flanking sequences of A-repeat STR (Ax10–Ax14; 2 nucleotides in each direction, totaling 4 nucleotides) by calculating the average sequencing accuracy of STR loci of each length, with different flanking sequences. To ensure that the impact of a given flanking sequence on the sequencing accuracy was consistent, we only included flanking sequences that were present in more than 10 A-repeats of every length. For example, although the CG/CA flanking motif was found in more than 10 Ax10 repeats, it was not found in the A-repeat of other lengths and thus was not included in our analyses. The results were visualized using Seaborn’s heatmap function.

### Subset of A-repeat STRs that harbor low-complexity flankings

A subset of STRs that were previously excluded from the main analysis due to low-complexity flanking sequences (see “Identification of STR regions” in Methods) was reintroduced to validate the association between flanking sequence complexity and STR sequencing accuracy. Specifically, A-repeat STRs were selected if the Levenshtein distance between the STR sequence and either the left or right flanking sequence was below 7. For example, consider the following 2 A-repeat STR loci:

Loci A: 5′-AAAAAAAAAAAC (A)12 GCAATCCATACT-3′
Loci B: 5′-TTTTTTTTCCCC (A)12 GCAATCCATACT-3′

In locus A, although the Levenshtein distance between the right flanking sequence and the STR sequence is 8, the distance for the left flanking sequence is 1 and therefore excluded. In contrast, locus B has a Levenshtein distance of 12 between the left flanking sequence and the STR sequence and therefore included.

### Benchmarking NanoMnT, NanoRepeat, and NanoSTR

Six hundred 1-bp repeat STRs and six hundred 2-bp repeat STRs were incorporated. Unlike NanoRepeat and NanoMnT, NanoSTR requires the genomic coordinates of STR regions to be based on the hg38 assembly. Thus, we converted the genomic coordinates of STR regions from the HG002 maternal assembly to the hg38 assembly using LiftOff. After filtering out loci that could not be confidently converted, 712 loci remained for 1-bp repeat STRs and 619 loci for 2-bp repeat STRs. The STR loci used for benchmark are available in Supplementary Table S2. In-house scripts were used to summarize the outputs of each program.

### MSI detection of cancer sequencing datasets

Ax10–Ax14 STR loci with coverage above 30 were used for MSI identification, as mononucleotide repeats of these lengths have been shown to be vulnerable to deletion mutations in MMR-deficient cells [45]. For the SG-NEx dataset, loci that were not covered in at least 3 of 4 samples were discarded, while for the CRC organoid WGS dataset, loci that were not covered in at least 10 of 15 samples were discarded. For the “read + loci selection approach” , we filtered out (i) A-repeat STR loci that had guanine nucleotides directly next to the A-repeat tracts and (ii) loci whose allele prominence (calculated by NanoMnT) satisfied the following expression:


\begin{eqnarray*}
\mu - \,\,\frac{1}{2}\sigma \le \textit{allele}\,\,\textit{prominence} \le \mu + \,\,\frac{1}{2}\sigma
\end{eqnarray*}

where μ is the mean of allele prominences of all genotyped loci, and σ is the standard deviation of these prominences.

Subsequently, we obtained the allele size histogram and calculated the relative allele size of each STR locus using the following expression:


\begin{eqnarray*}
\mathop \sum \limits_{i = 1}^n \left( {{A_i} - {R_i}} \right){f_i}
\end{eqnarray*}

where ${A_i}$ is the observed allele, ${R_i}\,\,$ is the reference allele (CHM13), and ${f_i}$ is the frequency of ${A_i}$. The STR loci used for MSI detection are available in Supplementary Tables S3 and S4.

### Implementation of NanoMnT

NanoMnT provides 3 functions: (i) error correction of reads, (ii) STR allele size estimation, and (iii) informative loci identification.

1. Error correction of individual reads
  NanoMnT collects reads that aligned to the user-provided STR loci using Pysam (v0.20.0) [46] and realigns them to a modified STR region that excludes the STR sequence itself, consisting only of the STR-flanking regions. This approach prevents alignment bias caused by the reference genome, as minimap2 tends to produce slightly different alignments in STR regions, depending on the STR sequence length. For each realigned read, the sequences that aligned to the STR regions are extracted and compared against a list of possible alleles by calculating the Levenshtein distance. When defining the list of possible alleles (i.e., number of repeats), NanoMnT assumes that the allele for a given aligned read falls between 0 repeats (lower bound) and the longest allele among all the reads (determined by counting the number of repeats prior to error correction) plus 5 additional repeats (upper bound). The 5 additional repeats serve as a buffer to account for potential outliers. The allele with the minimum Levenshtein distance is chosen as the most likely allele. If the total Levenshtein distance exceeds a certain threshold, the read is considered excessively erroneous and discarded. This process yields corrected STR alleles for each ONT read, which are then used for subsequent STR allele size estimation.
2. Estimation of STR allele size
  Using the corrected reads, NanoMnT creates an allele size histogram for each locus. The user can decide whether to use all reads or forward/reverse strand reads—which is very beneficial when analyzing A-/T-repeats—when creating allele size histogram. To estimate the STR allele size, NanoMnT generates synthetic allele size histograms for each possible allele and calculates the distance between the observed allele size histogram against each synthetic allele size histogram. The synthetic histogram with the minimum distance to the observed histogram is then chosen as the best match. The allele associated with this chosen histogram is selected as the most probable STR allele. Finally, SciPy’s find_peak function is used to calculate the prominence of the observed allele size histogram.
3. Informative loci identification
  Given the outputs of NanoMnT (Allele Table and Locus Table, see Fig. 8A) of paired normal and tumor samples, NanoMnT finds STR loci that have been sequenced in both samples (namely, commonly covered loci) and compares the STR allele size histogram by calculating the distance between the 2 histograms. This distance information tells us about the similarity between the STR allele size histogram of 2 samples; if the similarity is low, this locus may be an indication of MSI phenotype. Lastly, the “score” of each STR locus is calculated using the following expression: $locus\,\,\textit{score} = \textit{distance}( {{H_n},\,\,{H_t}} )\,\, \times \textit{Peak}\,\,\textit{prominenc}\,\,of\,\,{H_n}$, where ${H_n}$ and ${H_t}$ are the allele size histogram of normal and tumor samples, respectively. This score informs the reliability of each result.

## Results

### Distribution of sequencing errors in STR regions

The STR regions analyzed in this study were carefully selected because many exhibited excessively low-complexity sequences in their flanking regions, which often introduce alignment bias and hampers downstream analyses (Methods). The number and distribution of STRs analyzed in this study are available in Supplementary Fig. S2A. Note that GC-rich STR could not be robustly represented in our analyses due to the scarcity of GC-rich STR in the human genome (Supplementary Fig. S2B).

**Figure 1:**
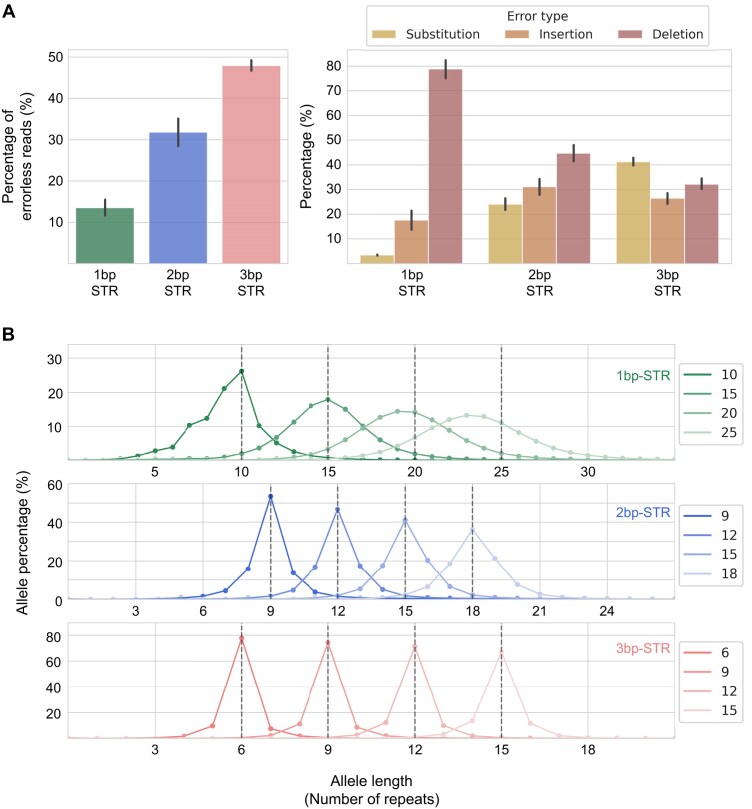
Distribution of ONT sequencing error in STRs. (A) Percentage of errorless reads in 1-bp, 2-bp, and 3-bp repeat STRs (left) and distribution of sequencing error types (right). The 1-bp repeats with 10 to 30 repeats, 2-bp repeats with 7 to 24 repeats, and 3-bp repeats with 5 to 15 repeats were analyzed. (B) STR allele size histograms of various lengths of 1-bp, 2-bp, and 3-bp repeat STRs.

We first measured the abundance of each type of sequencing error (deletions, insertions, and substitutions) by counting the number of errors in ONT reads that aligned to the STR regions (Fig. 1A). Overall, the error rates were higher in STRs with shorter repeat units, with 1-bp repeats exhibiting the highest rate. Indel errors accounted for most of the sequencing errors (96%, 73%, and 55% for 1-bp, 2-bp, and 3-bp repeats, respectively). Among these, deletion errors were the most prevalent, particularly in 1-bp repeats, where 59% of reads (14,989,206 out of 25,392,291) contained at least 1 deletion, aligning with previous reports [20, 22, 24].

To provide a more practical analysis of STR error profile, we performed rudimentary polishing of sequencing errors using in-house scripts, as doing so considerably increased the number of reads that can be analyzed. This was achieved by calculating the Levenshtein distance between the observed STR sequence and a list of possible STR sequences, then selecting the STR sequence with the minimum distance. Reads with distances exceeding 4, which constituted approximately 2.8% of the total reads, were discarded. Using these polished reads, we visualized the distribution of STR allele sizes by generating histograms for various STR alleles. We observed that ONT tended to underestimate STR sizes, causing some histograms to shift slightly leftward (Fig. 1B, Supplementary Fig. S3). While many histograms exhibited clear peaks that matched the actual STR alleles, prominent peaks could not be generated for 1-bp repeats.

### Sequencing accuracy of STR across different repeat units and lengths

**Figure 2:**
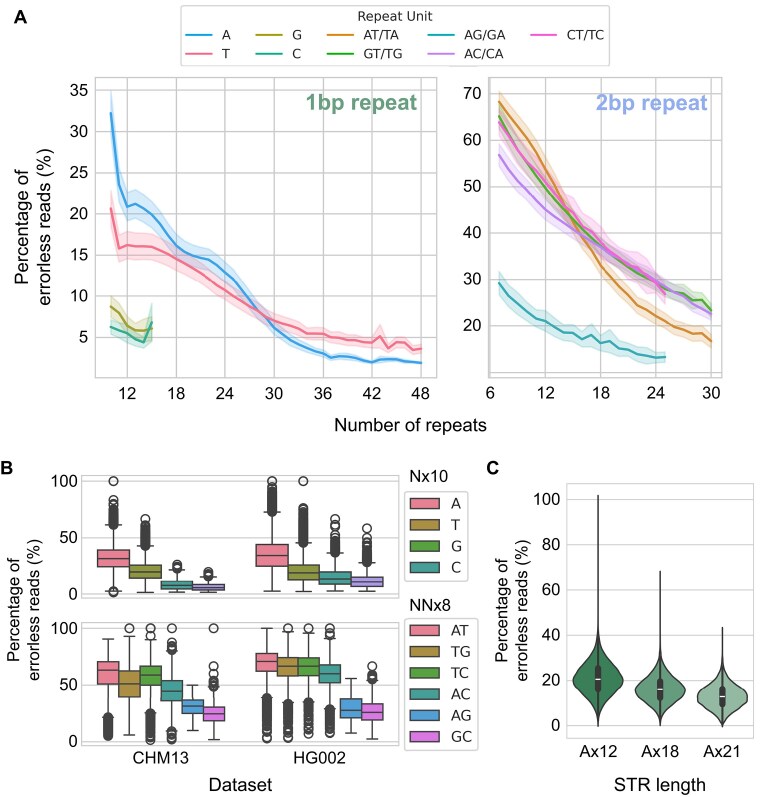
Sequencing accuracy of STRs based on their repeat units. (A) Sequencing accuracy (measured by calculating the percentage of errorless reads) of varying lengths of 1-bp repeat STRs (left) and 2-bp repeat STRs (right) based on their repeat units. (B) Sequencing accuracy of Nx10 and NNx8 STRs of various repeat units, compared between 2 separate datasets. (C) Distribution of sequencing accuracy of Ax12, Ax18, and Ax21 STRs, demonstrating the extreme variability of sequencing accuracy among identical types of STRs.

Next, we measured ONT sequencing accuracy for different STR types. In this study, we define *sequencing accuracy* as the percentage of errorless reads (i.e., reads that do not contain any sequencing errors within the STR sequence), terms we will use interchangeably throughout the article. When conducting this analysis, we made an important consideration. Given that only 1 strand of the double-stranded DNA enters the nanopore, the error profiles generated by the 2 different orientations of reads are probably different. For example, the error profile of forward strand reads originating from an A-repeat STR locus (reads that map to the forward strand of the reference genome) may differ from that of reverse strand reads from the same locus (reads that map to the reverse strand of the reference genome). This is because the former set of reads encompasses the sequencing of A-repeats, while the latter encompasses the sequencing of T-repeats. Thus, we calculated the sequencing accuracy for each type of STR, based on their lengths and the repeat units that were actually sequenced by the nanopore. As a result, we found that the sequencing accuracy varied substantially among different types of STR (Fig. 2A). Among 1-bp repeats, A-repeats were generally better sequenced than other 1-bp repeats, whereas in 2-bp repeats, AT/TA-repeats were better sequenced than other 2-bp repeats. However, we emphasize that this trend only applies in a general sense, as there are considerable exceptions (Supplementary Fig. S4). We validated this hierarchy of sequencing accuracy among repeat units using the HG002 R9.4.1 dataset (Fig. 2B). For STRs with longer repeat units, most STRs displayed much better accuracy compared to 1-bp and 2-bp repeat STRs (Supplementary Fig. S5), although it should be noted that their scarce nature limited our analysis to their relatively shorter forms. Moreover, we noticed a substantial variability of sequencing accuracy among STRs, even those with identical repeat units and lengths (Fig. 2C).

### Relationship between STR sequencing accuracy and flanking sequences

**Figure 3:**
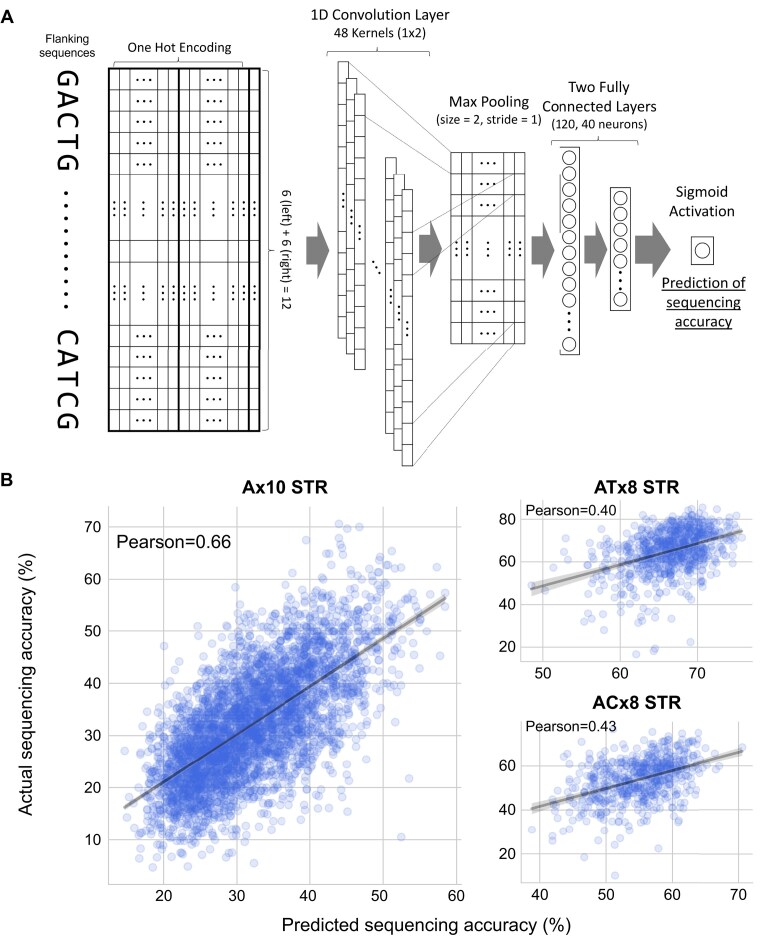
CNN-based machine learning prediction of STR sequencing accuracy using flanking sequences. (A) Illustration of CNN-based machine learning prediction workflow. (B) Prediction results of Ax10 STR (left), ATx8 STR (upper right), and ACx8 STR (lower right) sequencing accuracy.

To explain the variability in sequencing accuracy among identical STR types (i.e., STRs with same repeat unit and same length) (as shown in Fig. 2C), we hypothesized that the flanking sequences of STRs may influence the sequencing accuracy. We tested this hypothesis by training a convolutional neural network (CNN) machine learning model using the flanking sequences of Ax10 STR regions (6 nucleotides for each direction, totaling 12 nucleotides) to predict the sequencing accuracy of Ax10 STR regions (*n* = 39,594) (Fig. 3, Methods). The model displayed considerable predictive accuracy, as shown by the Pearson correlation value of 0.66 (Fig. 3B). Repeating the same process with either left or right flanking sequences yielded markedly lower Pearson correlation values, suggesting that flanking sequences influence the STR sequencing accuracy in both directions (Supplementary Fig. S6A). The STR accuracy of 2-bp repeats such as ATx8 (*n* = 7,660) and ACx8 (*n* = 5,916) repeats was also moderately predicted by our model, indicating that the association between sequencing accuracy and flanking sequences extends beyond Ax10 to other STR types. However, outliers in the CNN prediction suggest that flanking sequences alone do not fully determine the sequencing accuracy of STR regions. Although STR regions with identical flanking sequences generally exhibit similar sequencing accuracies, noticeable variation remained evident (Supplementary Fig. S6B).

**Figure 4:**
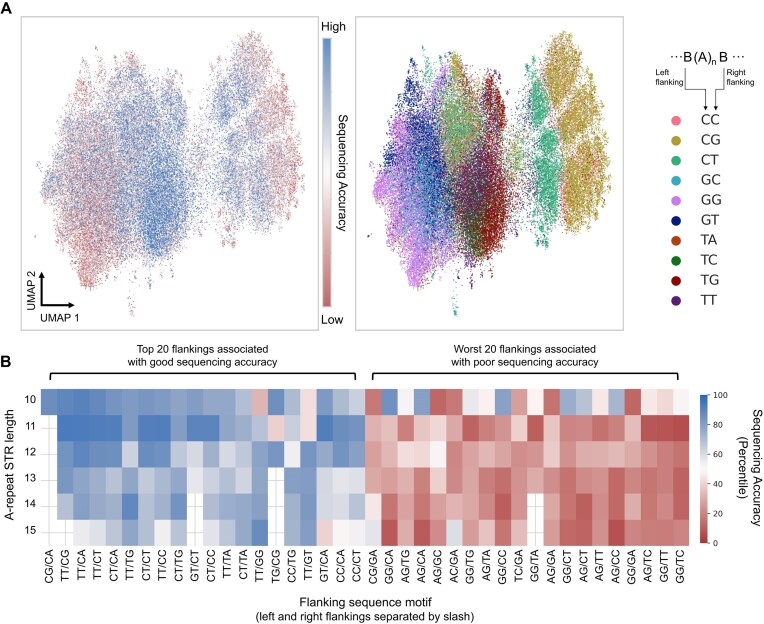
Identification of motifs associated with good/bad sequencing accuracy of A-repeat STRs. (A) UMAP projection of flanking sequences of Ax10–Ax15 repeat STRs. Each dot represents an A-repeat STR locus, colored by its sequencing accuracy (left) and by its most adjacent flanking nucleotides (right). (B) Flanking sequences of A-repeat STRs (2 nucleotides in each direction, 4 nucleotides total) associated with good and bad sequencing accuracy. Two nucleotides in each direction are separated by slash (e.g., CT/CG motif indicates CT-(A)_n_-CG).

Nonetheless, motivated by this finding, we identified motifs associated with high or low sequencing accuracy within the CNN model. We selected Ax10–Ax15 STR regions whose sequencing accuracies were well predicted by the CNN model and applied UMAP using the flanking sequences as features (Methods, Fig. 4A). Mapping sequencing accuracy onto the UMAP revealed an interesting pattern: the sequencing accuracy appeared to be primarily with the nucleotides closest to the A-repeats. Notably, A-repeats flanked by 2 guanine nucleotides consistently exhibited poor sequencing accuracy. Furthermore, the distance between the flanking nucleotides and the A-repeats appeared to be inversely proportional to their influence on sequencing accuracy (Supplementary Fig. S7A). We identified the top 20 motifs and the worst 20 motifs of A-repeats (defined by the 2 flanking nucleotides on each side) whose effects on sequencing accuracy were relatively consistent across varying lengths of A-repeats (Fig. 4B, Methods). Top motifs were enriched with pyrimidine bases, while the worst motifs were enriched with purine bases (Supplementary Fig. S7B). These results were validated using the HG002 R9.4.1 dataset (Supplementary Fig. S7C). Finally, to address the potential bias introduced by filtering STR regions with low-complexity flanking sequences, we repeated the same analysis using these STR regions and obtained similar results (Supplementary Fig. S8, Methods).

### The impact of basecaller on STR sequencing accuracy

**Figure 5:**
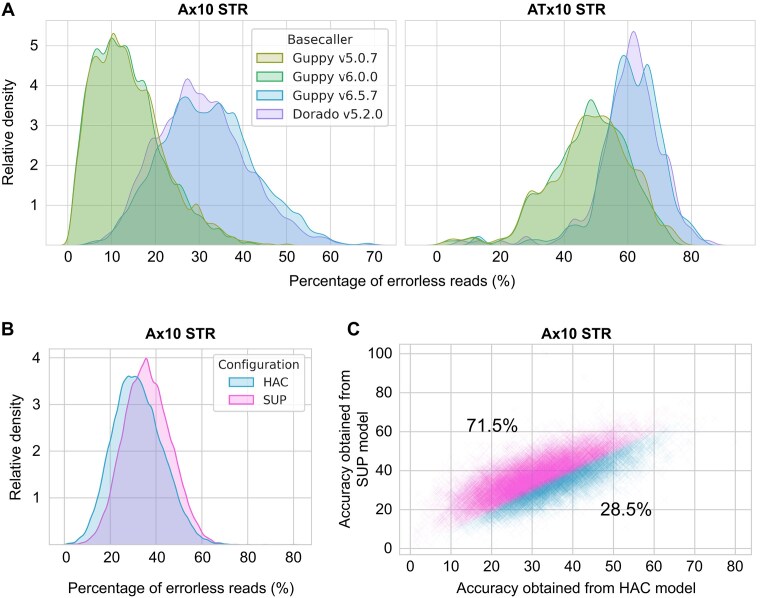
Influence of basecaller on STR sequencing accuracy. (A) Comparison of 4 ONT basecallers in basecalling Ax10 STRs (left) and ATx10 STRs (right), visualized with kernel density estimate plots. (B) Comparison of the HAC model and SUP model in basecalling Ax10 STRs, visualized with kernel density estimate plots. Both models are from Guppy v6.5.7. (C) Sequencing accuracy of Ax10 STRs obtained by the HAC basecaller model (x-axis) and the SUP basecaller model (y-axis), where each cross represents a single Ax10 STR locus. Loci that exhibited better sequencing accuracy with the SUP basecaller model (71.5%) or HAC basecaller model (28.5%) were marked with different colors.

A unique aspect of ONT data analysis is the basecalling process, which uses machine learning to convert electric signals into nucleotide sequences. Basecallers are regularly updated, allowing users to reanalyze their data using different versions. To explore the impact of basecallers on STR sequencing accuracy, we compared the performance of 4 basecaller versions—Guppy v5.0.7, Guppy v6.0.0, Guppy v6.5.7 (the final Guppy version), and Dorado v5.2.0—by comparing the sequencing accuracy of Ax10 STR (*n* = 1,552) and ATx10 STR (*n* = 102) located in chromosome 1 of the T2T-CHM13 genome (Fig. 5A). The HAC model was used for all 4 basecaller versions. Guppy v6.5.7 and Dorado v5.2.0 exhibited similar performances and vastly outperformed the other 2 versions, highlighting the importance of using the most up-to-date basecaller. Next, we compared the influence of the HAC model against the super accuracy (SUP) model of Guppy v6.5.7 and observed considerable improvements (Fig. 5B). Given that the SUP model is known to offer only marginal improvements over the HAC model, this improvement was unexpectedly significant. However, this improvement was not uniform; while 71.5% of Ax10 STR regions were better resolved using the SUP model, the remaining 28.5% regions were better resolved with the HAC model (Fig. 5C). Notably, UMAP analysis of these loci revealed no segregation based on the basecalling model that best resolved each locus, suggesting that flanking sequences do not determine the better model (Supplementary Fig. S9). Nevertheless, in general, choosing the latest version and model provides significant benefit for STR analysis.

### Base quality score of sequencing error in STR regions

**Figure 6:**
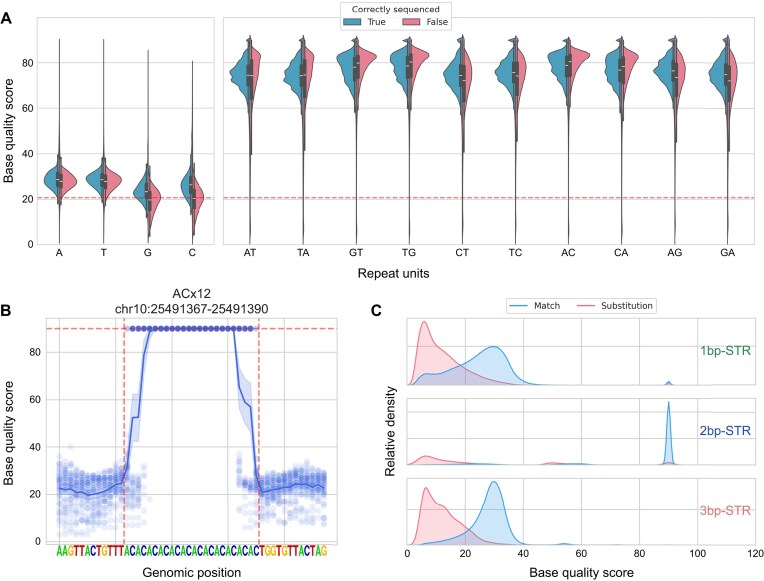
ONT base quality scores in STR regions. (A) Base quality score comparison between correctly sequenced reads and reads containing sequencing error across various STR types. The red dashed horizontal line indicates the estimated base quality average across the entire CHM13 dataset (20.7). (B) Base quality score distribution in an ACx12 STR locus (chr10:25,491,367–25,491,390, T2T-CHM13 v2.0). Each dot represents the base quality score reported by a single read, and the line represents the average score of each genomic position. (C) Base quality score comparison of correctly sequenced bases and substitution error bases, visualized with kernel density estimate plots.

Next, we examined whether the elevated error rates in STR regions were reflected in the base quality. First, we compared the average base quality scores of correctly sequenced reads against incorrectly sequenced reads and noted marginal differences (Fig. 6A). However, we observed a significant overestimation of the base quality scores of bases within the STR regions, regardless of the presence of errors (the average base quality score within the entire CHM13 dataset was approximately 20.67). Upon investigating the distribution of base quality scores of bases within and adjacent to STR regions (Supplementary Fig. S10A), we observed bursts of quality scores to abnormally high values. While this phenomenon was observed in 1-bp, 2-bp, and 3-bp repeat STRs, it was most evident in 2-bp repeat STRs. For the majority of 2-bp repeat STRs, the basecaller consistently assigned a fixed value of 90 as the base quality score for bases within the STR regions. Figure 6B shows the base quality score distribution within an ACx12 STR locus (chr10:25,491,367–25,491,390, T2T-CHM13v2.0), which demonstrates the typical base quality score distribution within 2-bp repeat STR regions. Notably, such quality score bursts were not observed in 4-bp, 5-bp, and 6-bp repeat STR.

We also examined the base quality scores of sequencing errors to assess their potential utility in sequencing error inference. The overall base quality scores of substitution errors within the STR regions were markedly lower than those of the correct bases (Fig. 6C). In contrast, the differences between base quality scores between correctly sequenced reads and reads harboring indel errors were unnoticeable, which was disappointing, considering that indel errors accounted for most sequencing errors (Supplementary Fig. S10B). We validated these findings by repeating the same analysis on the HG002 R9.4.1 dataset (Supplementary Fig. S11).

### STR error profile of R10.4.1 flowcell

**Figure 7:**
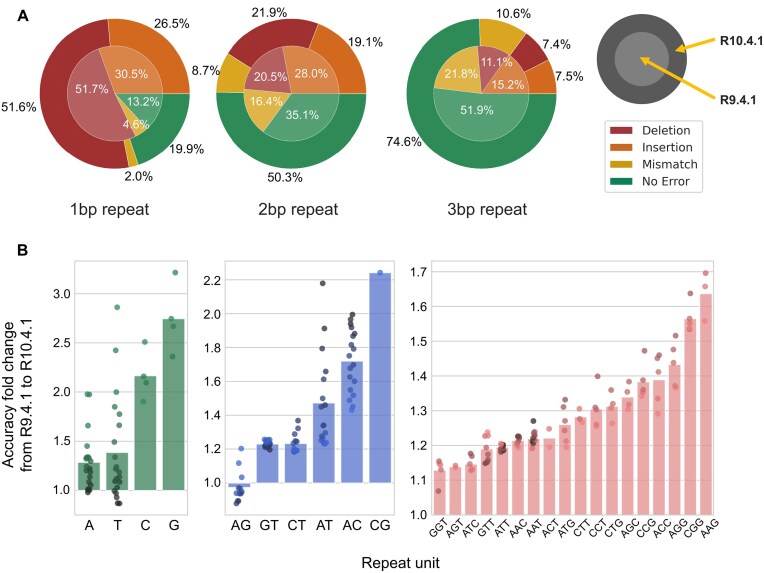
Comparison of ONT STR sequencing profile between the R9.4.1 and the R10.4.1 flowcell. (A) Distribution of correctly sequenced reads and various types of sequencing errors in 1-bp, 2-bp, and 3-bp repeat STRs, compared between the R9.4.1 (HG002 R9.4.1 dataset) and the R10.4.1 (HG002 R10.4.1 dataset) flowcell. (B) Changes in sequencing accuracy from R9.4.1 to R10.4.1 of various types of STRs. Accuracy change (y-axis) is measured by dividing the sequencing accuracy observed in R9.4.1 with the sequencing accuracy of R10.4.1 (e.g., 2.0 indicates 200% improvement). The dots in each bar plot represent STRs of different lengths, with longer STRs represented by darker colors.

We expanded our analysis by comparing the HG002 R10.4.1 dataset with the HG002 R9.4.1 dataset, incorporating reads that mapped to 1-bp, 2-bp, and 3-bp repeat STRs. The error profile of R10.4.1 resembled that of R9.4.1 (Fig. 7A). Although indel errors still accounted for most sequencing errors (91%), R10.4.1 demonstrated significant improvement over its predecessor across nearly all 3 STR types, particularly for the GC-rich STR (Fig. 7B). We performed similar analyses performed throughout the study on the HG002 R10.4.1 dataset and show that all the topics discussed in this article—sequencing accuracy of various STR types, impact of basecallers, and the association of flanking sequences with sequencing accuracy—are largely maintained in R10.4.1 as well (Supplementary Figs. S12–S14). Notably, unlike with R9.4.1 (Fig. 5C), the advantages of the SUP model over the HAC model (Dorado v8.1.0) were more prominent and consistent, with 92.99% of Ax12 STR loci showing improvements (Supplementary Fig. S12B).

### Development of NanoMnT

**Figure 8:**
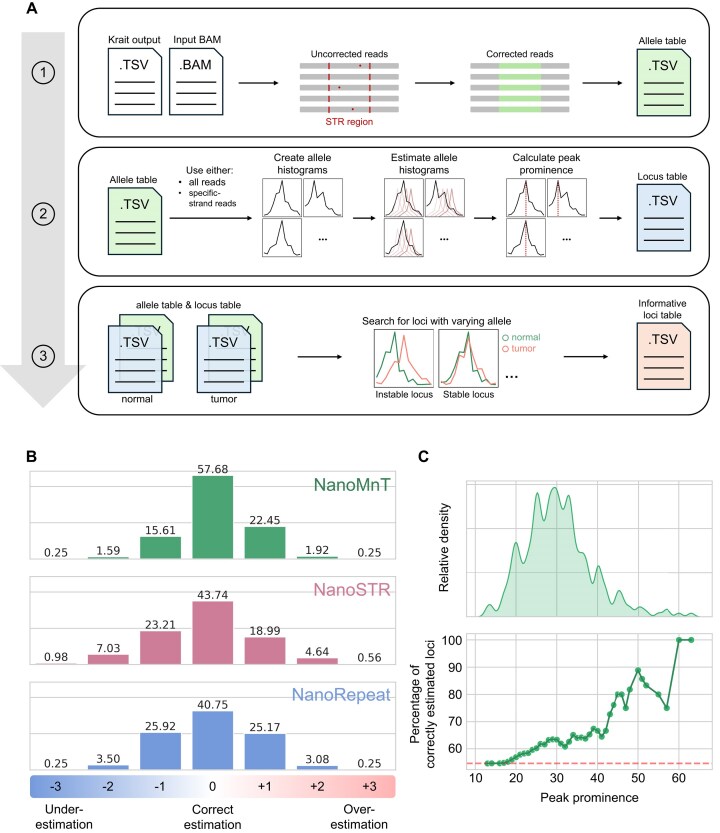
Development of NanoMnT. (A) Schematic overview of NanoMnT functionality. First, NanoMnT performs rudimentary STR error correction in read level and generates a tab-delimited file (TSV) named Allele Table. The Allele Table is then used to estimate STR allele sizes of user-specified STR loci by comparing the observed STR allele size histogram with many synthetic STR allele size histograms, where the synthetic histogram with the most resemblance is chosen as the putative allele histogram. This process generates another TSV file, called the Locus Table. Given the Allele Table and the Locus Table of paired normal and tumor samples, NanoMnT compares the STR loci captured in both samples to search for loci that may provide useful information regarding the tumor’s MSI status. (B) Benchmark results of NanoMnT, NanoSTR, and NanoRepeat in estimating STR allele sizes of three hundred 1-bp repeat STR loci. (C) Distribution of peak prominence value (which indicates the prominence of the STR allele size histogram of each STR locus, calculated using SciPy *find_peaks* function) reported by NanoMnT (top) and the change in percentage of STR loci whose allele size has been correctly estimated by thresholding peak prominence (bottom). For example, ∼90% of STR loci whose peak prominences exceed 50 are correctly estimated. The red dashed horizontal line represents the total percentage of STR loci that have been correctly estimated by NanoMnT, as shown in B.

Although existing STR analysis tools discussed in the Introduction section excel in genotyping 3-bp, 4-bp, 5-bp, and 6-bp repeats, they are not designed for analyzing 2-bp and, especially, 1-bp repeats. Thus, we developed NanoMnT, a lightweight Python-based tool that (i) corrects STR sequencing errors for ONT reads, (ii) estimates allele sizes of user-specified STR loci using the corrected reads, and (iii) searches for informative STR loci given the output files for paired normal and tumor samples (Methods, Fig. 8A). Using the HG002 R9.4.1 dataset, we tested the performance of NanoMnT against NanoRepeat and NanoSTR and confirmed that NanoMnT provides better STR allele size estimation for monoallelic 1-bp repeats and 2-bp repeats (Fig. 8B, Supplementary Fig. S15A). NanoMnT provides the prominence of the allele histogram peak, which can be used as a quality measure, with high peak prominence generally corresponding to confident allele estimation results (Fig. 8C, Supplementary Fig. S15B). When estimating Ax10 repeats, selectively using reads based on their sequencing orientation (e.g., forward strand reads for A-repeat STRs and reverse strand reads for T-repeat STRs) improves accuracy from 55% (without read selection) to 78% (Supplementary Fig. S15C). This accuracy can be further increased to 85% by excluding loci flanked by guanine nucleotides. These results highlight the practicability of our findings.

**Figure 9:**
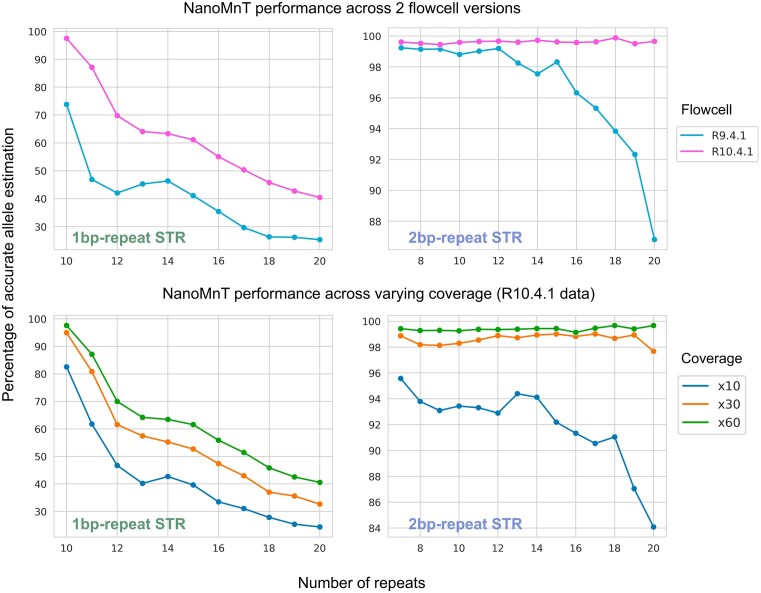
NanoMnT performance across flowcell versions and coverage. Percentage of correct allele estimation for 1-bp repeat and 2-bp repeat STRs by NanoMnT across varying sequencing coverages and flowcell types. The HG002 R9.4.1 dataset and the HG002 R10.4.1 dataset were employed for this benchmark. The upper 2 plots compare NanoMnT performance between the 2 flowcells, while the lower 2 plots illustrate the impact of sequencing coverage (10×, 30×, 60×) on allele estimation accuracy.

Next, we evaluated the impact of sequencing coverage and the flowcell version on NanoMnT performance by using inputs of varying sequencing parameters (Fig. 9). The HG002 R9.4.1 and HG002 R10.4.1 datasets were used to compare the 2 flowcell versions. STR allele estimation with the R10.4.1 data produced more accurate results for both 1-bp repeat STR (44% more accurate on average) and 2-bp repeat STR (23% more accurate on average), reflecting the advancements introduced by the R10.4.1 flowcell and the V14 chemistry. This improvement was especially pronounced in 2-bp repeat STR regions, where R10.4.1 maintained consistently high accuracy even for longer repeat lengths. Coverage was also an important factor that affected allele estimation results for R10.4.1 data. As expected, higher coverage led to more accurate results.

### MSI detection of cancer samples from ONT data using NanoMnT

**Figure 10:**
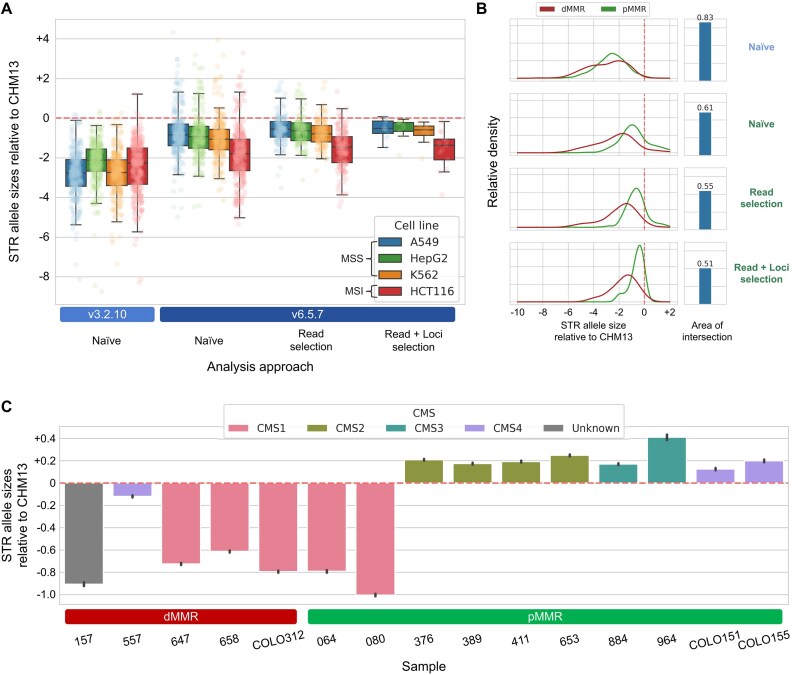
MSI identification results of SG-NEx and CRC organoid WGS sequencing data. (A) Distribution of STR allele sizes relative to those of the CHM13 genome in 4 cancer cell lines, visualized by box plots and strip plots. Four analysis approaches are compared: naive (Guppy v3.2.10), naive (Guppy v6.5.7), read selection approach, and read + loci selection approach. (B) Comparison of STR allele size distributions between the MSS cell lines and the MSI cell line when employing the 4 different analysis approaches, visualized by kernel density estimate plots (left) and the area of intersection between the kernel density estimate plots of MSS and MSI. The red dashed vertical lines in the left figure represent the reference STR allele size (zero). (C) Average STR allele sizes of 15 CRC samples visualized by bar plots, with each sample colored by its reported CMS type.

We integrated our findings into a biological context by identifying MSI status of cancer samples from the bulk RNA sequencing dataset created by SG-NEx [36]. Among the many types of ONT sequencing datasets provided by SG-NEx, we chose the PCR-free direct cDNA sequencing data to ensure the absence of PCR stutter. Conventionally, the MSI status is determined by comparing the STR allele size histograms of the tumor sample with those of the corresponding normal sample. Unfortunately, due to the lack of matched normal sample, we used the CHM13 genome as the substitute normal sample. We calculated the relative allele sizes of STR loci and compared their distribution among samples to identify MSI and microsatellite stable (MSS) cancers (Methods). To showcase the importance of bioinformatics strategies for analyzing ONT data, we compared the MSI identification results derived from 3 distinct versions of FASTQ data obtained from the same sample: (i) raw FASTQ files provided by SG-NEx, which were basecalled using Guppy version 3.2.10; (ii) data re-basecalled using the latest version of Guppy, version 6.5.7 (HAC); and (iii) data re-basecalled using Guppy version 6.5.7 (HAC), while applying read selection and the STR loci selection process to achieve better accuracy (Fig. 10A, Methods). Overall, the STR allele sizes of the MSI cell line were shorter than those of the MSS cell lines, aligning with previous reports that deletion mutations predominate in mononucleotide repeats within MSI [45]. While simply re-basecalling the data with Guppy v6.5.7 was enough in separating the STR allele profiles of the MSI cell line from those of the MSS cell lines, the read selection and/or loci selection process gave markedly better results (Fig. 10B).

We performed a similar analysis on the CRC organoid dataset created by Pickles et al. [37], who conducted WGS on 15 primary CRC organoids, each labeled with MMR status and consensus molecular subtype (CMS) (Fig. 10C). Although we could not re-basecall this dataset with the latest basecaller because the raw FAST5/POD5 files were unavailable, we still showed that dMMR status could be readily identified, except for samples 064 and 080. Although these 2 discordant results may be false positives, they could also reflect the intratumoral heterogeneity of CRC. Indeed, several studies have reported coexistence of CMS1—which is almost exclusively enriched in MSI CRC—and other CMS CRC within individual patients [47–49].

## Discussion

The capacity of ONT to generate long reads, along with its portability and versatility, makes it an attractive approach in many research fields. However, the high error rate of ONT in low-complexity regions hinders its application in STR-related fields. This study provides a comprehensive overview of ONT sequencing profiles in STR regions by measuring the abundance of sequencing errors in various STR types and identifying factors that influence STR sequencing accuracy. Indels were responsible for most sequencing errors, with deletions being more prevalent than insertions. In addition, we observed a substantial overestimation of base quality scores in STR regions, which may suggest that the basecaller machine learning models are not properly tailored for STR regions. While base quality scores of substitution error bases and correct bases differed significantly—suggesting the potential of base quality score in inferring substitution errors—we did not observe such difference between indel errors and correctly sequenced bases, which is unfortunate, considering the abundance of indel errors. We also compared the STR error profiles between the R10.4.1 and R9.4.1 flowcells and observed a significant improvement in the R10.4.1 flowcell. Although the overall frequency of each type of sequencing error was similar between the 2 flowcells (with indels comprising the majority), the total number of errors was markedly reduced in the R10.4.1 flowcell. Consequently, as shown in Fig. 9, allele estimation accuracy for both 1-bp repeat and 2-bp repeat STR loci was substantially enhanced.

In this study, we identified 3 factors that influence the ONT sequencing accuracy of STR. First, the sequencing accuracy of STRs was heavily influenced by the repeat unit of the sequenced STR, specifically the repeat units that entered the nanopores. This finding suggests a strategic approach when analyzing STR from ONT data: preferential usage of reads with specific orientation over reads with opposite orientation may achieve superior accuracy, given that the sequencing depth is sufficiently high. This finding was consistently observed in both versions of flowcells (R9.4.1 and R10.4.1) and basecalling programs, indicating that the electric signals associated with some repeat units may be intrinsically more resolvable for ONT compared to others. Second, flanking sequences were also associated with the sequencing accuracy of STR regions, implying that careful selection of STR loci based on their flanking sequences may help mitigate the high error rate of ONT. For example, A-repeats with purine-rich flanking sequences were linked to worse sequencing accuracy compared to A-repeat with pyrimidine-rich flanking sequences. This could be due to the high similarity of electric signals produced by the A-repeats and the purine-rich flanking sequences. Third, we highlighted the significance of the basecaller version, which is possibly the most influential factor of sequencing accuracy, as shown in Fig. 5. Therefore, we encourage researchers who have previously generated ONT sequencing data to re-basecall their data using the latest basecaller for STR-related analyses such as MSI identification.

We introduced NanoMnT, a lightweight Python-based tool that performs error correction in STR regions by choosing the most parsimonious allele (i.e., allele with the minimum Levenshtein distance compared to the observed allele) and genotyping STR regions using these corrections. Although existing tools serve similar purposes, none of them have been designed to genotype 1-bp and 2-bp repeat STR. Instead, to the best of our knowledge, most tools are designed to genotype STR with longer repeat units to study areas such as neurological disease [34] and forensics [31]. Benchmarking analyses demonstrate that NanoMnT provides superior STR allele estimation for monoallelic 1-bp and 2-bp repeat STR loci. By applying NanoMnT on 2 cancer datasets, we were able to identify MSI status of various cancer samples. However, we acknowledge a major caveat of NanoMnT: NanoMnT lacks the capability to phase multiple alleles, making it unsuitable for analyzing polyallelic STR loci. If heterozygosity is expected, we encourage users to examine NanoMnT output metrics (e.g., peak prominence or allele histogram visualizations) or to use a different tool capable of detecting heterozygotic STR alleles, such as NanoRepeat, NanoSTR, or WarpSTR.

We acknowledge several limitations of this study. First, our study was solely focused on perfect tandem repeats, excluding many types of repeats such as compound repeats and imperfect tandem repeats. Future studies will be needed to assess the performance of ONT in analyzing these types of repeats. Second, although we identified certain motifs that are enriched in well-/poorly sequenced A-repeat STR, we failed to provide a comprehensive mechanism that explains the influence of flanking sequences on STR sequencing accuracy. Also, the CNN machine learning model did not exhibit optimal predictive accuracy, indicating the presence of additional factors that we could not detect and/or the stochastic nature of ONT error profile. We note that the lack of diversity of flanking sequences within the human genome—since a major portion of A-/T-repeat STR originates from mobile genetic elements such as Alu elements—may have exacerbated the CNN prediction results. Thus, using a sufficiently diverse set of flanking sequences may improve our understanding of the association between flanking sequences and sequencing accuracy. Lastly, while NanoMnT outperforms existing tools in estimating 1-bp repeat and 2-bp repeat STR loci, its overall accuracy remains suboptimal for 1-bp repeat STRs. This limitation is expected to improve as ONT continues to update its flowcells and enhance sequencing accuracy.

## Availability of Source Code and Requirements

Project name: NanoMnT

Project homepage: https://github.com/18parkky/NanoMnT

Operating systems: Tested on Ubuntu, CentOS 7, and macOS (Sonoma 14.1.2)

Programming language: Python

Other requirements: Python 3.x, Matplotlib>3.7.1, Numpy>1.20.3, Pysam>0.20.0, Pandas>2.0.0, Scipy>1.7.1, Seaborn>0.13.0

License: MIT

RRID: RRID:SCR_026210

bio.tools ID: nanomnt

## Supplementary Material

giaf013_Supplemental_Files

giaf013_GIGA-D-24-00346_Original_Submission

giaf013_GIGA-D-24-00346_Revision_1

giaf013_GIGA-D-24-00346_Revision_2

giaf013_Response_to_Reviewer_Comments_Original_Submission

giaf013_Response_to_Reviewer_Comments_Revision_1

giaf013_Reviewer_1_Report_Original_Submission Qian Chris Liu -- 9/11/2024

giaf013_Reviewer_1_Report_Revision_1 Qian Chris Liu -- 1/10/2025

giaf013_Reviewer_2_Report_Original_Submission Rupesh Kesharwani -- 9/24/2024

giaf013_Reviewer_2_Report_Revision_1 Rupesh Kesharwani -- 1/14/2025

giaf013_Reviewer_3_Report_Original_Submission Roxanne Richelle Zascavage, Ph.D. -- 10/1/2024

giaf013_Reviewer_3_Report_Revision_1 Roxanne Richelle Zascavage, Ph.D. -- 1/13/2025

## Data Availability

The sequencing dataset generated for CHM13 was accessed via the Telomere-to-Telomere consortium CHM13 project GitHub page [50]. Sequencing datasets for HG002 were accessed through the Dataset Releases made available by EPI2ME [51, 52]. The SG-NEx RNA-seq dataset was accessed through the SG-NEx GitHub page [53], and the WGS dataset of CRC organoids generated by Pickles et al. was downloaded from NCBI (PRJNA978372). Other data further supporting this work are openly available in the *GigaScience* repository, GigaDB [54]. DOME-ML (Data, Optimisation, Model, and Evaluation in Machine Learning) annotations supporting the current study are available in the DOME Registry [55]. A snapshot of the GitHub repository [56] has also been archived in Software Heritage [57].

## Additional Files

**Supplementary Fig. S1**. (A) Percentage of chromosomes which reads (of the CHM13 dataset, whose primary alignments aligned to chromosome 21) realigned to. (B) Mapping quality distribution of 2 groups of the realigned reads: reads that mapped back to chromosome 21 and reads that mapped elsewhere. (C) Distribution of the number of alignments per read. For example, 1 indicates that a read aligned to a single region, and 2 indicates that a read aligned to 2 different regions.

**Supplementary Fig. S2**. (A) Number of STRs analyzed in this study. (B) Percentage of 1-bp/2-bp repeat STRs by repeat units.

**Supplementary Fig. S3**. STR allele size histograms of various lengths of 4-bp, 5-bp, and 6-bp repeat STR.

**Supplementary Fig. S4**. Distribution of forward strand sequencing accuracy (i.e., sequencing accuracy calculating using only forward strand reads) minus reverse strand sequencing accuracy in Ax12 STRs. Of Ax12 STR loci, 21.7% exhibited better sequencing accuracy when using reverse strand reads, while the remaining 78.3% of Ax12 STR loci exhibited the opposite.

**Supplementary Fig. S5**. Sequencing accuracy of 3-bp, 4-bp, 5-bp, and 6-bp repeat STRs. Synonymous STR types (e.g., ACG, CGA, GAC repeats) were grouped together and represented by the “representative” repeat unit (e.g., ACG repeat). STRs with at least 30 observations were included in this plot. The colorbar next to each figure represents the sequencing accuracy.

**Supplementary Fig. S6**. (A) Prediction of sequencing accuracy of Ax10 STRs using left or right flanking sequences as inputs. (B) Standard deviation of sequencing accuracy of Ax10 STRs that share the identical flanking sequences, compared against randomly sampled Ax10 STRs, demonstrating that flanking sequences indeed influence the sequencing accuracy of A-repeat STRs.

**Supplementary Fig. S7**. (A) Sequencing accuracy of A-repeat STRs that possesses specific pairs of nucleotides in specific distances within their flanking sequences. The influence of nucleotide pair on sequencing accuracy is proportionate to its proximity to A-repeat STR. (B) Sequencing accuracy of A-repeat STRs based on the number of purine counts in their flanking sequences of 4 nucleotides (2 nucleotides in each direction). (C) Sequencing accuracy of A-repeat STRs measured from the HG002 R9.4.1 dataset. A-repeat STRs that were flanked by the motifs identified in the CHM13 dataset (see the x-axis of Fig. 4B) are shown.

**Supplementary Fig. S8**. (A) Sequencing accuracy (percentage of errorless reads) of A-repeat STRs with low-complex (LC) flanking sequences that are flanked by motifs shown in Fig. 4B. Each column represents a motif that flanks the A-repeat STR, and each row represents the sequencing accuracy of A-repeat STRs with different numbers of repeats. (B) Average sequencing accuracy of Ax10–Ax13 STRs with LC flanking sequences (converted to percentile), where each dot represents A-repeat STRs with different flanking sequence motifs. (C) Relationship between purine counts of A-repeat STRs with LC flanking sequences and sequencing accuracy.

**Supplementary Fig. S9**. UMAP visualization of A-repeat STRs, created by using flanking sequences as features. Each dot represents an A-repeat locus, colored by sequencing accuracy (upper left), better basecalling model (i.e., model that generated better results for the given locus) (upper right), and the most adjacent flanking sequence (lower left).

**Supplementary Fig. S10**. (A) Base quality scores of STRs. The 2 vertical lines represent the start and end of the repeat sequences, while the horizontal lines represent the average base quality of the CHM13 dataset, highlighting the base quality “burst” observed within the STR regions of some STR types. (B) Average base quality score of reads that are presumed to harbor indel errors in STR regions.

**Supplementary Fig. S11**. (A) The average base quality of reads in various STR types observed in the HG002 R9.4.1 dataset, comparing correctly sequenced reads (i.e., reads with no error within STR region) against incorrectly sequenced reads. The horizontal line represents the average base quality of the HG002 R9.4.1 dataset. (B) The base quality “burst” observed in the HG002 R9.4.1 dataset. (C) Distribution of base quality compared between correctly sequenced bases and substitution errors.

**Supplementary Fig. S12**. (A) Sequencing accuracy of Nx12 and NNx8 STRs by various repeat units, compared between the HG002 R9.4.1 dataset and the HG002 R10.4.1 dataset. (B) Sequencing accuracy of Ax12 STRs observed in the HG002 R10.4.1 dataset, comparing the HAC basecalling model with the SUP basecalling model. Each cross in the scatterplot (left) represents an Ax12 STR locus. Around 92.99% of Ax12 STRs are better resolved using the SUP basecaller model, whereas 7.01% of Ax12 STRs are better resolved using the HAC basecaller model (right).

**Supplementary Fig. S13**. (HG002 R10.4.1 dataset) Sequencing accuracy of A-repeat STRs that harbor certain motifs in their flanking sequences, ordered by the “best” and “worst” motifs found in the CHM13 dataset (top) and the HG002 R10.4.1 dataset (bottom).

**Supplementary Fig. S14**. (A) The average base quality of reads in various STR types observed in the HG002 R10.4.1 dataset, comparing correctly sequenced reads (i.e., reads with no error within the STR region) with incorrectly sequenced reads. The horizontal line represents the average base quality of the HG002 R10.4.1 dataset. (B) The base quality “burst” observed in the HG002 R10.4.1 dataset. (C) Distribution of base quality compared between correctly sequenced bases and substitution errors.

**Supplementary Fig. S15**. (A) NanoMnT, NanoSTR, and NanoRepeat genotyping results of three hundred 2-bp repeat STR loci. (B) STR allele size histograms of 2 example Ax15 loci, one with a highly prominent peak and the other with a less prominent peak. The dashed line represents the genotyped STR allele for each locus.

**Supplementary Table S1**. Metadata of all datasets used in this study.

**Supplementary Table S2**. STR loci used for tool benchmarking.

**Supplementary Table S3**. STR loci used MSI detection (SG-NEx).

**Supplementary Table S4**. STR loci used MSI detection (CRC WGS, Pickles et al.).

## Abbreviations

bp: base pair; CMS: consensus molecular subtype; CNN: convolutional neural network; CRC: colorectal cancer; dMMR: mismatch repair deficiency; HAC: high accuracy; MSI: microsatellite instability; MSS: microsatellite stability; NGS: next-generation sequencing; ONT: Oxford Nanopore Technology; SG-NEx: The Singaporean Nanopore Expression Dataset; STR: short tandem repeat; SUP: super accuracy; WGS: whole-genome sequencing.
