# Supplementary material for: NanoMnT: an STR analysis tool for Oxford Nanopore sequencing data driven by a comprehensive analysis of error profile in STR regions
Source: Gigascience. 2025 Mar 17;14:giaf013. doi: 10.1093/gigascience/giaf013 (PMC11912559; doi:10.1093/gigascience/giaf013)
Supplement: giaf013_Supplemental_Files [file giaf013_supplemental_files.zip › Supplementary Figures.pdf]

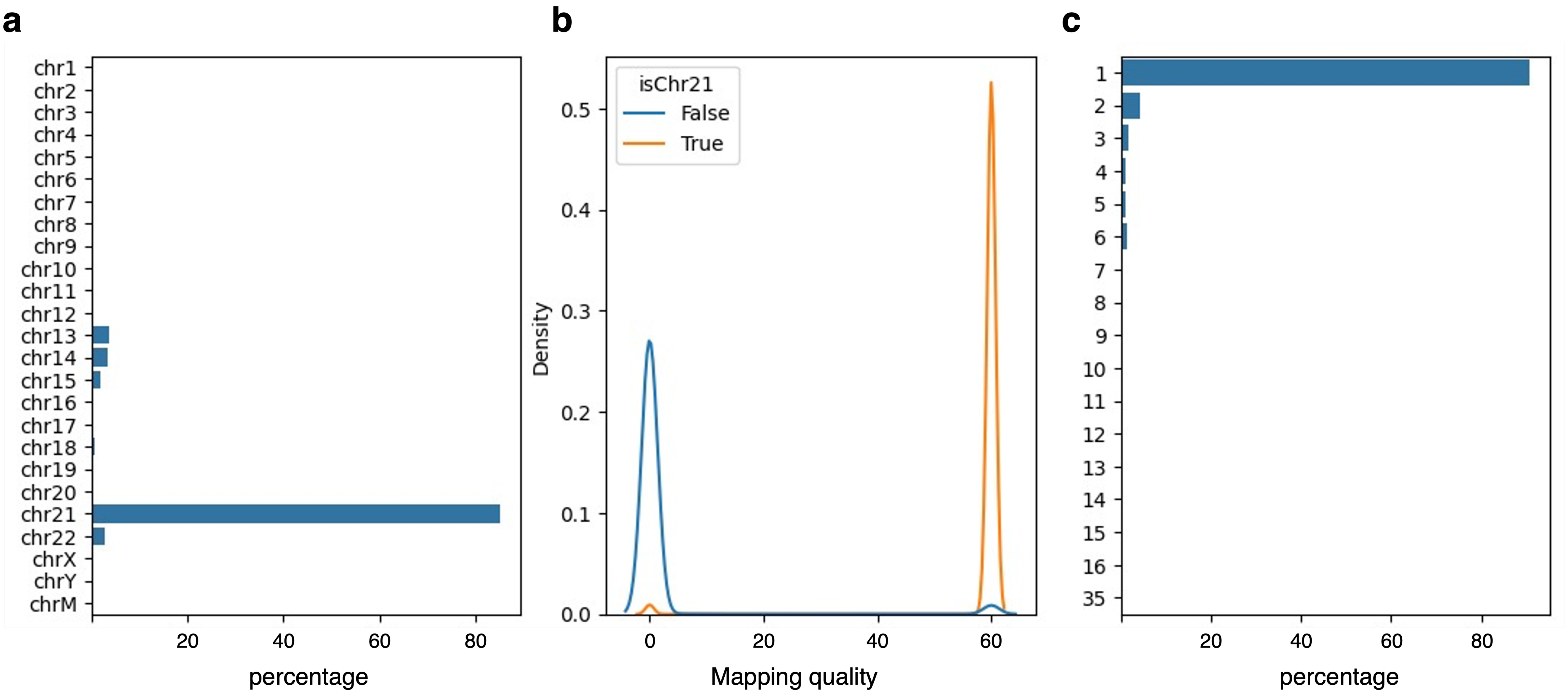

Supplementary Figure 1.
(a) Percentage of chromosomes which reads (of the CHM13 dataset, whose primary alignments aligned to chromosome 21) realigned to. **(b)** Mapping quality distribution of two groups of the realigned reads: reads that mapped back to chromosome 21 and reads that mapped elsewhere. **(c)** Distribution of the number of alignments per read. E.g., 1 indicates that a read aligned to a single region, and 2 indicates that a read aligned to two different regions.

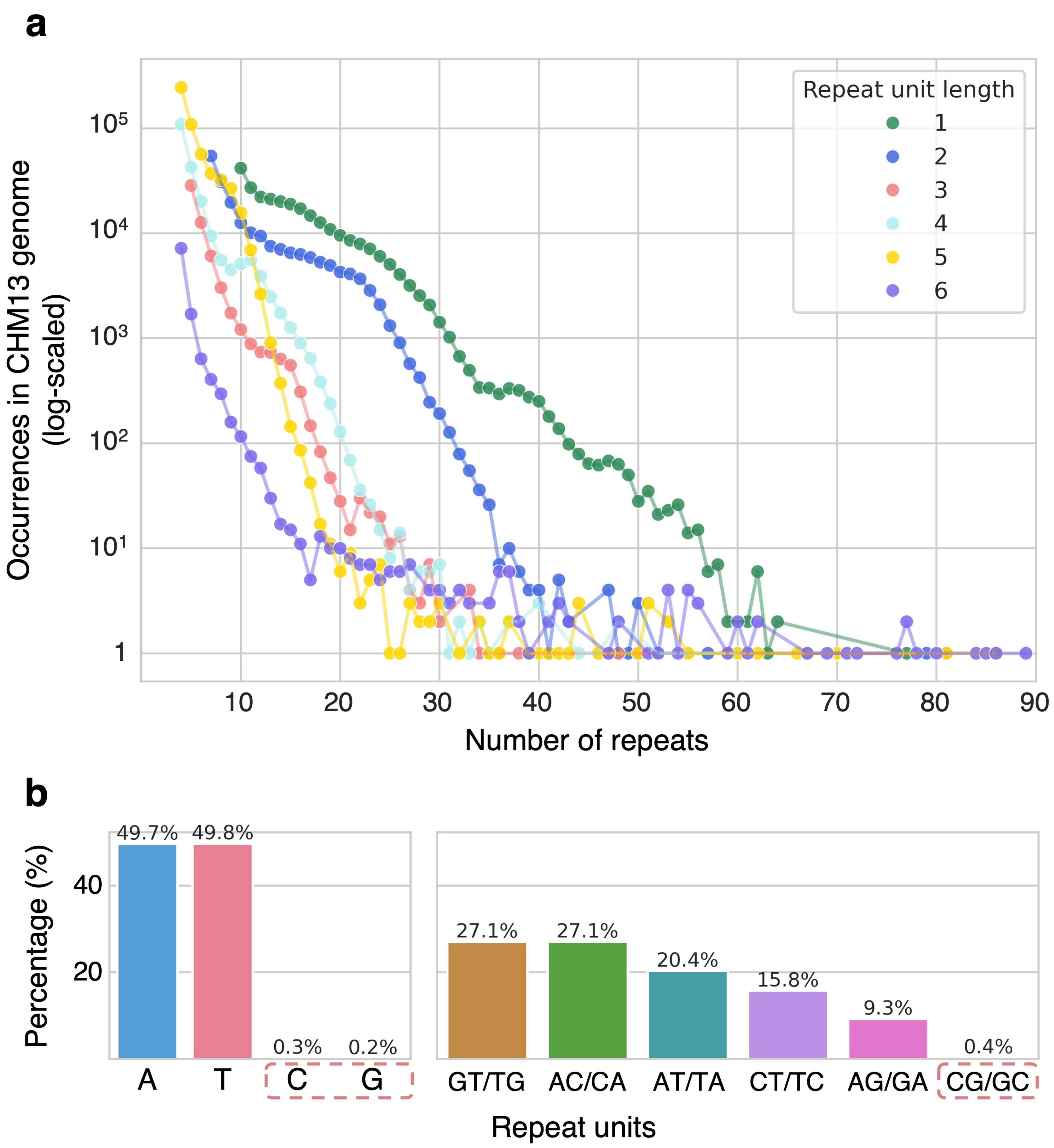

Supplementary Figure 2.
(a) Number of STRs analyzed in this study. **(b)** Percentage of 1bp-/2bp-repeat STRs by repeat units.

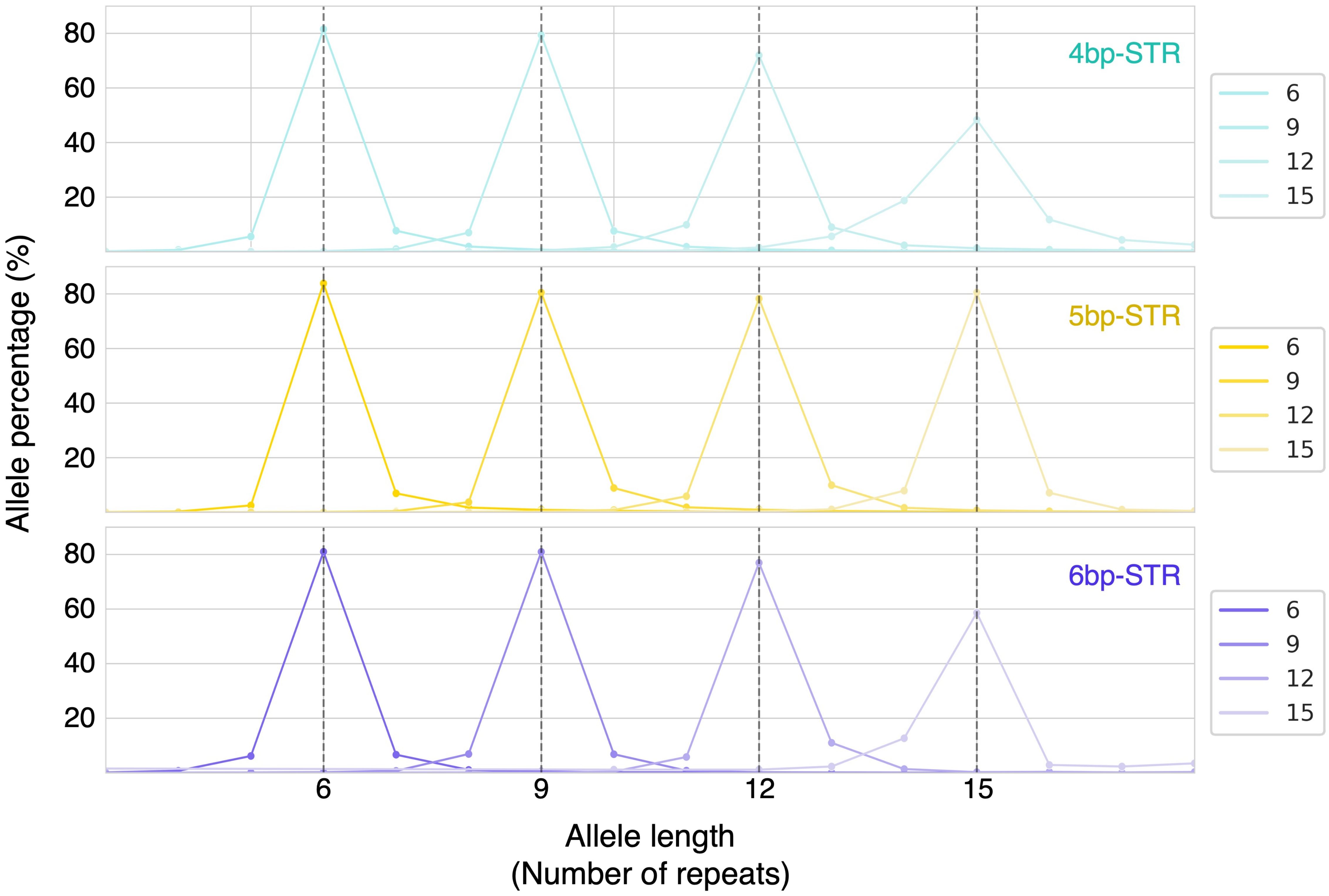

Supplementary Figure 3.
STR allele size histograms of various lengths of 4bp-, 5bp- and 6bp-repeat STR.

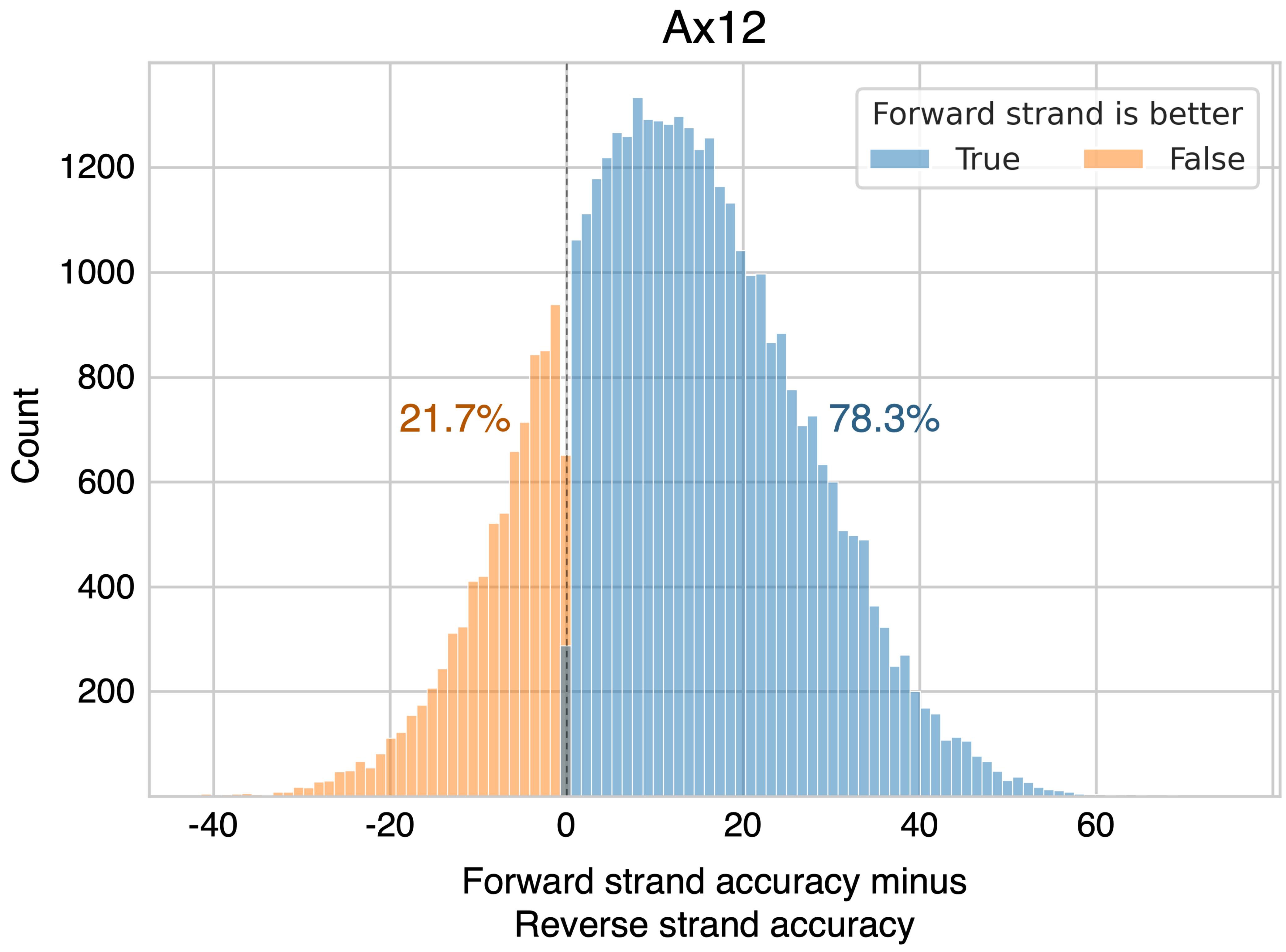

Supplementary Figure 4.
Distribution of forward strand sequencing accuracy (i.e., sequencing accuracy calculating using only forward strand reads) minus reverse strand sequencing accuracy in Ax12 STRs. 21.7% of Ax12 STR loci exhibited better sequencing accuracy when using reverse strand reads, while the remaining 78.3% of Ax12 STR loci exhibited the opposite.

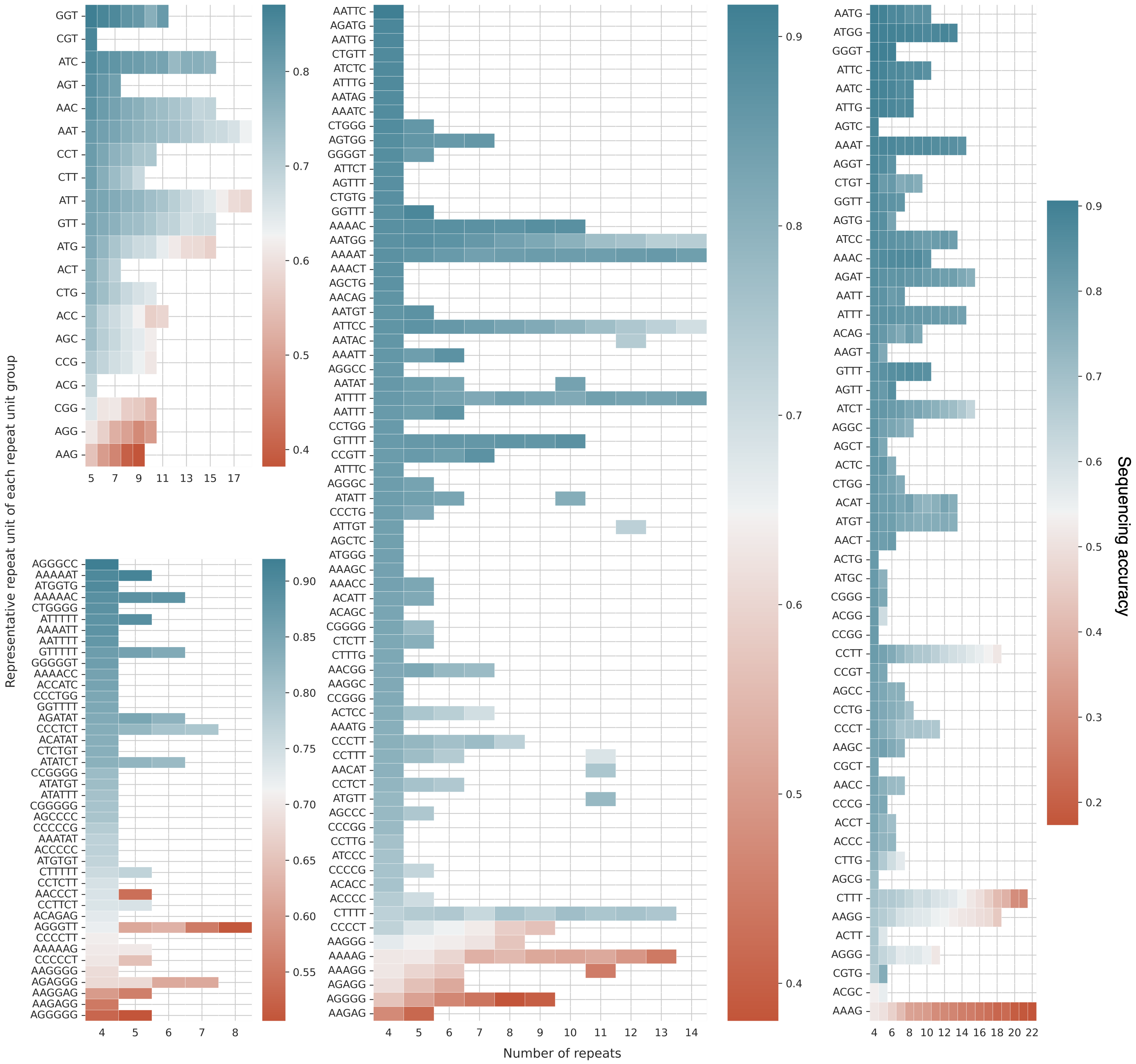

Supplementary Figure 5.
Sequencing accuracy of 3bp-, 4bp-, 5bp- and 6bp-repeat STRs. Synonymous STR types (e.g., ACG-, CGA-, GAC-repeats) were grouped together and represented by the 'representative' repeat unit (e.g., ACG-repeat). STRs with at least 30 observations were included in this plot. The colorbar next to each figure represents the sequencing accuracy.

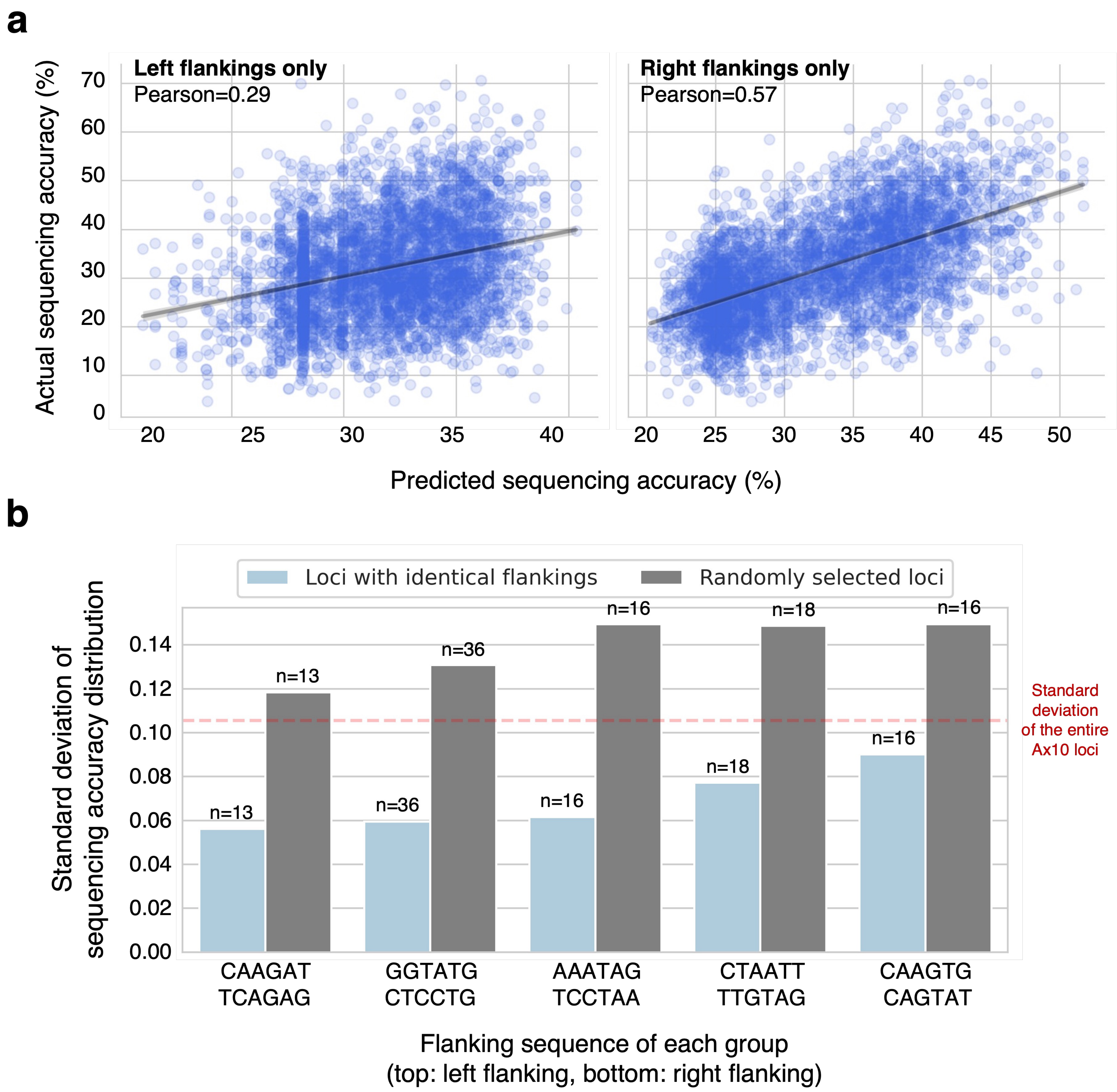

Supplementary Figure 6.

(a) Prediction of sequencing accuracy of Ax10 STRs using left or right flanking sequences as inputs. **(b)** Standard deviation of sequencing accuracy of Ax10 STRs that share the identical flanking sequences, compared against randomly sampled Ax10 STRs, demonstrating that flanking sequences indeed influence the sequencing accuracy of A-repeat STRs.

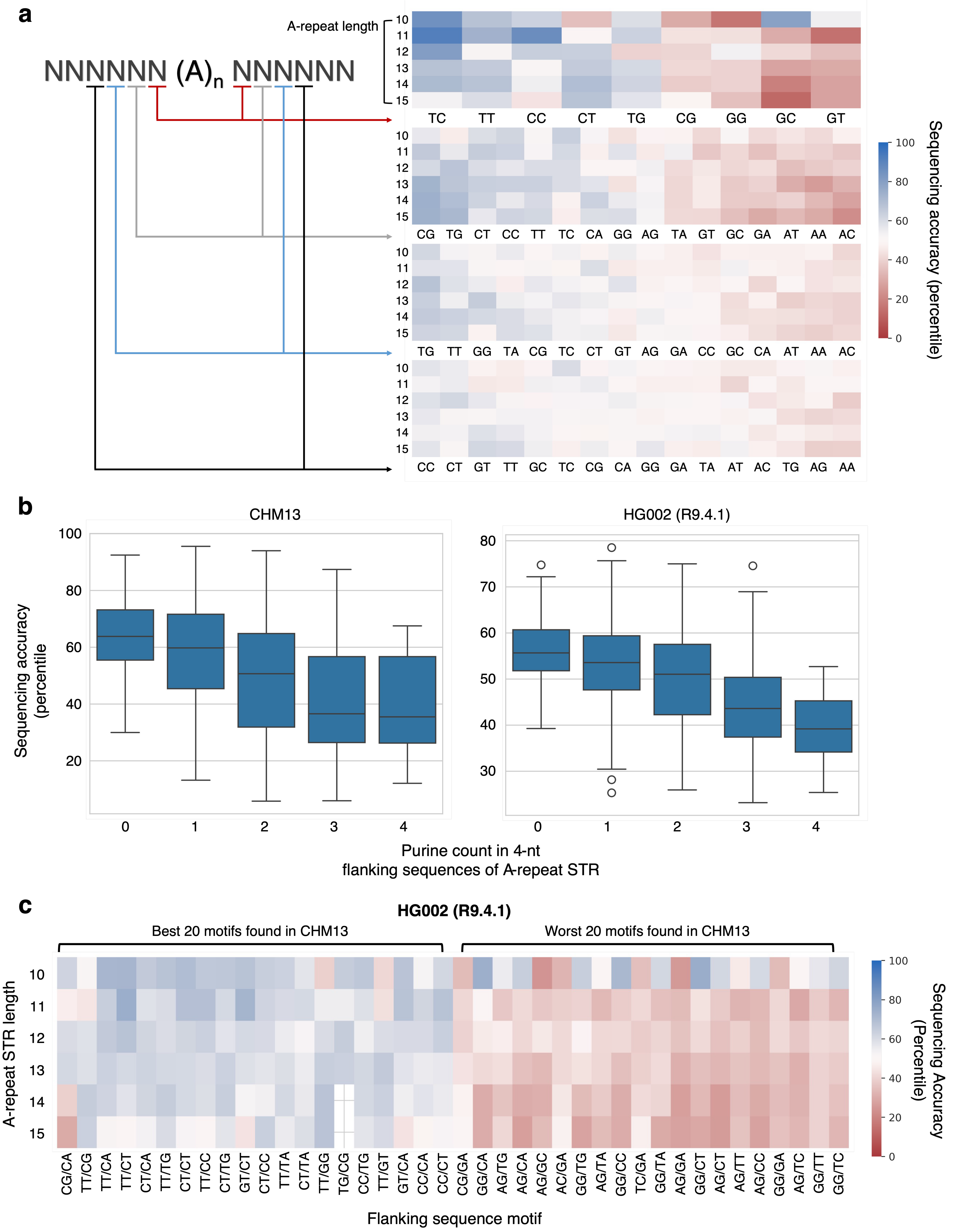
a
A-repeat length
NNNNNN (A)n NNNNNN
TC TT CC CT TG CG GG GC GT
CG TG CT CC TT TC CA GG AG TA GT GC GA AT AA AC
TG TT GG TA CG TC CT GT AG GA CC GC CA AT AA AC
CC CT GT TT GC TC CG CA GG GA TA AT AC TG AG AA
Sequencing accuracy (percentile)
b
CHM13
HG002 (R9.4.1)
Sequencing accuracy (percentile
Purine count in 4-nt flanking sequences of A-repeat STR
c
HG002 (R9.4.1)
Best 20 motifs found in CHM13
Worst 20 motifs found in CHM13
A-repeat STR length
CG/CA TT/CG TT/CA TT/CT CT/CA TT/TG CT/CT TT/CC CT/TG GT/CT CT/CC TT/TA CT/TA TT/GG TG/CG CC/TG TT/GT GT/CA CC/CA CC/CT CG/GA GG/CA AG/TG AG/CA AG/GC AC/GA GG/TG AG/TA GG/CC TC/GA GG/TA AG/GA GG/CT AG/CT AG/TT AG/CC GG/GA AG/TC GG/TT GG/TC
Flanking sequence motif
Sequencing Accuracy (Percentile)

Supplementary Figure 7.
(a) Sequencing accuracy of A-repeat STRs that possesses specific pairs of nucleotides in specific distances within their flanking sequences. The influence of nucleotide pair on sequencing accuracy is proportionate to its proximity to A-repeat STR. **(b)** Sequencing accuracy of A-repeat STRs based on the number of purine counts in their flanking sequences of 4 nucleotides (2 nucleotide in each direction) **(c)** Sequencing accuracy of A-repeat STRs measured from the HG002 R9.4.1 dataset. A-repeat STRs that were flanked by the motifs identified in the CHM13 dataset (see the x-axis of Figure 4b) were shown.

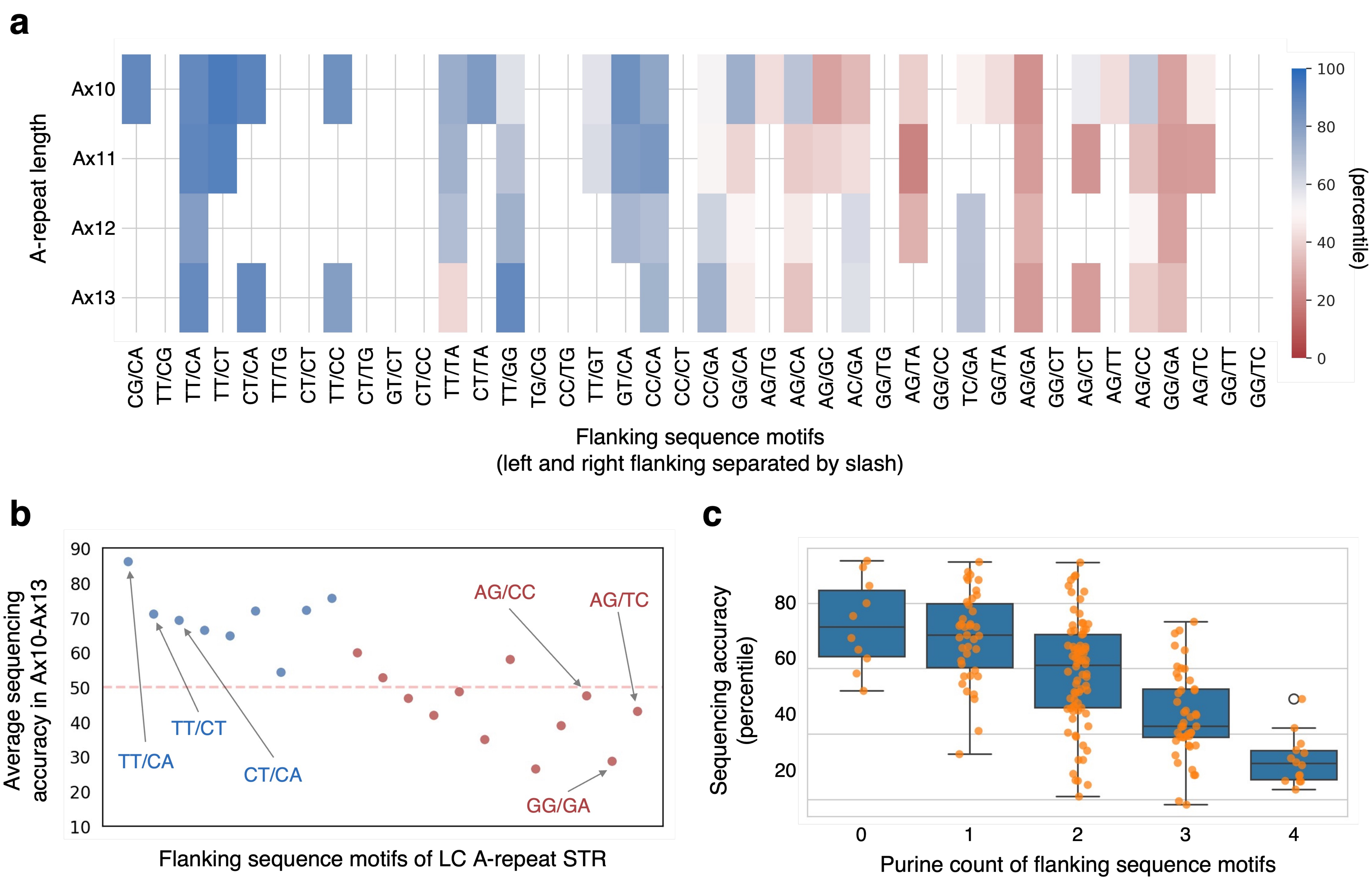

Supplementary Figure 8.

(a) Sequencing accuracy (percentage of errorless reads) of A-repeat STRs with low-complex (LC) flanking sequences, that are flanked by motifs shown in Figure 4b. Each column represents a motif that flank the A-repeat STR, and each row represents the sequencing accuracy of A-repeat STRs with different numbers of repeats. **(b)** Average sequencing accuracy of Ax10-Ax13 STRs with LC flanking sequences (converted to percentile), where each dot represents A-repeat STRs with different flanking sequence motifs. **(c)** Relationship between purine counts of A-repeat STRs with LC flanking sequences and sequencing accuracy.

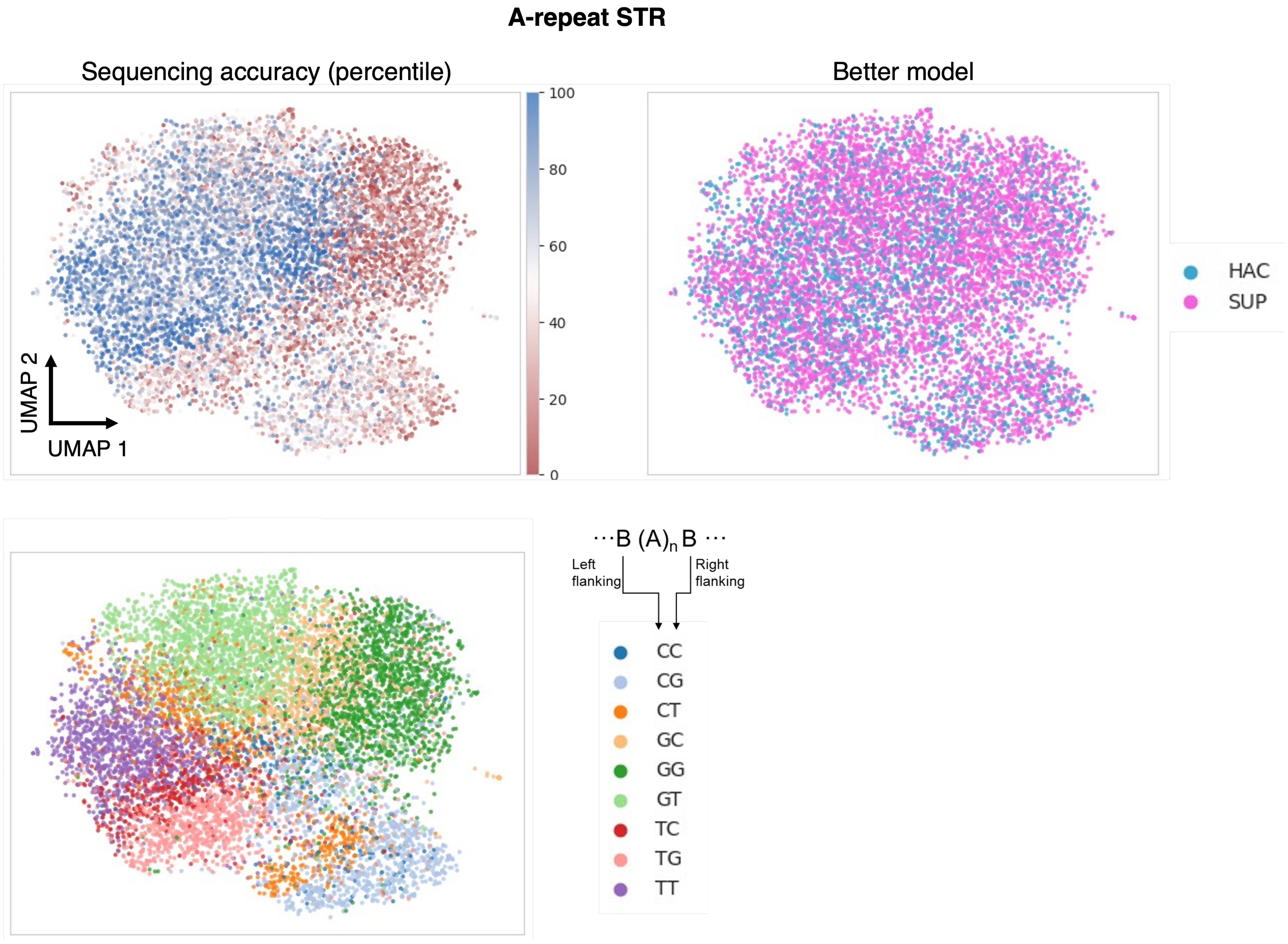

Supplementary Figure 9.
UMAP visualization of A-repeat STRs, created by using flanking sequences as features. Each dot represents a A-repeat locus, colored by sequencing accuracy (upper left), better basecalling model (i.e., model that generated better results for the given locus) (upper right) and the most adjacent flanking sequence (lower left).

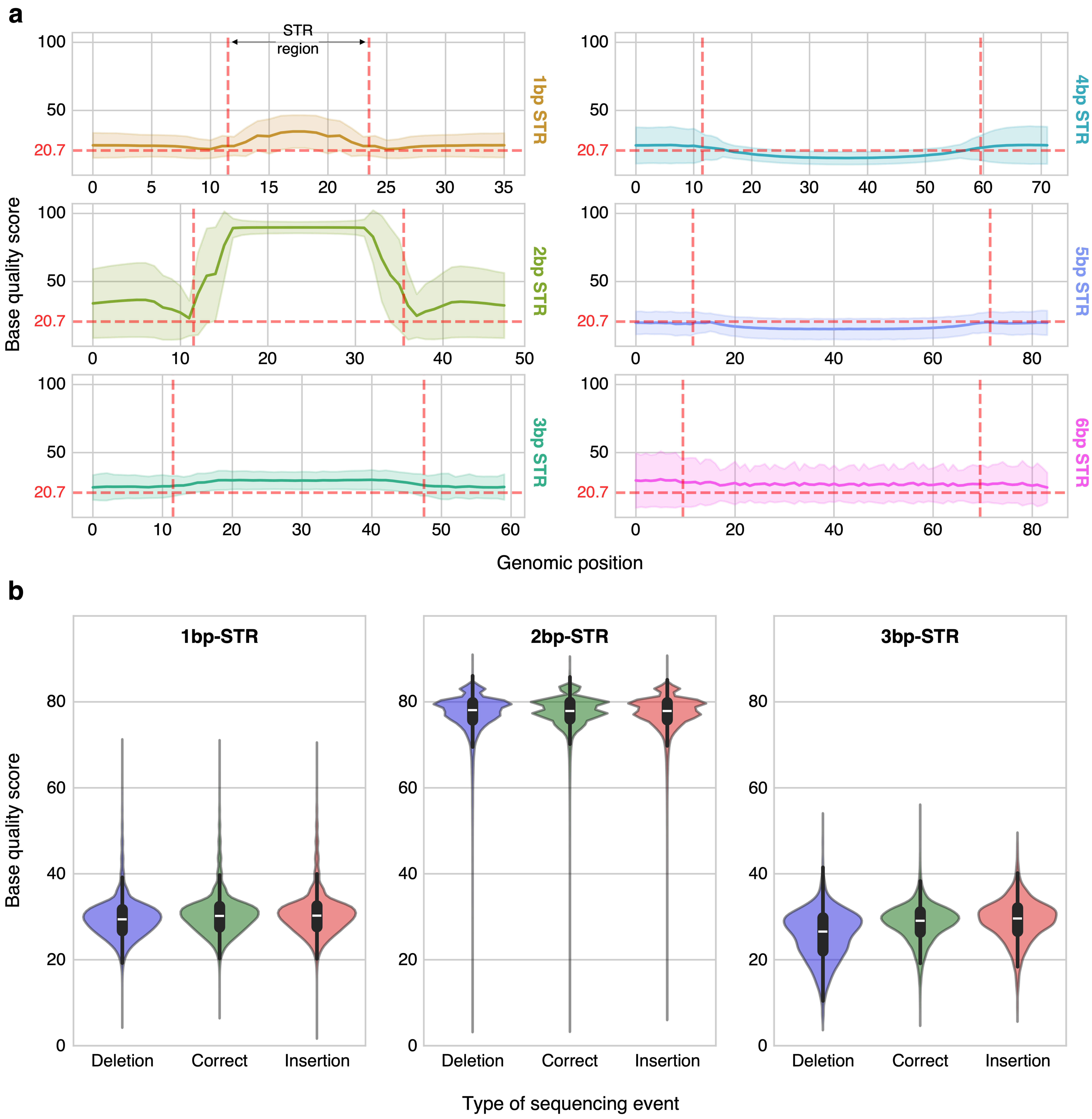

Supplementary Figure 10.
(a) Base quality scores of STRs. The two vertical lines represent the start and end of the repeat sequences, while the horizontal lines represent the average base quality of the CHM13 dataset, highlighting the base quality 'burst' observed within the STR regions of some STR types. **(b)** Average base quality score of reads that are presumed to harbor indel errors in STR regions.

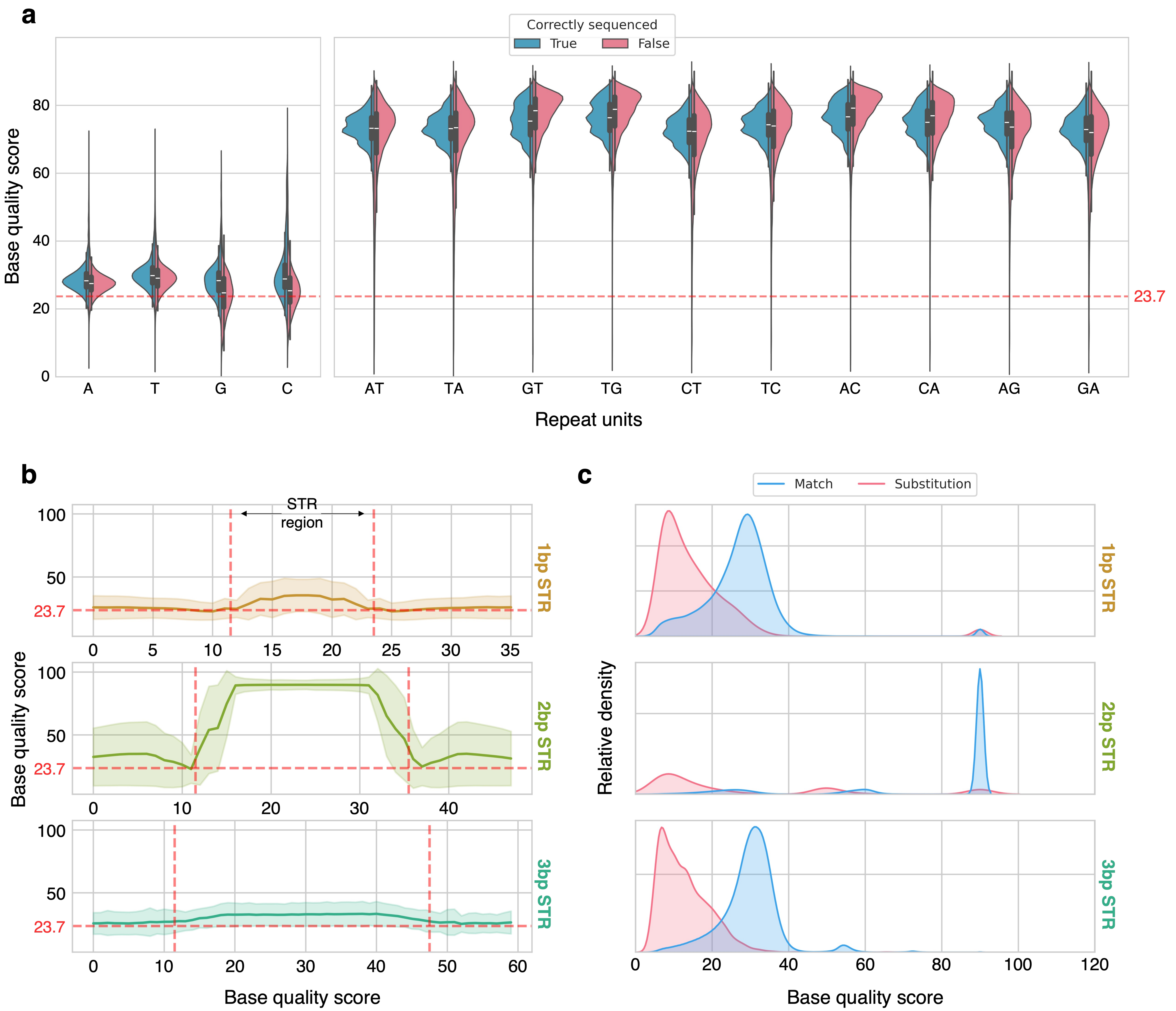

Supplementary Figure 11.
(a) The average base quality of reads in various STR types observed in the HG002 R9.4.1 dataset, comparing correctly sequenced reads (i.e., reads with no error within STR region) against incorrectly sequenced reads. The horizontal line represents the average base quality of the HG002 R9.4.1 dataset. **(b)** The base quality 'burst' observed in the HG002 R9.4.1 dataset. **(c)** Distribution of base quality compared between correctly sequenced bases and substitution errors.

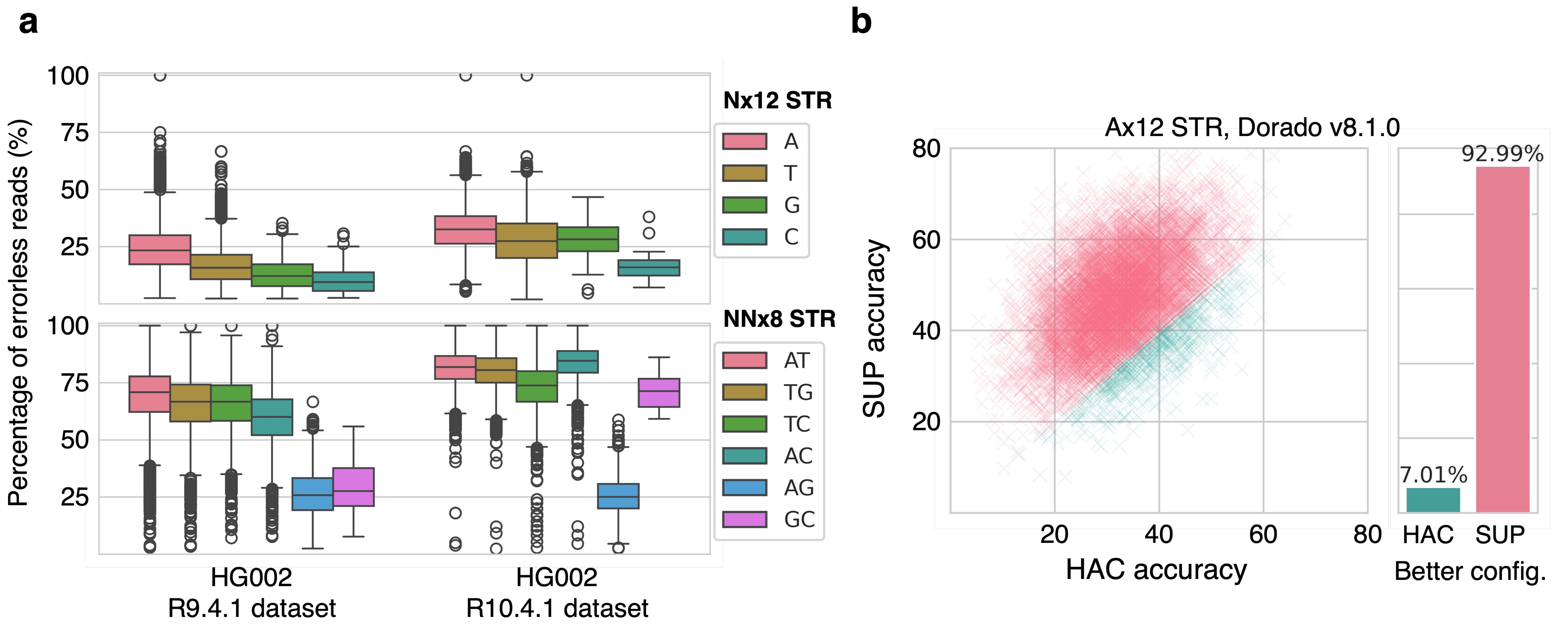

Supplementary Figure 12.
(a) Sequencing accuracy of Nx12 and NNx8 STRs by various repeat units, compared between HG002 R9.4.1 dataset and HG002 R10.4.1 dataset. **(b)** Sequencing accuracy of Ax12 STRs observed in HG002 R10.4.1 dataset, comparing HAC basecalling model against SUP basecalling model. Each cross in the scatterplot (left) represents a Ax12 STR locus. Around 92.99% of Ax12 STRs are better resolved using SUP basecaller model, whereas 7.01% of Ax12 STRs are better resolved using HAC basecaller model (right).

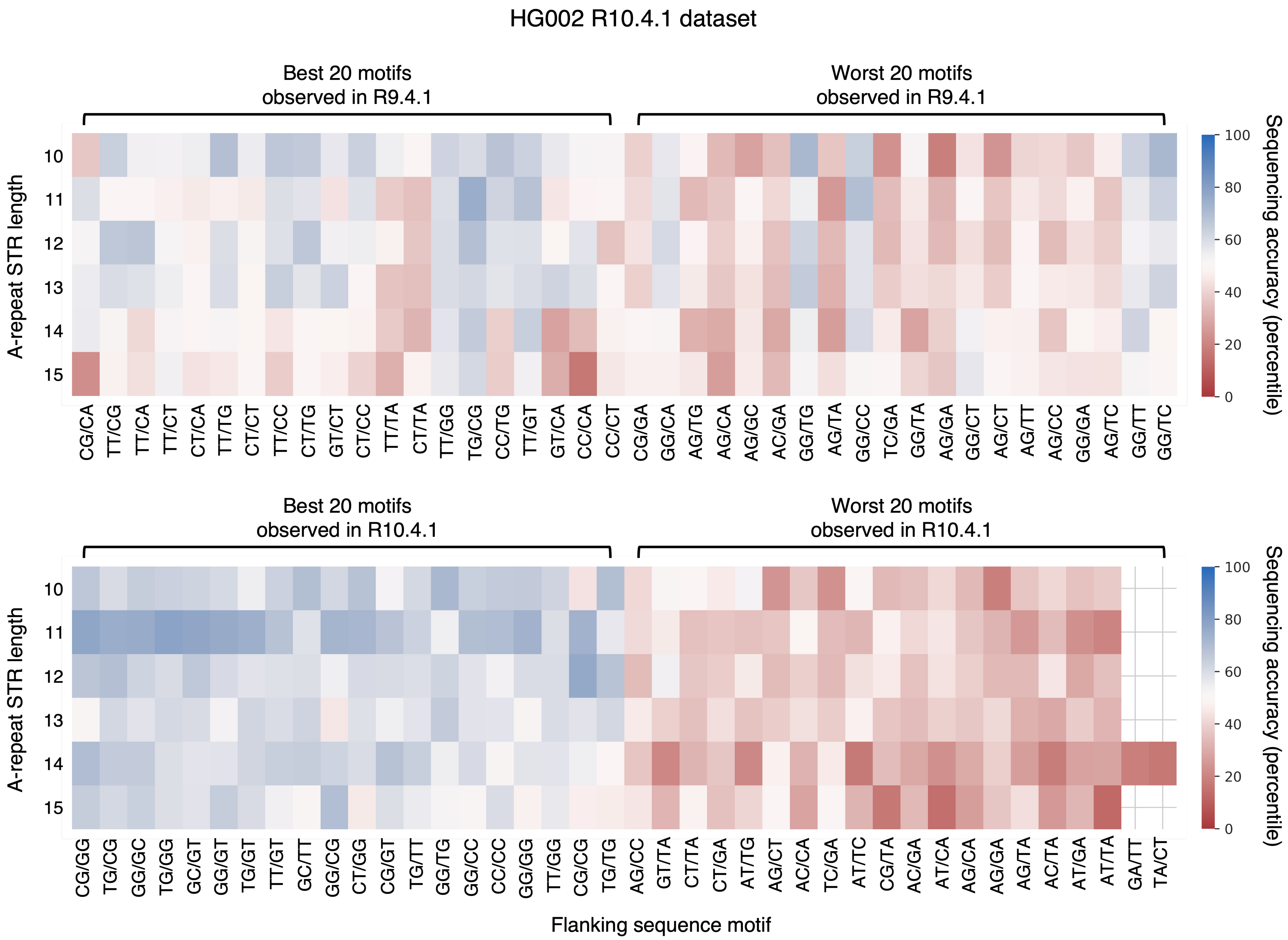

Supplementary Figure 13.

(HG002 R10.4.1 dataset) Sequencing accuracy of A-repeat STRs that harbor certain motifs in their flanking sequences, ordered by the 'best' and 'worst' motifs found in the CHM13 dataset (top) and the HG002 R10.4.1 dataset (bottom).

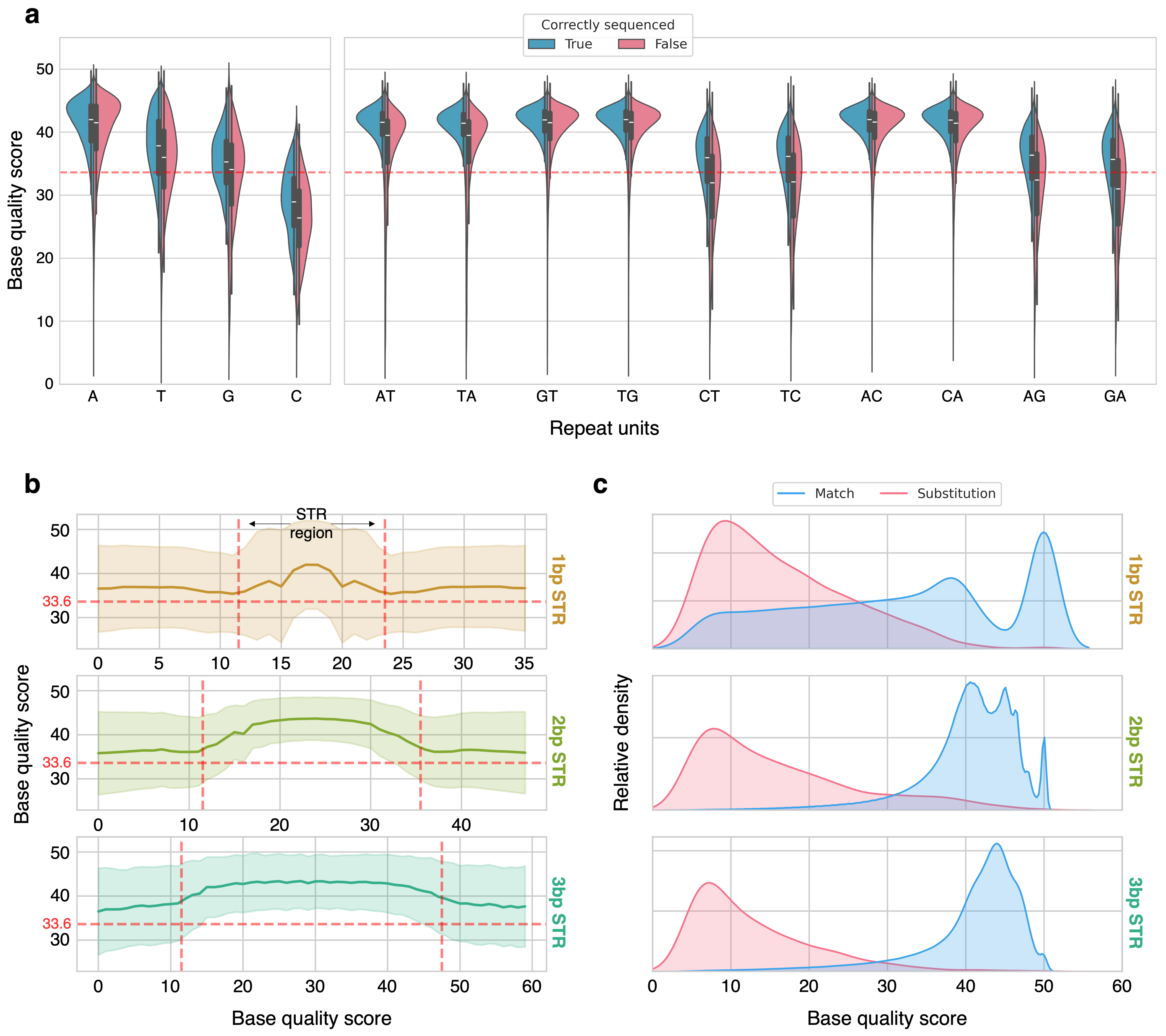

Supplementary Figure 14.
(a) The average base quality of reads in various STR types observed in the HG002 R10.4.1 dataset, comparing correctly sequenced reads (i.e., reads with no error within STR region) against incorrectly sequenced reads. The horizontal line represents the average base quality of the HG002 R10.4.1 dataset. **(b)** The base quality 'burst' observed in the HG002 R10.4.1 dataset. **(c)** Distribution of base quality compared between correctly sequenced bases and substitution errors.

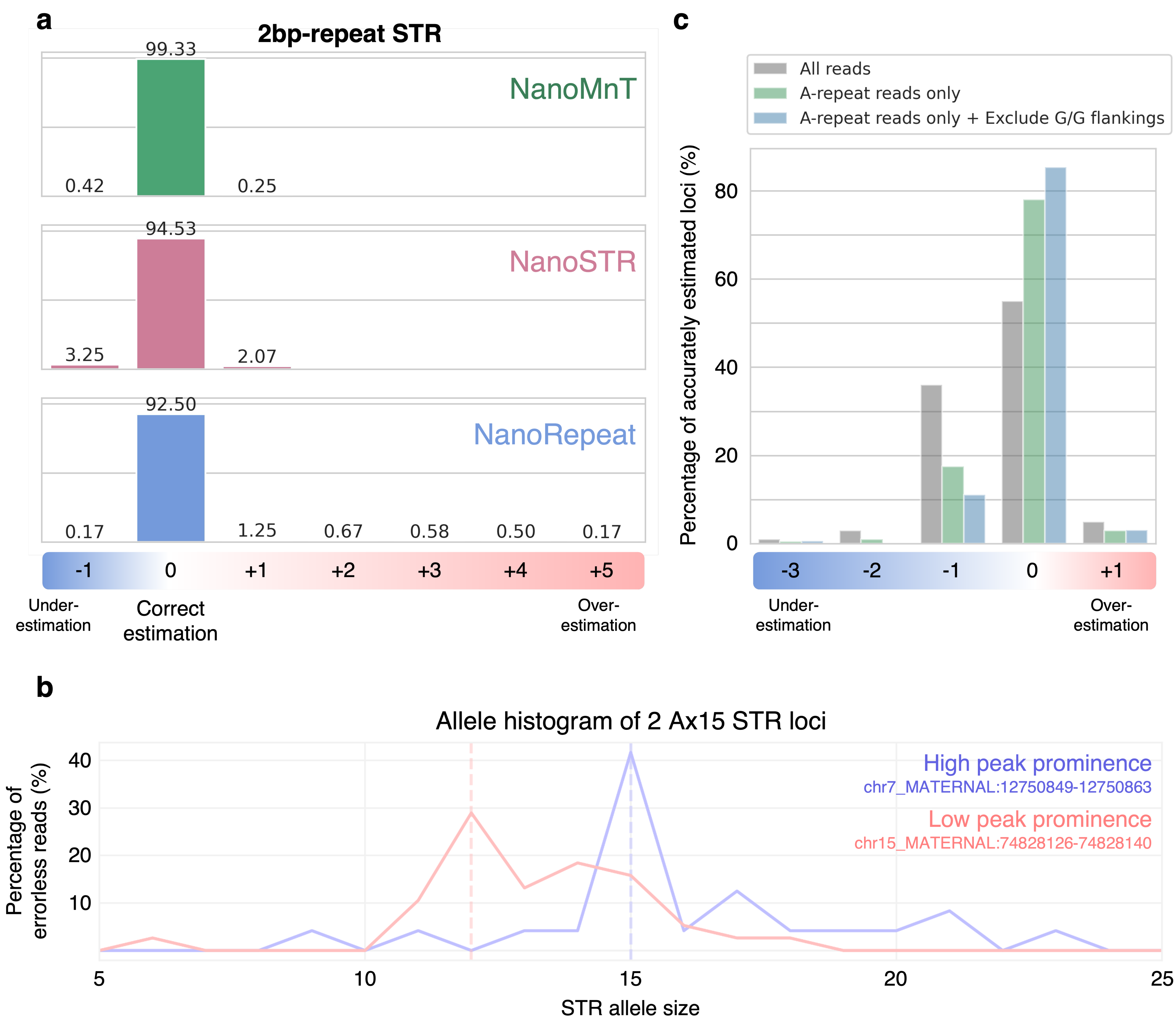

Supplementary Figure 15.
(a) NanoMnT, NanoSTR and NanoRepeat genotyping results of 300 2bp-repeat STR loci. **(b)** STR allele size histograms of 2 example Ax15 loci, one with a highly prominent peak, and the other with a less prominent peak. The dashed line represents the genotyped STR allele for each locus.
