# Supplementary material for: NanoMnT: an STR analysis tool for Oxford Nanopore sequencing data driven by a comprehensive analysis of error profile in STR regions
Source: Gigascience. 2025 Mar 17;14:giaf013. doi: 10.1093/gigascience/giaf013 (PMC11912559; doi:10.1093/gigascience/giaf013)
Supplement: giaf013_GIGA-D-24-00346_Original_Submission [file giaf013_giga-d-24-00346_original_submission.pdf]

GigaScience

NanoMnT: A STR analysis tool for Oxford Nanopore sequencing data driven by comprehensive analysis of error profile in STR regions.

--Manuscript Draft--

**Manuscript Number:**	GIGA-D-24-00346
**Full Title:**	NanoMnT: A STR analysis tool for Oxford Nanopore sequencing data driven by comprehensive analysis of error profile in STR regions.
**Article Type:**	Research
**Funding Information:**	National Research Foundation of Korea (RS-2024-00335026) — Dr Jihwan Park
**Abstract:**	Nanopore sequencing is a third-generation sequencing technology that offers cost-effective long-read sequencing. Although Nanopore sequencing offers exciting opportunities to research various areas of biology, its elevated sequencing error rate in low-complexity regions hampers its applications in short tandem repeat (STR) related research. To address this issue, we comprehensively analyzed publicly available Nanopore sequencing datasets. We show that the sequencing error rate is not only STR length-dependent but also dependent on repeat unit and flanking sequence of STR regions. In particular, some flanking sequences were associated with good sequencing accuracy of STR, implying that certain STR loci are more viable for Nanopore sequencing compared to other loci. Moreover, while the base quality scores of substitution errors within STR regions were markedly lower than correctly sequenced bases, such discerning patterns could not be observed in indel errors. Furthermore, we show that choosing the most up to date basecaller version as well as using the SUP configuration confers significant improvements in STR sequencing accuracy. Finally, we present NanoMnT, a lightweight Python-based tool that corrects STR sequencing errors in ONT data and estimates STR allele sizes. Using NanoMnT, we present the utility of our findings by identifying MSI/MSS status in cancer sequencing data. NanoMnT is available in https://github.com/18parkky/NanoMnT.
**Corresponding Author:**	Jihwan Park<br>Gwangju Institute of Science and Technology<br>Gwangju, KOREA, REPUBLIC OF
**Corresponding Author Secondary Information:**	
**Corresponding Author's Institution:**	Gwangju Institute of Science and Technology
**Corresponding Author's Secondary Institution:**	
**First Author:**	Gyumin Park
**First Author Secondary Information:**	
**Order of Authors:**	Gyumin Park
	Hyunsu An
	Han Luo, PhD
	Jihwan Park, PhD
**Order of Authors Secondary Information:**	
**Additional Information:**	
**Question**	**Response**
Are you submitting this manuscript to a special series or article collection?	No
**Experimental design and statistics**	Yes

Powered by Editorial Manager® and ProduXion Manager® from Aries Systems Corporation

Full details of the experimental design and statistical methods used should be given in the Methods section, as detailed in our Minimum Standards Reporting Checklist. Information essential to interpreting the data presented should be made available in the figure legends.<br><br>Have you included all the information requested in your manuscript?	
**Resources**<br><br>A description of all resources used, including antibodies, cell lines, animals and software tools, with enough information to allow them to be uniquely identified, should be included in the Methods section. Authors are strongly encouraged to cite Research Resource Identifiers (RRIDs) for antibodies, model organisms and tools, where possible.<br><br>Have you included the information requested as detailed in our Minimum Standards Reporting Checklist?	Yes
**Availability of data and materials**<br><br>All datasets and code on which the conclusions of the paper rely must be either included in your submission or deposited in publicly available repositories (where available and ethically appropriate), referencing such data using a unique identifier in the references and in the “Availability of Data and Materials” section of your manuscript.<br><br>Have you have met the above requirement as detailed in our Minimum Standards Reporting Checklist?	Yes

Powered by Editorial Manager® and ProduXion Manager® from Aries Systems Corporation

Click here to access/download;Manuscript;NanoMnT_FinalManuscript.docx

NanoMnT: A STR analysis tool for Oxford Nanopore sequencing data driven by comprehensive analysis of error profile in STR regions.

Gyumin Park[1], Hyunsu An[1], Han Luo[2]*, Jihwan Park[1]*

[1] School of Life Sciences, Gwangju Institute of Science and Technology (GIST), Republic of Korea.

[2] Department of Thyroid and Parathyroid Surgery, Laboratory of thyroid and parathyroid disease, Frontiers Science Center for Disease-related Molecular Network, West China Hospital, Sichuan University, Chengdu, Sichuan, China

* Correspondence:

Han Luo

Department of Thyroid and Parathyroid Surgery, Laboratory of Thyroid and Parathyroid Disease, Frontiers Science Center for Disease-related Molecular Network, West China Hospital, Sichuan University, Chengdu, Sichuan, China.

No. 37 Guoxue Alley, Chengdu, Sichuan, China, 610041

Phone: +86-18980605139

Fax: +86-28-85422467

Email: luohan-hx@scu.edu.cn

Jihwan Park

School of Life Sciences, Gwangju Institute of Science and Technology

123 Cheomdangwagi-ro, Buk-gu, Gwangju, Republic of Korea, 61005

Phone: +82-627152522

Fax: +82-627152484

Email: jihwan.park@gist.ac.kr

Keywords: Oxford Nanopore; Long-read sequencing; Short tandem repeats; Microsatellite; Error profile; Bioinformatics.

Abstract

Nanopore sequencing is a third-generation sequencing technology that offers cost-effective long-read sequencing. Although Nanopore sequencing offers exciting opportunities to research various areas of biology, its elevated sequencing error rate in low-complexity regions hampers its applications in short tandem repeat (STR) related research. To address this issue, we comprehensively analyzed publicly available Nanopore sequencing datasets. We show that the sequencing error rate is not only STR length-dependent but also dependent on repeat unit and flanking sequence of STR regions. In particular, some flanking sequences were associated with good sequencing accuracy of STR, implying that certain STR loci are more viable for Nanopore sequencing compared to other loci. Moreover, while the base quality scores of substitution errors within STR regions were markedly lower than correctly sequenced bases, such discerning patterns could not be observed in indel errors. Furthermore, we show that choosing the most up to date basecaller version as well as using the SUP configuration confers significant improvements in STR sequencing accuracy. Finally, we present NanoMnT, a lightweight Python-based tool that corrects STR sequencing errors in ONT data and estimates STR allele sizes. Using NanoMnT, we present the utility of our findings by identifying MSI/MSS status in cancer sequencing data. NanoMnT is available in https://github.com/18parkky/NanoMnT.

Introduction

Short tandem repeats (STRs), also known as microsatellites, are DNA composed of tandemly repeated units which can range from 1 base pair (bp) to 6 bp (Gymrek, 2017) and are routinely employed as ‘genetic fingerprints’ in a variety of fields such as forensics (Alonso et al., 2018), population genetics (Bruford & Wayne, 1993). Furthermore, much previous research has showed that STR are involved in gene regulation by modulating DNA methylation and transcription (Fotsing et al., 2019). Owing to their elevated mutation rate, STRs are also known to play important roles in the pathogenesis of numerous diseases (Brinkmann et al., 1998). For example, the repeat expansions of certain STRs are responsible for the development of various genetic and neurological diseases including Fragile X syndrome, spinal and bulbar muscular atrophy (SBMA), Huntington disease (Malik et al., 2021). In addition, the genomic instability of STRs in mismatch repair-deficient (dMMR) cancers manifests as a molecular phenotype known as microsatellite instability (MSI), which is generally associated with a favorable response to cancer immunotherapy (Chang et al., 2018; Li et al., 2020). In any case, the allele size of STRs, i.e., the length of the repeat units, is crucial for understanding their roles in these biological contexts. Therefore, precise quantification of STR allele size is essential for accurate assessment and interpretation of STRs and their functions in gene regulation, disease development, and cancer biology.

Although conventional PCR-based methods for analyzing STR have proven to be effective for this purpose (Suraweera et al., 2002), they are constrained by the number of STR regions that can be simultaneously analyzed. The advancement of next-generation sequencing (NGS) has significantly expanded the number of STR regions that can be analyzed, to an extent that whole-genome sequencing (WGS) with sufficient sequencing depth can enable the analysis of most STR regions. Among various sequencing technologies, long-read sequencing is generally advantageous for the characterization of STR regions compared to short-read sequencing, as it can sequence the flanking sequences of STR regions and generate robust alignments of STR regions. On the contrary, short-read sequencing often fails to sequence the flanking sequences, which may result in producing ambiguous alignments or misalignments. Furthermore,

the lengths of some STR regions exceed the read length of short-read sequencing, requiring longer read lengths for accurate STR allele identification (Liu et al., 2020; Wei et al., 2014). PacBio sequencing and Oxford Nanopore sequencing (ONT) are two of the most widely applied long-read sequencing technologies and have been extensively employed in a multitude of research fields (Rhoads & Au, 2015; Wang et al., 2021). Although PacBio HiFi sequencing is superior to ONT in sequencing accuracy, ONT offers unique advantages such as real-time analysis, portability, and the generation of ultra-long reads. In addition to these factors, the prices of ONT devices are more affordable compared to other sequencing devices, and provide superior cost-effectiveness (throughput per cost), making ONT an attractive choice for numerous research groups. Nonetheless, ONT's high sequencing error rate poses a significant challenge, which is particularly high in low-complexity regions, critically limiting its utility in STR-related analyses.

Despite ONT’s high error rate, several bioinformatic tools designed to analyze STR from ONT data have been developed, such as NanoSTR and NanoRepeat (Fang et al., 2023; Lang et al., 2023). NanoSTR is a Perl-based program that genotypes STR regions and has demonstrated good performance for 75 forensic STR markers. NanoRepeat, developed by Fang et al., can genotype not only STR composed of single repeat units, but also adjacent pairs of STR regions. However, both tools have several computational limitations, most notably, neither tool can provide metrics that indicate the confidence of genotyping results and has not been tested for mono- and di-nucleotide repeats. Indeed, the high error rate of ONT in low-complexity regions is known to be further exacerbated in mono- and di-nucleotide repeats (Delahaye & Nicolas, 2021) In particular, mononucleotide repeats are widely known to be the most unstable in MSI cancers, prompting most PCR-based MSI detection kits to use mononucleotide-based panel (Li et al., 2020). Furthermore, each tool possesses an inconvenient disadvantage: NanoSTR relies on Porechop, a bioinformatic tool that performs adapter trimming for ONT reads, which require the entire FASTQ file to be loaded onto the memory (RAM) and is no longer maintained as of October 2018. On the other hand, while NanoRepeat is regularly updated and has shown to be useful in in trinucleotide repeat related studies, the file sizes of outputs can get relatively big, and thus not suitable for analyzing large numbers of STR. All in all, although NanoSTR and NanoRepeat are useful tools within their scope of design, bioinformatic tools that enable STR analysis – including mono- and di-nucleotide repeats – by accounting for ONT-specific error profiles are yet to be developed.

In this article, we provide a comprehensive analysis of ONT error profile in STR regions using 3 publicly available ONT sequencing datasets (Supplementary Table 1). We centered our analysis on the T2T-CHM13 dataset (generated using R9.4.1 flowcells) due to its high sequencing depth (~120x). The near homozygosity of the CHM13 cell line removes the need to consider biallelic STR signals, enabling a straightforward analysis. In addition, we employed 2 additional datasets – sequencing dataset of HG002 generated using the R9.4.1 flowcell and the R10.4.1 flowcell, both publicly available through EPI2ME – to validate our findings and translate them into the current R10.4.1 flowcell version. These HG002 datasets are referred to as ‘HG002 R9.4.1 dataset’ and ‘HG002 R10.4.1 dataset’ throughout the article. Unless otherwise specified, all R9.4.1 data presented in this article have been basecalled using Guppy v6.5.7 high accuracy (HAC) model, while R10.4.1 data have been basecalled using Dorado v5.2.0 HAC model. Finally, we present NanoMnT, a lightweight Python-based tool that performs error-correction for ONT reads in STR regions and estimates STR allele size. We demonstrate the utility of our findings by identifying MSI/MSS status of 4 cancer cell lines from The Singapore Nanopore Expression Data set (SG-NEx) (Chen et al., 2021) and 15 colorectal cancer (CRC) organoids (Pickles et al., 2023), using NanoMnT.

Methods

Identification of STR regions

We employed Krait (v1.3.3, default parameters) (Du et al., 2018), an ultrafast bioinformatic program designed to identify STR from genomes via brute force search algorithm 2 described by Sokol *et al* (http://archive.dimacs.rutgers.edu/Publications/Modules/Module09-2/dimacs09-2.pdf), to search for STR regions within the T2T-CHM13 (v2.0) genome and the HG002 (maternal genome. v1.0.1) genome.

1. T2T-CHM13
   Running Krait on T2T-CHM13 (v2.0) assembly resulted in an initial set of 1,723,161 STR regions. Because many 1bp-/2bp-/3bp-repeat STR possessed low-complex flanking sequences that closely resembled the STR sequences, 1bp-/2bp-/3bp-repeat STR regions whose flanking sequences had severely low k-mer diversity (see Calculation of k-mer diversity in Methods) were filtered out (k-mer ≤ 2.5 for 1bp-repeat, ≤ 2.0 for 2bp-repeat, ≤ 5.0 for 3bp-repeat), as they may introduce ambiguity when measuring STR repeat sizes. This resulted in 1,147,462 STR regions left. Subsequently, regions with a read orientation-specific coverage of at least 20 (i.e., at least 20 forward strand reads, or 20 reverse strand reads) were selected, resulting in the final set of 762,311 STR regions.
2. HG002 (Maternal genome)
   First,1bp-/2bp-/3bp-repeat STR regions were identified from the HG002 maternal assembly using Krait, resulting in an initial set of 980,818 regions. Due to the heterozygosity of HG002, the HG002 assembly contains a considerable number of biallelic STR regions which complicate the analysis of ONT error profile. Therefore, we decided to exclusively use monoallelic STR regions in our analysis by employing LiftOff (v1.6.3, default parameters) to convert genomic coordinates from HG002 paternal assembly to HG002 maternal assembly (Shumate & Salzberg, 2021). During this process, 334,592 STR regions were unable to be converted, resulting in 646,226 remaining STR regions, of which 460,572 were found to be monoallelic. Finally, STR regions whose flanking sequences with low k-mer diversity (same thresholds applied to CHM13 STR regions) and regions with coverage lower than 5 were filtered out, resulting in the final set of 195,182 STR regions.

Data processing and visualization

After downloading FAST5 files and POD5 files from sources specified by the authors, FASTQ files were obtained by employing the appropriate basecaller for each dataset (Supplementary Table 1). We aligned the FASTQ files to the reference genomes using minimap2 (v2.24-r1122) (Li, 2018) with `-ax map-ont` parameters for all data except for SG-NEx data, where `-ax splice` was used instead. As the CHM13 data is the center of our analysis, we filtered out reads with mapping quality below 60 and excluded supplementary reads. Subsequent data analysis and visualization were performed using Seaborn (0.13.0), Matplotlib (3.7.1), Pandas (2.0.0) and Numpy (1.22.4). All datasets analyzed in this study were PCR-free, ensuring the absence of PCR stutters. Moreover, all major datasets – CHM13 data, HG002 data and SG-NEx data– provided raw FAST5/POD5 files, allowing us to compare the influence of basecalling programs and their configurations on STR sequencing accuracy.

Calculation of k-mer diversity

First, the frequency of each k-mer within the given DNA sequence is counted. The counting process involves sliding a window across the DNA sequence by one nucleotide at a time, extracting all possible k-mers and storing their frequency in a dictionary data structure. For example, if the DNA sequence is ATCGC, the 2-mer counting process produces the following Python dictionary: {AT: 1, TC:1, CG:1, GC:1}.

Then, the k-mer diversity is calculated using the following expression:

$$k - mer\ diversity = \frac{L - (k - 1)}{\sum_{i=1}^{L-1} F_i^2}$$

where L is the length of the given DNA sequence ($L - (k - 1)$ equals to the maximum number of k-mer that can be found in the DNA sequence), and $\{F_1, F_2, F_3, F_4 \ldots\}$ represents the frequency of each found k-mer.

CNN prediction of sequencing accuracy using flanking sequences

The flanking sequences (6 nucleotides in each direction of STR, resulting in 12 nucleotides) were one-hot encoded and converted into a Numpy array. STR loci with coverage less than 40 were discarded, and a training-validation ratio of 9:1 was used with the remaining loci. Briefly, we used TensorFlow (Martín Abadi et al., 2015) to implement a sequential neural network featuring a 1D convolutional layer with 48 filters and a kernel size of 2, followed by a flattening layer and two dense layers with 120 and 40 nodes, respectively, both using ReLU activation . The output layer consists of a single node with a sigmoid activation function. Using this model, we predicted the sequencing accuracy of STR regions using the one-hot encoded flanking sequences as inputs. Linear regression was performed to assess the prediction results and visualized using Seaborn's `regplot` function, while Pearson correlation values were calculated using SciPy (1.7.1).

UMAP projection of STR regions

We considered a STR region's sequencing accuracy to be well predicted if it satisfied the following expression:

$$\mu - \frac{1}{2}\sigma \leq (predicted\ accuracy - actual\ accuracy) \leq \mu + \frac{1}{2}\sigma$$

where μ is the mean of differences between predicted and actual accuracies, and σ is the standard deviation these differences. The flanking sequences of these well-predicted STR regions were one-hot encoded and converted into an Anndata (v0.10.6) and subjected to UMAP visualization (Virshup et al., 2023). The following functions and parameters of Scanpy (1.10.0) were used: `sc.pp.neighbors(adata, n_neighbors=15, n_pcs=18)` and `sc.tl.umap(adata, spread=1)` (Wolf et al., 2018).

Benchmarking NanoMnT, NanoRepeat, and NanoSTR

Unlike NanoRepeat and NanoMnT, NanoSTR requires the genomic coordinates of STR regions to be based on the hg19 or the hg38 assembly. Thus, utilizing LiftOff again, we converted the genomic coordinates of STR regions from HG002 maternal assembly to the hg38 assembly, allowing us to use the same STR regions for all three tools in our benchmark. The STR loci used for benchmark are available in Supplementary Table 2. In-house scripts were used to summarize the outputs of each program.

MSI detection of cancer sequencing datasets

Ax10-14 STR loci with coverage above 30 were used for MSI identification, as mononucleotide repeats of these lengths have been shown to be vulnerable to deletion mutations in MMR-deficient cells (Aska et al., 2022). For the SG-NEx dataset, loci not covered in at least 3 out of 4 samples were discarded, while for the CRC organoid WGS dataset, loci not covered in at least 10 out of 15 samples were discarded. For the 'read + loci selection approach' shown in Figure 9a, we filtered out (1) A-repeat STR loci that had guanine nucleotides directly next to the A-repeat tracts, and (2) loci whose allele prominence (calculated by NanoMnT) satisfied the following expression:

$$\mu - \frac{1}{2}\sigma \leq allele\ prominence \leq \mu + \frac{1}{2}\sigma$$

where μ is the mean of allele prominences of all genotyped loci, and σ is the standard deviation these prominences.

Subsequently, we obtained the allele size histogram and calculated the relative allele size of each STR locus using the following expression:

$$\sum_{i=1}^{n}(A_i - R_i)f_i$$

where A_i is the observed allele, R_i is the reference allele (CHM13) and f_i is the frequency of A_i.

Implementation of NanoMnT

NanoMnT provides three functions: (1) error-correction of reads, (2) STR allele size estimation, and (3) informative loci identification.

1. Error correction of individual reads
   NanoMnT collects reads that aligned to the user-provided STR loci using Pysam (v0.20.0) (Bonfield et al., 2021) and realigns them to a modified STR region that excludes the STR sequence itself, consisting only of the STR-flanking regions. This approach prevents alignment bias caused by the reference genome, as minimap2 tends to produce slightly different alignments in STR regions, depending on the reference genome. For each realigned read, the sequences that aligned to the STR regions are extracted and compared against a list of possible alleles by calculating the Levenshtein distance. The allele with the minimum Levenshtein distance is chosen as the most likely allele. If the total Levenshtein distance exceeds a certain threshold, the read is considered excessively erroneous and discarded. This process yields corrected STR alleles for each ONT read, which are then used for subsequent STR allele size estimation.

2. Estimation of STR allele size
   Using the corrected reads, NanoMnT creates an allele size histogram for each locus. The user can decide whether to use all reads or forward/reverse strand reads – which is very beneficial when analyzing A-/T-repeats – when creating allele size histogram. To estimate the STR allele size, NanoMnT generates synthetic allele size histograms for each possible allele and calculates the distance between the observed allele size histogram against each synthetic allele size histogram. The synthetic histogram with the minimum distance to the observed histogram is then chosen as the best match. The allele associated with this chosen histogram is selected as the most probable STR allele. Finally, SciPy's `find_peak` function is used to calculate the prominence of the observed allele size histogram.
3. Informative loci identification
   Given the outputs of NanoMnT (Allele Table and Locus Table, see Figure 8a) of paired normal and tumor samples, NanoMnT finds STR loci that have been sequenced in both sample (namely, commonly covered loci) and compares the STR allele size histogram by calculating the distance between the two histograms. This distance information tells us about the similarity between the STR allele size histogram of two samples; if the similarity is low, this locus may be an indication of MSI phenotype. Lastly, the 'score' of each STR locus is calculated using the following expression: $locus\ score = distance(H_n, H_t)\ \times Peak\ prominenc\ of\ H_n$, where H_n and H_t is the allele size histogram of normal and tumor sample, respectively. This score informs the reliability of each result.

Results

Distribution of sequencing errors in STR regions

We first sought to measure the abundance of each type of sequencing error, i.e., deletions, insertions, and substitutions, by counting the number of errors in ONT reads that aligned to STR regions. The STR regions used in this study were carefully selected as many exhibited excessively low-complex sequences in their flanking regions, which often introduces alignment bias and thus hampers downstream analyses (Methods). The number and distribution of STR analyzed in this study is available in Supplementary Figure 1a. Due to the scarcity of GC-rich STR regions in the human genome, we note that GC-rich STR could not be robustly represented in our analyses (Supplementary Fig. 1b). Furthermore, the sequencing accuracy of STR is measured by calculating the percentage of errorless reads, and these two terms are used interchangeably throughout the study.

We gathered ONT reads that aligned to STR regions and counted the percentage of errorless reads and the percentage of each sequencing error type (Fig. 1a). Overall, the occurrence of error was higher in STR with shorter repeat units, with 1bp-repeats exhibiting particularly high rate of sequencing errors. While indel errors accounted for most of the sequencing errors, deletion errors seemed to be most frequent especially in 1bp-repeats, which is in line with previous reports (Delahaye & Nicolas, 2021; Gunter et al., 2024; Sereika et al., 2022).

To provide a more practical analysis of STR sequencing results, we performed rudimentary polishing of sequencing errors using in-house scripts, as doing so considerably increased the number of reads that can be analyzed. This was

achieved by calculating the Levenshtein distance between the observed STR sequence and a list of possible STR sequences, then selecting the STR sequence with the minimum distance. Reads with distances exceeding 4, which make up ~2.8% of the total reads, were discarded. Using these polished reads, we visualized the distribution of STR allele sizes by generating histograms for various STR alleles. We observed that ONT tends to underestimate STR sizes, causing some histograms to shift slightly left (Fig. 1b, Supplementary Fig. 2). While many histograms exhibit clear peaks that match the actual STR alleles, prominent peaks could not be generated for 1bp-repeats and other types of longer STR.

Sequencing accuracy of STR across different repeat units and lengths

ONT sequencing measures the change in electric current applied to the membrane embedded nanopore to determine the DNA sequence (Wang et al., 2021). Given that only one strand of the double-stranded DNA enters the nanopore, we wondered whether the repeat units of the STR would influence the sequencing accuracy. Thus, we calculated the percentage of errorless reads for each type of STR based on its repeat unit and lengths. In this process, we separated forward strand reads (reads that map to the forward strand of the reference genome) from reverse strand reads (reads that map to the reverse strand of the reference genome), as these two groups of reads have had different types of repeat tracts sequenced. For example, the error profile of forward strand reads originating from an A-repeat STR locus may differ from that of reverse strand reads at the same locus because the former set of reads encompasses the sequencing of A-repeats, while the latter encompasses the sequencing of T-repeats. As a result, we found that the sequencing accuracy varied substantially among STR with differing repeat units (Fig. 2a). A-repeats were generally better sequenced than other 1bp-repeats, whereas in 2bp-repeats, AT/TA-repeats were better sequenced than other 2bp-repeats. However, we emphasize that this trend only applies in a general sense, as there are considerable exceptions (Supplementary Fig. 3). We validated this hierarchy of sequencing accuracy among repeat units using the HG002 R9.4.1 dataset and confirmed very similar results (Fig. 2b). For STR with longer repeat units, most STR displayed much better accuracy, although it should be noted that their scarce nature limited our analysis to their relatively shorter forms (Supplementary Fig. 4). Moreover, we noticed substantial variability of sequencing accuracy among STR, even among STR with identical repeat units and lengths (Fig. 2c).

Relationship between STR sequencing accuracy and flanking sequences

To explain the variability in sequencing accuracy among identical STR types (as seen in Figure 2c), we hypothesized that the flanking sequences of STR may influence the sequencing accuracy. We tested our hypothesis by training a convolutional neural network (CNN) machine learning model using the flanking sequences of Ax10 STR regions, 6 nucleotides for each direction (adding up to 12 nucleotides) to predict the sequencing accuracy of Ax10 STR regions (n=23,393) (Methods, Fig. 3). The model displayed considerable predictive accuracy, as shown by the Pearson correlation value of 0.66. Upon repeating the same process using only using either left or right flanking sequences, we observed markedly lower Pearson correlation value, suggesting that the flanking sequences of both directions influence the sequencing accuracy of STR (Supplementary Fig. 5a). The STR accuracy of 2bp-repeats such as ATx8 (n=4,636) and ACx8 (n=3,263) repeats was also moderately predicted by our model (Fig. 3b), indicating that the association between sequencing accuracy and flanking sequences is not limited to Ax10, but extends to other STR types as well. However, despite these results, the presence of outliers in CNN prediction implies that flanking sequences alone do not fully dictate the sequencing accuracy of STR regions. Indeed, while STR regions with identical flanking sequences

generally exhibit similar sequencing accuracy, noticeable variation remains evident among them (Supplementary Fig. 5b).

Nonetheless, motivated by this finding, we attempted to identify specific motifs which the CNN model associated with good/poor sequencing accuracy, by analyzing well-predicted Ax10~Ax15 STR regions and subjecting them to UMAP projection (Methods). After labeling each A-repeat STR by its sequencing accuracy onto the UMAP, we found a very interesting pattern: the sequencing accuracy seemed to be associated mainly with the nucleotides closest to the A-repeats (Fig. 4a). Most notably, A-repeats with two guanines flanking each side consistently exhibited poor sequencing accuracy. Furthermore, the distance between the flanking nucleotides and the A-repeats seemed to be inversely proportional to the influence of the flankings on sequencing accuracy (Supplementary Fig. 6a). We identified top 20 motifs and worst 20 motifs of A-repeats (defined by the left 2 nucleotides and the right 2 nucleotides that flank the A-repeats) regarding sequencing accuracy, whose effects were relatively consistent across varying lengths of A-repeats (Fig. 4b). We found that the top motifs were enriched with pyrimidine bases, whereas worst motifs were enriched with purine bases (Supplementary Fig. 6b). We validated our results using HG002 R9.4.1 dataset and obtained concordant results (Supplementary Fig. 6c).

The impact of basecaller on STR sequencing accuracy

One of the unique aspects of ONT data analysis is the basecalling process, which applies machine learning to convert electric signals into nucleotide sequences. Basecallers are regularly updated, allowing users to reanalyze their data using different basecaller versions. To explore the impact of basecallers on STR sequencing accuracy, we compared the performance of 4 basecaller versions: Guppy v5.0.7, Guppy v6.0.0, Guppy v6.5.7 (the final Guppy version) and Dorado v5.2.0 (the latest Dorado basecaller as of this study) (Fig. 5a). High accuracy (HAC) model was used for all 4 basecaller versions. As a result, we found that Guppy v6.5.7 and Dorado v5.2.0 both vastly outperformed the other two Guppy versions, demonstrating the importance of applying the most up-to-date basecaller. Next, we compared the influence of high accuracy (HAC) model against super accuracy (SUP) model within Guppy v6.5.7 and observed considerable improvements (Fig. 5b). Given that the SUP model is known to offer only marginal improvements over the HAC model, this improvement was unexpectedly significant. However, this improvement was not uniformly applied to the data: while 70.6% of Ax10 STR regions were better resolved using the SUP model, the remaining 29.4% regions were better resolved with the HAC model (Fig. 5c). Nevertheless, in general, choosing the latest version and model of basecaller enables significant benefit for STR analysis.

Base quality score of sequencing error in STR regions

Next, we sought to examine whether the elevated error rate in STR regions is reflected onto the base quality score by analyzing the base quality scores of bases within STR regions. First, we compared the average base quality scores of correctly sequenced reads against incorrectly sequenced reads and noted marginal differences (Fig. 6a). However, a striking overestimation of the base quality scores of bases within the STR regions was observed regardless of the presence of errors, given that the average base quality score within the entire CHM13 dataset was estimated to be approximately 20.67. Consequently, we explored the distribution of base quality scores of bases within and adjacent to

STR regions (Supplementary Fig. 7a). We observed ‘bursts’ of quality scores to an abnormally high value, and while this phenomenon was observed in 1bp-, 2bp-, 3bp-repeat STR, it was most evident in 2bp-repeat STR. For the majority of 2bp-repeat STR, the basecaller consistently assigned a fixed value of 90 as the base quality score for bases within the STR regions. Figure 6b shows the base quality score distribution within a ACx12 STR locus (chr10:25491367-25491390, T2T-CHM13v2.0), which well demonstrates the typical base quality score distribution within 2bp-repeat STR regions. Notably, prominent quality score bursts were not observed in 4bp-, 5bp-, and 6bp-repeat STR.

We also examined the base quality scores of sequencing errors to assess their potential utility in sequencing error inference. The overall base quality scores of substitution errors within STR regions were markedly lower than those of correct bases (Fig. 6c). On the contrary, the differences between base quality scores between correctly sequenced reads and reads harboring indel errors were unnoticeable, which was disappointing, considering that indel errors account for most sequencing errors (Supplementary Fig. 7b). We validated these findings by applying the same analysis on the HG002 R9.4.1 dataset (Supplementary Fig. 8).

STR error profile of R10.4.1 flowcell.

We expanded our analysis by comparing the HG002 R10.4.1 dataset against the HG002 R9.4.1 dataset. We show that the error profile of R10.4.1 resembles that of R9.4.1 (Fig. 7a). Although overall more accurate, indel errors still accounted for the majority of sequencing errors. R10.4.1 also showed improvements in almost all STR types, especially in GC-rich STR (Fig. 7b). We performed similar analyses performed throughout the study on the HG002 R10.4.1 dataset and show that all the topics discussed in this article – sequencing accuracy of various STR types, impact of basecallers, association of flanking sequences with sequencing accuracy – are largely maintained in R10.4.1 as well (Supplementary Fig. 9-11).

Development of NanoMnT

Although existing tools such as NanoRepeat and NanoSTR excel in genotyping 3bp-, 4bp-, 5bp-, and 6bp-repeats, it is our understanding that they are not designed for analyzing 2bp- and, especially 1bp-repeats. Thus, we developed NanoMnT, a lightweight Python-based tool that (1) corrects STR sequencing errors for ONT reads, (2) estimate allele sizes of user-specified STR loci using the corrected reads, and (3) given the output files for paired normal and tumor samples, searches for informative STR loci (Methods, Fig. 8a). We tested the performance of NanoMnT against NanoRepeat and NanoSTR and confirmed that NanoMnT provides better STR allele size estimation for 1bp-repeats and 2bp-repeats (Fig. 8b, Supplementary Fig. 12a). NanoMnT provides the prominence of the allele histogram peak, which can be used as quality measure, with high peak prominence generally corresponding to confident allele estimation results (Fig. 8c, Supplementary Fig. 12b-c).

MSI detection of cancer samples from ONT data using NanoMnT

We integrated our findings into a biological context by identifying MSI/MSS status of cancer samples from the bulk RNA-sequencing dataset created by SG-NEx. Among the various types of ONT sequencing dataset provided by SG-NEx, we chose PCR-free direct cDNA sequencing data to ensure the absence of PCR stutter. Conventionally, the MSI/MSS status is often determined by comparing the STR allele size histograms of the tumor sample with those of the corresponding normal sample. Unfortunately, due to the absence of matched normal sample, we employed the CHM13 genome as a substitute ‘normal’ sample. We calculated the relative allele sizes of STR loci and compared their distribution among each sample to identify MSI and MSS cancers (Methods). To showcase the importance of bioinformatics strategies for analyzing ONT data, we compared the MSI/MSS identification results derived from 3 distinct versions of FASTQ data obtained from the same sample: (1) raw FASTQ files provided by SG-NEx which were basecalled using Guppy version 3.2.10; (2) data re-basecalled using the latest version of Guppy, version 6.5.7 (HAC); and (3) data re-basecalled using Guppy version 6.5.7 (HAC), while applying read-selection and STR loci-selection process to achieve better accuracy (Methods, Fig. 9a). The overall STR allele sizes of the MSI cell line were overall shorter than those of MSS cell lines, consistent with previous reports indicating the predominance of deletion mutations in mononucleotide repeats within MSI (Aska et al., 2022). While simply re-basecalling the data with Guppy v6.5.7 significantly separated the MSI cell line from the MSS cell lines, the read selection and/or loci selection step gave markedly better results (Fig. 9b).

We performed a similar analysis on the CRC organoid dataset created by Pickles et al, which performed WGS on 15 primary CRC organoids, each labeled with MMR status and consensus molecular subtype (CMS) (Fig. 9c). While we could not re-basecall this dataset with the latest basecaller due to the unavailability of raw FAST5/POD5 files, we still show that dMMR and pMMR status could be well identified, apart from sample 064 and 080. Although these two discordant results may be false positives, it is possible that these results may reflect the intratumoral heterogeneity of CRC. Indeed, several studies have reported coexistence of CMS1 – which is almost exclusively enriched in MSI CRC – and other CMS CRC within individual patients(Guinney et al., 2015; Lee et al., 2020; Valdeolivas et al., 2024).

Discussion

The capacity of ONT to generate long reads along with its portability and versatility makes it an attractive approach for countless research fields. Despite these benefits, the excessive error rate of ONT in low-complexity regions hinders its application in STR related fields. This study provides a comprehensive overview of ONT sequencing profile in STR regions by measuring the abundance of sequencing errors in various STR types and identifying factors that influence STR sequencing accuracy. We observed that indels were responsible for most of the sequencing errors, with deletions generally more prevalent than insertions. The excess number of insertions and substitutions renders raw ONT data critically inadequate for analyzing STR regions. In addition, the overestimation of Phred quality score may suggest that the basecaller machine learning models are not properly tailored for STR regions. Moreover, while Phred quality scores of substitution error bases and correct bases differed significantly, – suggesting the potential of Phred quality score in inferring substitution errors – we could not observe such difference between indel errors and correctly sequenced bases, which is unfortunate, considering the abundance of indel errors.

In this study, we identified 3 factors that influence the sequencing accuracy of STR. First, the sequencing accuracy of STR was shown to be heavily influenced by the repeat unit of the sequenced STR; that is, the nucleotides that entered the nanopore proteins. This finding suggests a strategic approach when analyzing STR from ONT data; preferential usage of reads with specific orientation over reads with opposite orientation may achieve superior accuracy, given that the sequencing depth is sufficiently high. Such influence of repeat units was consistently observed in both versions of flowcells (R9.4.1 and R10.4.1) and basecalling programs, indicating that the electric signals associated with some repeat units may be intrinsically more resolvable for ONT compared to others. Second, flanking sequences were also associated with the sequencing accuracy of STR regions, implying that careful selection of STR loci based on their flanking sequences may mitigate the high error rate of ONT. For example, purine-rich flanking sequences were linked to worse sequencing accuracy in A-repeat STR regions. This could be due to the high similarity of electric signals produced by both the A-repeats and the purine-rich flanking sequences. Thirdly, we highlight the significance of basecaller version, which is possibly the most influential factor of sequencing accuracy, as shown in Figure 5. Therefore, we encourage researchers who have previously generated ONT sequencing data to re-analyze using the latest basecaller if they are interested in STR related analyses, such as MSI identification.

We also introduced NanoMnT, a lightweight Python-based tool that performs error correction in STR regions by choosing the most parsimonious allele, i.e., allele with the minimum Levenshtein distance compared to the observed allele, and genotypes STR regions using these corrections. Although there are existing tools that serve similar purposes, none of them have been designed to genotype 1bp- and 2bp-repeat STR. Instead, to the best of our knowledge, most tools are designed to genotype STR with longer repeat units to study areas such as neurological disease (Fang et al., 2023) and forensics (Lang et al., 2023). By applying NanoMnT on two cancer datasets, we were able to identify MSI status of various cancer samples.

We acknowledge several limitations of this study. First, our study was solely focused on perfect tandem repeats, excluding many types repeats such as compound repeats, imperfect tandem repeats. Second, even though we identified certain motifs enriched in well-/poorly sequenced A-repeat STR, we failed to provide a comprehensive mechanism that explains the influence of flanking sequences on STR sequencing accuracy. Also, the CNN machine learning model did not exhibit optimal predictive accuracy, indicating the presence of additional factors that we could not detect and/or the stochastic nature of ONT error profile. We note that the lack of diversity of flanking sequences

within the human genome – since a major portion of A-/T-repeat STR originates from mobile genetic elements such as Alu elements – may have exacerbated the CNN prediction results. Thus, using a sufficiently diverse set of flanking sequences may improve our understanding of the association between flanking sequences and sequencing accuracy.

Data Availability

The sequencing dataset generate for CHM13 was accessed via the Telomere-to-Telomere consortium CHM13 project GitHub page, using the following link: https://github.com/marbl/CHM13/blob/master/Sequencing_data.md. Sequencing datasets for HG002 were accessed through the Dataset Releases presented by EPI2ME, using the following links: https://labs.epi2me.io/gm24385_2020.09/ (R9.4.1) and https://labs.epi2me.io/giab-2023.05/ (R10.4.1). SG-NEx RNA-seq dataset was accessed through SG-NEx GitHub page through the following link: https://github.com/GoekeLab/sg-nex-data and WGS dataset of CRC organoids generated by Pickles et al was downloaded from NCBI (PRJNA978372).

Supplementary Data statement

Supplementary Data are available at NAR Online

Acknowledgements

Funding

This work was supported GIST-CNUH Research Collaboration grant and GIST-MIT Research collaboration grant funded by the GIST in 2024, and the National Research Foundation of Korea (NRF), funded by the Korean government (RS-2024-00335026)

Conflict of Interest Disclosure

None

References

Alonso, A., Barrio, P. A., Muller, P., Kocher, S., Berger, B., Martin, P., Bodner, M., Willuweit, S., Parson, W., Roewer, L., & Budowle, B. (2018). Current state-of-art of STR sequencing in forensic genetics. *Electrophoresis*, *39*(21), 2655-2668. https://doi.org/10.1002/elps.201800030

Aska, E. M., Zagidullin, B., Pitkanen, E., & Kauppi, L. (2022). Single-Cell Mononucleotide Microsatellite Analysis Reveals Differential Insertion-Deletion Dynamics in Mouse T Cells. *Front Genet*, *13*, 913163. https://doi.org/10.3389/fgene.2022.913163

Bonfield, J. K., Marshall, J., Danecek, P., Li, H., Ohan, V., Whitwham, A., Keane, T., & Davies, R. M. (2021). HTSlib: C library for reading/writing high-throughput sequencing data. *Gigascience*, *10*(2). https://doi.org/10.1093/gigascience/giab007

Brinkmann, B., Klintschar, M., Neuhuber, F., Huhne, J., & Rolf, B. (1998). Mutation rate in human microsatellites: influence of the structure and length of the tandem repeat. *Am J Hum Genet*, *62*(6), 1408-1415. https://doi.org/10.1086/301869

Bruford, M. W., & Wayne, R. K. (1993). Microsatellites and their application to population genetic studies. *Curr Opin Genet Dev*, *3*(6), 939-943. https://doi.org/10.1016/0959-437x(93)90017-j

Chang, L., Chang, M., Chang, H. M., & Chang, F. (2018). Microsatellite Instability: A Predictive Biomarker for Cancer Immunotherapy. *Appl Immunohistochem Mol Morphol*, *26*(2), e15-e21. https://doi.org/10.1097/PAI.0000000000000575

Chen, Y., Davidson, N., Wan, Y. K., Patel, H., Yao, F., Low, H. M., Hendra, C., Watten, L., Sim, A., Sawyer, C., Iakovleva, V., Lee, P. L., Xin, L., Ng, H. E. V., Loo, J. M., Ong, X., Ng, H. Q. A., Wang, J., Koh, W. Q. C., . . . consortium, S. G. N. (2021). A systematic benchmark of Nanopore long read RNA sequencing for transcript level analysis in human cell lines. In: bioRxiv.

Delahaye, C., & Nicolas, J. (2021). Sequencing DNA with nanopores: Troubles and biases. *PLoS One*, *16*(10), e0257521. https://doi.org/10.1371/journal.pone.0257521

Du, L., Zhang, C., Liu, Q., Zhang, X., Yue, B., & Hancock, J. (2018). Krait: an ultrafast tool for genome-wide survey of microsatellites and primer design. *Bioinformatics*, *34*(4), 681-683. https://doi.org/10.1093/bioinformatics/btx665

Fang, L., Monteys, A. M., Durr, A., Keiser, M., Cheng, C., Harapanahalli, A., Gonzalez-Alegre, P., Davidson, B. L., & Wang, K. (2023). Haplotyping SNPs for allele-specific gene editing of the expanded huntingtin allele using long-read sequencing. *HGG Adv*, *4*(1), 100146. https://doi.org/10.1016/j.xhgg.2022.100146

Fotsing, S. F., Margoliash, J., Wang, C., Saini, S., Yanicky, R., Shleizer-Burko, S., Goren, A., & Gymrek, M. (2019). The impact of short tandem repeat variation on gene expression. *Nat Genet*, *51*(11), 1652-1659. https://doi.org/10.1038/s41588-019-0521-9

Guinney, J., Dienstmann, R., Wang, X., de Reynies, A., Schlicker, A., Soneson, C., Marisa, L., Roepman, P., Nyamundanda, G., Angelino, P., Bot, B. M., Morris, J. S., Simon, I. M., Gerster, S., Fessler, E., De Sousa, E. M. F., Missiaglia, E., Ramay, H., Barras, D., . . . Tejpar, S. (2015). The consensus molecular subtypes of colorectal cancer. *Nat Med*, *21*(11), 1350-1356. https://doi.org/10.1038/nm.3967

Gunter, H. M., Youlten, S. E., Reis, A. L. M., McCubbin, T., Madala, B. S., Wong, T., Stevanovski, I., Cipponi, A., Deveson, I. W., Santini, N. S., Kummerfeld, S., Croucher, P. I., Marcellin, E., & Mercer, T. R. (2024). A universal molecular control for DNA, mRNA and protein expression. *Nat Commun*, *15*(1), 2480. https://doi.org/10.1038/s41467-024-46456-9

Gymrek, M. (2017). A genomic view of short tandem repeats. *Curr Opin Genet Dev*, *44*, 9-16. https://doi.org/10.1016/j.gde.2017.01.012

Lang, J., Xu, Z., Wang, Y., Sun, J., & Yang, Z. (2023). NanoSTR: A method for detection of target short tandem repeats based on nanopore sequencing data. *Front Mol Biosci*, *10*, 1093519. https://doi.org/10.3389/fmolb.2023.1093519

Lee, H. O., Hong, Y., Etlioglu, H. E., Cho, Y. B., Pomella, V., Van den Bosch, B., Vanhecke, J., Verbandt, S., Hong, H., Min, J. W., Kim, N., Eum, H. H., Qian, J., Boeckx, B., Lambrechts, D., Tsantoulis, P., De Hertogh, G., Chung, W., Lee, T., . . . Park, W. Y. (2020). Lineage-dependent gene expression programs influence the immune landscape of colorectal cancer. *Nat Genet*, *52*(6), 594-603. https://doi.org/10.1038/s41588-020-0636-z

Li, H. (2018). Minimap2: pairwise alignment for nucleotide sequences. *Bioinformatics*, *34*(18), 3094-3100. https://doi.org/10.1093/bioinformatics/bty191

Li, K., Luo, H., Huang, L., Luo, H., & Zhu, X. (2020). Microsatellite instability: a review of what the oncologist should know. *Cancer Cell Int*, *20*, 16. https://doi.org/10.1186/s12935-019-1091-8

Liu, Q., Tong, Y., & Wang, K. (2020). Genome-wide detection of short tandem repeat expansions by long-read sequencing. *BMC Bioinformatics*, *21*(Suppl 21), 542. https://doi.org/10.1186/s12859-020-03876-w

Malik, I., Kelley, C. P., Wang, E. T., & Todd, P. K. (2021). Molecular mechanisms underlying nucleotide repeat expansion disorders. *Nat Rev Mol Cell Biol*, *22*(9), 589-607. https://doi.org/10.1038/s41580-021-00382-6

Martín Abadi, A. A., Paul Barham, Eugene Brevdo,, Zhifeng Chen, C. C., Greg S. Corrado, Andy Davis,, Jeffrey Dean, M. D., Sanjay Ghemawat, Ian Goodfellow,, Andrew Harp, G. I., Michael Isard, Rafal Jozefowicz, Yangqing Jia,, Lukasz Kaiser, M. K., Josh Levenberg, Dan Mané, Mike Schuster,, Rajat Monga, S. M., Derek Murray, Chris Olah, Jonathon Shlens,, Benoit Steiner, I. S., Kunal Talwar, Paul Tucker,, Vincent Vanhoucke, V. V., Fernanda Viégas,, Oriol Vinyals, P. W., Martin Wattenberg, Martin Wicke,, & Yuan Yu, a. X. Z. (2015). TensorFlow: Large-scale machine learning on heterogeneous systems. *tensorflow2015-whitepaper*. https://www.tensorflow.org/

Pickles, O. J., Wanigasooriya, K., Ptasinska, A., Patel, A. J., Robbins, H. L., Bryer, C., Whalley, C. M., Tee, L., Lal, N., Pinna, C. M. A., Elzefzafy, N., Taniere, P., Beggs, A. D., & Middleton, G. M. (2023). MHC Class II is Induced by IFNgamma and Follows Three Distinct Patterns of Expression in Colorectal Cancer Organoids. *Cancer Res Commun*, *3*(8), 1501-1513. https://doi.org/10.1158/2767-9764.CRC-23-0091

Rhoads, A., & Au, K. F. (2015). PacBio Sequencing and Its Applications. *Genomics Proteomics Bioinformatics*, *13*(5), 278-289. https://doi.org/10.1016/j.gpb.2015.08.002

Sereika, M., Kirkegaard, R. H., Karst, S. M., Michaelsen, T. Y., Sorensen, E. A., Wollenberg, R. D., & Albertsen, M. (2022). Oxford Nanopore R10.4 long-read sequencing enables the generation of near-finished bacterial genomes from pure cultures and metagenomes without short-read or reference polishing. *Nat Methods*, *19*(7), 823-826. https://doi.org/10.1038/s41592-022-01539-7

Shumate, A., & Salzberg, S. L. (2021). Liftoff: accurate mapping of gene annotations. *Bioinformatics*, *37*(12), 1639-1643. https://doi.org/10.1093/bioinformatics/btaa1016

Suraweera, N., Duval, A., Reperant, M., Vaury, C., Furlan, D., Leroy, K., Seruca, R., Iacopetta, B., & Hamelin, R. (2002). Evaluation of tumor microsatellite instability using five quasimonomorphic mononucleotide repeats and pentaplex PCR. *Gastroenterology*, *123*(6), 1804-1811. https://doi.org/10.1053/gast.2002.37070

Valdeolivas, A., Amberg, B., Giroud, N., Richardson, M., Galvez, E. J. C., Badillo, S., Julien-Laferriere, A., Turos, D., Voith von Voithenberg, L., Wells, I., Pesti, B., Lo, A. A., Yanguez, E., Das Thakur, M., Bscheider, M., Sultan, M., Kumpesa, N., Jacobsen, B., Bergauer, T., . . . Hahn, K. (2024). Profiling the heterogeneity of colorectal cancer consensus molecular subtypes using spatial transcriptomics. *NPJ Precis Oncol*, *8*(1), 10. https://doi.org/10.1038/s41698-023-00488-4

Virshup, I., Bredikhin, D., Heumos, L., Palla, G., Sturm, G., Gayoso, A., Kats, I., Koutrouli, M., Scverse, C., Berger, B., Pe'er, D., Regev, A., Teichmann, S. A., Finotello, F., Wolf, F. A., Yosef, N., Stegle, O., & Theis, F. J. (2023). The scverse project provides a computational ecosystem for single-cell omics data analysis. *Nat Biotechnol*, *41*(5), 604-606. https://doi.org/10.1038/s41587-023-01733-8

Wang, Y., Zhao, Y., Bollas, A., Wang, Y., & Au, K. F. (2021). Nanopore sequencing technology, bioinformatics and applications. *Nat Biotechnol*, *39*(11), 1348-1365. https://doi.org/10.1038/s41587-021-01108-x

Wei, N., Bemmels, J. B., & Dick, C. W. (2014). The effects of read length, quality and quantity on microsatellite discovery and primer development: from Illumina to PacBio. *Mol Ecol Resour*, *14*(5), 953-965. https://doi.org/10.1111/1755-0998.12245
Wolf, F. A., Angerer, P., & Theis, F. J. (2018). SCANPY: large-scale single-cell gene expression data analysis. *Genome Biol*, *19*(1), 15. https://doi.org/10.1186/s13059-017-1382-0

Supplementary Tables Click here to access/download;Table;Supplementary tables.xlsx

Supplementary Table 1. Metadata of all dataset used in this study

Dataset	Flowcell version	Type	Provides FAST5/POD5
CHM13	R9.4.1	WGS	Yes
HG002			
SG-NEx		RNA-seq	
CRC WGS data (Pickles et al)		WGS	No
HG002	R10.4.1	WGS	Yes

Basecaller	Basecaller version	Basecaller model
Guppy	v5.0.7	HAC
	v6.0.0	HAC
	v6.5.7	HAC
		SUP
Dorado	v5.2.0	HAC
Guppy	v6.5.7	HAC
		SUP
Guppy	v6.5.7	HAC
Guppy	-	-
Dorado	v6.5.7	HAC
		SUP

Supplementary Table 2. STR loci used for tool benchmarking

hg38 coordinates	hg002 coordinates
chrX_MATERNAL:48470648-48470657	chrX_MATERNAL:48470648-48470657
chr7_MATERNAL:147931948-147931957	chr7_MATERNAL:147931948-147931957
chrX_MATERNAL:76370968-76370977	chrX_MATERNAL:76370968-76370977
chr8_MATERNAL:16097059-16097068	chr8_MATERNAL:16097059-16097068
chr2_MATERNAL:88654871-88654880	chr2_MATERNAL:88654871-88654880
chrX_MATERNAL:123654897-123654906	chrX_MATERNAL:123654897-123654906
chr5_MATERNAL:111147226-111147235	chr5_MATERNAL:111147226-111147235
chr3_MATERNAL:97800380-97800390	chr3_MATERNAL:97800380-97800390
chr6_MATERNAL:94105591-94105600	chr6_MATERNAL:94105591-94105600
chr2_MATERNAL:170885816-170885825	chr2_MATERNAL:170885816-170885825
chr5_MATERNAL:93964046-93964055	chr5_MATERNAL:93964046-93964055
chr12_MATERNAL:31014818-31014827	chr12_MATERNAL:31014818-31014827
chr12_MATERNAL:57929358-57929367	chr12_MATERNAL:57929358-57929367
chr11_MATERNAL:4033766-4033775	chr11_MATERNAL:4033766-4033775
chr17_MATERNAL:55906960-55906969	chr17_MATERNAL:55906960-55906969
chr2_MATERNAL:58214088-58214097	chr2_MATERNAL:58214088-58214097
chr11_MATERNAL:5303774-5303783	chr11_MATERNAL:5303774-5303783
chr5_MATERNAL:92687495-92687504	chr5_MATERNAL:92687495-92687504
chr19_MATERNAL:40922846-40922855	chr19_MATERNAL:40922846-40922855
chr6_MATERNAL:134943138-134943147	chr6_MATERNAL:134943138-134943147
chr12_MATERNAL:80736022-80736031	chr12_MATERNAL:80736022-80736031
chr1_MATERNAL:70397884-70397893	chr1_MATERNAL:70397884-70397893
chrX_MATERNAL:86464549-86464558	chrX_MATERNAL:86464549-86464558
chr9_MATERNAL:31776036-31776046	chr9_MATERNAL:31776036-31776046
chr8_MATERNAL:133473423-133473432	chr8_MATERNAL:133473423-133473432
chrX_MATERNAL:93475620-93475629	chrX_MATERNAL:93475620-93475629
chr15_MATERNAL:40622338-40622347	chr15_MATERNAL:40622338-40622347
chr5_MATERNAL:99692829-99692838	chr5_MATERNAL:99692829-99692838
chr13_MATERNAL:49966503-49966512	chr13_MATERNAL:49966503-49966512
chr15_MATERNAL:55464688-55464697	chr15_MATERNAL:55464688-55464697
chr1_MATERNAL:207156988-207156997	chr1_MATERNAL:207156988-207156997
chr17_MATERNAL:19653029-19653038	chr17_MATERNAL:19653029-19653038
chr17_MATERNAL:73392104-73392113	chr17_MATERNAL:73392104-73392113
chr12_MATERNAL:53532777-53532786	chr12_MATERNAL:53532777-53532786
chr1_MATERNAL:230552375-230552384	chr1_MATERNAL:230552375-230552384
chrX_MATERNAL:76189748-76189757	chrX_MATERNAL:76189748-76189757
chr5_MATERNAL:58890015-58890024	chr5_MATERNAL:58890015-58890024
chr4_MATERNAL:64327810-64327819	chr4_MATERNAL:64327810-64327819
chr4_MATERNAL:152629758-152629767	chr4_MATERNAL:152629758-152629767
chr8_MATERNAL:53362849-53362858	chr8_MATERNAL:53362849-53362858
chr1_MATERNAL:99655237-99655246	chr1_MATERNAL:99655237-99655246
chrX_MATERNAL:139433195-139433204	chrX_MATERNAL:139433195-139433204
chr1_MATERNAL:5779065-5779074	chr1_MATERNAL:5779065-5779074
chr16_MATERNAL:48440991-48441000	chr16_MATERNAL:48440991-48441000
chr4_MATERNAL:127586793-127586802	chr4_MATERNAL:127586793-127586802

chr2_MATERNAL:101938968-101938977	chr2_MATERNAL:101938968-101938977
chr7_MATERNAL:54077967-54077976	chr7_MATERNAL:54077967-54077976
chr9_MATERNAL:125935988-125935998	chr9_MATERNAL:125935988-125935998
chr12_MATERNAL:45035161-45035170	chr12_MATERNAL:45035161-45035170
chr8_MATERNAL:25870856-25870865	chr8_MATERNAL:25870856-25870865
chr12_MATERNAL:21542238-21542248	chr12_MATERNAL:21542238-21542248
chr3_MATERNAL:14388680-14388690	chr3_MATERNAL:14388680-14388690
chr4_MATERNAL:94697336-94697346	chr4_MATERNAL:94697336-94697346
chr10_MATERNAL:61345689-61345699	chr10_MATERNAL:61345689-61345699
chr10_MATERNAL:132894108-132894118	chr10_MATERNAL:132894108-132894118
chr12_MATERNAL:66717555-66717565	chr12_MATERNAL:66717555-66717565
chr17_MATERNAL:32264302-32264312	chr17_MATERNAL:32264302-32264312
chr7_MATERNAL:148779621-148779631	chr7_MATERNAL:148779621-148779631
chr4_MATERNAL:21057484-21057494	chr4_MATERNAL:21057484-21057494
chr4_MATERNAL:174105983-174105993	chr4_MATERNAL:174105983-174105993
chr9_MATERNAL:71803189-71803198	chr9_MATERNAL:71803189-71803198
chr12_MATERNAL:67667139-67667149	chr12_MATERNAL:67667139-67667149
chr5_MATERNAL:133498123-133498133	chr5_MATERNAL:133498123-133498133
chrX_MATERNAL:73329442-73329452	chrX_MATERNAL:73329442-73329452
chr18_MATERNAL:67597310-67597320	chr18_MATERNAL:67597310-67597320
chr2_MATERNAL:82186026-82186035	chr2_MATERNAL:82186026-82186035
chr2_MATERNAL:50248145-50248155	chr2_MATERNAL:50248145-50248155
chr3_MATERNAL:96422861-96422871	chr3_MATERNAL:96422861-96422871
chr1_MATERNAL:45853134-45853144	chr1_MATERNAL:45853134-45853144
chr2_MATERNAL:190431589-190431600	chr2_MATERNAL:190431589-190431600
chr11_MATERNAL:82126100-82126110	chr11_MATERNAL:82126100-82126110
chrX_MATERNAL:131543007-131543017	chrX_MATERNAL:131543007-131543017
chr5_MATERNAL:152903281-152903291	chr5_MATERNAL:152903281-152903291
chr3_MATERNAL:125739408-125739418	chr3_MATERNAL:125739408-125739418
chr5_MATERNAL:118878237-118878247	chr5_MATERNAL:118878237-118878247
chr6_MATERNAL:105302103-105302113	chr6_MATERNAL:105302103-105302113
chr12_MATERNAL:87884468-87884478	chr12_MATERNAL:87884468-87884478
chr20_MATERNAL:61498375-61498385	chr20_MATERNAL:61498375-61498385
chr2_MATERNAL:19801639-19801649	chr2_MATERNAL:19801639-19801649
chr1_MATERNAL:104140311-104140320	chr1_MATERNAL:104140311-104140320
chr16_MATERNAL:52987220-52987230	chr16_MATERNAL:52987220-52987230
chr12_MATERNAL:126116784-126116794	chr12_MATERNAL:126116784-126116794
chr1_MATERNAL:5598408-5598418	chr1_MATERNAL:5598408-5598418
chr15_MATERNAL:81375841-81375851	chr15_MATERNAL:81375841-81375851
chr4_MATERNAL:55101723-55101735	chr4_MATERNAL:55101723-55101735
chr4_MATERNAL:166071015-166071025	chr4_MATERNAL:166071015-166071025
chr11_MATERNAL:83464797-83464807	chr11_MATERNAL:83464797-83464807
chr6_MATERNAL:151053380-151053390	chr6_MATERNAL:151053380-151053390
chr12_MATERNAL:83086864-83086874	chr12_MATERNAL:83086864-83086874
chr7_MATERNAL:83355416-83355427	chr7_MATERNAL:83355416-83355427
chr6_MATERNAL:67711817-67711827	chr6_MATERNAL:67711817-67711827
chr9_MATERNAL:25993299-25993309	chr9_MATERNAL:25993299-25993309

chr17_MATERNAL:36679903-36679913	chr17_MATERNAL:36679903-36679913
chr14_MATERNAL:69854791-69854801	chr14_MATERNAL:69854791-69854801
chr13_MATERNAL:69039045-69039056	chr13_MATERNAL:69039045-69039056
chr6_MATERNAL:128109404-128109414	chr6_MATERNAL:128109404-128109414
chr1_MATERNAL:208039072-208039082	chr1_MATERNAL:208039072-208039082
chr9_MATERNAL:91699792-91699802	chr9_MATERNAL:91699792-91699802
chr2_MATERNAL:34282069-34282079	chr2_MATERNAL:34282069-34282079
chr16_MATERNAL:273912-273922	chr16_MATERNAL:273912-273922
chr22_MATERNAL:47626705-47626716	chr22_MATERNAL:47626705-47626716
chr12_MATERNAL:4318463-4318474	chr12_MATERNAL:4318463-4318474
chr5_MATERNAL:141370583-141370594	chr5_MATERNAL:141370583-141370594
chr20_MATERNAL:55518226-55518237	chr20_MATERNAL:55518226-55518237
chr4_MATERNAL:181879006-181879017	chr4_MATERNAL:181879006-181879017
chr3_MATERNAL:138927065-138927076	chr3_MATERNAL:138927065-138927076
chr18_MATERNAL:69458403-69458414	chr18_MATERNAL:69458403-69458414
chr12_MATERNAL:24243870-24243881	chr12_MATERNAL:24243870-24243881
chrX_MATERNAL:93589419-93589429	chrX_MATERNAL:93589419-93589429
chr8_MATERNAL:86009503-86009514	chr8_MATERNAL:86009503-86009514
chr2_MATERNAL:142572686-142572697	chr2_MATERNAL:142572686-142572697
chr11_MATERNAL:112001441-112001452	chr11_MATERNAL:112001441-112001452
chr2_MATERNAL:111633891-111633903	chr2_MATERNAL:111633891-111633903
chr8_MATERNAL:17891102-17891113	chr8_MATERNAL:17891102-17891113
chr5_MATERNAL:99612935-99612946	chr5_MATERNAL:99612935-99612946
chr11_MATERNAL:116630734-116630745	chr11_MATERNAL:116630734-116630745
chr9_MATERNAL:127606586-127606597	chr9_MATERNAL:127606586-127606597
chr19_MATERNAL:47409359-47409370	chr19_MATERNAL:47409359-47409370
chr11_MATERNAL:81795086-81795097	chr11_MATERNAL:81795086-81795097
chr16_MATERNAL:75276419-75276430	chr16_MATERNAL:75276419-75276430
chr1_MATERNAL:69393523-69393534	chr1_MATERNAL:69393523-69393534
chr18_MATERNAL:27492285-27492297	chr18_MATERNAL:27492285-27492297
chr1_MATERNAL:208040110-208040121	chr1_MATERNAL:208040110-208040121
chr19_MATERNAL:46040753-46040764	chr19_MATERNAL:46040753-46040764
chr13_MATERNAL:20045334-20045345	chr13_MATERNAL:20045334-20045345
chr14_MATERNAL:94502831-94502843	chr14_MATERNAL:94502831-94502843
chr11_MATERNAL:72234586-72234597	chr11_MATERNAL:72234586-72234597
chr2_MATERNAL:118816100-118816111	chr2_MATERNAL:118816100-118816111
chr9_MATERNAL:77108061-77108071	chr9_MATERNAL:77108061-77108071
chr10_MATERNAL:122064911-122064922	chr10_MATERNAL:122064911-122064922
chr4_MATERNAL:34010293-34010304	chr4_MATERNAL:34010293-34010304
chr5_MATERNAL:35148520-35148531	chr5_MATERNAL:35148520-35148531
chr17_MATERNAL:4631311-4631322	chr17_MATERNAL:4631311-4631322
chr7_MATERNAL:15422564-15422576	chr7_MATERNAL:15422564-15422576
chr6_MATERNAL:140503244-140503254	chr6_MATERNAL:140503244-140503254
chr11_MATERNAL:73519784-73519795	chr11_MATERNAL:73519784-73519795
chr2_MATERNAL:26074092-26074103	chr2_MATERNAL:26074092-26074103
chr12_MATERNAL:106550117-106550128	chr12_MATERNAL:106550117-106550128
chr10_MATERNAL:10848748-10848759	chr10_MATERNAL:10848748-10848759

chr2_MATERNAL:186490199-186490210	chr2_MATERNAL:186490199-186490210
chr7_MATERNAL:28791693-28791704	chr7_MATERNAL:28791693-28791704
chr14_MATERNAL:87506894-87506905	chr14_MATERNAL:87506894-87506905
chr2_MATERNAL:157705860-157705871	chr2_MATERNAL:157705860-157705871
chr12_MATERNAL:7673649-7673660	chr12_MATERNAL:7673649-7673660
chr16_MATERNAL:77617834-77617845	chr16_MATERNAL:77617834-77617845
chr1_MATERNAL:218681488-218681499	chr1_MATERNAL:218681488-218681499
chr18_MATERNAL:61035536-61035547	chr18_MATERNAL:61035536-61035547
chr4_MATERNAL:31046794-31046805	chr4_MATERNAL:31046794-31046805
chr12_MATERNAL:2467748-2467759	chr12_MATERNAL:2467748-2467759
chr7_MATERNAL:44903752-44903763	chr7_MATERNAL:44903752-44903763
chr4_MATERNAL:214844-214856	chr4_MATERNAL:214844-214856
chr3_MATERNAL:185437240-185437253	chr3_MATERNAL:185437240-185437253
chr1_MATERNAL:185563257-185563269	chr1_MATERNAL:185563257-185563269
chr2_MATERNAL:75737526-75737538	chr2_MATERNAL:75737526-75737538
chr12_MATERNAL:47023001-47023013	chr12_MATERNAL:47023001-47023013
chr12_MATERNAL:10797963-10797976	chr12_MATERNAL:10797963-10797976
chr9_MATERNAL:93764113-93764125	chr9_MATERNAL:93764113-93764125
chr12_MATERNAL:51908650-51908662	chr12_MATERNAL:51908650-51908662
chr2_MATERNAL:203963484-203963496	chr2_MATERNAL:203963484-203963496
chr14_MATERNAL:44403669-44403681	chr14_MATERNAL:44403669-44403681
chr1_MATERNAL:43953079-43953091	chr1_MATERNAL:43953079-43953091
chr7_MATERNAL:141946630-141946642	chr7_MATERNAL:141946630-141946642
chr10_MATERNAL:92926216-92926228	chr10_MATERNAL:92926216-92926228
chr11_MATERNAL:17004482-17004494	chr11_MATERNAL:17004482-17004494
chr14_MATERNAL:92504357-92504369	chr14_MATERNAL:92504357-92504369
chr1_MATERNAL:230734050-230734062	chr1_MATERNAL:230734050-230734062
chr3_MATERNAL:47949663-47949675	chr3_MATERNAL:47949663-47949675
chr3_MATERNAL:167799372-167799384	chr3_MATERNAL:167799372-167799384
chr20_MATERNAL:14979292-14979304	chr20_MATERNAL:14979292-14979304
chr2_MATERNAL:68500337-68500349	chr2_MATERNAL:68500337-68500349
chr11_MATERNAL:68313600-68313612	chr11_MATERNAL:68313600-68313612
chr1_MATERNAL:75353540-75353552	chr1_MATERNAL:75353540-75353552
chr9_MATERNAL:105696036-105696048	chr9_MATERNAL:105696036-105696048
chr7_MATERNAL:1866311-1866323	chr7_MATERNAL:1866311-1866323
chr1_MATERNAL:6505815-6505827	chr1_MATERNAL:6505815-6505827
chr9_MATERNAL:128210552-128210564	chr9_MATERNAL:128210552-128210564
chr13_MATERNAL:29442335-29442347	chr13_MATERNAL:29442335-29442347
chr5_MATERNAL:103801586-103801597	chr5_MATERNAL:103801586-103801597
chr9_MATERNAL:2828259-2828271	chr9_MATERNAL:2828259-2828271
chr12_MATERNAL:93681181-93681193	chr12_MATERNAL:93681181-93681193
chr9_MATERNAL:127297618-127297630	chr9_MATERNAL:127297618-127297630
chr19_MATERNAL:37628479-37628491	chr19_MATERNAL:37628479-37628491
chr3_MATERNAL:72805064-72805076	chr3_MATERNAL:72805064-72805076
chr10_MATERNAL:94634498-94634510	chr10_MATERNAL:94634498-94634510
chr6_MATERNAL:34226905-34226917	chr6_MATERNAL:34226905-34226917
chr13_MATERNAL:36084213-36084225	chr13_MATERNAL:36084213-36084225

chr13_MATERNAL:29185006-29185017	chr13_MATERNAL:29185006-29185017
chr9_MATERNAL:136506316-136506328	chr9_MATERNAL:136506316-136506328
chr16_MATERNAL:68136837-68136849	chr16_MATERNAL:68136837-68136849
chr15_MATERNAL:54415887-54415899	chr15_MATERNAL:54415887-54415899
chr6_MATERNAL:37630148-37630160	chr6_MATERNAL:37630148-37630160
chr10_MATERNAL:75193637-75193648	chr10_MATERNAL:75193637-75193648
chr11_MATERNAL:59894127-59894139	chr11_MATERNAL:59894127-59894139
chr1_MATERNAL:119007870-119007883	chr1_MATERNAL:119007870-119007883
chr11_MATERNAL:118449218-118449230	chr11_MATERNAL:118449218-118449230
chr9_MATERNAL:36815472-36815482	chr9_MATERNAL:36815472-36815482
chr15_MATERNAL:95274658-95274670	chr15_MATERNAL:95274658-95274670
chr18_MATERNAL:27022528-27022540	chr18_MATERNAL:27022528-27022540
chr1_MATERNAL:107152703-107152715	chr1_MATERNAL:107152703-107152715
chr18_MATERNAL:2694408-2694420	chr18_MATERNAL:2694408-2694420
chr11_MATERNAL:126461509-126461523	chr11_MATERNAL:126461509-126461523
chr4_MATERNAL:148505136-148505149	chr4_MATERNAL:148505136-148505149
chr4_MATERNAL:153970958-153970971	chr4_MATERNAL:153970958-153970971
chr1_MATERNAL:239302586-239302599	chr1_MATERNAL:239302586-239302599
chr10_MATERNAL:64148762-64148775	chr10_MATERNAL:64148762-64148775
chr12_MATERNAL:79904686-79904699	chr12_MATERNAL:79904686-79904699
chr17_MATERNAL:411984-411997	chr17_MATERNAL:411984-411997
chr9_MATERNAL:29171424-29171437	chr9_MATERNAL:29171424-29171437
chr5_MATERNAL:109690764-109690778	chr5_MATERNAL:109690764-109690778
chr7_MATERNAL:93964003-93964016	chr7_MATERNAL:93964003-93964016
chr7_MATERNAL:151404954-151404967	chr7_MATERNAL:151404954-151404967
chr12_MATERNAL:86030733-86030746	chr12_MATERNAL:86030733-86030746
chr4_MATERNAL:151805604-151805617	chr4_MATERNAL:151805604-151805617
chr9_MATERNAL:4695083-4695096	chr9_MATERNAL:4695083-4695096
chr17_MATERNAL:65236512-65236525	chr17_MATERNAL:65236512-65236525
chr4_MATERNAL:146909372-146909385	chr4_MATERNAL:146909372-146909385
chr19_MATERNAL:24027825-24027838	chr19_MATERNAL:24027825-24027838
chr20_MATERNAL:36478036-36478049	chr20_MATERNAL:36478036-36478049
chr1_MATERNAL:28459224-28459237	chr1_MATERNAL:28459224-28459237
chr2_MATERNAL:60457950-60457963	chr2_MATERNAL:60457950-60457963
chr11_MATERNAL:30448949-30448961	chr11_MATERNAL:30448949-30448961
chr9_MATERNAL:101732506-101732519	chr9_MATERNAL:101732506-101732519
chr8_MATERNAL:67765068-67765081	chr8_MATERNAL:67765068-67765081
chr21_MATERNAL:37905167-37905180	chr21_MATERNAL:37905167-37905180
chr4_MATERNAL:146691584-146691597	chr4_MATERNAL:146691584-146691597
chr7_MATERNAL:113742608-113742621	chr7_MATERNAL:113742608-113742621
chr12_MATERNAL:117972444-117972457	chr12_MATERNAL:117972444-117972457
chr2_MATERNAL:143951983-143951996	chr2_MATERNAL:143951983-143951996
chr19_MATERNAL:5482839-5482853	chr19_MATERNAL:5482839-5482853
chr1_MATERNAL:85408555-85408568	chr1_MATERNAL:85408555-85408568
chr16_MATERNAL:25066804-25066816	chr16_MATERNAL:25066804-25066816
chr13_MATERNAL:46076001-46076014	chr13_MATERNAL:46076001-46076014
chr1_MATERNAL:9783973-9783986	chr1_MATERNAL:9783973-9783986

chr15_MATERNAL:74828126-74828140	chr15_MATERNAL:74828126-74828140
chr3_MATERNAL:72952066-72952078	chr3_MATERNAL:72952066-72952078
chr8_MATERNAL:51359562-51359575	chr8_MATERNAL:51359562-51359575
chr11_MATERNAL:35497849-35497862	chr11_MATERNAL:35497849-35497862
chr6_MATERNAL:17431101-17431114	chr6_MATERNAL:17431101-17431114
chr12_MATERNAL:101055415-101055428	chr12_MATERNAL:101055415-101055428
chr6_MATERNAL:46421526-46421539	chr6_MATERNAL:46421526-46421539
chr4_MATERNAL:31240679-31240692	chr4_MATERNAL:31240679-31240692
chr7_MATERNAL:100194842-100194856	chr7_MATERNAL:100194842-100194856
chr17_MATERNAL:15869906-15869919	chr17_MATERNAL:15869906-15869919
chr14_MATERNAL:98231340-98231353	chr14_MATERNAL:98231340-98231353
chr13_MATERNAL:90869118-90869131	chr13_MATERNAL:90869118-90869131
chr5_MATERNAL:160044172-160044185	chr5_MATERNAL:160044172-160044185
chr1_MATERNAL:168903122-168903135	chr1_MATERNAL:168903122-168903135
chr8_MATERNAL:15120988-15121001	chr8_MATERNAL:15120988-15121001
chr19_MATERNAL:5645274-5645287	chr19_MATERNAL:5645274-5645287
chr15_MATERNAL:93818065-93818078	chr15_MATERNAL:93818065-93818078
chr7_MATERNAL:34167665-34167679	chr7_MATERNAL:34167665-34167679
chr6_MATERNAL:43432886-43432900	chr6_MATERNAL:43432886-43432900
chr12_MATERNAL:69107584-69107598	chr12_MATERNAL:69107584-69107598
chr4_MATERNAL:119644808-119644822	chr4_MATERNAL:119644808-119644822
chr17_MATERNAL:64174496-64174510	chr17_MATERNAL:64174496-64174510
chr5_MATERNAL:148917009-148917023	chr5_MATERNAL:148917009-148917023
chr11_MATERNAL:110989595-110989609	chr11_MATERNAL:110989595-110989609
chr13_MATERNAL:70360547-70360561	chr13_MATERNAL:70360547-70360561
chr6_MATERNAL:90880416-90880430	chr6_MATERNAL:90880416-90880430
chr1_MATERNAL:146514783-146514795	chr1_MATERNAL:146514783-146514795
chr11_MATERNAL:62647624-62647638	chr11_MATERNAL:62647624-62647638
chr12_MATERNAL:30818977-30818991	chr12_MATERNAL:30818977-30818991
chr2_MATERNAL:135847019-135847033	chr2_MATERNAL:135847019-135847033
chr1_MATERNAL:219113131-219113145	chr1_MATERNAL:219113131-219113145
chr17_MATERNAL:73506639-73506652	chr17_MATERNAL:73506639-73506652
chr5_MATERNAL:33688307-33688321	chr5_MATERNAL:33688307-33688321
chr22_MATERNAL:26836303-26836317	chr22_MATERNAL:26836303-26836317
chr11_MATERNAL:62118809-62118823	chr11_MATERNAL:62118809-62118823
chr7_MATERNAL:12750849-12750863	chr7_MATERNAL:12750849-12750863
chr4_MATERNAL:30023128-30023142	chr4_MATERNAL:30023128-30023142
chr10_MATERNAL:85086821-85086835	chr10_MATERNAL:85086821-85086835
chr19_MATERNAL:30370931-30370945	chr19_MATERNAL:30370931-30370945
chr3_MATERNAL:89746457-89746471	chr3_MATERNAL:89746457-89746471
chr1_MATERNAL:20918088-20918102	chr1_MATERNAL:20918088-20918102
chr18_MATERNAL:74961638-74961652	chr18_MATERNAL:74961638-74961652
chr1_MATERNAL:32575068-32575082	chr1_MATERNAL:32575068-32575082
chr2_MATERNAL:15076103-15076117	chr2_MATERNAL:15076103-15076117
chr18_MATERNAL:14314185-14314199	chr18_MATERNAL:14314185-14314199
chr5_MATERNAL:40476191-40476205	chr5_MATERNAL:40476191-40476205
chr1_MATERNAL:104805-104819	chr1_MATERNAL:104805-104819

chr9_MATERNAL:89607242-89607256	chr9_MATERNAL:89607242-89607256
chr11_MATERNAL:83761525-83761539	chr11_MATERNAL:83761525-83761539
chr1_MATERNAL:81552733-81552746	chr1_MATERNAL:81552733-81552746
chr16_MATERNAL:5570926-5570939	chr16_MATERNAL:5570926-5570939
chr16_MATERNAL:29804407-29804421	chr16_MATERNAL:29804407-29804421
chr19_MATERNAL:3144824-3144838	chr19_MATERNAL:3144824-3144838
chr10_MATERNAL:86638709-86638723	chr10_MATERNAL:86638709-86638723
chr15_MATERNAL:55087926-55087940	chr15_MATERNAL:55087926-55087940
chr6_MATERNAL:136655347-136655361	chr6_MATERNAL:136655347-136655361
chr4_MATERNAL:105964507-105964521	chr4_MATERNAL:105964507-105964521
chr1_MATERNAL:194910641-194910655	chr1_MATERNAL:194910641-194910655
chr1_MATERNAL:238242275-238242289	chr1_MATERNAL:238242275-238242289
chr7_MATERNAL:132996594-132996608	chr7_MATERNAL:132996594-132996608
chr17_MATERNAL:19447369-19447383	chr17_MATERNAL:19447369-19447383
chr15_MATERNAL:48835639-48835653	chr15_MATERNAL:48835639-48835653
chr15_MATERNAL:43590033-43590047	chr15_MATERNAL:43590033-43590047
chr8_MATERNAL:2955196-2955210	chr8_MATERNAL:2955196-2955210
chr10_MATERNAL:83214519-83214533	chr10_MATERNAL:83214519-83214533
chr1_MATERNAL:61582504-61582518	chr1_MATERNAL:61582504-61582518
chr1_MATERNAL:114533250-114533264	chr1_MATERNAL:114533250-114533264
hg38 coordinates	hg002 coordinates
chr10:106929774-106929797	chr10_MATERNAL:108976978-108977001
chr18:80195549-80195572	chr18_MATERNAL:78839234-78839257
chr14:72548349-72548372	chr14_MATERNAL:74535531-74535554
chr2:42452221-42452244	chr2_MATERNAL:42467513-42467544
chr4:59440189-59440212	chr4_MATERNAL:61085242-61085265
chr2:87537409-87537432	chr2_MATERNAL:87564284-87564307
chr5:31585717-31585740	chr5_MATERNAL:31710982-31711005
chr11:80480948-80480971	chr11_MATERNAL:80541449-80541472
chr3:74381064-74381087	chr3_MATERNAL:74344781-74344804
chr7:133089301-133089324	chr7_MATERNAL:134635431-134635454
chr3:120971639-120971662	chr3_MATERNAL:123486289-123486312
chr9:118145068-118145091	chr9_MATERNAL:123458449-123458472
chr6:96301416-96301439	chr6_MATERNAL:100168589-100168612
chr15:32963924-32963947	chr15_MATERNAL:31884340-31884363
chr4:31223873-31223896	chr4_MATERNAL:31382915-31382938
chr4:20283121-20283144	chr4_MATERNAL:20438499-20438522
chr7:147618709-147618732	chr7_MATERNAL:149184712-149184735
chr9:98422497-98422520	chr9_MATERNAL:103709794-103709817
chr17:48808375-48808398	chr17_MATERNAL:49302378-49302401
chr12:84956999-84957022	chr12_MATERNAL:85192161-85192184
chr9:30121545-30121568	chr9_MATERNAL:30139720-30139743
chr3:181563375-181563398	chr3_MATERNAL:184022094-184022117
chr11:97004629-97004652	chr11_MATERNAL:97119309-97119332
chr11:70449729-70449752	chr11_MATERNAL:70594546-70594569
chr3:143287102-143287125	chr3_MATERNAL:145828050-145828073
chr1:144907139-144907162	chr1_MATERNAL:139897861-139897884

chr10:25696681-25696704	chr10_MATERNAL:25707361-25707384
chr4:81326649-81326672	chr4_MATERNAL:82737830-82737853
chr18:40167266-40167289	chr18_MATERNAL:38918047-38918070
chr10:46188712-46188735	chr10_MATERNAL:47518939-47518962
chr18:46564094-46564117	chr18_MATERNAL:45310137-45310160
chr13:31916334-31916357	chr13_MATERNAL:30866941-30866972
chr2:116891886-116891909	chr2_MATERNAL:116750593-116750616
chr6:43289905-43289928	chr6_MATERNAL:43239714-43239737
chr4:140067201-140067224	chr4_MATERNAL:141456455-141456478
chr1:83196119-83196142	chr1_MATERNAL:83356008-83356031
chr2:19382163-19382186	chr2_MATERNAL:19402830-19402853
chr13:27346182-27346205	chr13_MATERNAL:26301609-26301632
chr2:15416086-15416109	chr2_MATERNAL:15435840-15435863
chr2:14552872-14552895	chr2_MATERNAL:14572420-14572443
chr10:60763099-60763122	chr10_MATERNAL:62724760-62724783
chr2:33365627-33365650	chr2_MATERNAL:33407180-33407203
chr3:14995669-14995692	chr3_MATERNAL:14994967-14994990
chr10:14868502-14868525	chr10_MATERNAL:14870674-14870697
chr14:42299029-42299052	chr14_MATERNAL:44263093-44263116
chr5:123409993-123410016	chr5_MATERNAL:125159240-125159263
chr16:18314135-18314158	chr16_MATERNAL:18519043-18519066
chr3:48926243-48926266	chr3_MATERNAL:48892700-48892723
chr16:23409065-23409088	chr16_MATERNAL:23885246-23885269
chr8:126158238-126158261	chr8_MATERNAL:127765409-127765432
chr1:221055973-221055996	chr1_MATERNAL:215930994-215931017
chr2:200252720-200252743	chr2_MATERNAL:200146472-200146495
chr6:141143177-141143200	chr6_MATERNAL:145008741-145008764
chr8:3315438-3315461	chr8_MATERNAL:3260125-3260156
chr18:60846307-60846330	chr18_MATERNAL:59655410-59655433
chr10:113225105-113225128	chr10_MATERNAL:115269272-115269295
chr3:129764734-129764757	chr3_MATERNAL:132304593-132304624
chr2:103296917-103296940	chr2_MATERNAL:103187323-103187346
chr8:58721221-58721244	chr8_MATERNAL:59542099-59542122
chr8:76808911-76808934	chr8_MATERNAL:77637275-77637298
chr1:234226962-234226985	chr1_MATERNAL:229211135-229211158
chr13:109305921-109305944	chr13_MATERNAL:108292031-108292054
chr2:165128427-165128450	chr2_MATERNAL:165006516-165006539
chr1:157054656-157054679	chr1_MATERNAL:151890932-151890955
chr6:157893609-157893632	chr6_MATERNAL:161822146-161822169
chr12:129585816-129585839	chr12_MATERNAL:129883556-129883579
chr21:28788280-28788303	chr21_MATERNAL:29362768-29362791
chr22:35709368-35709391	chr22_MATERNAL:38247261-38247284
chr3:112233027-112233050	chr3_MATERNAL:114749834-114749865
chr13:84388047-84388070	chr13_MATERNAL:83377440-83377463
chr1:248356779-248356802	chr1_MATERNAL:243376489-243376512
chr17:4891201-4891224	chr17_MATERNAL:4786811-4786834
chr7:11976855-11976878	chr7_MATERNAL:12091131-12091154

chr18:46440274-46440297	chr18_MATERNAL:45186290-45186313
chr7:94340982-94341005	chr7_MATERNAL:95611277-95611300
chr15:80957600-80957623	chr15_MATERNAL:79919966-79919989
chr12:62717452-62717475	chr12_MATERNAL:62929686-62929709
chr16:55639819-55639842	chr16_MATERNAL:55729006-55729029
chr4:84156024-84156047	chr4_MATERNAL:85565487-85565510
chr11:40376722-40376745	chr11_MATERNAL:40474979-40475002
chr2:235981451-235981474	chr2_MATERNAL:235881710-235881733
chr15:47787912-47787935	chr15_MATERNAL:46719249-46719272
chr8:26772860-26772883	chr8_MATERNAL:26718733-26718756
chr16:19798422-19798445	chr16_MATERNAL:19927083-19927106
chr4:93844776-93844799	chr4_MATERNAL:95234202-95234225
chr2:35709689-35709712	chr2_MATERNAL:35750641-35750664
chr2:32849874-32849897	chr2_MATERNAL:32894432-32894455
chr14:72302682-72302705	chr14_MATERNAL:74289848-74289871
chr3:109266791-109266814	chr3_MATERNAL:111773739-111773762
chr18:43108348-43108371	chr18_MATERNAL:41856604-41856627
chr6:135292110-135292133	chr6_MATERNAL:139157941-139157964
chr2:184458088-184458111	chr2_MATERNAL:184355965-184355988
chr8:135219709-135219732	chr8_MATERNAL:136819879-136819902
chr3:40321544-40321567	chr3_MATERNAL:40334957-40334980
chr2:124279225-124279248	chr2_MATERNAL:124155710-124155733
chr3:99647476-99647499	chr3_MATERNAL:102146613-102146636
chr13:45521578-45521601	chr13_MATERNAL:44474813-44474844
chr1:32075840-32075863	chr1_MATERNAL:32242491-32242514
chr14:37906739-37906762	chr14_MATERNAL:39865205-39865228
chr2:144197296-144197319	chr2_MATERNAL:144078136-144078159
chr10:74488375-74488406	chr10_MATERNAL:76458494-76458525
chr1:163667249-163667280	chr1_MATERNAL:158575003-158575034
chr20:4035679-4035710	chr20_MATERNAL:4049336-4049367
chr4:61966574-61966605	chr4_MATERNAL:63607513-63607544
chr17:27794715-27794746	chr17_MATERNAL:28140225-28140256
chr2:154798189-154798220	chr2_MATERNAL:154664580-154664611
chr6:118751589-118751620	chr6_MATERNAL:122626727-122626758
chr6:125784988-125785019	chr6_MATERNAL:129662007-129662038
chr5:97416354-97416385	chr5_MATERNAL:99162027-99162058
chr8:36077236-36077267	chr8_MATERNAL:36019081-36019112
chr9:2102133-2102164	chr9_MATERNAL:2102443-2102474
chr6:133168411-133168442	chr6_MATERNAL:137035125-137035156
chr16:33166046-33166077	chr16_MATERNAL:33672692-33672723
chr7:109431714-109431745	chr7_MATERNAL:110993409-110993440
chr18:5941081-5941112	chr18_MATERNAL:5941624-5941647
chr5:173674446-173674477	chr5_MATERNAL:175411535-175411566
chr10:122538584-122538615	chr10_MATERNAL:124588683-124588714
chr19:55840345-55840376	chr19_MATERNAL:58544997-58545028
chr2:57124579-57124610	chr2_MATERNAL:57128364-57128395
chr5:16923664-16923695	chr5_MATERNAL:16957343-16957374

chr16:2495003-2495034	chr16_MATERNAL:2520524-2520555
chr20:21469846-21469877	chr20_MATERNAL:21502639-21502670
chr2:217331345-217331376	chr2_MATERNAL:217237726-217237749
chr12:64788193-64788224	chr12_MATERNAL:65000024-65000055
chr11:131881478-131881509	chr11_MATERNAL:132030589-132030620
chr10:106910284-106910315	chr10_MATERNAL:108957501-108957532
chr6:154248307-154248338	chr6_MATERNAL:158131046-158131077
chr9:71687463-71687494	chr9_MATERNAL:76936136-76936175
chr2:211708613-211708644	chr2_MATERNAL:211610870-211610901
chr9:28632882-28632913	chr9_MATERNAL:28648290-28648321
chr17:15444715-15444746	chr17_MATERNAL:15347486-15347517
chr3:147035483-147035514	chr3_MATERNAL:149581441-149581472
chr6:83406972-83407003	chr6_MATERNAL:87339776-87339807
chr9:125156745-125156776	chr9_MATERNAL:130474093-130474124
chr18:74551362-74551393	chr18_MATERNAL:73403522-73403545
chr17:37177270-37177301	chr17_MATERNAL:37318485-37318516
chr5:85057600-85057631	chr5_MATERNAL:86781777-86781808
chr15:25276134-25276165	chr15_MATERNAL:24140329-24140360
chr13:76368699-76368730	chr13_MATERNAL:75346232-75346263
chr2:17134858-17134889	chr2_MATERNAL:17155098-17155121
chr5:95455371-95455402	chr5_MATERNAL:97199777-97199808
chr16:51560952-51560983	chr16_MATERNAL:51648795-51648826
chr3:8868171-8868202	chr3_MATERNAL:8858670-8858701
chr6:6007567-6007598	chr6_MATERNAL:5974840-5974871
chr2:162972202-162972233	chr2_MATERNAL:162850339-162850370
chr13:22781020-22781051	chr13_MATERNAL:21719816-21719847
chr11:124678517-124678548	chr11_MATERNAL:124815972-124816003
chr4:118434497-118434528	chr4_MATERNAL:119808313-119808344
chr3:24477086-24477117	chr3_MATERNAL:24482214-24482245
chr12:27611786-27611817	chr12_MATERNAL:27485337-27485368
chr10:127934025-127934056	chr10_MATERNAL:130003228-130003259
chr8:3602717-3602748	chr8_MATERNAL:3548236-3548267
chr2:85289222-85289253	chr2_MATERNAL:85309571-85309602
chr7:67544345-67544376	chr7_MATERNAL:68870542-68870581
chr2:143063997-143064028	chr2_MATERNAL:142945024-142945055
chr1:47738901-47738932	chr1_MATERNAL:47931617-47931648
chr6:139705783-139705814	chr6_MATERNAL:143571695-143571726
chr15:25845323-25845354	chr15_MATERNAL:24712636-24712667
chr4:33683511-33683542	chr4_MATERNAL:33822928-33822959
chr18:65652217-65652248	chr18_MATERNAL:64465454-64465485
chr5:20351161-20351192	chr5_MATERNAL:20448487-20448518
chr20:60134741-60134772	chr20_MATERNAL:61876292-61876323
chr7:153455281-153455312	chr7_MATERNAL:155012133-155012164
chr18:24488273-24488304	chr18_MATERNAL:23235271-23235302
chr7:80219028-80219059	chr7_MATERNAL:81488532-81488571
chr12:67675636-67675667	chr12_MATERNAL:67885871-67885902
chr1:186063035-186063066	chr1_MATERNAL:180957874-180957905

chr12:85825784-85825815	chr12_MATERNAL:86060912-86060943
chr3:190776186-190776217	chr3_MATERNAL:193239647-193239670
chr13:51472422-51472453	chr13_MATERNAL:50429335-50429366
chr6:95218945-95218976	chr6_MATERNAL:99083578-99083609
chr4:85211465-85211496	chr4_MATERNAL:86620939-86620970
chr4:136491454-136491485	chr4_MATERNAL:137882682-137882713
chr14:75315627-75315658	chr14_MATERNAL:77279067-77279098
chr3:54682162-54682193	chr3_MATERNAL:54652570-54652601
chr6:138100543-138100574	chr6_MATERNAL:141966569-141966600
chr13:39245005-39245036	chr13_MATERNAL:38188584-38188615
chr3:181406845-181406876	chr3_MATERNAL:183865597-183865628
chr18:73990016-73990047	chr18_MATERNAL:72839998-72840029
chr3:144586953-144586984	chr3_MATERNAL:147133303-147133334
chr12:2067450-2067481	chr12_MATERNAL:2059279-2059318
chr4:99467136-99467167	chr4_MATERNAL:100853086-100853117
chr9:123607489-123607520	chr9_MATERNAL:128924225-128924256
chr3:103548021-103548052	chr3_MATERNAL:106051992-106052023
chr1:34653990-34654021	chr1_MATERNAL:34825410-34825441
chr18:67018159-67018190	chr18_MATERNAL:65831251-65831282
chr13:76372506-76372537	chr13_MATERNAL:75350039-75350070
chr3:167633089-167633120	chr3_MATERNAL:170090182-170090213
chr4:13861881-13861912	chr4_MATERNAL:14015543-14015582
chr2:41214692-41214723	chr2_MATERNAL:41237344-41237375
chr4:113333299-113333330	chr4_MATERNAL:114713803-114713834
chr12:127210885-127210916	chr12_MATERNAL:127500873-127500904
chr3:59898443-59898474	chr3_MATERNAL:59869280-59869311
chr16:7189555-7189586	chr16_MATERNAL:7223100-7223131
chr11:109781827-109781858	chr11_MATERNAL:109898715-109898746
chr4:78578299-78578330	chr4_MATERNAL:79989742-79989773
chr9:3522557-3522588	chr9_MATERNAL:3525275-3525306
chr12:77938932-77938963	chr12_MATERNAL:78170205-78170236
chr1:182787928-182787959	chr1_MATERNAL:177688666-177688697
chr9:122487459-122487490	chr9_MATERNAL:127803965-127803996
chr7:35987818-35987857	chr7_MATERNAL:36093116-36093155
chr10:107942346-107942385	chr10_MATERNAL:109989598-109989637
chr3:75131893-75131932	chr3_MATERNAL:75095054-75095093
chr4:133413785-133413824	chr4_MATERNAL:134806174-134806213
chr18:38356444-38356483	chr18_MATERNAL:37106502-37106541
chr10:22683836-22683875	chr10_MATERNAL:22690953-22690992
chr8:25331231-25331270	chr8_MATERNAL:25274870-25274909
chr2:61930618-61930657	chr2_MATERNAL:61941689-61941728
chr2:62290569-62290608	chr2_MATERNAL:62301974-62302013
chr10:99168088-99168127	chr10_MATERNAL:101214739-101214770
chr16:83490010-83490049	chr16_MATERNAL:83614865-83614904
chr3:26770707-26770746	chr3_MATERNAL:26773784-26773823
chr4:81163864-81163903	chr4_MATERNAL:82575037-82575076
chr8:35864604-35864643	chr8_MATERNAL:35806445-35806484

chr5:23873273-23873312	chr5_MATERNAL:23992498-23992537
chr2:60373450-60373489	chr2_MATERNAL:60385578-60385617
chr12:66617086-66617125	chr12_MATERNAL:66827203-66827242
chr2:108551492-108551531	chr2_MATERNAL:108443044-108443083
chr7:119829561-119829600	chr7_MATERNAL:121369458-121369497
chr2:236137046-236137085	chr2_MATERNAL:236037320-236037359
chr1:242622265-242622304	chr1_MATERNAL:237628240-237628279
chr7:73601177-73601216	chr7_MATERNAL:74877999-74878038
chr6:46383169-46383208	chr6_MATERNAL:46339784-46339823
chr6:105906382-105906421	chr6_MATERNAL:109771875-109771914
chr17:44937978-44938017	chr17_MATERNAL:45617233-45617272
chr16:51541853-51541892	chr16_MATERNAL:51629696-51629735
chr1:198332970-198333009	chr1_MATERNAL:193227171-193227210
chr1:145371310-145371349	chr1_MATERNAL:140336618-140336657
chr12:49606487-49606526	chr12_MATERNAL:49792113-49792152
chr7:116080977-116081016	chr7_MATERNAL:117623865-117623904
chr12:92516138-92516177	chr12_MATERNAL:92761381-92761420
chr4:147490148-147490187	chr4_MATERNAL:148874649-148874688
chr14:87625597-87625636	chr14_MATERNAL:89590128-89590167
chr10:85057502-85057541	chr10_MATERNAL:87101289-87101328
chr1:235924919-235924958	chr1_MATERNAL:230916493-230916532
chr4:70590507-70590546	chr4_MATERNAL:72006231-72006270
chr6:83919761-83919800	chr6_MATERNAL:87852676-87852715
chr15:91857030-91857069	chr15_MATERNAL:90724506-90724545
chr7:67211151-67211190	chr7_MATERNAL:68537017-68537056
chr7:129662384-129662423	chr7_MATERNAL:131203592-131203631
chr4:118184603-118184642	chr4_MATERNAL:119558392-119558431
chr19:55307748-55307787	chr19_MATERNAL:58008394-58008433
chr1:30671823-30671862	chr1_MATERNAL:30832646-30832685
chr3:155412538-155412577	chr3_MATERNAL:157988508-157988547
chr12:74859484-74859523	chr12_MATERNAL:75087078-75087117
chr15:40083335-40083374	chr15_MATERNAL:39012456-39012495
chr3:167742819-167742858	chr3_MATERNAL:170199900-170199939
chr11:128719357-128719396	chr11_MATERNAL:128861060-128861099
chr11:134184811-134184850	chr11_MATERNAL:134339877-134339916
chr11:126689043-126689082	chr11_MATERNAL:126831549-126831588
chr6:119832065-119832104	chr6_MATERNAL:123707412-123707451
chr2:101560730-101560769	chr2_MATERNAL:101450767-101450806
chr16:47214922-47214961	chr16_MATERNAL:47301452-47301491
chr10:72403630-72403669	chr10_MATERNAL:74374040-74374079
chr4:104073733-104073772	chr4_MATERNAL:105456984-105457023
chr15:40050450-40050489	chr15_MATERNAL:38978003-38978042
chr11:109358340-109358379	chr11_MATERNAL:109475236-109475275
chr2:101971755-101971794	chr2_MATERNAL:101861918-101861957
chr3:34548087-34548126	chr3_MATERNAL:34551138-34551177
chr9:82062838-82062877	chr9_MATERNAL:87320809-87320848
chr16:66560799-66560838	chr16_MATERNAL:66656315-66656354

chr12:80640026-80640065	chr12_MATERNAL:80872110-80872149
chr6:97284484-97284523	chr6_MATERNAL:101153463-101153502
chr21:13425654-13425693	chr21_MATERNAL:13971616-13971647
chr9:16155330-16155369	chr9_MATERNAL:16173037-16173076
chr13:37127473-37127512	chr13_MATERNAL:36071394-36071433
chr3:41706850-41706889	chr3_MATERNAL:41720265-41720304
chr9:111849486-111849525	chr9_MATERNAL:117137236-117137275
chr2:36915233-36915272	chr2_MATERNAL:36935527-36935558
chr9:72672824-72672863	chr9_MATERNAL:77928753-77928792
chr8:105664322-105664361	chr8_MATERNAL:107266360-107266399
chr17:31063152-31063191	chr17_MATERNAL:31412268-31412307
chr1:159464271-159464310	chr1_MATERNAL:154298299-154298338
chr3:160713943-160713982	chr3_MATERNAL:163290061-163290100
chr6:27064585-27064624	chr6_MATERNAL:27015225-27015264
chr16:82029623-82029662	chr16_MATERNAL:82154158-82154197
chr14:50034492-50034531	chr14_MATERNAL:52008347-52008386
chr3:26504108-26504147	chr3_MATERNAL:26507157-26507196
chr13:56330839-56330878	chr13_MATERNAL:55293371-55293410
chr8:17128230-17128269	chr8_MATERNAL:17085204-17085243
chr1:187995908-187995947	chr1_MATERNAL:182891160-182891199
chr16:8682187-8682226	chr16_MATERNAL:8717012-8717051
chr2:142590593-142590632	chr2_MATERNAL:142471559-142471598
chr4:5625308-5625347	chr4_MATERNAL:5616527-5616566
chr3:140807810-140807849	chr3_MATERNAL:143348512-143348551
chr7:103185325-103185364	chr7_MATERNAL:104736066-104736097
chr7:149489234-149489273	chr7_MATERNAL:151053565-151053604
chr1:194947174-194947213	chr1_MATERNAL:189843268-189843307
chr10:49120534-49120573	chr10_MATERNAL:51082033-51082072
chr5:174199939-174199978	chr5_MATERNAL:175937907-175937946
chr6:111648918-111648957	chr6_MATERNAL:115520230-115520269
chr1:111784104-111784143	chr1_MATERNAL:111943465-111943504
chr10:95479958-95479997	chr10_MATERNAL:97524411-97524450
chr15:35491549-35491588	chr15_MATERNAL:34419935-34419974
chr2:162288918-162288957	chr2_MATERNAL:162166594-162166633
chr7:82984409-82984448	chr7_MATERNAL:84254611-84254650
chr1:168398964-168399003	chr1_MATERNAL:163312752-163312791
chr5:64796003-64796042	chr5_MATERNAL:66469302-66469341
chr1:95614001-95614040	chr1_MATERNAL:95774519-95774558
chr7:99646798-99646837	chr7_MATERNAL:100914588-100914627

repeat units	repeat number	*Figure 8
T	10	
A	10	
A	10	
A	10	
T	10	
T	10	
T	10	
T	10	
T	10	
T	10	
T	10	
T	10	
A	10	
A	10	
A	10	
T	10	
T	10	
A	10	
A	10	
C	10	
T	10	
A	10	
T	10	
T	10	
T	10	
T	10	
A	10	
T	10	
T	10	
A	10	
T	10	
A	10	
T	10	
A	10	
T	10	
T	10	
T	10	
A	10	
A	10	
T	10	
A	10	
T	10	
A	10	
T	10	
T	10	

A	10
A	10
A	10
A	10
A	10
A	11
T	11
T	11
T	11
T	11
A	11
A	11
A	11
A	11
A	11
A	11
A	11
A	11
T	11
A	11
T	11
A	11
A	11
T	11
A	11
T	11
A	11
T	11
A	11
T	11
T	11
T	11
T	11
A	11
T	11
A	11
A	11
T	11
T	11
T	11
A	11
A	11
T	11
T	11
T	11
C	11
A	11

A	11
T	11
A	11
A	11
T	11
T	11
A	11
T	11
A	12
A	12
A	12
T	12
T	12
T	12
T	12
A	12
A	12
A	12
A	12
A	12
A	12
T	12
T	12
A	12
A	12
A	12
T	12
T	12
T	12
A	12
T	12
A	12
A	12
A	12
A	12
A	12
T	12
A	12
T	12
T	12
A	12
A	12
T	12
T	12
T	12
T	12
A	12

T	12
G	12
A	12
T	12
T	12
A	12
T	12
T	12
T	12
T	12
T	12
A	13
A	13
A	13
A	13
T	13
A	13
A	13
A	13
T	13
T	13
A	13
T	13
A	13
A	13
T	13
A	13
A	13
A	13
A	13
T	13
T	13
T	13
T	13
A	13
T	13
A	13
T	13
A	13
T	13
A	13
T	13
A	13
A	13
A	13
A	13
T	13

A	13
T	13
T	13
T	13
A	13
T	13
A	13
A	13
A	13
T	13
A	13
T	13
T	13
T	13
T	14
A	14
T	14
T	14
A	14
A	14
T	14
T	14
T	14
A	14
T	14
T	14
T	14
T	14
T	14
T	14
A	14
T	14
A	14
A	14
A	14
T	14
T	14
T	14
A	14
A	14
A	14
T	14
A	14
T	14
T	14
T	14
A	14

A	14
A	14
A	14
A	14
T	14
A	14
A	14
T	14
T	14
T	14
A	14
A	14
A	14
A	14
T	14
A	14
T	14
T	15
A	15
T	15
A	15
A	15
A	15
A	15
A	15
A	15
A	15
A	15
A	15
T	15
A	15
A	15
T	15
A	15
A	15
A	15
A	15
T	15
A	15
T	15
A	15
T	15
T	15
T	15
T	15
T	15
T	15

T	15	
A	15	
T	15	
T	15	
T	15	
T	15	
T	15	
T	15	
T	15	
A	15	
T	15	
A	15	
T	15	
T	15	
A	15	
T	15	
T	15	
T	15	
A	15	
T	15	
repeat units	repeat number	*Supplementary Figure 12
CA	12	
AT	12	
GT	12	
AT	12	
TG	12	
TG	12	
GT	12	
AC	12	
TC	12	
TG	12	
AC	12	
TG	12	
TC	12	
CA	12	
AC	12	
CA	12	
AC	12	
TG	12	
TG	12	
TG	12	
CA	12	
TC	12	
CA	12	
TG	12	
TG	12	
GT	12	

TC	12
TG	12
TC	12
TC	12
GT	12
TG	12
GT	12
TG	12
GT	12
GT	12
TG	12
AC	12
AG	12
GT	12
AC	12
GT	12
GT	12
CA	12
TG	12
CA	12
CA	12
GT	12
GT	12
CA	12
TG	12
AC	12
GT	12
GT	12
AT	12
AC	12
TG	12
CA	12
AC	12
TC	12
CA	12
TG	12
GA	12
AG	12
TG	12
TG	12
AC	12
CA	12
AC	12
AC	12
AC	12
CA	12
AC	12

GA	12
AC	12
AC	12
AG	12
TC	12
TG	12
TG	12
AC	12
GT	12
CA	12
AT	12
GT	12
TG	12
GT	12
AC	12
AT	12
GT	12
AC	12
AC	12
TG	12
TG	12
GT	12
TC	12
CA	12
GT	12
AC	12
TG	12
GT	16
GT	16
GT	16
GT	16
CA	16
CA	16
TG	16
AC	16
AC	16
GT	16
GT	16
CA	16
AC	16
TG	16
CA	16
TG	16
CA	16
TG	16
AC	16
AC	16

CA	16
TG	16
AC	16
GT	16
AC	16
TG	16
GT	16
TG	16
TC	16
AG	16
AG	16
AC	16
AC	16
CT	16
AC	16
GT	16
CA	16
GT	16
TG	16
AT	16
AC	16
AC	16
AC	16
CA	16
AC	16
TG	16
TG	16
AC	16
GT	16
CT	16
CA	16
AC	16
GT	16
TG	16
GT	16
AC	16
GT	16
TG	16
GT	16
AC	16
TG	16
TG	16
AC	16
AC	16
TG	16
TG	16
TG	16

AC	16
TG	16
TG	16
TG	16
TG	16
TG	16
TG	16
CA	16
TG	16
TC	16
AT	16
AC	16
AC	16
GT	16
TG	16
AC	16
GT	16
CA	16
TG	16
TC	16
GT	16
AC	16
GT	16
TG	16
AC	16
TG	16
AC	16
CA	16
GA	16
GT	16
GT	16
CA	16
AC	16
AC	20
AC	20
AC	20
TG	20
TC	20
CA	20
AC	20
TG	20
AC	20
AC	20
CA	20
CA	20
AC	20
TA	20

TG	20
GT	20
TG	20
CA	20
GT	20
CA	20
TG	20
GT	20
GT	20
CA	20
AC	20
GT	20
TG	20
AC	20
AC	20
GT	20
TG	20
CA	20
AC	20
GT	20
AT	20
CA	20
TG	20
GT	20
TG	20
GT	20
AC	20
AC	20
AC	20
AC	20
AT	20
GT	20
TG	20
GT	20
AC	20
AG	20
AC	20
AC	20
AC	20
TG	20
AC	20
TG	20
TG	20
GT	20
TG	20
AC	20
AC	20

GT	20
CA	20
CA	20
GT	20
AC	20
TG	20
AC	20
AT	20
AC	20
TG	20
TC	20
TG	20
GT	20
TG	20
AC	20
AC	20
GT	20
CA	20
AC	20
CA	20
AC	20
AC	20
AC	20
TG	20
TG	20
CA	20
GT	20
AC	20
TG	20
AC	20
CA	20
CT	20
AC	20
CA	20
TG	20
GT	20
GT	20
GT	20
AC	20

Click here to access/download;Figure;Main figures raw.pptx

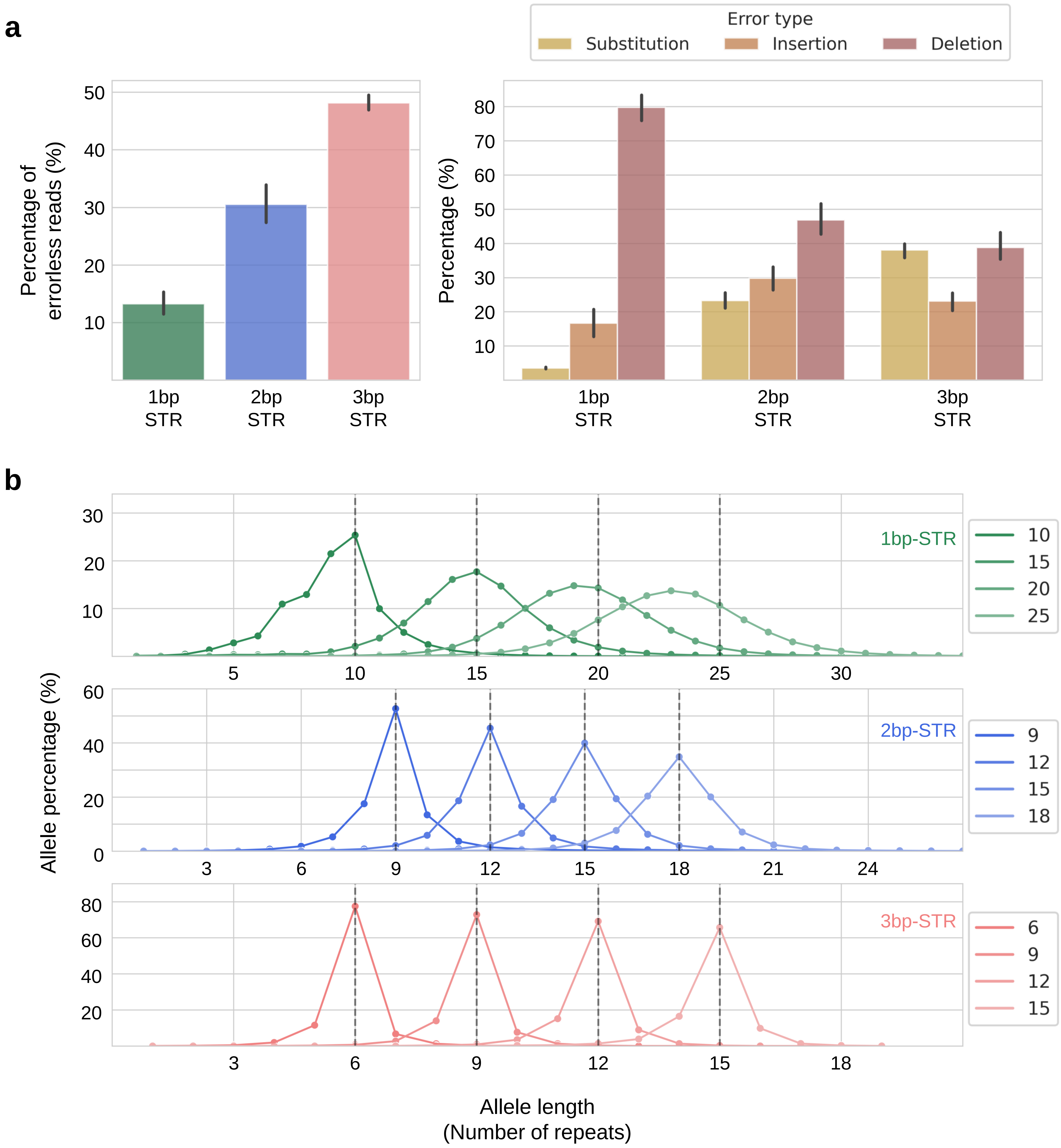

Figure 1. Distribution of sequencing error in STR regions.
(a) Percentage of errorless reads in 1bp-, 2bp- and 3bp-repeat STR (left) and distribution of sequencing error types (right). 1bp-repeats with 10~30 repeats, 2bp-repeats with 7~24 repeats, 3bp-repeats with 5~15 repeats were subjected to analysis. **(b)** STR allele size histogram of various lengths of 1bp-, 2bp- and 3bp-repeat STR.

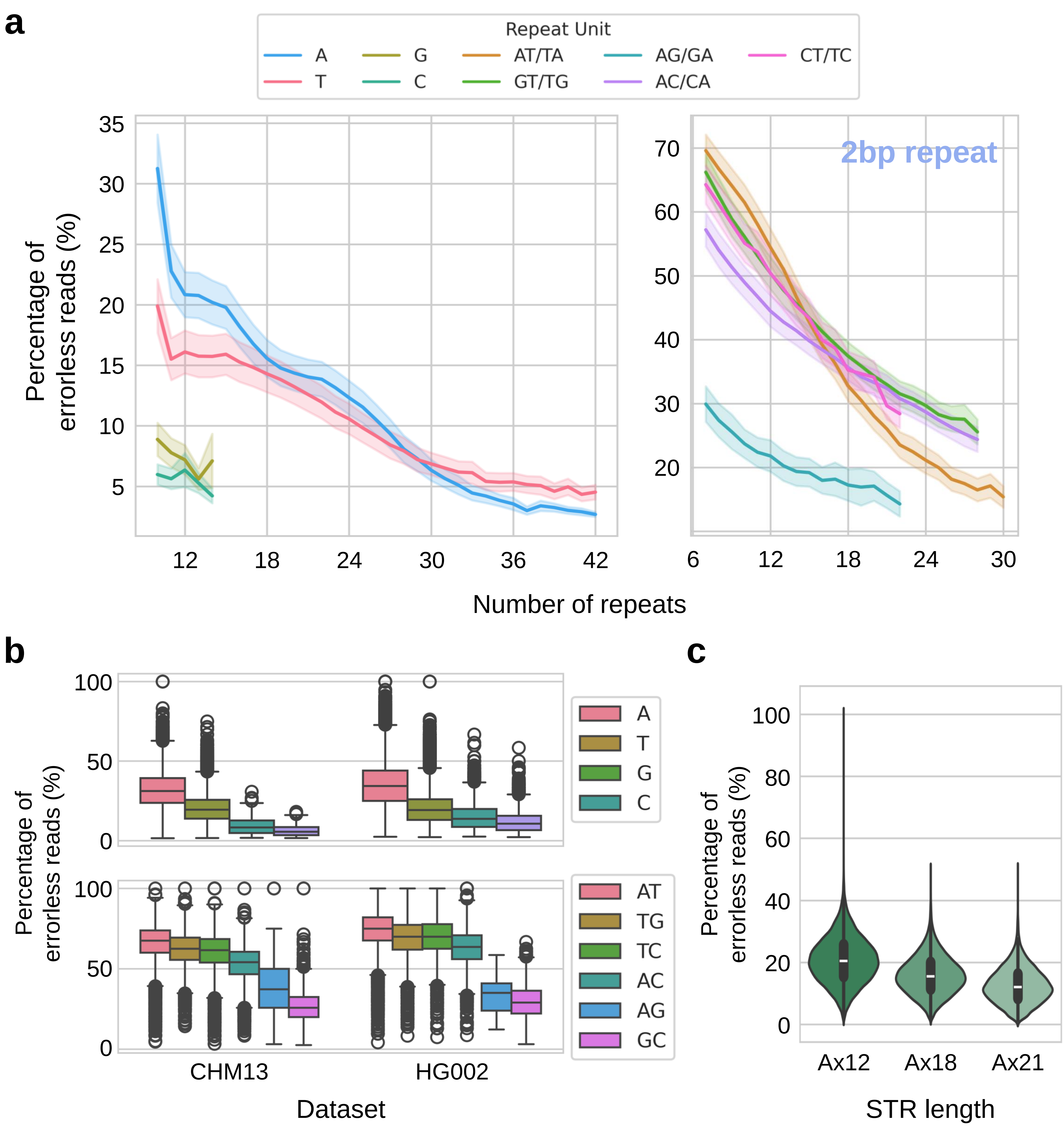

Figure 2. Sequencing accuracy of STR based on its repeat unit
(a) Sequencing accuracy (assessed by calculating the percentage of errorless reads) of varying lengths of 1bp-repeat STR (left) and 2bp-repeat STR (right) based on its repeat unit. **(b)** Sequencing accuracy of various types of STR compared between two different datasets. **(c)** Distribution of sequencing accuracy of Ax12, Ax18 and Ax21 STR, demonstrating the extreme variability of sequencing accuracy among identical type of STR.

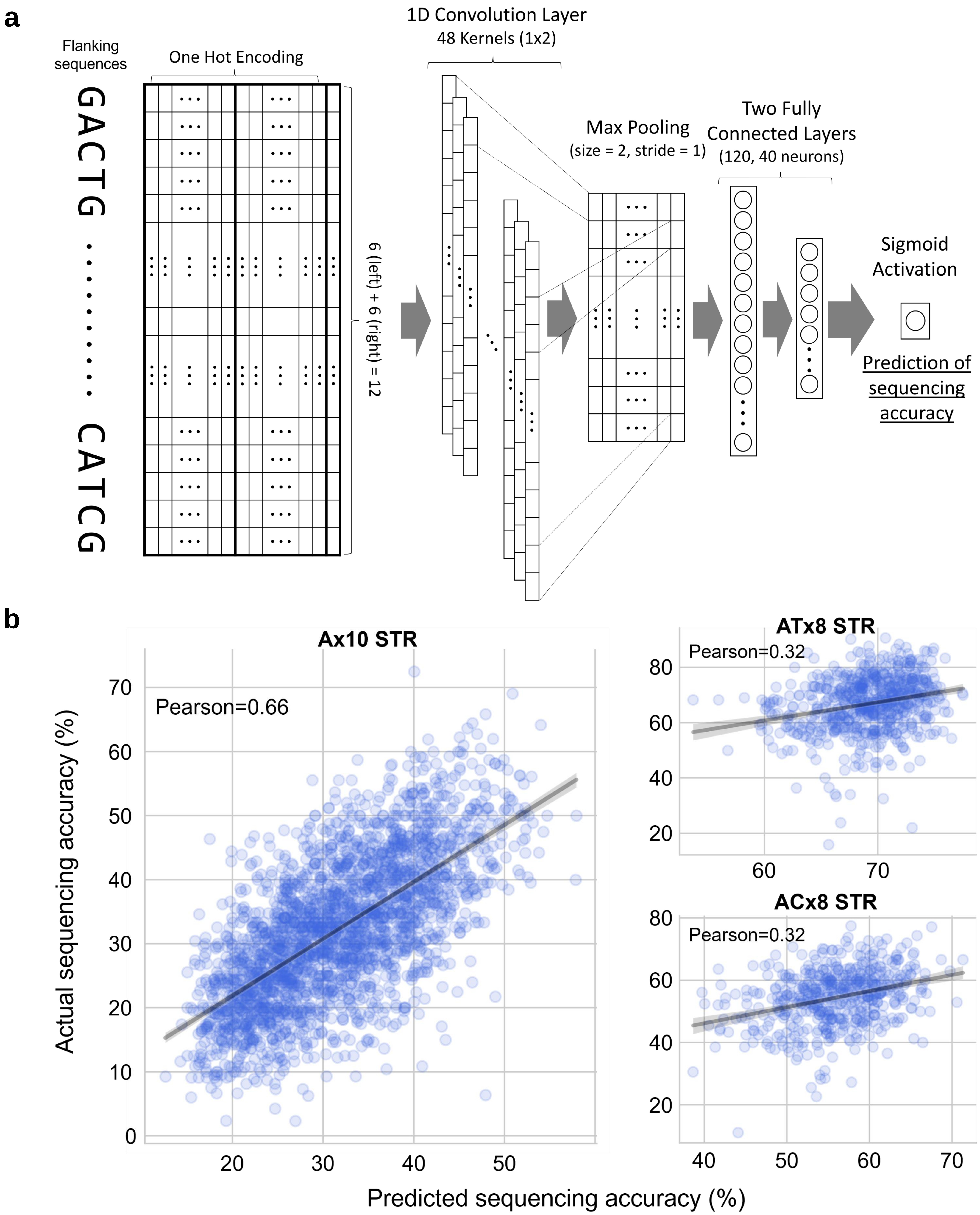

Figure 3. CNN-based machine learning prediction of STR sequencing accuracy using flanking sequences **(a)** Illustration of CNN-based machine learning prediction workflow. **(b)** Prediction results of Ax10 (left), ATx8 (upper right), and ACx8 (lower right) sequencing accuracy.

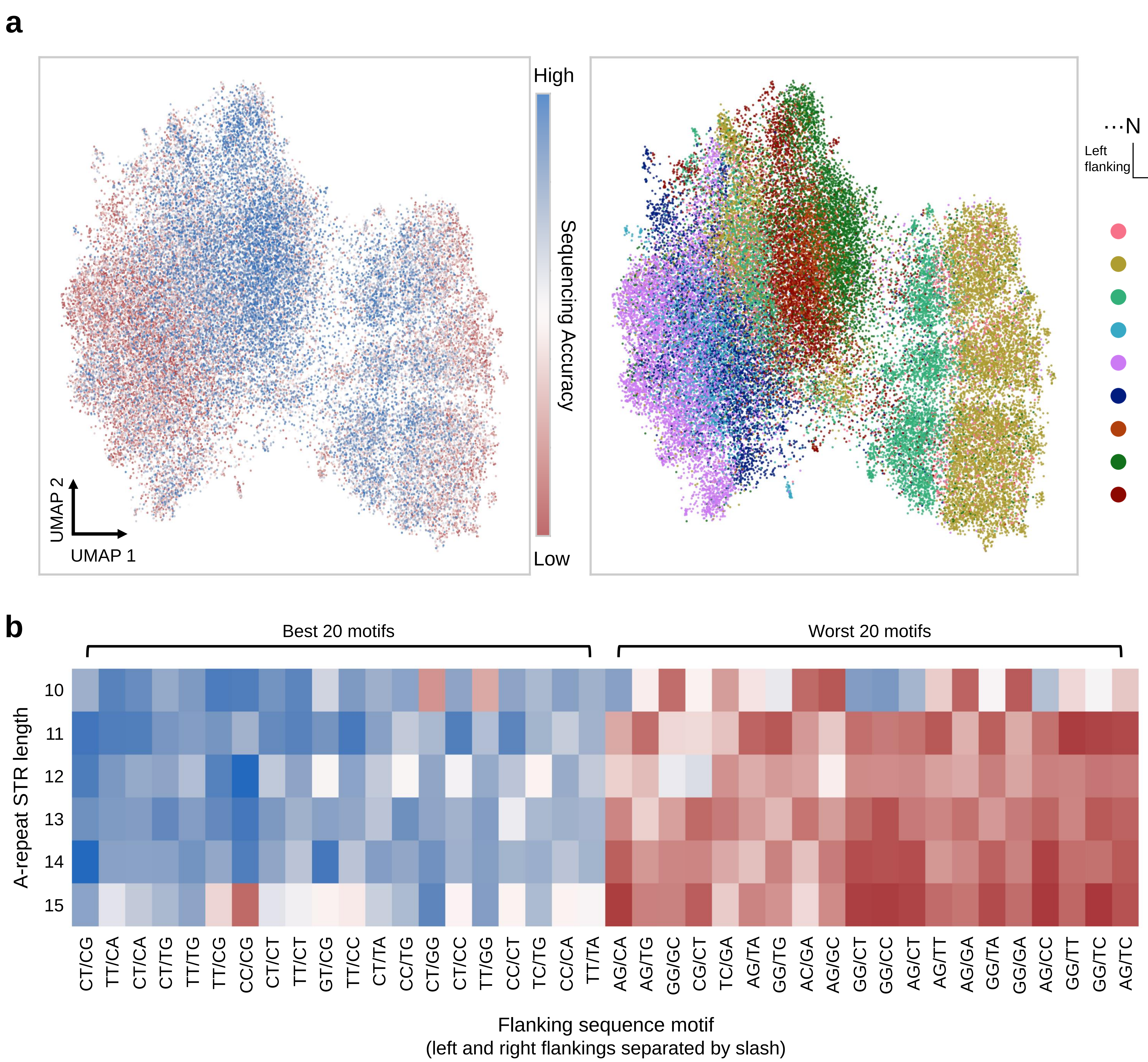

Figure 4. Identification of motifs associated with good/bad sequencing accuracy of A-repeat STR
(a) UMAP projection of flanking sequences of Ax10-Ax15 repeat STR whose sequencing accuracy were well predicted by CNN machine learning model. Each dot represents an A-repeat STR locus, colored by its sequencing accuracy (left) and by its most adjacent flanking nucleotide (right). **(b)** Flanking sequences of (2 nucleotides in each direction, 4 nucleotides total) A-repeat STR associated with good and bad sequencing accuracy. 2 nucleotides in each direction are separated by slash (e.g., CT/CG motif indicates CT-$(A)_n$-CG).

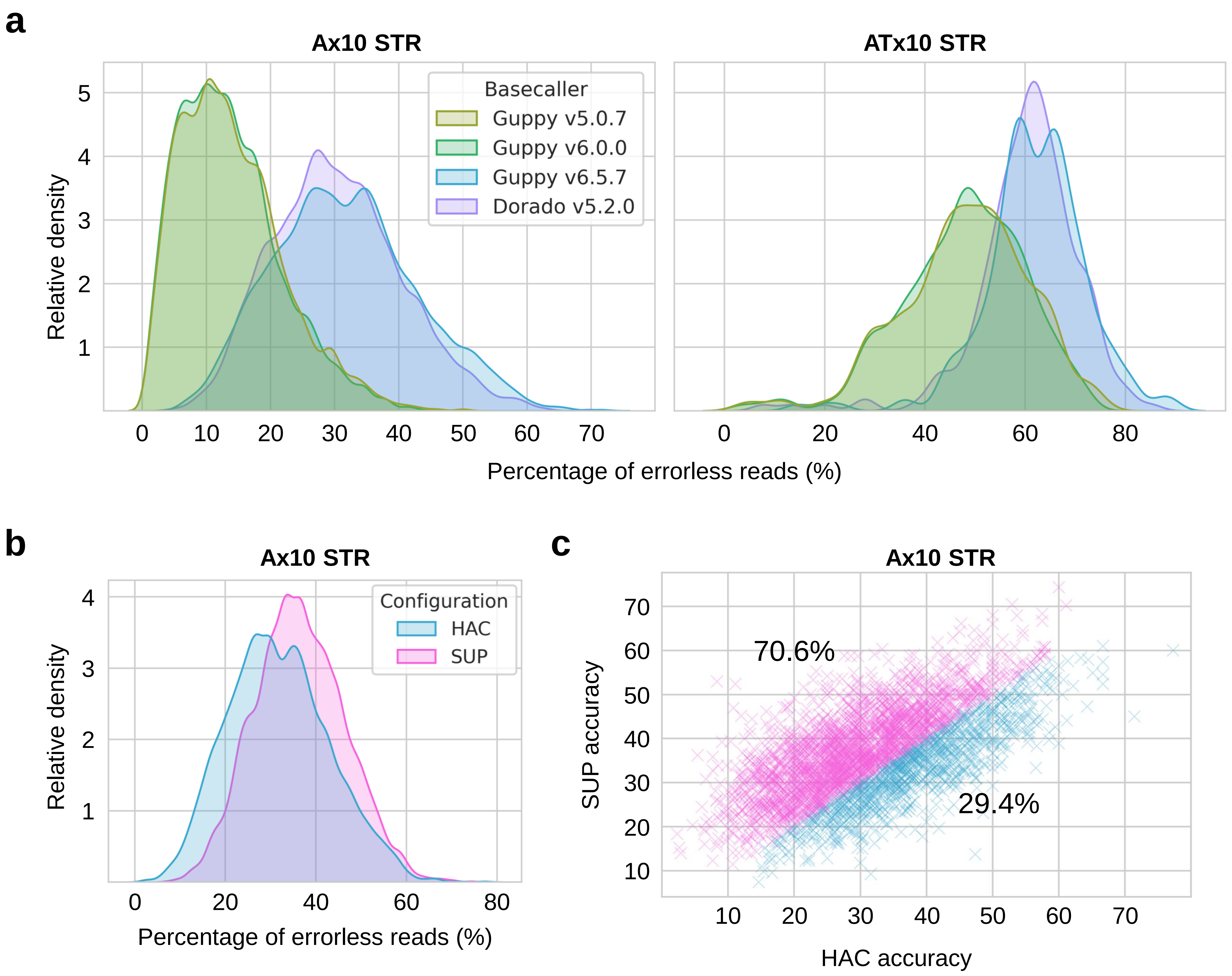

Figure 5. Influence of basecaller on STR sequencing accuracy
(a) Comparison of 4 ONT basecallers in basecalling Ax10 STR (left) and ATx10 STR (right), visualized with kernel density estimate plots. **(b)** Comparison of HAC model and SUP model in basecalling Ax10 STR, visualized with kernel density estimate plots. Both model is from Guppy v6.5.7. **(c)** Scatter plot showing the sequencing results of Ax10 STR loci obtained by HAC basecaller model (x-axis) and SUP basecaller model (y-axis), where each cross represents a Ax10 STR locus. Loci that exhibited better sequencing accuracy with either SUP basecaller model (70.6%) or HAC basecaller model (29.4%) were marked with different colors.

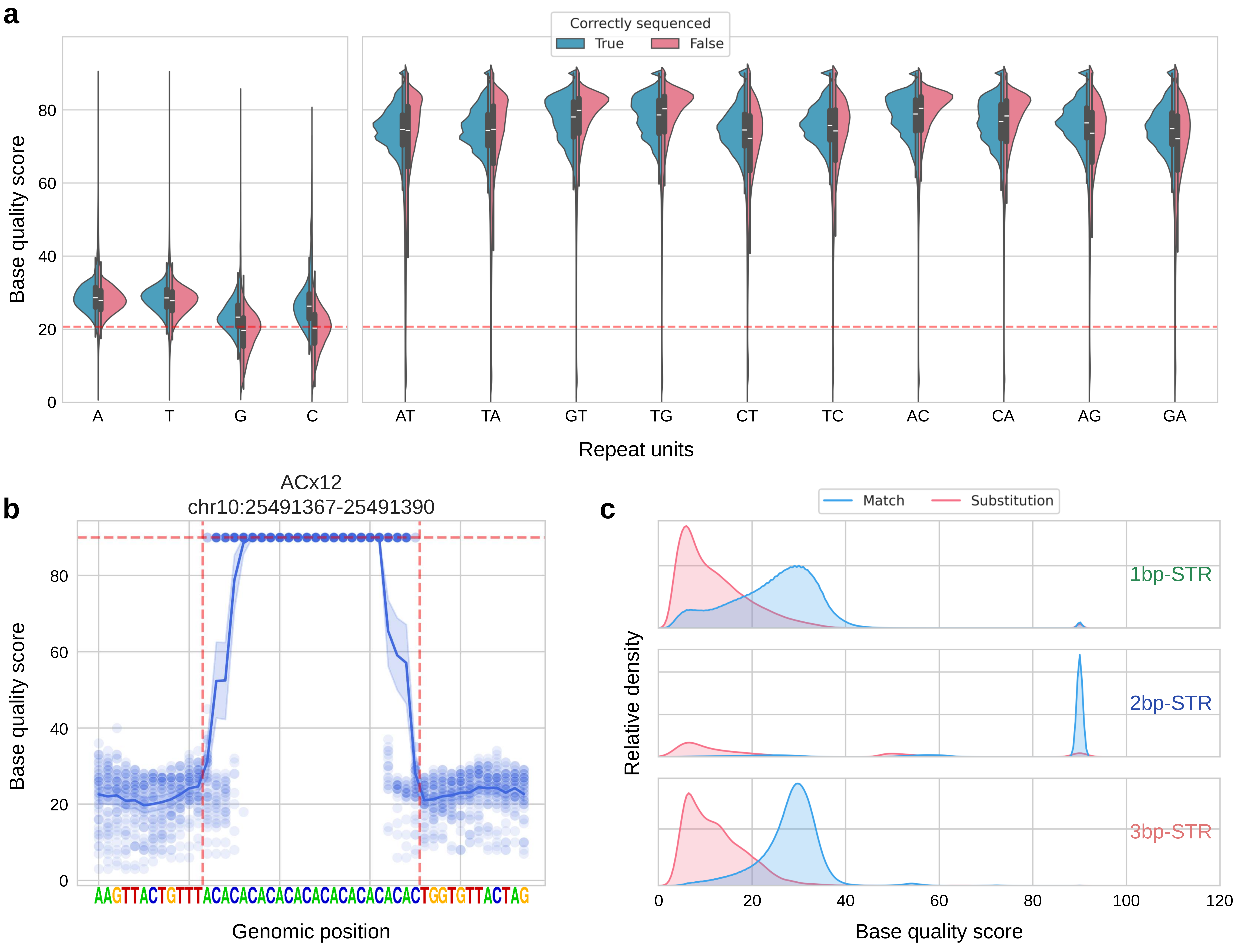

Figure 6. Base quality score of STR regions

(a) Base quality score comparison between correctly sequenced reads against reads that contained sequencing error, across various STR types. The red dashed horizontal line indicates the estimated base quality average across the entire CHM13 dataset (20.7). **(b)** Base quality score distribution in an ACx12 STR locus (chr10:25491367-25491390, T2T-CHM13 v2.0). Each dot represents the base quality of the given genomic position in a single read. The line represents the average base quality of each genomic position. **(c)** Phred quality score comparison of correctly sequenced bases versus substitution error bases, visualized with kernel density estimate plots.

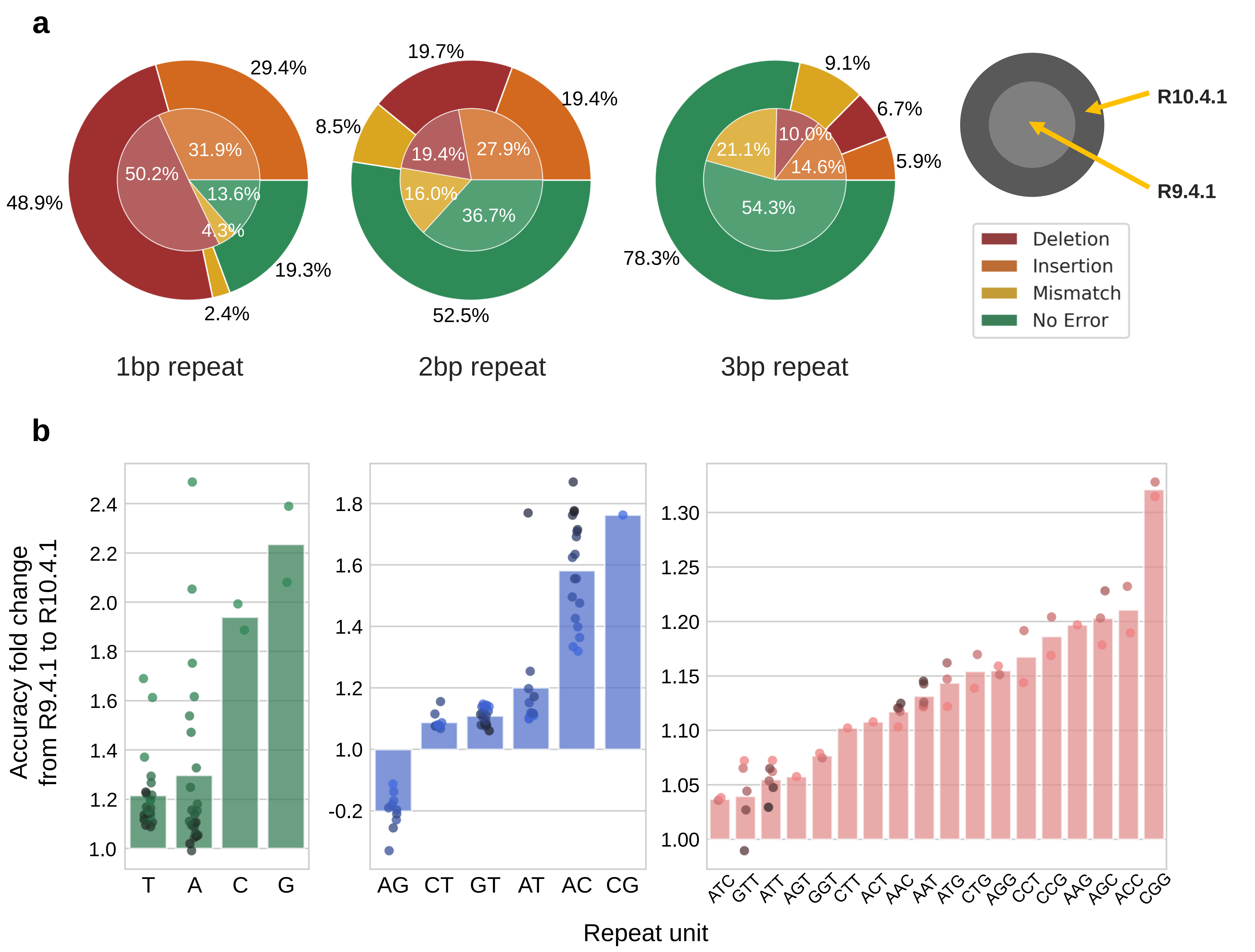

Figure 7. Comparison of ONT STR sequencing profile between R9.4.1 and R10.4.1 flowcell
(a) Distribution of correctly sequenced reads and sequencing errors in 1bp-, 2bp- and 3bp-repeat STR compared between R9.4.1 (HG002 R9.4.1 dataset) and R10.4.1 (HG002 R10.4.1 dataset). **(b)** Change in sequencing accuracy from R9.4.1 to R10.4.1 of various types of STR. Accuracy change is measured by dividing the sequencing accuracy observed in R9.4.1 with the sequencing accuracy of R10.4.1 (e.g., 2.0 indicates 200% improvement). The dots in each bar plot represents STR of fixed lengths, with longer STR represented by darker colors.

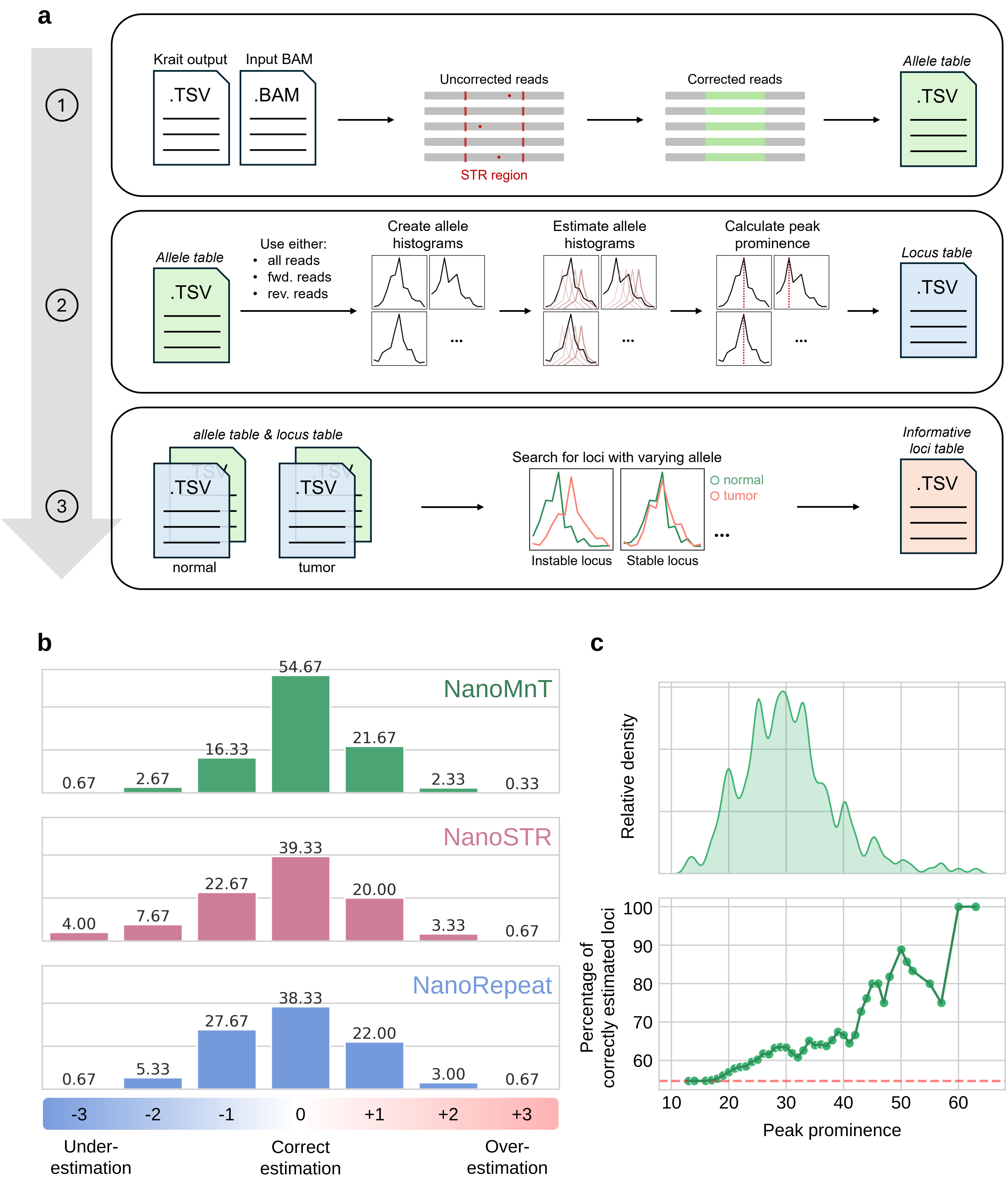
a
Krait output
Input BAM
.TSV
.BAM
Uncorrected reads
STR region
Corrected reads
Allele table
.TSV
1
2
3
Allele table
.TSV
Use either:
• all reads
• fwd. reads
• rev. reads
Create allele histograms
Estimate allele histograms
Calculate peak prominence
Locus table
.TSV
allele table & locus table
.TSV
.TSV
normal
tumor
Search for loci with varying allele
normal
tumor
Instable locus
Stable locus
Informative loci table
.TSV
b
NanoMnT
0.67
2.67
16.33
54.67
21.67
2.33
0.33
NanoSTR
4.00
7.67
22.67
39.33
20.00
3.33
0.67
NanoRepeat
0.67
5.33
27.67
38.33
22.00
3.00
0.67
-3
-2
-1
0
+1
+2
+3
Under-estimation
Correct estimation
Over-estimation
c
Relative density
Percentage of correctly estimated loci
100
90
80
70
60
10
20
30
40
50
60
Peak prominence

Figure 8. Development of NanoMnT

(a) Schematic overview of NanoMnT functionality. First, NanoMnT performs rudimentary STR error correction for reads and generates a tab-delimited file (TSV) named Allele Table. Allele table is then used to estimate STR allele size of user-specified STR loci by comparing the observed STR allele size histogram against many synthetic STR allele size histograms, generating another TSV file, namely Locus Table. Given Allele Table and Locus Table of paired normal and tumor samples, NanoMnT compares the STR allele size histogram of commonly captured STR loci to search for loci that could provide useful information regarding the tumor's MSI status. **(b)** Benchmark result of NanoMnT, NanoSTR and NanoRepeat in estimating STR allele size of 300 1bp-repeat STR loci. **(c)** Distribution of peak prominence value (which indicates the prominence of the STR allele size histogram of each STR locus, calculated using SciPy find_peaks function) reported by NanoMnT (top), and the change in percentage of STR loci whose allele size have been correctly estimated by thresholding peak prominence (bottom). For example, ~90% of STR loci whose peak prominences exceed 50 are correctly estimated. The red dashed horizontal line represents the total percentage of STR loci that have been correctly estimated by NanoMnT, as shown in Figure b.

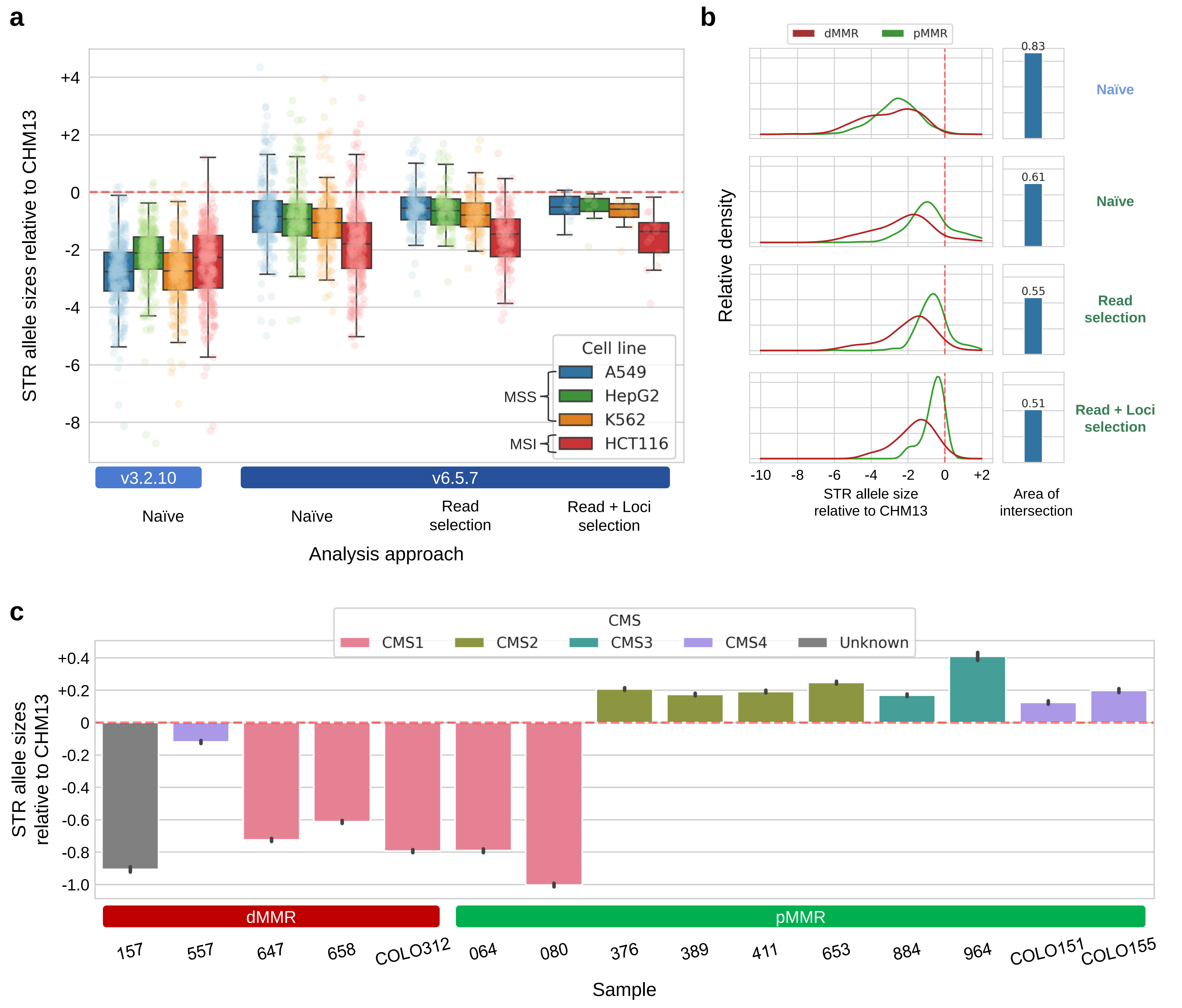

Figure 9. MSI identification results of SG-NEx and CRC organoid WGS sequencing data
(a) Distribution of STR allele size relative to CHM13 in 4 cancer cell lines, visualized by box plots and strip plots. 4 analysis approaches are compared; Naïve (Guppy v3.2.10), Naïve, read selection approach, and read + loci selection approach. **(b)** Comparison of STR allele size distribution between MSS cell lines and MSI cell line when employing the 4 different analysis approaches, visualized by kernel density estimate plots (left) and the area of intersection between the kernel density estimate plots of MSS and MSI. The red dashed vertical lines in the left figure represents the reference STR allele size (zero). **(c)** Average STR allele sizes of 15 CRC samples visualized by bar plots, with each sample colored by its reported CMS type.

 Click here to access/download;Figure;Supplementary figures raw.pptx

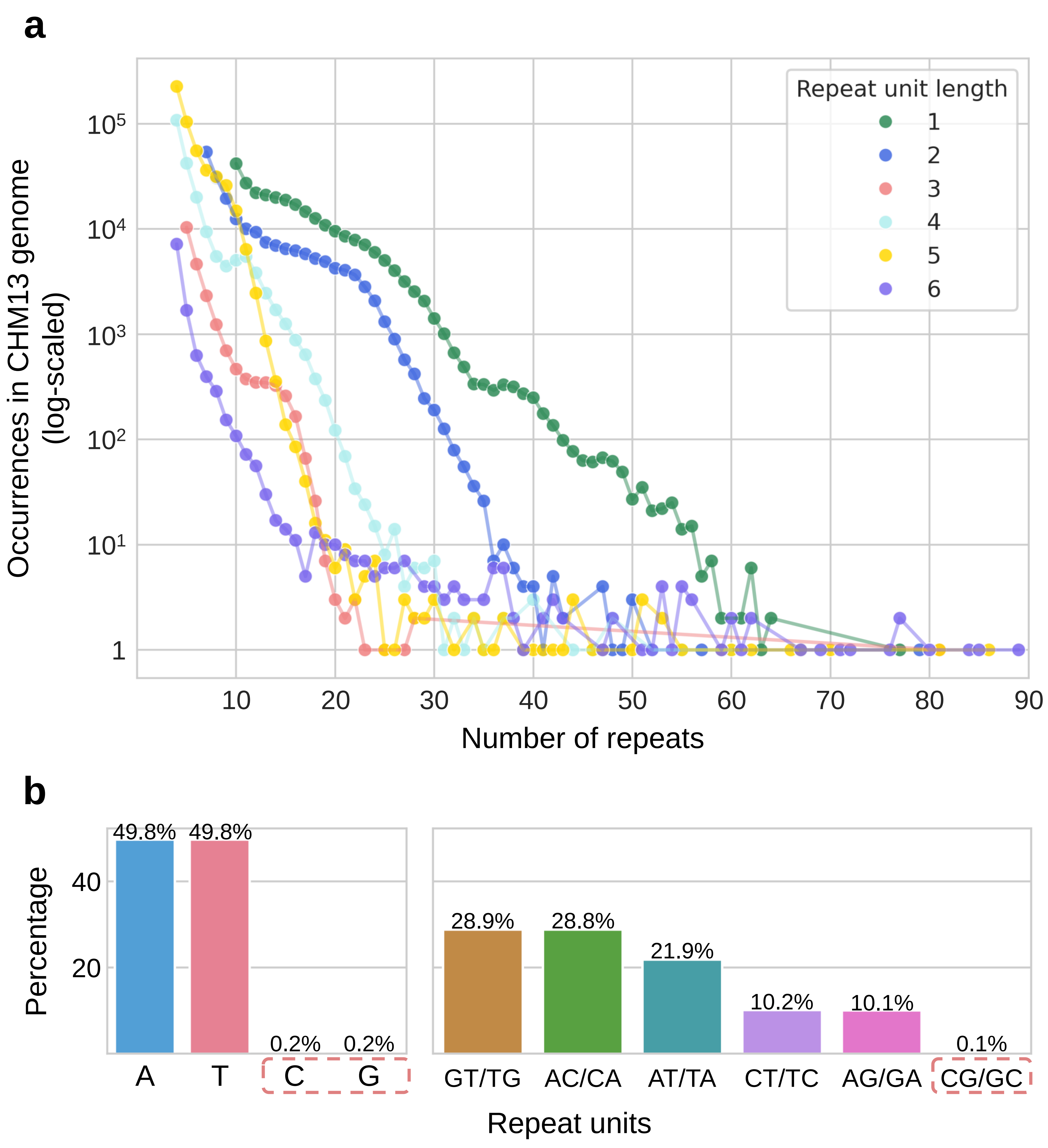

Supplementary Figure 1.
(a) Number of STR regions analyzed in this study. STR regions were identified from the T2T-CHM13 (v2.0) using Krait with default parameters, then preprocessed to filter out STR with low-complex flanking sequences. **(b)** Percentage of 1bp-/2bp-repeat STR by repeat units. CG-rich STR were very rare in the human genome.

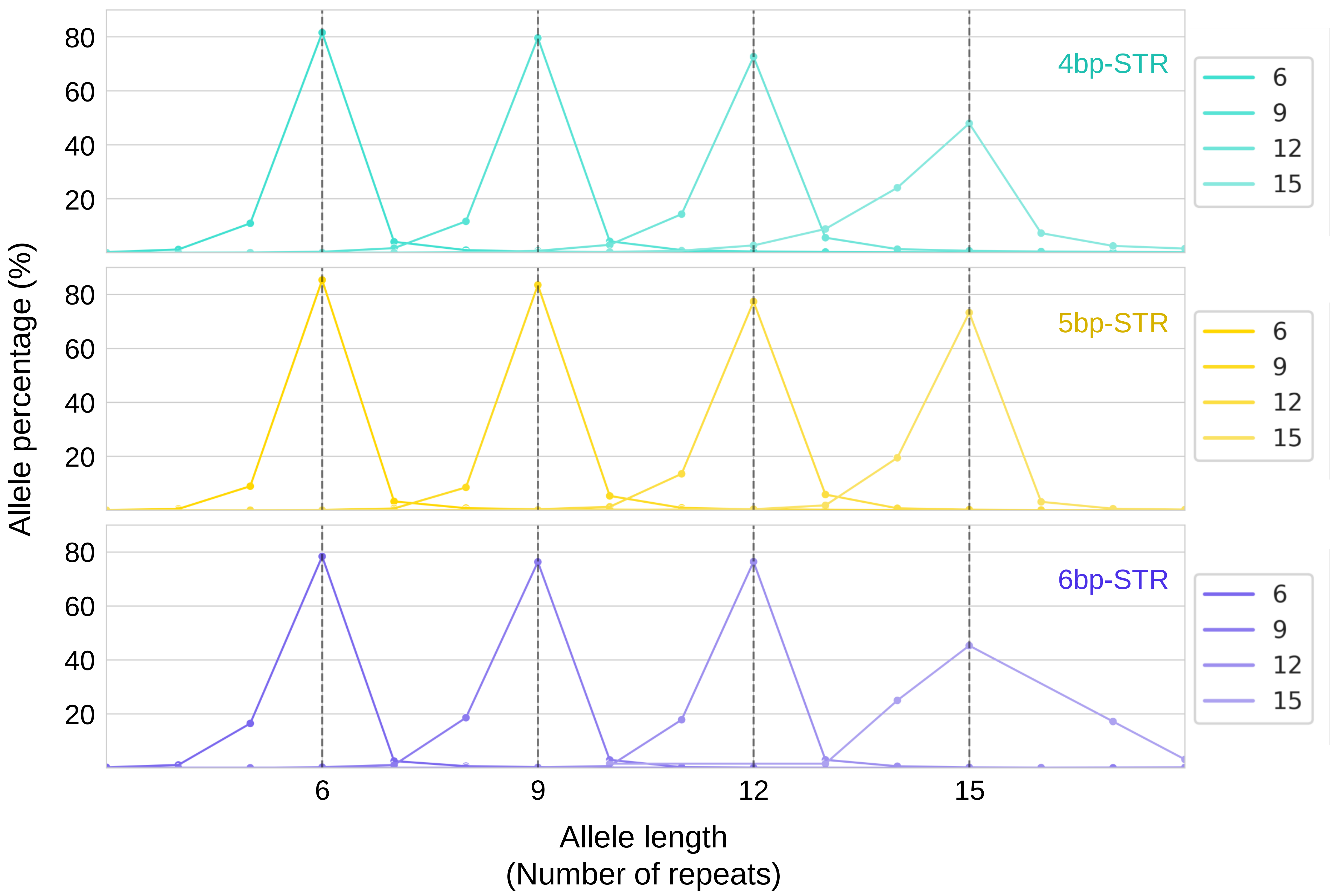

Supplementary Figure 2.
STR allele size histogram of various lengths of 4bp-, 5bp- and 6bp-repeat STR.

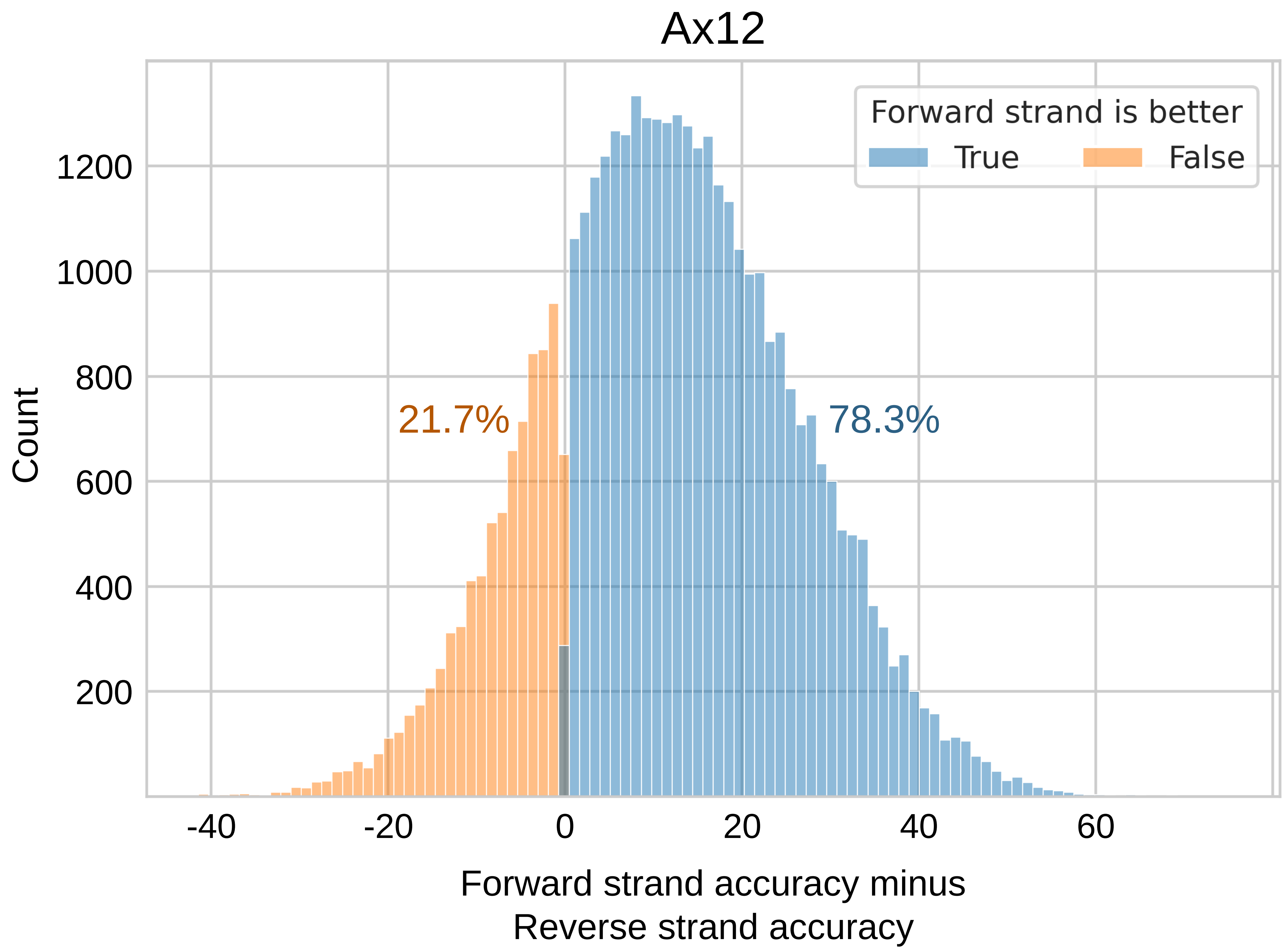

Supplementary Figure 3.
Distribution of sequencing accuracy of forward strand reads (i.e., percentage of errorless reads among forward strand reads) minus sequencing accuracy of reverse strand reads (i.e., percentage of errorless reads among reverse strand reads), in Ax12 STR. 21.7% of Ax12 STR loci exhibited better sequencing accuracy when using reverse strand reads, while the remaining 78.3% of Ax12 STR loci exhibited the opposite.

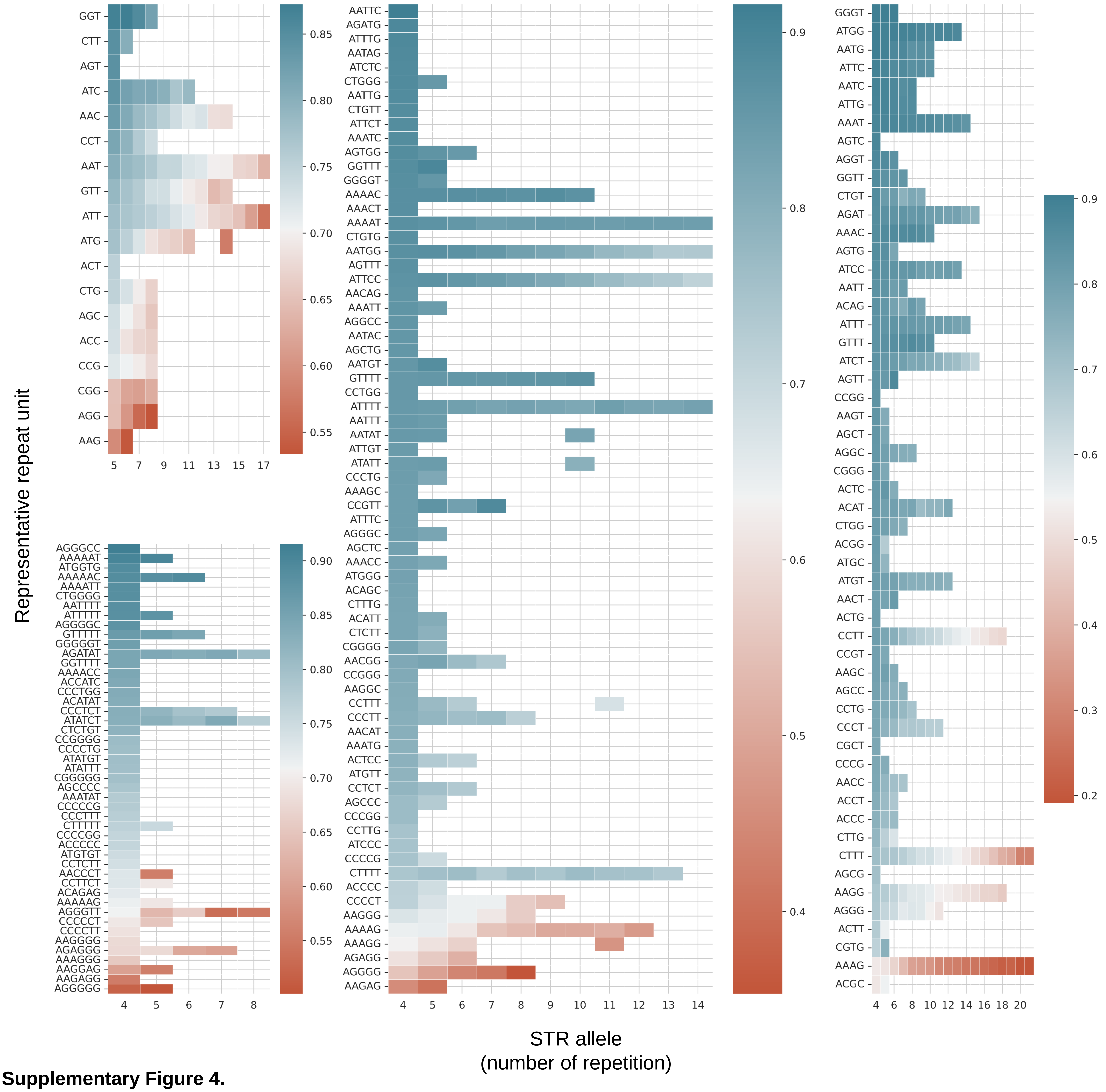

Supplementary Figure 4.
Sequencing accuracy of 3bp-, 4bp-, 5bp- and 6bp-repeat STR. Synonymous STR types (e.g., ACG-, CGA-, GAC-repeats) are grouped together and represented by the ‘representative’ repeat unit (e.g., ACG-repeat). STR with at least 30 observations were included in this plot.

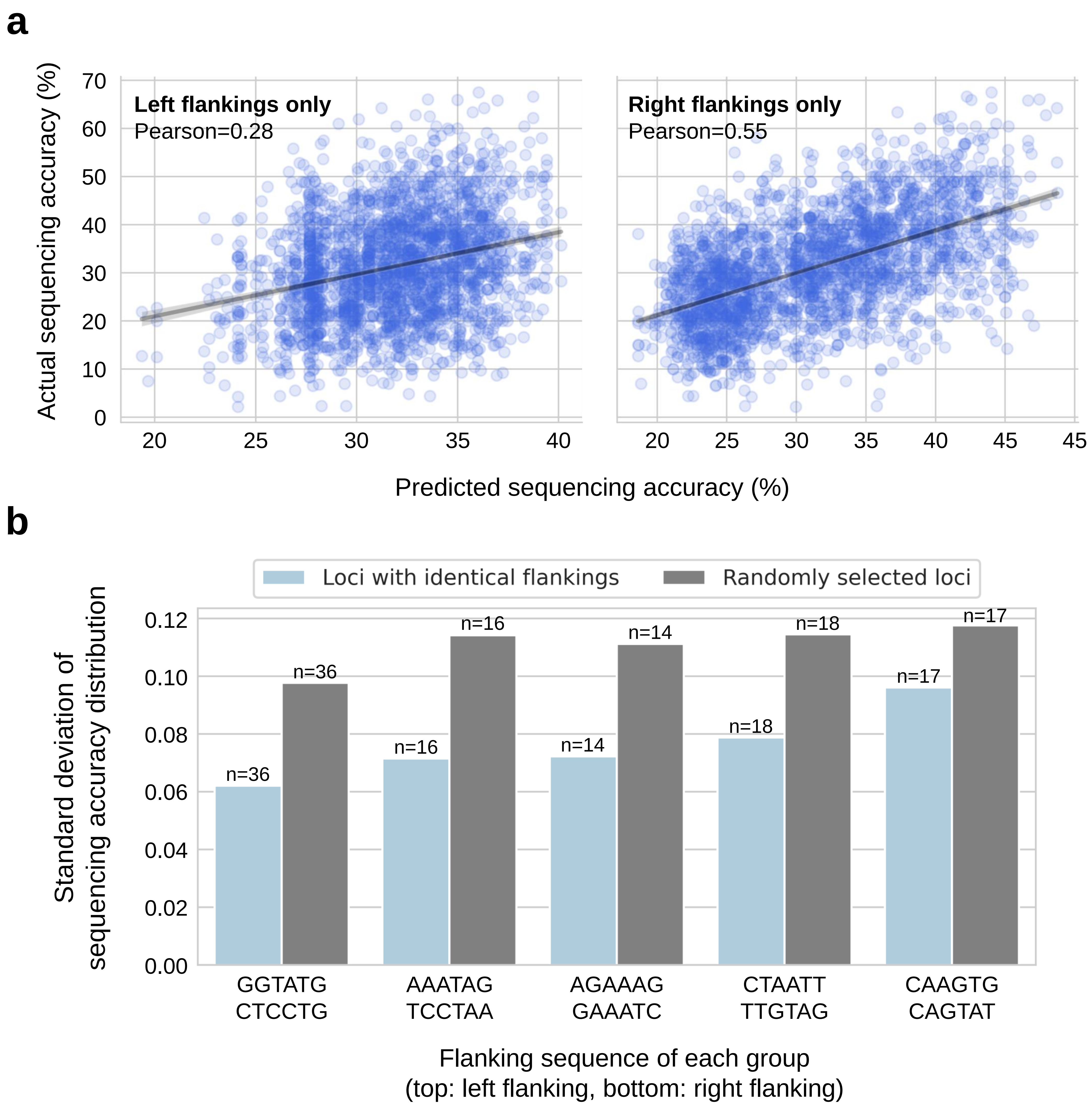

Supplementary Figure 5.
(a) Prediction of sequencing accuracy of Ax10 STR using left or right flanking sequences as inputs. **(b)** Standard deviation of sequencing accuracy of Ax10 STR that share the identical flanking sequences, compared against randomly sampled Ax10 STR, demonstrating that flanking sequences indeed influence the sequencing accuracy of A-repeat STR.

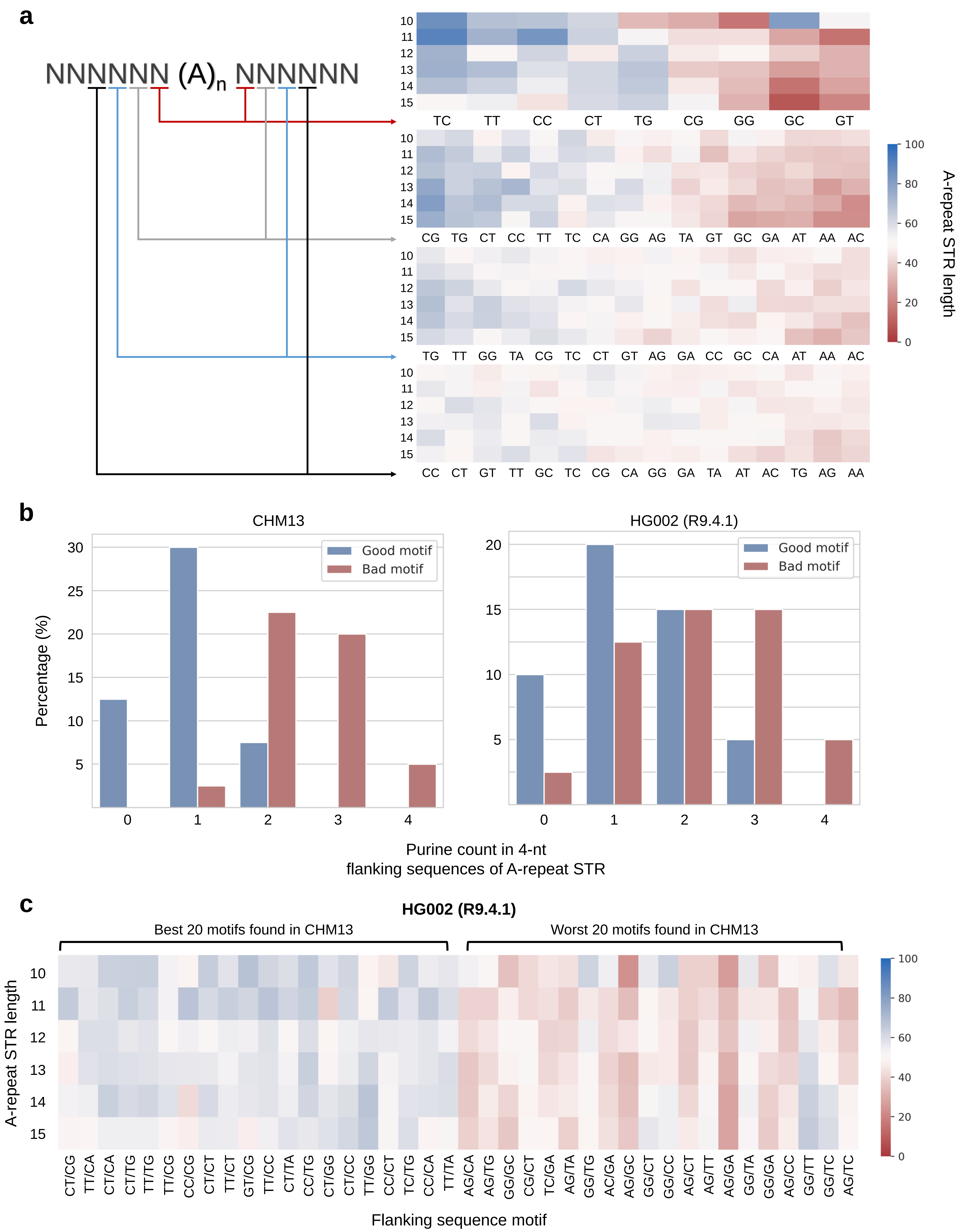

Supplementary Figure 6.
(a) Sequencing accuracy of A-repeat STR that possesses specific pairs of nucleotides in specific distances within their flanking sequences. The influence of nucleotide pair on sequencing accuracy is proportionate to its proximity to A-repeat STR. **(b)** Number of purine counts in flanking sequences of 4 nucleotide (2 nucleotide in each direction) **(c)** (HG002 R9.4.1 dataset) Sequencing accuracy of A-repeat STR that harbor certain motifs in their flanking sequences, ordered by the 'best' and 'worst' motifs found in the CHM13 dataset.

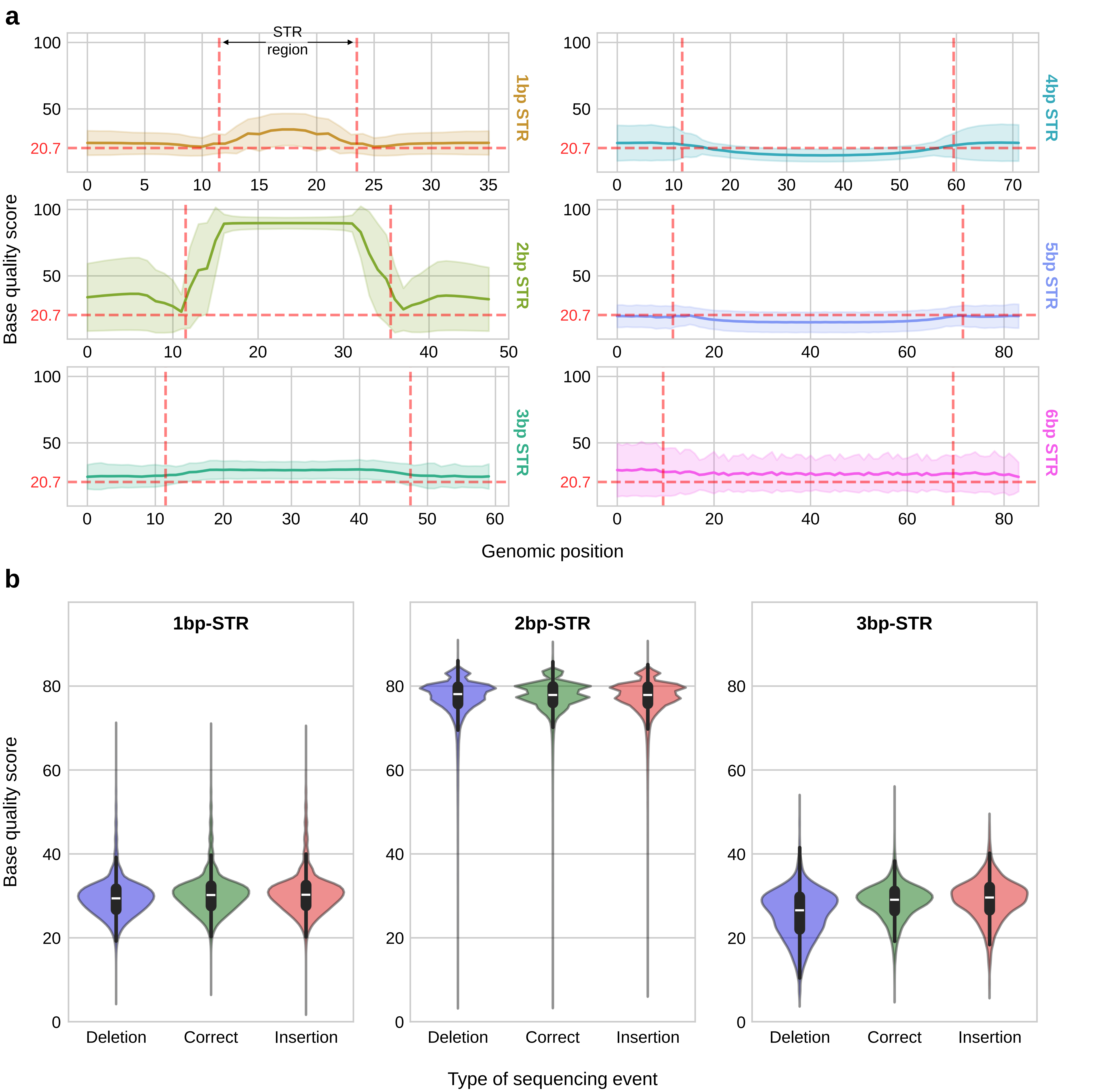

Supplementary Figure 7.
(a) Base quality of STR regions. The two vertical lines represent the start and end of the repeat sequences, while the horizontal lines represent the average base quality of the CHM13 dataset, highlighting the base quality 'burst' observed within the STR regions of some STR types. **(b)** Average base quality score of reads that are presumed to harbor indel errors in STR regions.

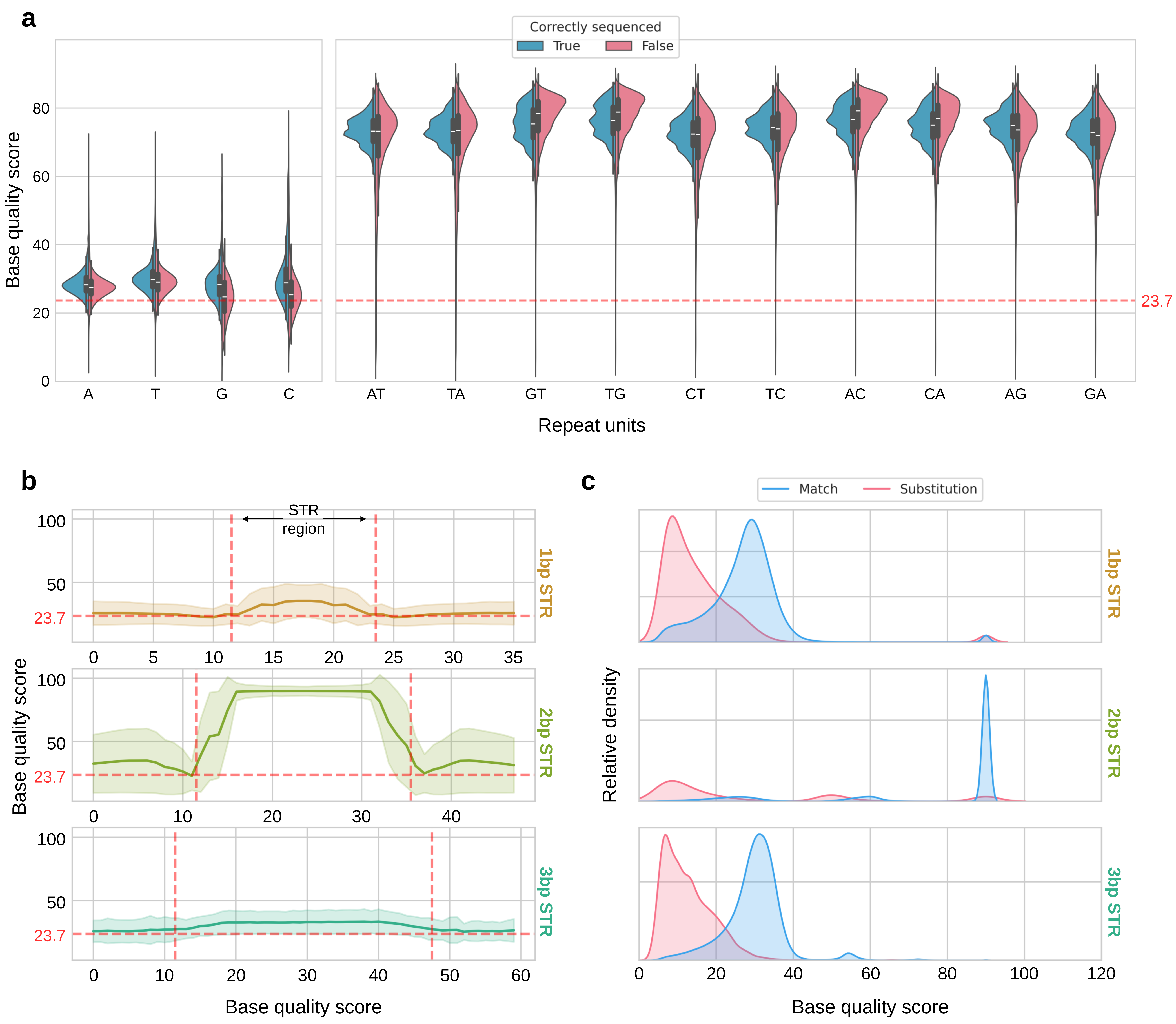

Supplementary Figure 8.
(a) The average base quality of reads in various STR types observed in the HG002 R9.4.1 dataset, comparing correctly sequenced reads (i.e., reads with no error within STR region) against incorrectly sequenced reads. The horizontal line represents the average base quality of the HG002 R9.4.1 dataset. **(b)** The base quality 'burst' observed in the HG002 R9.4.1 dataset. **(c)** Distribution of base quality compared between correctly sequenced bases and substitution errors.

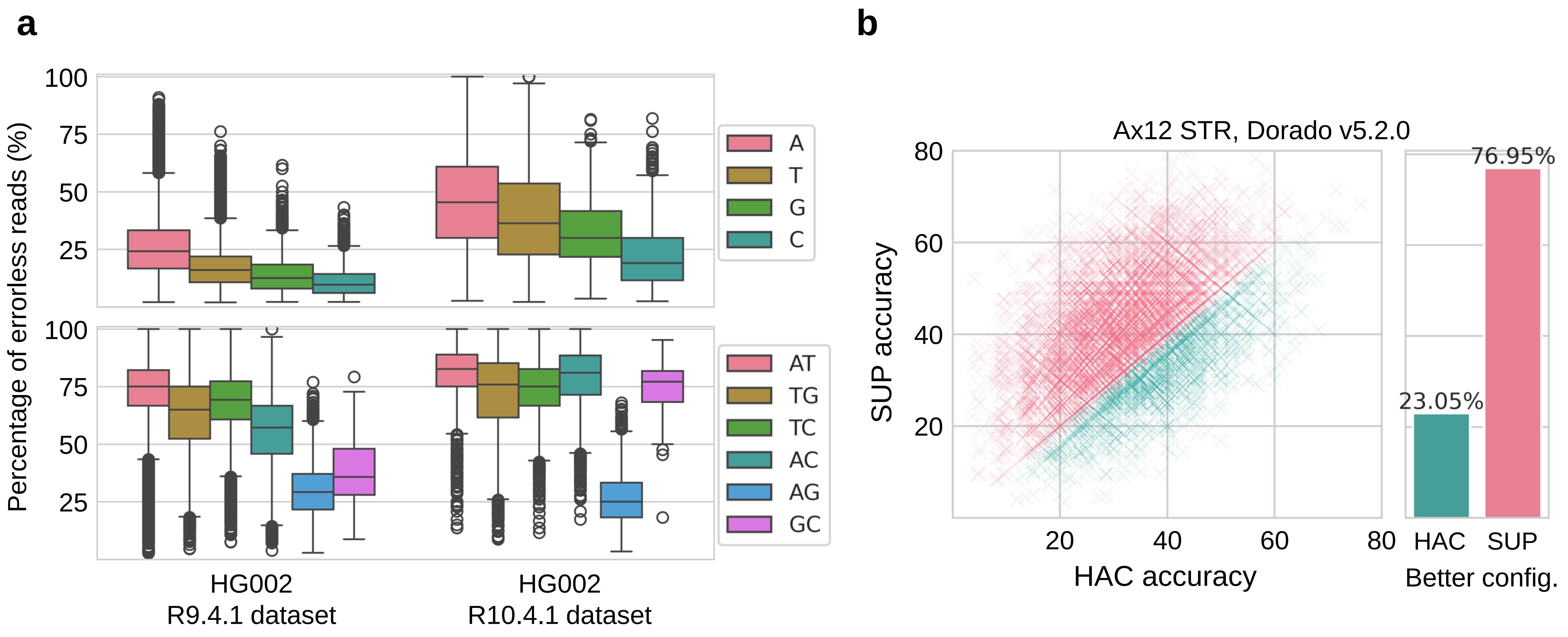

Supplementary Figure 9.
(a) Sequencing accuracy of STR by various repeat units, compared between HG002 R9.4.1 dataset and HG002 R10.4.1 dataset. **(b)** Sequencing accuracy of Ax12 STR observed in HG002 R10.4.1 dataset, comparing HAC basecalling model against SUP basecalling model. Each cross in the scatterplot (left) represents a Ax12 STR locus. Around 76.95% of Ax12 STR are better resolved using SUP basecaller model, whereas 23.05% of Ax12 STR are better resolved using HAC basecaller model (right).

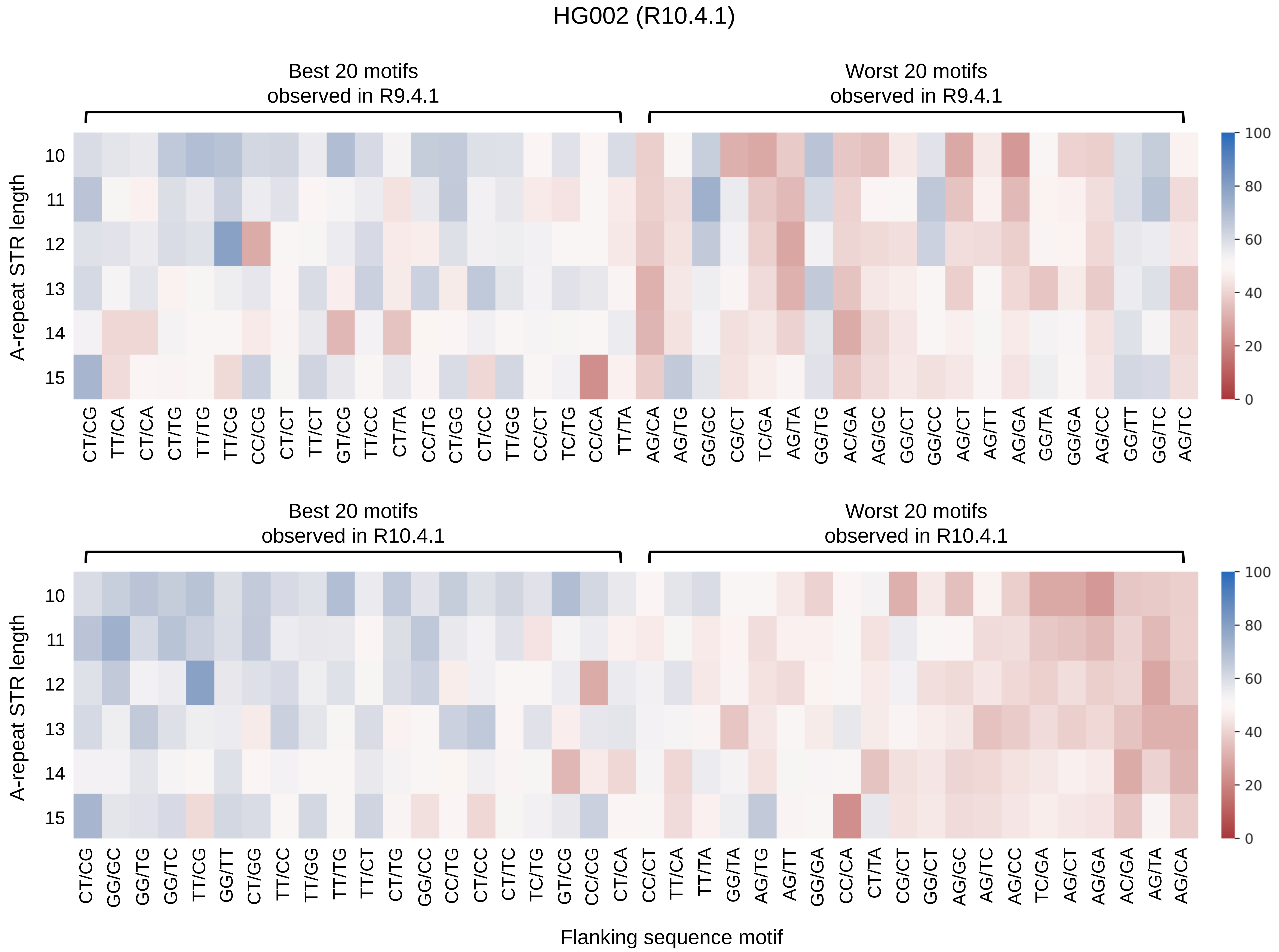

Supplementary Figure 10.
(HG002 R10.4.1 dataset) Sequencing accuracy of A-repeat STR that harbor certain motifs in their flanking sequences, ordered by the 'best' and 'worst' motifs found in the CHM13 dataset (top) and the HG002 R10.4.1 dataset (bottom).

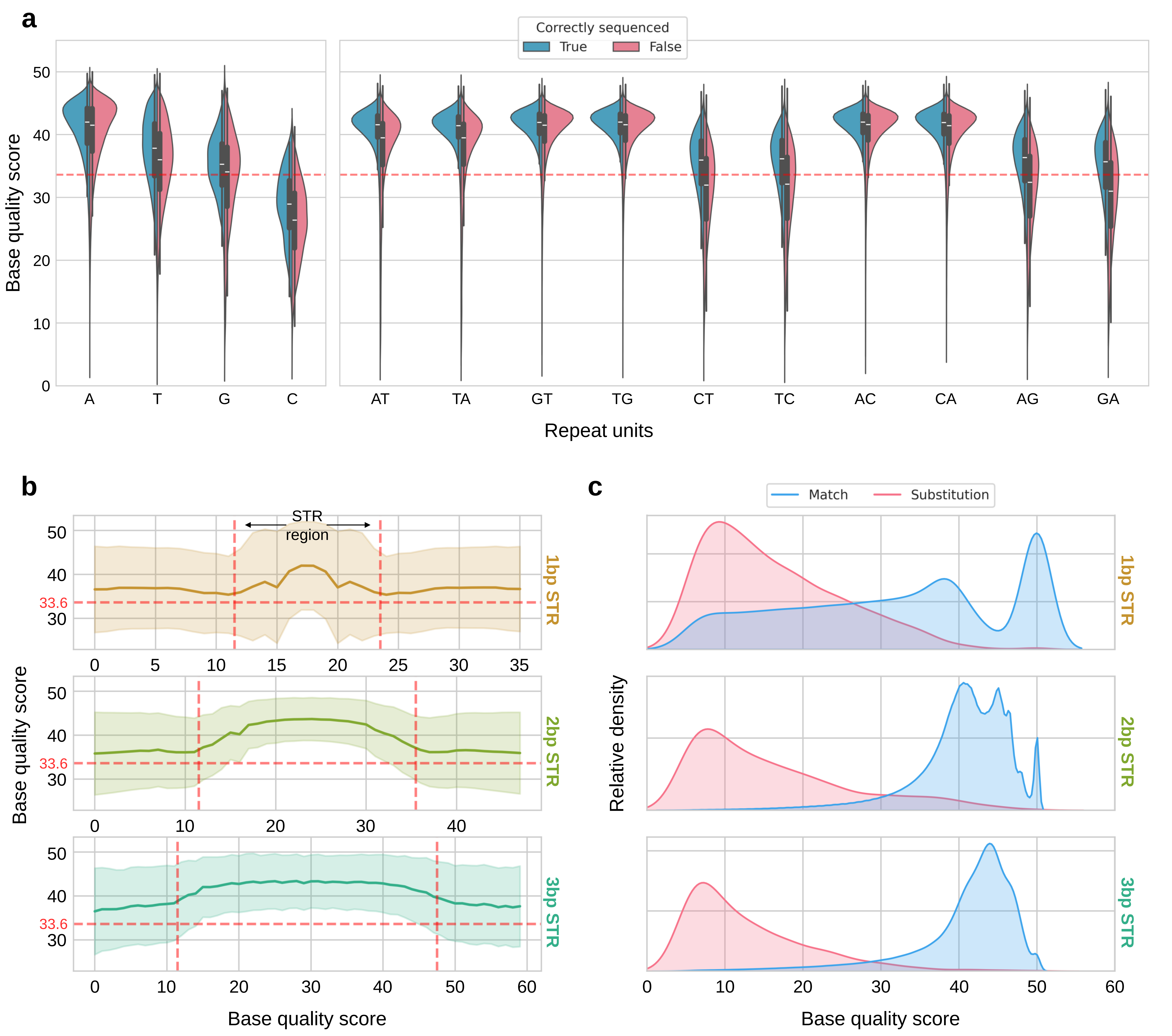

Supplementary Figure 11.
(a) The average base quality of reads in various STR types observed in the HG002 R10.4.1 dataset, comparing correctly sequenced reads (i.e., reads with no error within STR region) against incorrectly sequenced reads. The horizontal line represents the average base quality of the HG002 R10.4.1 dataset. **(b)** The base quality 'burst' observed in the HG002 R10.4.1 dataset. **(c)** Distribution of base quality compared between correctly sequenced bases and substitution errors.

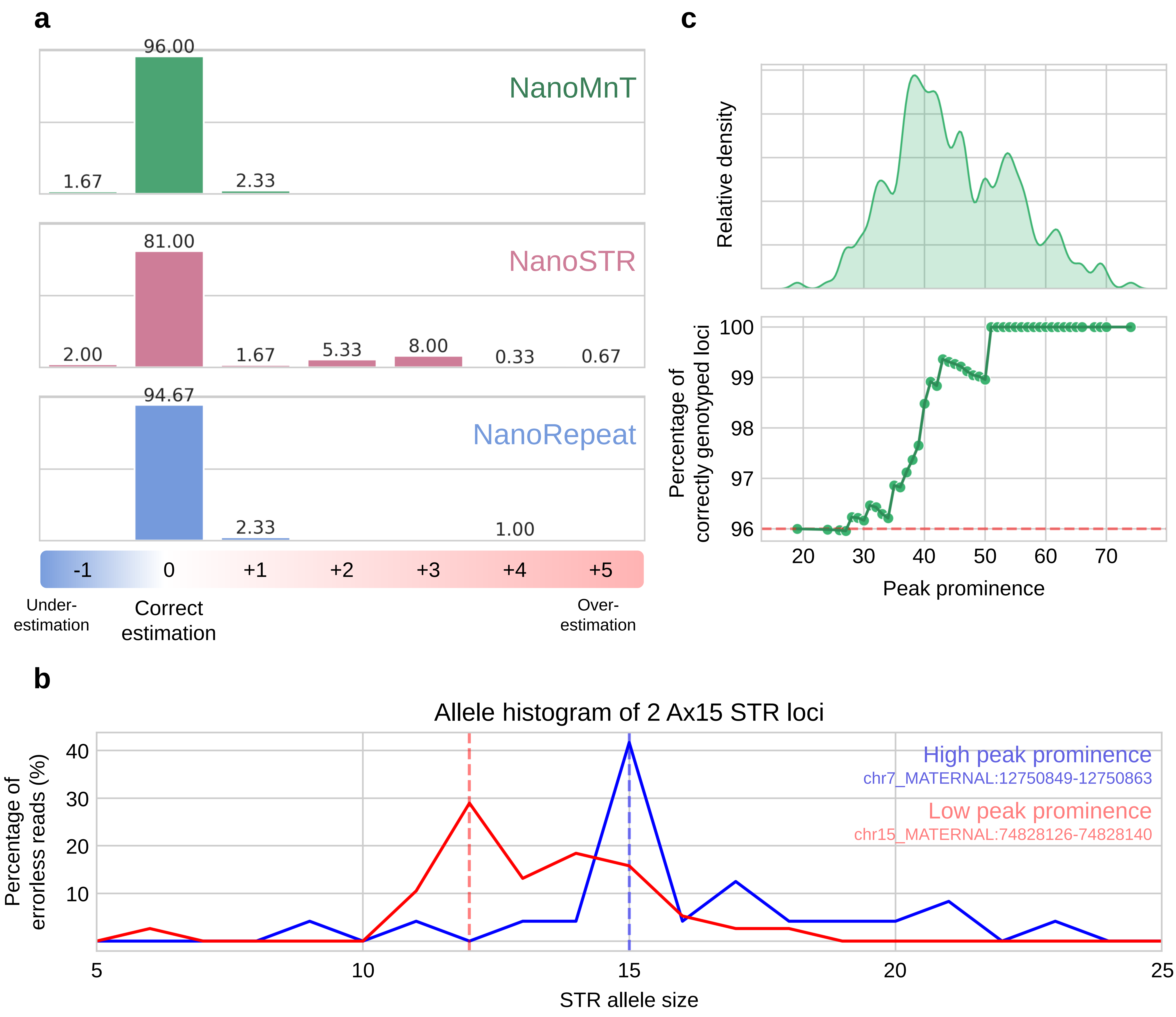

Supplementary Figure 12.
(a) NanoMnT, NanoSTR and NanoRepeat genotyping results of 300 2bp-repeat STR loci. **(b)** STR allele size histograms of 2 example Ax15 loci, one with a highly prominent peak, and the other with a less prominent peak. The dashed line represents the genotyped STR allele for each locus. **(c)** Kernal density estimate plot of the peak prominences of each genotyped STR locus (top), and percentage of correctly genotyped loci based on using peak prominence as thresholds (bottom). For example, ~98.5% of loci whose peak prominence exceed 40 are correctly genotyped.
