# Supplementary material for: NanoMnT: an STR analysis tool for Oxford Nanopore sequencing data driven by a comprehensive analysis of error profile in STR regions
Source: Gigascience. 2025 Mar 17;14:giaf013. doi: 10.1093/gigascience/giaf013 (PMC11912559; doi:10.1093/gigascience/giaf013)
Supplement: giaf013_GIGA-D-24-00346_Revision_2 [file giaf013_giga-d-24-00346_revision_2.pdf]

GigaScience

NanoMnT: An STR Analysis Tool for Oxford Nanopore Sequencing Data Driven by a Comprehensive Analysis of Error Profile in STR regions.

--Manuscript Draft--

**Manuscript Number:**	GIGA-D-24-00346R2
**Full Title:**	NanoMnT: An STR Analysis Tool for Oxford Nanopore Sequencing Data Driven by a Comprehensive Analysis of Error Profile in STR regions.
**Article Type:**	Research
**Funding Information:**	GIST (GIST-MIT Research Collaboration grant) — Dr Jihwan Park<br>GIST (GIST-CNUH Research Collaboration grant) — Dr Jihwan Park<br>National Research Foundation of Korea (RS-2024-00335026) — Dr Jihwan Park<br>Korea Technology and Information Promotion Agency for SMEs (RS-2024-00506966) — Dr Jihwan Park<br>National Natural Science Foundation of China (82103031) — Dr Han Luo<br>National Natural Science Foundation of China (82272933) — Dr Han Luo<br>National Natural Science Foundation of China (82422050) — Dr Han Luo
**Abstract:**	Oxford Nanopore sequencing (ONT) is a third-generation sequencing technology that enables cost-effective long-read sequencing, with broad applications in biological research. However, its high sequencing error rate in low-complexity regions hampers its applications in short tandem repeat (STR) related research. To address this, we generated a comprehensive STR error profile of ONT by analyzing publicly available Nanopore sequencing datasets. We show that the sequencing error rate is influenced not only by STR length but also by the repeat unit and the flanking sequences of STR regions. Interestingly, certain flanking sequences were associated with higher sequencing accuracy, suggesting that certain STR loci are more suitable for Nanopore sequencing compared to other loci. While base quality scores of substitution errors within the STR regions were lower than those of correctly sequenced bases, such patterns were not observed for indel errors. Furthermore, choosing the most recent basecaller version and using the super accuracy (SUP) model significantly improved STR sequencing accuracy. Finally, we present NanoMnT, a lightweight Python tool that corrects STR sequencing errors in sequencing data and estimates STR allele sizes. NanoMnT leverages the characteristics of ONT when estimating STR allele size and exhibits superior results for 1bp- and 2bp-repeat STR compared to existing tools. By integrating our findings, we improved STR allele estimation accuracy for Ax10 repeats from 55% to 78%, and up to 85% when excluding loci with unfavorable flanking sequences. Using NanoMnT, we present the utility of our findings by identifying microsatellite instability (MSI) status in cancer sequencing data. NanoMnT is publicly available at https://github.com/18parkky/NanoMnT.
**Corresponding Author:**	Jihwan Park<br>Gwangju Institute of Science and Technology<br>Gwangju, KOREA, REPUBLIC OF
**Corresponding Author Secondary Information:**	
**Corresponding Author's Institution:**	Gwangju Institute of Science and Technology
**Corresponding Author's Secondary Institution:**	

Powered by Editorial Manager® and ProduXion Manager® from Aries Systems Corporation

**First Author:**	Gyumin Park
**First Author Secondary Information:**	
**Order of Authors:**	Gyumin Park
	Hyunsu An
	Han Luo, PhD
	Jihwan Park, PhD
**Order of Authors Secondary Information:**	
**Response to Reviewers:**	Dear Editor of GigaScience,<br><br>We would like to express our sincere thanks and appreciation for your kind words and help.<br><br>We have filled in the missing required sections in our manuscript, including various citations, author contributions, citation styles, etc.<br><br>We would like to mention that while going through the Dome-ML annotation of our machine learning model used in our study, we found a minor mistake in our manuscript regarding the number of data points used for training/validating the model. During revision, we incorporated additional data points (STR loci), but we did not update the corresponding numbers. The numbers, which are at line 326 and 330, are now corrected.<br><br>Thank you for your kind guidance, and please don't hesitate to let us know if there is anything to change.<br><br>Best regards,<br>Jihwan
**Additional Information:**	
**Question**	**Response**
Are you submitting this manuscript to a special series or article collection?	No
**Experimental design and statistics**<br><br>Full details of the experimental design and statistical methods used should be given in the Methods section, as detailed in our Minimum Standards Reporting Checklist. Information essential to interpreting the data presented should be made available in the figure legends.<br><br>Have you included all the information requested in your manuscript?	Yes
**Resources**<br><br>A description of all resources used, including antibodies, cell lines, animals	Yes

Powered by Editorial Manager® and ProduXion Manager® from Aries Systems Corporation

and software tools, with enough information to allow them to be uniquely identified, should be included in the Methods section. Authors are strongly encouraged to cite Research Resource Identifiers (RRIDs) for antibodies, model organisms and tools, where possible.<br><br>Have you included the information requested as detailed in our Minimum Standards Reporting Checklist?	
**Availability of data and materials**<br><br>All datasets and code on which the conclusions of the paper rely must be either included in your submission or deposited in publicly available repositories (where available and ethically appropriate), referencing such data using a unique identifier in the references and in the "Availability of Data and Materials" section of your manuscript.<br><br>Have you have met the above requirement as detailed in our Minimum Standards Reporting Checklist?	Yes

Powered by Editorial Manager® and ProduXion Manager® from Aries Systems Corporation

Click here to access/download;Manuscript;Revision_Comments.docx

We thank the reviewers for the insightful comments and suggestions, which we believe to have been vital for addressing important issues that have went unnoticed in our initial submission. We have addressed the reviewers' feedback point-by-point, bringing major changes to our manuscript.

Among many changes, we would like to specifically mention 2 major ones: (1) In our initial analysis, we have divided the dataset into multiple batches when analyzing STR-aligned reads due to the sheer size of the datasets (especially the CHM13 dataset). During the revision process, we noticed that few of the batches were not successfully processed. In other words, a minor portion of the data were left out in our initial analysis. We incorporated these into the analysis, which introduced subtle changes to the some of the Figures. However, we ensure that no major changes are observed in terms of the main contents and the flow of our manuscript. (2) Furthermore, we noticed that Dorado has been updated since our initial analysis of the R10.4.1 dataset (HG002 dataset). This has prompted us to re-analyze the HG002 R10.4.1 dataset using the most recent version of Dorado (v8.1.0 as of October 3rd, 2024).

Please see below for our detailed response to each feedback.

Reviewer 1

1) *Introduction: the discussion of existing tools needs to be expanded. This paper (https://www.nature.com/articles/s41592-023-01932-w#Sec16) has investigated 10 tools to estimate repeat size from long-read sequencing data, while this paper only discussed two of them. It is better to show the advantages of their tools compared to the 10 tools.*

The reviewer raises an important point, and we thank the reviewer for suggesting a paper (Ahsan, M.U., Liu, Q., Perdomo, J.E. et al.) for reference. In our revised manuscript, we now discuss 7 additional tools (9 total) in our Introduction section (please see line 94-109), discussing their features and limitations.

2) *CNN: STR regions might have different lengths but are used as input of CNN.*

2-1) *How they make CNN flexible to tolerate sequences with different lengths.*

The CNN model takes flanking sequences – **12 bases (6 bases from both left and right side of the STR region)** – as inputs, rather than the STR sequences themselves. For example, consider two A-repeat STRs with different number of repeats:

- STR locus 1: ⋯GATCGT $(A)_{12}$ CGATAA⋯
- STR locus 2: ⋯ACTATC $(A)_{15}$ TCCGAG⋯

While the lengths of the A-repeat sequences of the two loci differ, the model does not need to be flexible to the flanking sequence length because we simply select the 6 bp flanking of each direction as inputs.

2-2) *What is the definition of sequencing accuracy?*

Thank you for bringing this up to our attention. Although we have stated that '*the sequencing accuracy of STR is measured by calculating the percentage of errorless reads, and these two terms are used interchangeably throughout the study*' in our Result section, we recognize that this mention may be too brief and easily overlooked by readers. Therefore, to ensure that it stands out better to the readers, we first declare the definition of sequencing accuracy in the beginning of the second paragraph in the Results section (line 309-311).

2-3) *The input of the CNN is read sequences or reference sequences?*

The inputs are reference sequences (T2T-CHM13 v2.0), which represent the ground-truth sequences. Using the read sequences as inputs are out the scope of our interest because the purpose of employing the CNN model in our analysis was to explore the relationship between <u>the sequencing accuracy of a given STR locus</u> and <u>the actual flanking sequences of that locus</u>.

3) *NanoMnT error correction: how to define a list of possible alleles for each aligned read?*

We thank the reviewer for highlighting this important aspect of NanoMnT error correction process. When defining the list of possible alleles (i.e., number of repeats), NanoMnT assumes that the allele for a given aligned read falls between 0 repeats (lower bound) and the longest allele among all the reads (determined by counting the number of repeats <u>prior to error correction</u>) plus 5 additional repeats (upper bound). The 5 additional repeats serve as a buffer to account for potential outliers.

Read 1: AA**C**AAAAAAA (length: 10 bases)

Read 2: AAAAAAA (length: 7 bases)

Read 3: AAAAAAAAAA**T**AA (length: 13 bases)

In this case, the upper bound of the list of possible alleles is defined as 13 + 5 = 18 repeats. The Levenshtein distance between the STR sequence of each read and the list of possible alleles (0 repeats, 1 repeat, …, 18 repeats) is then calculated. In our revised manuscript, we now include a more in-depth explanation in the Methods section (line 257-262).

4) *It is great to share codes on github. But it is helpful to provide simple installations of the dependencies and their tool. It is also useful to provide instructions on how to re-generate their results.*

We agree with the reviewer regarding the good practices of Github code-sharing. We have significantly updated and improved our NanoMnT GitHub repository, providing detailed explanations of each NanoMnT commands and their respective outputs. Furthermore, we provide a tutorial Python notebook for detecting MSI status using ONT sequencing data.

5) *It is great to show how to use NanoMnT on cancer samples.*

5-1) *However, it is not clear how many loci are used for SG-Nex data and CRC data.*

We greatly thank the reviewer for the positive comment. The number of loci used when identifying MSI status differs by the strategy we applied. When analyzing the SG-NEx dataset, 407 loci were used for the 'v3.2.10' strategy, 347 loci for the 'v6.5.7 Naïve' strategy, 174 loci for the 'v6.5.7 Read selection strategy, 21 loci for the 'v6.5.7 Read + Loci selection' strategy. When analyzing the CRC WGS dataset, a total of 218,205 loci were used. In our revised submission, this information is added to our Supplementary Table 3 and 4.

5-2) *Also, there is no comparison between NanoMnT and other tools.*

We did not compare other tools against NanoMnT when analyzing cancer samples (Figure 10), as we have dedicated a Result section for tool comparison & benchmarking (Figure 8).

5-3) *Figure 9(c) y-axis: -1 means 1bp difference? If it is true, the average difference is less than 1 bp usually and might not be significant.*

We appreciate the reviewer's attention to detail. The reviewer is correct that the -1 in y-axis of Figure 9c indicates 1 base pair difference and MSI samples show difference less than 1 base pair. However, we believe this to be quite significant, as this indicates that a significant proportion of homopolymer microsatellite loci have undergone a -1 base pair deletion event, which have been shown to be most prevalent type of MSI event in recent studies[1,2].

---

[1] Ballhausen, A., Przybilla, M.J., Jendrusch, M. *et al.* The shared frameshift mutation landscape of microsatellite-unstable cancers suggests immunoediting during tumor evolution. *Nat Commun* **11**, 4740 (2020). https://doi.org/10.1038/s41467-020-18514-5

[2] Westcott, P.M.K., Muyas, F., Hauck, H. *et al.* Mismatch repair deficiency is not sufficient to elicit tumor immunogenicity. *Nat Genet* **55**, 1686–1695 (2023). https://doi.org/10.1038/s41588-023-01499-4

Furthermore, if you look at the rightmost boxplots in Figure 9a (our most accurate results), you will see that the average difference is roughly around -1.3 bp, which is similar to the result of the putative MSI samples in the WGS dataset (Figure 9c), albeit with a slight difference. We attribute this difference due to different basecalling process: the WGS dataset was basecalled using 'Guppy 6,' as stated by the authors in their manuscript, and the raw FAST5 files were not provided, preventing us from re-basecalling the data using the latest basecaller.

6) *Many terms are not clearly defined, for example: similar terms like Ax10: a 10 "A" repeat region with "A" is repeat unit? What is pMMR? What is MSS?*

We apologize for the unclear usage of many terminologies. We now explicitly wrote the full form of each abbreviation (e.g., pMMR, MSS) and made sure to clearly distinguish similar terms.

7) *Many figures are not self-explanatory.*

7-1) *Figure 1: in (a), how to define errorless reads?*

We greatly appreciate the reviewer's perceptive observation. Errorless reads are defined as reads that do not harbor any sequencing errors (i.e., substitution, deletion, insertion). In other words, reads that report the ground-truth STR sequence are considered errorless reads. In our revised manuscript, the definition of errorless read is explained along with our definition of sequencing accuracy (line 309-311).

7-2) *Which datasets are used to generate error profiles?*

We apologize for the oversight in our original manuscript. All primary analyses for R9.4.1 ONT error profile and R10.4.1 ONT error profile were performed using the CHM13 dataset and the HG002 R10.4.1 dataset, respectively. In the Introduction section of our revised manuscript, we now state that 'unless otherwise specified, analyses of the R9.4.1 STR error profile were performed using the CHM13 dataset (line 119-120).

7-3) *Figure 2(b): figure legend for repeat units?*

Apologies, we now added in figure legend for Figure 2b.

7-4) *Figure 4(a): "N(A)nN" should be "B(A)nB" based on HGMD nucleotide symbols. (b): what do colors mean?*

We thank the reviewer for informing us this, we were not familiar with the HGMD nucleotide symbol B. We have updated Figure 4a accordingly. The colors of Figure 4b indicate the average sequencing accuracy of A-repeat STR loci that

harbors a specific flanking sequence. We have updated Figure 4b by adding a color bar.

7-4) *Figure 5(c): what is HAC/ SUP accuracy for a Ax10 STR locus?*

We have changed the figure legend for Figure 5c, to indicate that HAC/SUP accuracy each represents the basecaller model used.

7-5) *Figure 6(a): repeat regions have high base quality scores?*

Yes, which is indeed strange. One of the key findings is that the base quality scores in STR regions do not actually reflect the sequencing fidelity, and thus users must be cautious when inferring sequencing results from base qualities. We have dedicated two paragraphs in the Result section: 'Base quality score of sequencing error in STR regions.' To make the Figure slightly more self-explanatory, we now change the y-axis label of Figure 6a, 6b and x-axis label of Figure 6c from 'Base quality score' into 'Base quality score reported by basecaller'.

7-6) *Supplementary Figure 4: What does color indicate?*

We apologize for leaving out the figure legend for the color bar. The color indicates the observed sequencing accuracy for each type of STR. Cyan color indicates higher sequencing accuracy, whereas warm red color indicates lower sequencing accuracy. We have updated the figure legend (Supplementary Figure 5 in the revised manuscript) accordingly.

7-7) *Supplementary Figure 6: How to define "good" motifs and "bad" motifs? What does color indicate in (c)?*

We apologize for omitting an important detail for this figure. In the revised manuscript (Supplementary Figure 7 in the revised manuscript), we switched the presentation of our results, showing the relationship between the <u>Purine counts in the flanking sequences</u> (x-axis) and the <u>sequencing accuracy of A-repeat loci that harbor such flanking sequences</u> (y-axis). We hope this new format makes our Figure more straight-forward. The colors for Supplementary Figure 7c indicate the average sequencing accuracy of A-repeat STR loci that harbors a specific flanking sequence, much like Figure 4b. We updated this figure by adding a color bar legend.

Reviewer 2

1) *The abstract provides a clear and comprehensive summary of the study. However, it could be improved by highlighting the novelty of the work, % of accuracy corrected from their results and its potential impact on the field. There are no data showed as claimed.*

We thank the reviewer for the positive feedback regarding our Abstract section. To highlight the novelty of our study, we adjusted our Abstract section by highlighting the improvement of STR allele estimation accuracy for Ax10 repeats when integrating the findings made in this study.

2) T*he introduction provides a good overview of the background and significance of STR analysis.*

We thank the reviewer for the positive feedback and many valuable suggestions.

2-1) *However, it could be strengthened by providing more specific examples of how ONT's high error rate limits its use in STR analysis.*

In our Introduction section, we now discuss specific examples that well demonstrate the limitation of ONT in STR analyses. Please see line 83-86 for details.

2-2) *Additionally, the authors could discuss the limitations of existing tools in more detail, highlighting the need for NanoMnT.*

We thank the reviewer for a valuable suggestion. In our revised manuscript, we now provide additional discussion regarding the limitations of existing tools in our Introduction section. Please see line 94-109 for details.

2-3) *Also, report which nanopore sequencing platforms have what level of errors. As best of my knowledge it has been evolved significantly over the years.*

We thank the reviewer for an important suggestion. Indeed, ONT has released many versions of flow-cells and kits as well as other major changes, some iterations addressing the error rate in low-complexity regions. Thus, we now discuss the changes made for ONT over the past years in our Introduction section, including the most recent V14 kit, which ONT reports to provide sequencing accuracy of approximately 99%. Please see line 86-91 for details.

2-4) *Also, good to have some numbers from PacBio hifi sequencing too along with the cost comparison as discussed in this section.*

Thank you for the suggestion. Although we wished to include numbers for cost comparison between ONT and PacBio, we chose not to because of country/regional differences. However, we now added numbers for sequencing

accuracy and average read length of PacBio HiFi sequencing in our Introduction section. Please see line 77-80 for details.

3) *The methods section is generally well-written and provides sufficient detail for reproducibility. However, some areas could benefit from further clarification.*

We thank the reviewer for the positive comments and important suggestions.

3-1) For example, the authors could provide more information on the training and validation of the CNN model.

Thank you for your attention to detail. We have provided additional information regarding our CNN model, detailing prevention of overfitting, number of epochs, batch sizes, etc., in our Methods section (line 181-192).

3-2) Additionally, the authors could provide more details on the implementation of NanoMnT using the CNN and why they have used this over other machine learning algorithms.

We would like to clarify that NanoMnT does not use any machine learning. Instead, a CNN model was employed solely to explore the relationship between STR flanking sequences and STR sequencing accuracy (and has not been used in other analyses). Moreover, we chose CNN over other algorithms due to its ease of implementation. While a more careful selection of machine learning algorithms could have improved prediction accuracy, model performance was not a priority, as our goal was simply to establish a statistically significant relationship between STR flanking sequences and STR sequencing accuracy. Once this relationship was confirmed, all subsequent analyses involving STR flanking sequences were done without machine learning. We hope the reviewer accept our answers.

3-3) *Also, would be good idea to brief why the authors choose to work on T2T over hg38. I know that T2T have repetitive sequences that hg38 but little more basics would be a good read.*

We appreciate the suggestion to clarify our choice of reference genome. Our primary dataset is the ONT sequencing data of the CHM13 cell line provided by the T2T Consortium[3]. Therefore, using the T2T-CHM13 genome (specific to the CHM13 cell line) as our reference, rather than GRCh38, is essential, because the T2T-CHM13 genome serves as the ground truth for the ONT sequencing data. In our revised manuscript, we now mention this in our Introduction section in line 111-114.

---

[3] https://github.com/marbl/CHM13

4) *The results section is well-organized and presents the findings clearly. The figures are informative and support the conclusions drawn. However, some additional analyses could be performed to strengthen the results.*

We thank the reviewer for the positive feedback and valuable suggestions.

> 4-1) *For example, the authors could compare the performance of NanoMnT to other existing tools (such as NanoSTR (Lang et al., 2023), NanoRepeat (Fang et al., 2023)) on a larger dataset or the study such as using the benchmarking sets fro the recent study i.e. English, A.C., et al. (2024). Analysis and benchmarking of small and large genomic variants across tandem repeats.*
>
> Indeed, we recognize that this segment of our Results section (comparing the performance of tools) requires additional benchmarking analyses, and we thank the reviewer for helping us notice this and suggesting the paper by English, A. C., et al. Unfortunately, this benchmarking dataset by English et al. excludes homopolymers, which are the major type of STR addressed in our study. Nonetheless, to compensate for the insufficient benchmarking analysis, we doubled the number of loci used for benchmarking (1200 loci in total). We hope the reviewer accepts our answer.
>
> 4-2) *Additionally, the authors could investigate the impact of different sequencing parameters on the performance of NanoMnT.*
>
> We greatly thank the reviewer for raising an important point regarding the performance of NanoMnT. We ran NanoMnT in varying coverage (10x, 30x, 60x) and flowcell version to assess the impact of these factors in accurately assessing 1bp- and 2bp-repeat STR loci alleles.

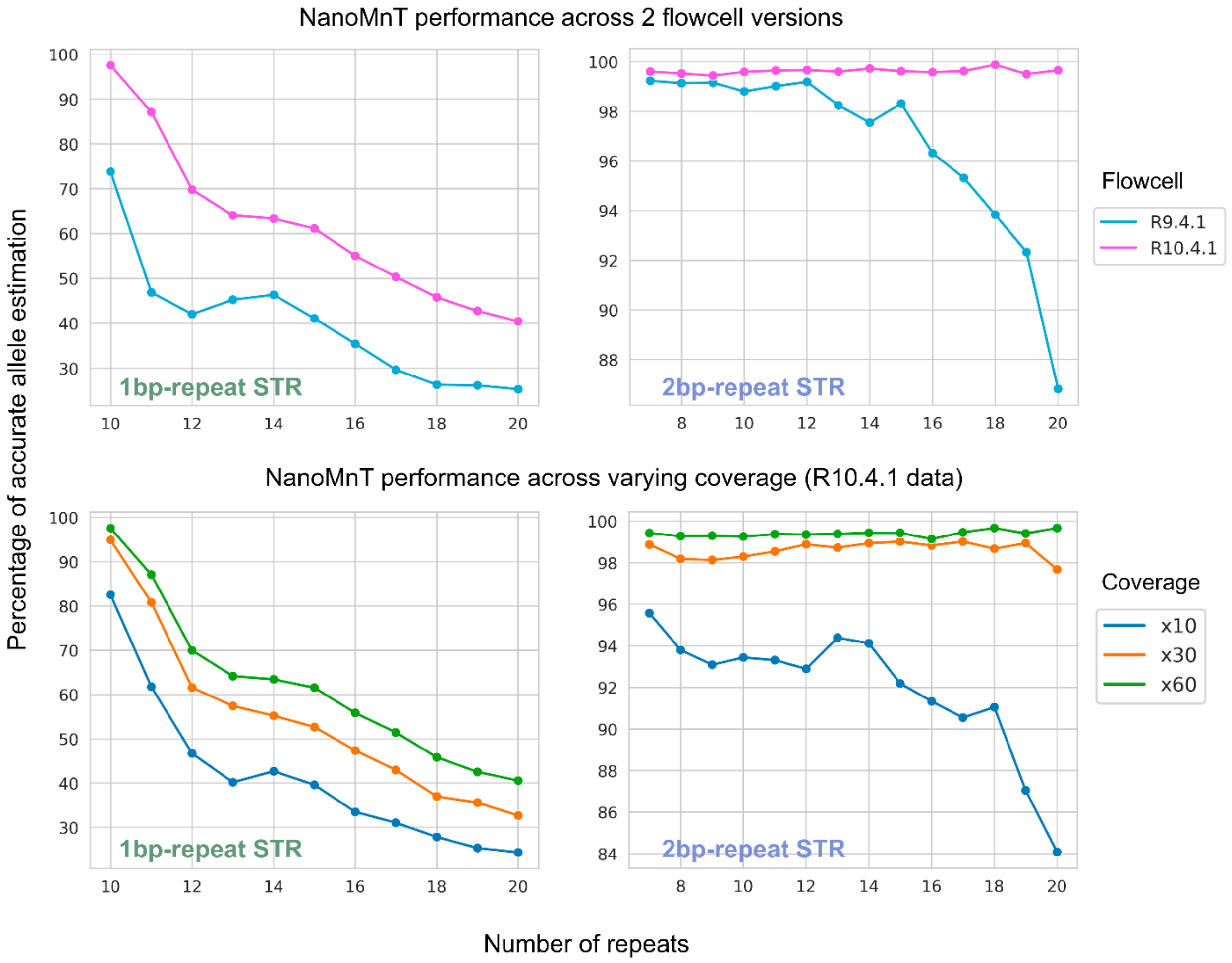

Impact of flowcell: Allele estimation with R10.4.1 data consistently outperformed R9.4.1, particularly in long 2bp-repeat STR loci (>16 repeats), demonstrating great improvements from the R9.4.1 flowcell.

Impact of coverage (R10.4.1): For 1bp-repeat STR loci, NanoMnT's accuracy improved significantly with higher coverage, especially for longer loci. In contrast, for 2bp-repeat STR loci, coverage levels of 30x and 60x yielded similarly optimal results, maintaining accuracy even as repeat lengths increased. However, at 10x coverage, allele estimation accuracy dropped substantially, with performance deteriorating further for longer 2bp-repeat STR loci.

Recognizing the importance of this information, these results are now added in the revised manuscript as an additional main Figure (Figure 9).

5) *The discussion is well written, but the results are not discussed properly, must be revised in-depth analysis it with acknowledge the limitations of the study and suggest future directions for research.*

In response to the reviewer's comment, we thoroughly improved our Discussion section: summaries of our analyses have been written more comprehensively, and the limitations of our study have been further explored. We thank the reviewer for the feedback.

6) The overall the manuscript is good, but there are places where clarity and conciseness are needed. For example, in the introduction, the authors state, "STRs are also known to play important roles in the pathogenesis of numerous diseases." This sentence should be made more specific, with examples of the diseases.

We are grateful to the reviewer for the insightful and positive feedback. To enhance conciseness, we now briefly explain the major pathomechanisms of repeat expansions (transcriptional repression, gain-of-function of repeat-containing proteins, etc.) and why MSI cancers show favorable outcome in our revised manuscript (line 57-60).

Reviewer 3

We appreciate the reviewer’s attention to detail, which has been critical in improving our manuscript in both contents and clarity.

Introduction

1) *Author states "much previous research has shown that STRs..." Only a single reference was provided here, which is insufficient to count as "much research". References are needed to back many claims made in the introduction. A few I noted: 1. NGS has expanded the number of STRs that can be analyzed, 2. WGS can enable analysis of most STR regions, 3. long read sequencing is advantageous for characterization of STR regions compared to short read sequencing (especially given the additional coverage and accuracy provided by MPS), 4. ONT provides superior throughput cost, and 5. ONT has particularly high error rates in low-complexity regions.*

We thank the reviewer for the valuable comment. We agree that more references are needed to support our claims. In our revised manuscript, nearly all sentences in the Introduction section are now supported by appropriate reference(s).

2) *ONT accuracy has increased significantly since their introduction, especially with the R14 chemistries and flow cells, but this paper still treats it like it is the same system that it was a decade ago.*

We appreciate the reviewer’s comment, which prompted us to revise our Introduction section to address ONT’s advancements in chemistry and flow cell technology. Indeed, ONT has made significant improvements in recent years, particularly with the V14 kit (SQK-LSK114). We apologize for the oversight in our initial manuscript.

In the revised version of our manuscript, we now discuss these advancements, including the change from the R9 to the R10 flowcell and the V14 chemistry. Please see line 86-91 for details. Additionally, we have explicitly mentioned that the dataset used to explore the STR sequencing error profile on the R10.4.1 flow cell— that is, the HG002 dataset—was generated with the V14 kit (SQK-LSK114), allowing us to analyze the STR error profile of the most up to date ONT data.

3) *Is the paper saying Nanopore is no longer supported as of 2018? I do not believe so, as NanoSTR was published in 2023, and their github website was updated as recently as 10 months ago. I think they are referring to Porechop, but this is not clear from the sentence, especially as the next sentence opens with "On the other hand, while Nanorepeat is regularly updated...”*

We would like to clarify that what we meant to say is that Porechop is no longer maintained as of 2018. We apologize if our manuscript was unclear in this context. We have changed the following sentence to enhance clarity (line 102-105):

> **Initial manuscript**: NanoSTR relies on Porechop, a bioinformatic tool that performs adapter trimming for ONT reads, which require the entire FASTQ file to be loaded onto the memory (RAM) and is no longer maintained as of October 2018.
>
> **Revised manuscript**: In contrast, while sequence-based tools are more versatile in this aspect, many rely on discontinued dependencies. For instance, NanoSTR (Lang et al., 2023) requires Porechop (RRID:SCR_016967), which was officially discontinued as of October 2018, and PacmonSTR (Ummat & Bashir, 2014) requires BLASR (RRID:SCR_000764), which is also no longer maintained.

Methods

1) *One of the major conclusions drawn is that flanking regions are essential for accurate sequencing. However, the authors eliminated any STRs with low complexity in their flanking regions. An assessment of even a small subset of these loci would go a long way in supporting the conclusions about flank importance. This is also valuable for a tool designed to evaluate STRs, particularly low complexity STRs. It would help to demonstrate the limitations of the software, which cannot be done if these data are removed prior to analysis due to the assumption of a limitation.*

We thank the reviewer for raising an important point regarding the flanking sequences.

There are two major reasons why we decided to exclude such STR regions:

(1) One is to rule out the possibility of flanking sequences distorting our measurement of error profile in STR regions. For example, if the flanking sequences are extremely repetitive, they may play a major factor in increasing the sequencing error rate in the STR regions which they flank.
(2) Another is to simplify the process of accurately determining the STR allele for each read. Consider the following Ax12 STR locus as an example:
    - ⋯ AAAAAAAAAAAC $(A)_{12}$ GCAATCCATACT ⋯

    The left flanking sequences exhibit extremely low sequence-complexity and are nearly identical to the STR sequences. When aligning ONT reads, the (left) flanking sequences of the reads align very ambiguously. Below are two IGV screenshots of ONT reads from the CHM13 dataset that aligned to this particular locus (upper), and another locus that is flanked by sequences with higher sequence-complexity (below). The STR locus of interest is specified by a horizontal bold red line. The blue dashed box

represents the error profile which we aim to analyze, and the red dashed box represents a portion of the alignment that confounds our analysis, because such alignments make allele measurement very difficult.

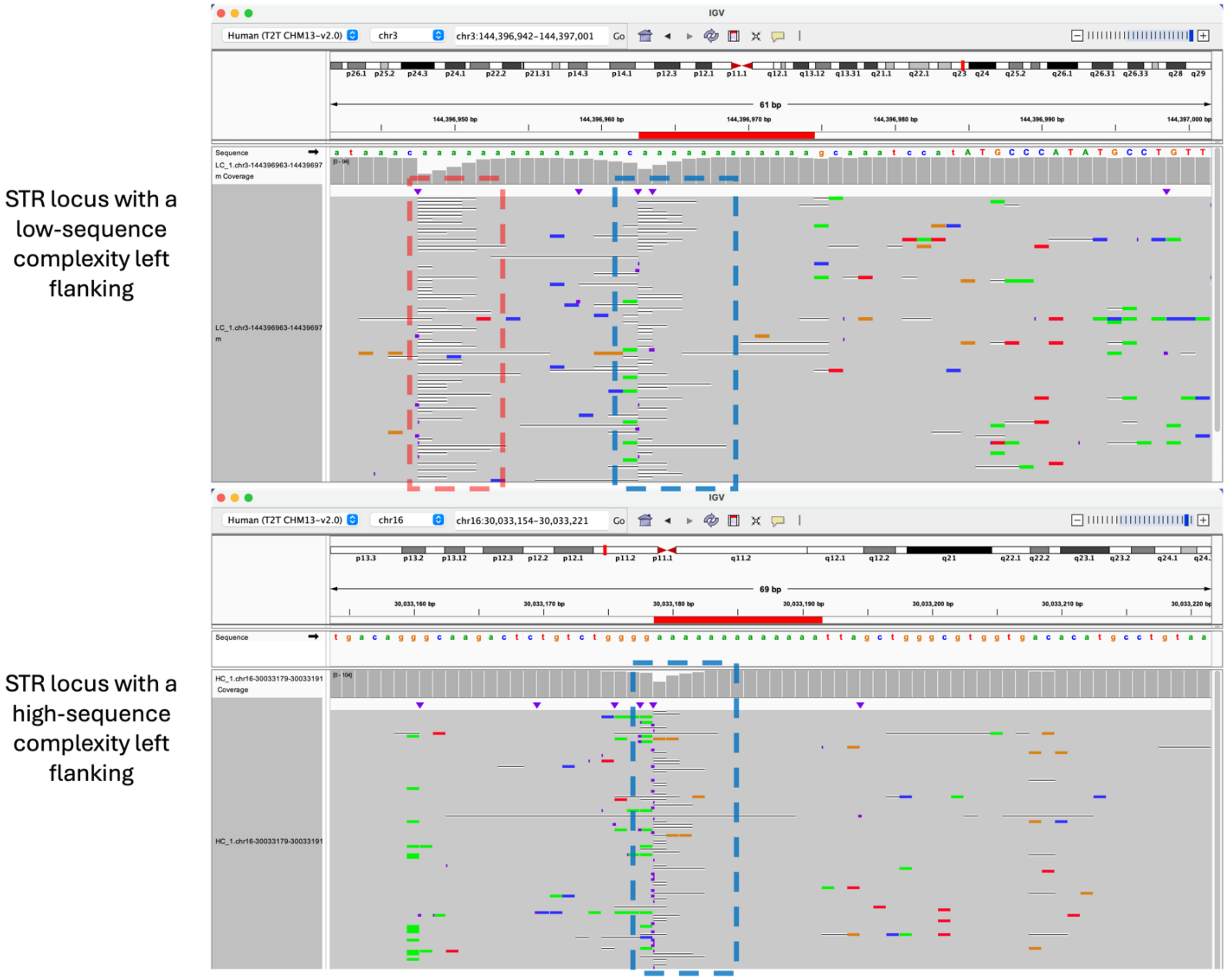

Nonetheless, the reviewer's suggestion has inspired us to further analyze a subset of the previously excluded STR regions, whose flanking sequences of 12 bp satisfy the following 2 conditions, which helps prevent awkward alignment of reads.

~~(1) Sequence complexity (calculated using the same method presented in our Methods section) of the left flanking sequence ≤ 2.5.~~

~~(2) Sequence complexity of the right flanking sequence ≤ 2.5.~~

(1) Levenshtein distance between the left flanking sequence (12 bp) and the STR sequence > 6. Using the locus shown above as example, this value would be 1.

(2) Levenshtein distance between the right flanking sequence (12bp) and the STR sequence > 6. Likewise, using the locus shown above as example, this value would be 8.

In our main analysis, we have identified top 20 and worst 20 flanking sequences motifs of A-repeat STR that are associated with good and poor sequencing accuracy, respectively (Figure 4b of our main manuscript). We first checked if these motifs retain their association in low-complexity (LC) A-repeat loci as well (Figure **a**, below). Although a considerable portion of entries were missing, we were able to confirm the association even in LC A-repeat loci. In addition, most of these loci showed their association regardless of the length of A-repeat, which further supports our hypothesis (Figure **b**). Finally, we counted the number of purine bases in these motifs and obtained results very similar to those presented in Supplementary Figure 7b. In summary, analysis using the previously excluded loci gave results very similar to those of our original analyses, strengthening our conclusion regarding flanking sequences' impact on sequencing accuracy. This supplementary analysis is now incorporated into our manuscript (Supplementary Figure 8), and we greatly thank the reviewer for suggesting the idea.

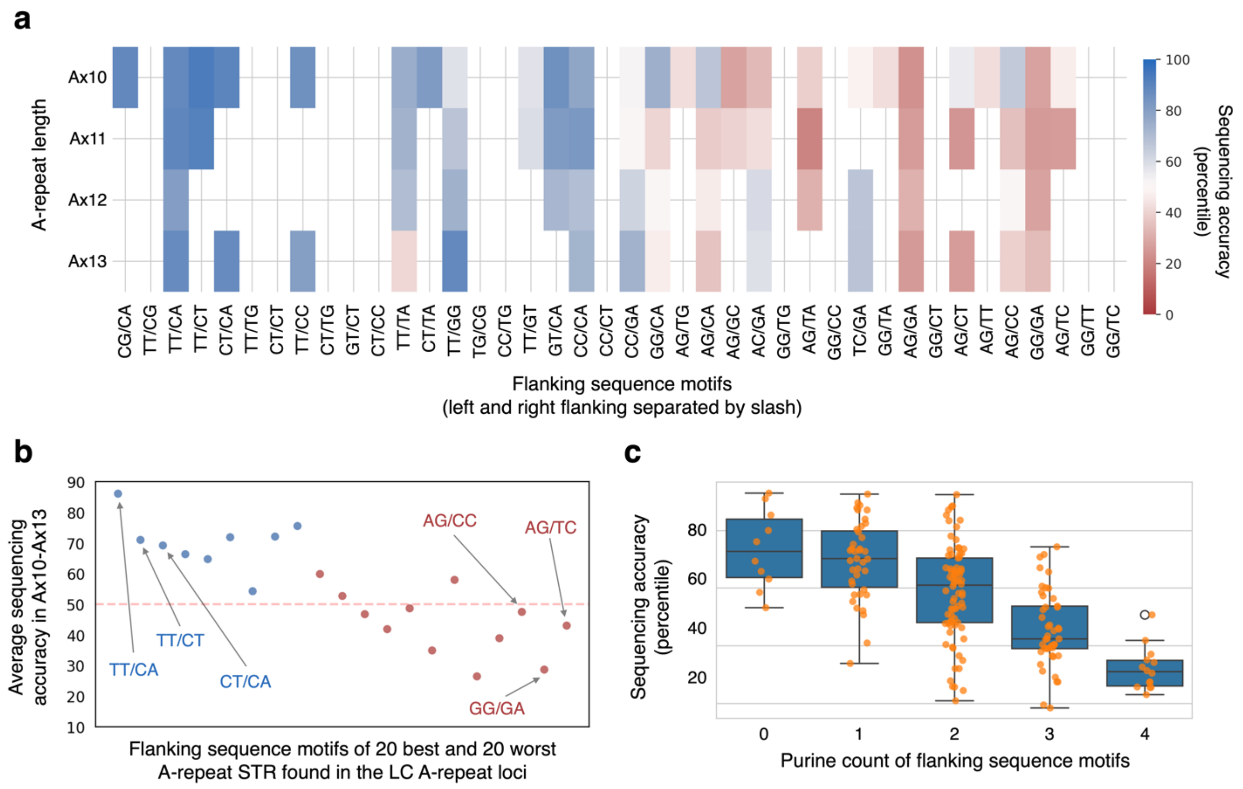

2) *It is unclear why the coverage requirements and loci assessed were different between the T2t-CHM13 dataset and the HG002 dataset.*

We appreciate the reviewer's attention to details. (1) The coverage requirements set in our study were different simply due to the coverage of each dataset. The coverage of the CHM13 dataset is ~120x[4], while the coverage of the HG002 R9.4.1 dataset and the HG002 R10.4.1 dataset was ~55x and 60x, respectively. Applying the same threshold to all datasets would indeed deliver more comparable results; however, it would disproportionately penalize the lower-coverage HG002 datasets. In our revised manuscript, we now mention this in our Methods section to enhance clarity. We thank the reviewer for pointing this out. (2) The difference in the assessed loci is due to the difference in the reference genome. When analyzing the reads from the CHM13 dataset, the T2T-CHM13v2.0 genome was used as the ground-truth, whereas when analyzing the HG002 dataset, the HG002 assembly (maternal genome)[5] was used as ground-truth. The different usage of reference genomes results in different loci being assessed. While this could be partially mitigated by the application of genomic coordinate conversion tools (e.g., LiftOff), a considerable number of STR loci are usually not well converted, resulting in a substantial exclusion of STR loci that can be analyzed. Because the primary goal of this study is to analyze the error profile of ONT, we did not find the conversion step to be essential. We hope the reviewer accepts our answer.

2-1) *I would presume that ultimate use of an STR analysis software would be working on samples with unknown profiles/background. What would be the required depth of coverage for accuracy calls?*

We greatly thank the reviewer for highlighting this important consideration, particularly from the user viewpoint. We have conducted additional NanoMnT benchmarks, evaluating the influence of sequencing coverage (x10, x30, x60) and flowcell (R9.4.1 versus R10.4.1) on the accuracy of NanoMnT allele estimation.

---

[4] https://github.com/marbl/CHM13
[5] https://github.com/marbl/HG002

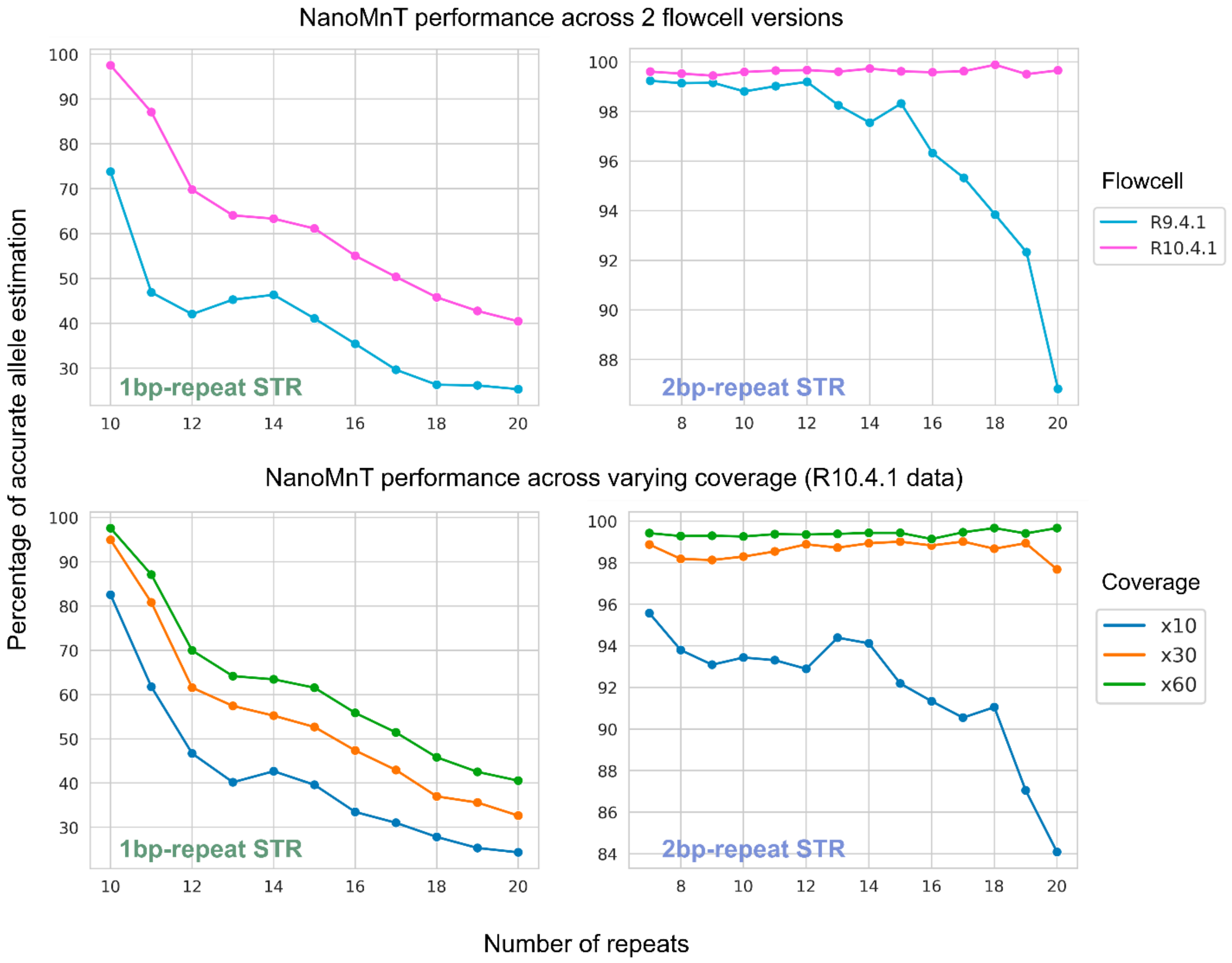

Impact of flowcell: Allele estimation with R10.4.1 data consistently outperformed R9.4.1, particularly in long 2bp-repeat STR loci (>16 repeats), demonstrating great improvements from the R9.4.1 flowcell.

Impact of coverage (R10.4.1): For 1bp-repeat STR loci, NanoMnT's accuracy improved significantly with higher coverage, especially for longer loci. In contrast, for 2bp-repeat STR loci, coverage levels of 30x and 60x yielded similarly optimal results, maintaining accuracy even as repeat lengths increased. However, at 10x coverage, allele estimation accuracy dropped substantially, with performance deteriorating further for longer 2bp-repeat STR loci.

Recognizing the importance of this information, these results are now added in the revised manuscript as an additional main Figure (Figure 9).

2-2) *Also, while I understand the loci assessment was different due to variations in heterozygosity, what if future users are wanting to evaluate unknown samples without any knowledge of the degree of heterozygosity?*

Certainly, the degree of heterozygosity is generally expected to be unknown in most practical settings. Unfortunately, this is not an issue that NanoMnT can robustly resolve, especially for mononucleotide repeats longer than 12 repeats. We believe the error rate is simply too high to resolve the heterozygosity by applying algorithms such as Gaussian mixture models.

Nonetheless, users can inspect (1) the peak prominence (which is expected to be inversely correlated to heterozygosity), (2) the number of identified peaks, and (3) perhaps most importantly, the allele size histograms of loci of interest if they suspect polyallelic signals. In our revised manuscript, we now acknowledge this as a major limitation of NanoMnT and explain that other tools (e.g., NanoRepeat) are better suited for this aspect (line 487-491). This will also be mentioned in the Caveat section in our NanoMnT GitHub page. We thank the reviewer for pointing out such important point.

2-3) *Are there guidelines for standard STR loci that are safe to be evaluated with these methods and software? A standard approach to all datasets, with conclusions being drawn about what would be considered acceptable results/loci (depth of coverage, heterozygosity, etc) that one could expect reliable results from would be more valuable than a presumptive screening based on knowledge of the dataset itself. How will future users know how to replicate these methods on novel dataset?*

We thank the reviewer for raising this important question. We believe that the newly added Figure 9 can help provide guidelines for users. Importantly, the standard criteria for determining the acceptability of results differs quite significantly depending on the flowcell and coverage. While attempting to exactly determine the STR alleles is not recommended for 1bp-repeats (except perhaps Ax10/Tx10), doing so for 2bp-repeats (using R10.4.1 flowcell) and repeats with longer repeat units are viable if coverage is sufficient, ideally above 30.

In other words, accurate determination of STR allele should be limited to short 1bp-repeats ($\leq$10 bp) is possible for all other repeats shown in this study. For all other purposes (i.e., crude estimation of STR alleles, which includes MSI detection), we believe all STR types are viable.

Furthermore, on the GitHub page of NanoMnT, we now provide a user-friendly tutorial for detecting MSI status of cancer ONT sequencing data. The tutorial

contains legends that help the user understand the process step-by-step. We appreciate the reviewer's inquiry regarding the usage of NanoMnT.

3) *Did the original alignment first include alignment to the reference genome? Given the simplicity of the loci being assessed, how did the authors account for/ensure elimination of off-target alignments, as these could result in an increase in observed errors.*

Yes, the original alignments were made to the reference genome. To minimize the impact of off-target alignments, we only used primary alignments with mapping quality (MAPQ) value of 60, which is the maximum score given by minimap2. Nonetheless, the inquiry by the reviewer motivated us to double-check whether our 'MAPQ filter step' would be enough to account for the elimination of misalignments.

We extracted reads (of the CHM13 dataset) that aligned to chromosome 21 and realigned them to the T2T-CHM13v2.0 genome. We observed that ~85% of reads aligned to chromosome 21, and that the distribution of MAPQ of the reads that aligned elsewhere (the other ~15%) was significantly poor, with most approaching to 0. Moreover, ~91% of reads aligned only once. These results suggest that our strategy effectively eliminates most misalignments. We presume the absence (or minimal presence) of misalignments is due to the sheer length of the read. This misalignment-check analysis is now included in our revised manuscript (Supplementary Figure 1). We thank the reviewer for bringing this up.

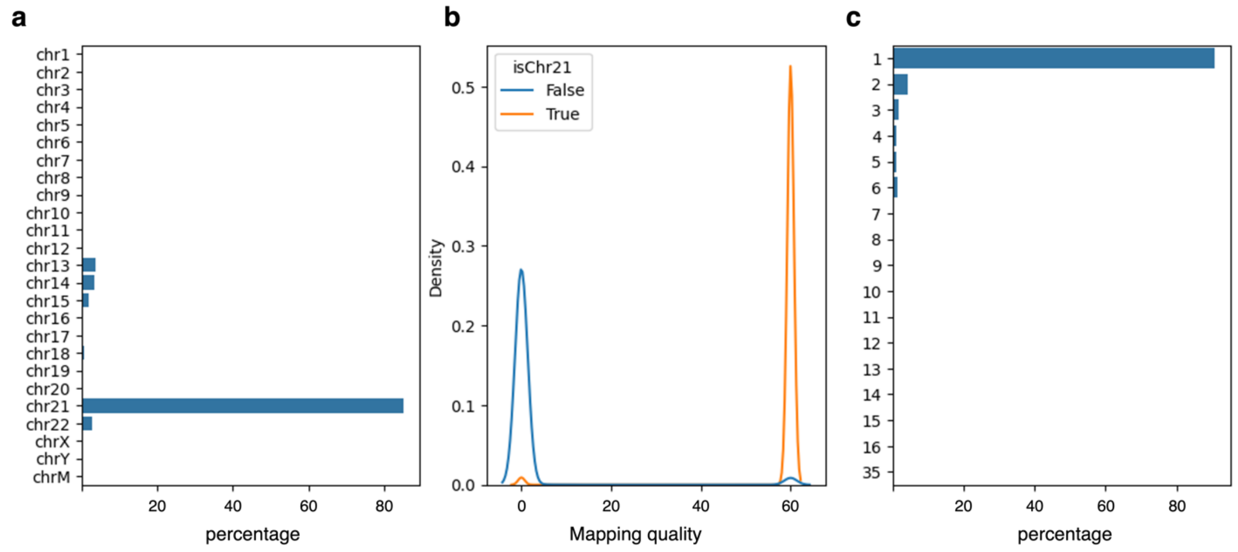

Results

1) *Sequencing error rates were calculated by counting the number of error-less reads. Accuracy between forward and reverse strands was also compared, which was a nice touch by the authors.*

We appreciate the positive comments made by the reviewer.

2) *It would help if the authors reported raw numbers in addition to percentages. Error section does not include numbers for types of errors at all.*

In our revised manuscript, we now explicitly provide raw number of reads and loci.

3) *While sequencing errors across the board are interesting to share, most analyses will evaluate a consensus sequence. It is unclear, so I could be wrong, but it does not appear the authors evaluated the consensus to see if the overall call included any errors. Such an analysis would help to allow this platform to be compared to other published studies, and give an overall assessment of the reliability of final results using this program.*

Thank you for your feedback and the opportunity to clarify our analysis. The ONT error profile was indeed evaluated at the raw read level to ensure the analysis was as direct and unprocessed as possible. However, the evaluation of NanoMnT and other tools (e.g., NanoRepeat and NanoSTR) was performed using the **final consensus allele calls**, which represent the repeat counts derived from all reads. This approach aligns with the standard output of STR analysis tools, which typically determine the consensus allele value (an integer rather than a string sequence) for their final predictions.

The performance of these tools, including NanoMnT, is shown in Figures 8, 9 and Supplementary Figures 15, where the consensus allele calls were used to measure accuracy. While we did not specifically evaluate a reconstructed **sequence-level consensus**, the focus of this study was on allele-level predictions, which are more relevant for STR analysis. We hope this explanation clarifies the scope of our analysis and aligns with your expectations.

4) *The applications of NanoMnT to cancer screening are valuable and well laid out.*

We greatly appreciate the reviewer’s positive comment.

Discussion

1) *Were any motifs identified that were more likely to result in Indels?*

Given that indels account for over 90% of sequencing errors in mononucleotide repeats, it is safe to say that the ‘bad’ motifs identified in Figure 4 – characterized by high purine counts in the case of A-repeat STR – are likely to result in indel errors.

2) *Typo: "The excess number of insertions and substitutions..." but authors said most of the errors were deletions.*

Apologies for the oversight, this sentence is indeed contradictory and does not contribute to the Discussion section. We removed this sentence.

3) *More details about what could have caused the differences in those STRs that responded well the the SUP basecaller vs those that performed better using the HAC would be beneficial.*

Yes, the observation that some loci are better basecalled using HAC model is indeed puzzling. In hopes of answering this, we have repeated the UMAP analysis for these loci but to no avail.

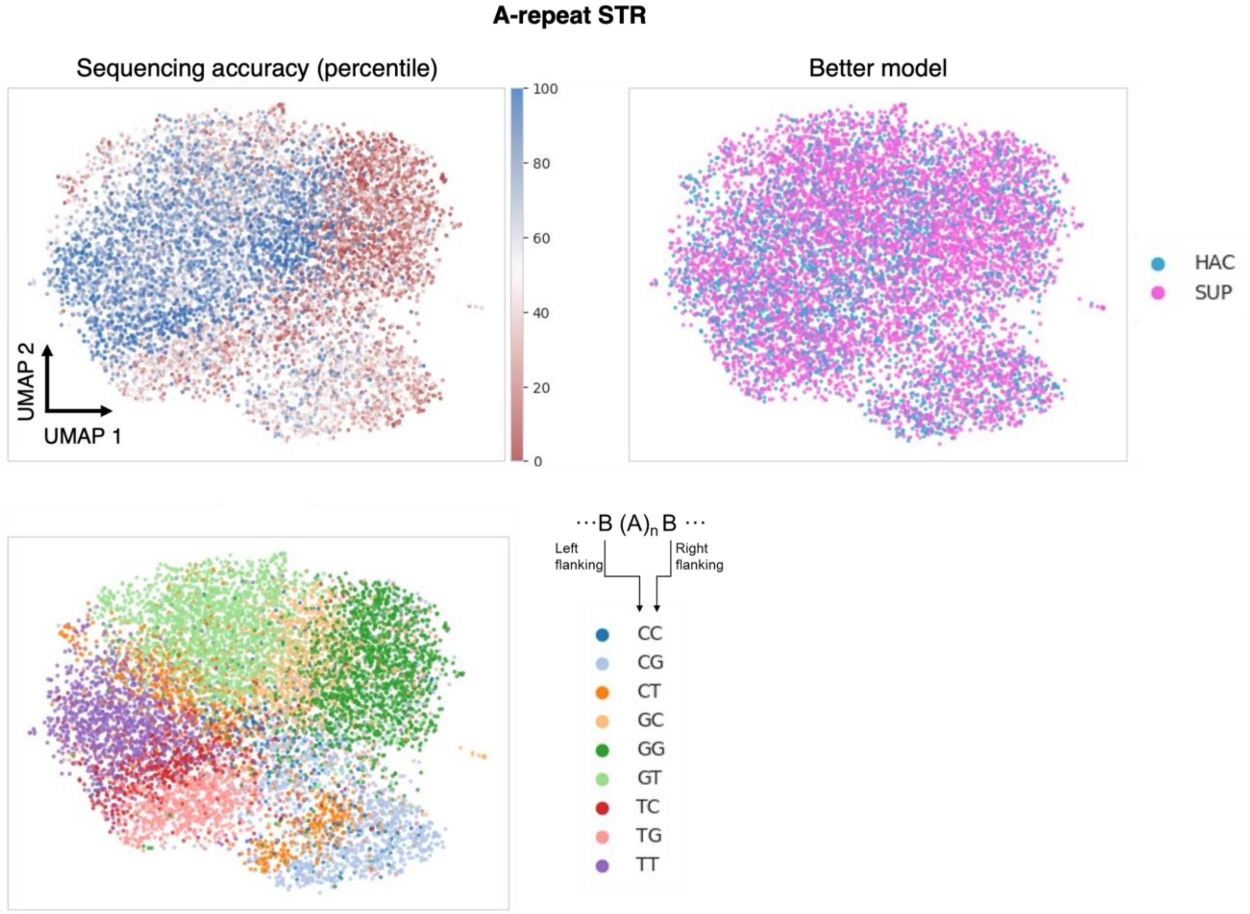

Flanking sequences does not seem to be associated with this phenomenon. Although speculative, ONT sequencing error profile may simply be too stochastic, especially for STR regions. In our revised manuscript, this result is now included (Supplementary Figure 9).

4) *The authors acknowledged the shortcomings of this study, including the exclusion of many imperfect STRs.*

.

Language

The manuscript could use heavy editing to improve grammar.

We thank the reviewer for bringing this to our attention. We have conducted a thorough review of the initial manuscript to improve its clarity and grammar. Redundant sentences were identified and removed, and several paragraphs with awkward order of contents were restructured. Finally, we have received academic editing services to further polish our manuscript.

Figures/Tables:

-The gradient effects on Figure 1 are negligible and would not be noticed if it were not for the legend.

Apologies, we have now adjusted the degree of gradient effects.

Manuscript

Click here to access/download;Manuscript;Revised_manuscript2_fontAdjuste

NanoMnT: An STR Analysis Tool for Oxford Nanopore Sequencing Data Driven by a Comprehensive Analysis of Error Profile in STR regions.

Gyumin Park[1], Hyunsu An[1], Han Luo[2]*, Jihwan Park[1]*

[1] School of Life Sciences, Gwangju Institute of Science and Technology (GIST), Republic of Korea.

[2] Department of Thyroid and Parathyroid Surgery, Laboratory of thyroid and parathyroid disease, Frontiers Science Center for Disease-related Molecular Network, West China Hospital, Sichuan University, Chengdu, Sichuan, China

* Correspondence:

Han Luo

Department of Thyroid and Parathyroid Surgery, Laboratory of Thyroid and Parathyroid Disease, Frontiers Science Center for Disease-related Molecular Network, West China Hospital, Sichuan University, Chengdu, Sichuan, China.

No. 37 Guoxue Alley, Chengdu, Sichuan, China, 610041

Phone: +86-18980605139

Fax: +86-28-85422467

Email: luohan-hx@scu.edu.cn

Jihwan Park

School of Life Sciences, Gwangju Institute of Science and Technology

123 Cheomdangwagi-ro, Buk-gu, Gwangju, Republic of Korea, 61005

Phone: +82-627152522

Fax: +82-627152484

Email: jihwan.park@gist.ac.kr

Keywords: Oxford Nanopore; Long-read sequencing; Short tandem repeats; Microsatellite; Error profile; Bioinformatics.

Abstract

Oxford Nanopore sequencing (ONT) is a third-generation sequencing technology that enables cost-effective long-read sequencing, with broad applications in biological research. However, its high sequencing error rate in low-complexity regions hampers its applications in short tandem repeat (STR) related research. To address this, we generated a comprehensive STR error profile of ONT by analyzing publicly available Nanopore sequencing datasets. We show that the sequencing error rate is influenced not only by STR length but also by the repeat unit and the flanking sequences of STR regions. Interestingly, certain flanking sequences were associated with higher sequencing accuracy, suggesting that certain STR loci are more suitable for Nanopore sequencing compared to other loci. While base quality scores of substitution errors within the STR regions were lower than those of correctly sequenced bases, such patterns were not observed for indel errors. Furthermore, choosing the most recent basecaller version and using the super accuracy (SUP) model significantly improved STR sequencing accuracy. Finally, we present NanoMnT, a lightweight Python tool that corrects STR sequencing errors in sequencing data and estimates STR allele sizes. NanoMnT leverages the characteristics of ONT when estimating STR allele size and exhibits superior results for 1bp- and 2bp-repeat STR compared to existing tools. By integrating our findings, we improved STR allele estimation accuracy for Ax10 repeats from 55% to 78%, and up to 85% when excluding loci with unfavorable flanking sequences. Using NanoMnT, we present the utility of our findings by identifying microsatellite instability (MSI) status in cancer sequencing data. NanoMnT is publicly available at https://github.com/18parkky/NanoMnT.

Introduction

Short tandem repeats (STRs), also known as microsatellites, are regions of DNA consisting of repeated sequence of 1-6 base pairs (bp) [1] and are routinely employed as 'genetic fingerprints' in a variety of fields, such as forensics [2] and population genetics [3]. Previous research has shown that STRs are also involved in gene regulation by modulating DNA methylation and providing binding sites for transcription factors [4-6]. Moreover, owing to their high mutation rate [7], STRs play critical roles in the pathogenesis of numerous degenerative diseases, including Fragile X syndrome, spinal and bulbar muscular atrophy (SBMA), and Huntington disease [8]. The major pathomechanisms of repeat expansions are diverse: loss-of-function via transcriptional repression, gain-of-function of canonically translated repeat-

containing proteins, and RNA-mediated gelation and sequestration of RNA-binding proteins, reflecting the functional importance of STR [8]. In addition, the genomic instability of STRs in mismatch repair-deficient (dMMR) cancers manifests as a molecular phenotype known as microsatellite instability (MSI), which is generally associated with a favorable response to cancer immunotherapy due to abundant generation of neo-antigens [9, 10]. The allele sizes of STRs, defined by the number of repeats, are crucial for understanding their roles in these biological contexts. Consequently, accurate quantification of STR allele size is essential for assessing their functional roles and advancing our understanding of their contributions to health and disease.

Although conventional PCR-based methods for analyzing STRs have proven effective, they are limited by the number of STR regions that can be analyzed simultaneously [11]. However, advancements in next-generation sequencing (NGS) have significantly expanded the number of analyzable STR regions, to the point where whole-genome sequencing (WGS) with sufficient sequencing depth can enable the analysis of most STR regions [12]. Long-read sequencing technologies are generally more advantageous than short-read sequencing for characterizing STR regions because they can span not only the STR sequences, but also their flanking sequences. This capability enables robust read alignments of STR regions, which is particularly valuable for analyzing STR with complex structures, such as compound and imperfect STRs [13]. In contrast, short-read sequencing often fails to sequence the flanking sequences of STR regions, which may result in producing misalignments. Furthermore, the lengths of some STR alleles exceed the read lengths of short-read sequencing, requiring longer reads for accurate STR allele identification [14, 15].

PacBio sequencing and Oxford Nanopore sequencing (also referred to as Oxford Nanopore Technologies or ONT) are two of the most widely used long-read sequencing technologies, with extensive applications in various research fields [16, 17]. Each technology has distinct advantages. PacBio HiFi sequencing achieves impressively high sequencing accuracy (99.8%, >Q30), comparable to that of Illumina sequencing (99.9%) and provides an average read length of 13.5 kb (although this varies depending on the fragmentation step) [18]. In contrast, ONT enables real-time analysis, portability, cost-effectiveness, and ‘ultra-long’ reads exceeding 100 kb [19]. However, many studies have reported that ONT exhibits higher sequencing error rates than other sequencing technologies [20, 21].

A previous comprehensive analysis reported that approximately half of all sequencing errors occur in STR regions [22], which complicates downstream analyses that require accurate assessments of STR lengths (i.e., number of repeats). For example, indel errors in homopolymer regions can critically affect gene calling and protein prediction by introducing false frameshift events in coding sequences [23]. In an effort to improve sequencing accuracy, ONT has progressively updated flowcells, sequencing kits and basecalling programs. One of the most notable improvements was seen after upgrading from the R9.4.1 flowcell (read accuracy Q17) to the R10.4.1 flowcell (read accuracy Q21), which greatly

reduced the number of errors [24, 25]. Furthermore, the recent introduction of the ligation sequencing kit V14, designed to achieve single-pass modal accuracy of >Q20, has further improved ONT sequencing accuracy [25]. Nevertheless, the high sequencing error rate of ONT in low-complexity regions remains a significant challenge, and a comprehensive study of ONT error profiles in STR regions is still lacking.

Despite these challenges, numerous bioinformatic tools have been developed to analyze STR regions from ONT data. These tools can be grouped into two categories based on the type of input file: electric signal-based tools, which analyze STR directly from the raw electric current data (e.g., FAST5 or POD5 files), and sequence-based tools, which analyze STR from the basecalled data (e.g., FASTQ or BAM files) [26]. Although each group of tools has been successfully applied to its respective areas of interest, many are either no longer maintained or have certain limitations. For example, electric signal-based tools such as WarpSTR [27], DeepRepeat [28], STRique [29] and NanoSatellite [30] require FAST5 files, which are often unavailable in public datasets. Additionally, many electric signal-based tools are incompatible with the newly introduced POD5 format, which is the current output format of ONT. In contrast, while sequence-based tools are more versatile in this aspect, many rely on discontinued dependencies. For instance, NanoSTR [31] requires Porechop (RRID:SCR_016967), which was officially discontinued as of October 2018, and PacmonSTR [32] requires BLASR (RRID:SCR_000764), which is also no longer maintained. Furthermore, many sequence-based tools do not support multi-threading, severely limiting their scalability. Notably, none of these tools, including well-maintained tools such as Straglr [33], NanoRepeat [34] and tandem-genotypes [35] have been tested for estimating mononucleotide repeats, which are the most error-prone regions in ONT [22].

In this study, we performed a comprehensive analysis of ONT error profiles in STR regions using 3 publicly available ONT sequencing datasets (Supplementary Table 1). We centered our analysis on the T2T-CHM13 dataset (generated using R9.4.1 flowcells) because of its high sequencing depth (~120x) and the availability of the T2T-CHM13 reference genome which serves as the ground truth. The near homozygosity of the CHM13 cell line removes the need to consider biallelic STR signals, enabling a straightforward analysis. To validate our findings and translate them to the current R10.4.1 flowcell version, we incorporated 2 additional datasets of the HG002 genome, one generated using the R9.4.1 flowcell and another using the R10.4.1 flowcell, both publicly available through EPI2ME. These HG002 datasets are referred to as “HG002 R9.4.1 dataset” and “HG002 R10.4.1 dataset” throughout the article. The HG002 R10.4.1 dataset was generated using the V14 kit (SQK-LSK114), allowing us to explore the error profile of the most up-to-date ONT configuration. Unless otherwise specified, (1) analyses of the R9.4.1 STR error profile were performed using the CHM13 dataset and (2) the R9.4.1 data presented in this article were basecalled with the Guppy v6.5.7 high-accuracy (HAC) model, whereas the R10.4.1 data were basecalled with the Dorado v8.1.0 HAC model. Finally, we present NanoMnT (RRID: SCR_026210), a lightweight Python-based tool that performs error-correction for ONT reads in STR regions

and estimates STR allele size. We demonstrate the utility of our findings by identifying MSI status of 4 cancer cell lines from The Singapore Nanopore Expression Project (SG-NEx) dataset [36] and 15 colorectal cancer (CRC) organoids [37] using NanoMnT.

Methods

Identification of STR regions

We employed Krait (v1.3.3, default settings, except the minimum repeat length requirement for 1bp-repeat STRs has been lowered to 10 bp) [38], an ultrafast bioinformatic program designed to identify STRs from genomes via brute force search algorithm 2 described by Sokol et al (http://archive.dimacs.rutgers.edu/Publications/Modules/Module09-2/dimacs09-2.pdf), to search for STR regions within the T2T-CHM13 (v2.0) genome and the HG002 (maternal genome. v1.0.1) genome.

1. T2T-CHM13

   Running Krait on the T2T-CHM13 genome (v2.0) resulted in an initial set of 2,103,586 STR regions. Because many 1bp-/2bp-/3bp-repeat STR regions were flanked by low-complex flanking sequences that closely resembled the STR sequences, 1bp-, 2bp-, and 3bp-repeat STR regions whose flanking sequences had excessively low k-mer diversity (see Calculation of k-mer diversity in Methods) were filtered out (k-mer ≤ 2.5 for 1bp-repeat, ≤ 2.0 for 2bp-repeat, ≤ 5.0 for 3bp-repeat), as they may introduce ambiguity when measuring STR repeat sizes. This resulted in 1,288,130 STR regions being left. Subsequently, regions with a read orientation-specific coverage of at least 20 (i.e., at least 20 forward strand reads, or 20 reverse strand reads) were selected, resulting in the final set of 762,311 STR regions.

2. HG002 (Maternal genome)

   First,1bp-, 2bp-, and 3bp-repeat STR regions were identified from the HG002 maternal assembly using Krait, resulting in an initial set of 1,316,436 STR regions. Unlike the CHM13 genome (which is haploid), the HG002 genome contains a considerable number of biallelic STR regions which complicate the analysis of ONT error profile. Therefore, we decided to exclusively use monoallelic STR regions in our analysis by employing LiftOff (v1.6.3, default parameters) to convert genomic coordinates from HG002 paternal assembly to HG002 maternal assembly [39]. During this process, 72,651 STR regions could not be converted, resulting in remaining 1,243,785 regions, of which 493,804 were confirmed to be monoallelic.

Finally, STR regions whose flanking sequences with low k-mer diversity (same thresholds applied to CHM13 STR regions) and regions with coverage lower than 5 (the coverage threshold was reduced to account for the lower coverage of the HG002 dataset compared to the CHM13 dataset) were filtered out, resulting in the final set of 186,237 STR regions.

Data processing and visualization

After downloading FAST5 files and POD5 files from sources specified by the authors, FASTQ files were obtained by employing the appropriate basecaller for each dataset (Supplementary Table 1). We aligned the FASTQ files to the reference genomes using minimap2 (v2.24-r1122) [40] with `-ax map-ont` parameters for all data except for SG-NEx data, where `-ax splice` was used instead. To reduce misalignments, we filtered out supplementary reads and reads with a mapping quality score below 60. We validated the effectiveness of this filtering step by re-aligning reads mapped to chromosome 21 back to the reference genome (Supplementary Fig. 1). Approximately 85% of the reads re-aligned to chromosome 21, while the remaining reads aligned elsewhere with markedly low mapping quality scores, close to 0. Filtering reads with mapping quality score below 60 resulted in 99.4% of accurately mapped reads. Subsequent data analysis and visualization were performed using Seaborn (0.13.0), Matplotlib (3.7.1), Pandas (2.0.0) and Numpy (1.22.4). All datasets analyzed in this study were PCR-free, ensuring the absence of PCR stutters. Moreover, all major datasets – CHM13 data, HG002 data and SG-NEx data– provided raw FAST5/POD5 files, allowing us to compare the influence of basecalling programs and their configurations on STR sequencing accuracy.

Calculation of k-mer diversity

First, the frequency of each k-mer within the given DNA sequence was counted. The counting process involved sliding a window across the DNA sequence by one nucleotide at a time, extracting all possible k-mers and storing their frequency in a dictionary data structure. For example, if the DNA sequence is ATCGC, the 2-mer counting process produces the following Python dictionary: {AT: 1, TC:1, CG:1, GC:1}.

Then, the k-mer diversity was calculated using the following expression:

$$k - mer\ diversity = \frac{L - (k-1)}{\sum_{i=1}^{L-1} F_i^2}$$

where L is the length of the given DNA sequence ($L-(k-1)$ equals to the maximum number of k-mer that can be found in the DNA sequence), and $\{F_1, F_2, F_3, F_4 \ldots\}$ represents the frequency of each found k-mer.

CNN prediction of sequencing accuracy using flanking sequences

The flanking sequences (6 nucleotides in each direction of STR, resulting in 12 nucleotides) were one-hot encoded and converted into a Numpy array. STR loci with coverage below 40 were discarded, and a training-validation ratio of 9:1 was used with the remaining loci. Briefly, we used TensorFlow [41] to implement a sequential neural network featuring a 1D convolutional layer with 48 filters and a kernel size of 2, followed by a flattening layer and two dense layers with 120 and 40 nodes, respectively, both using ReLU activation. The output layer consisted of a single node with a sigmoid activation function. The model used the Adam optimizer and mean absolute error (MAE) as the loss function. Training was performed with 20 epochs with a batch size of 400, with data shuffled at the start of each epoch, ensuring that the model encounters a random order of data. Finally, we enabled early stoppage by monitoring validation loss with a patience of 10 epochs, to prevent overfitting. Using this model, we predicted the sequencing accuracy of STR regions using the one-hot encoded flanking sequences as inputs. Linear regression was performed to assess the prediction results and visualized using Seaborn's `regplot` function, while Pearson correlation values were calculated using SciPy (1.7.1).

UMAP projection of STR regions

We considered a STR region's sequencing accuracy to be well predicted if it satisfied the following expression:

$$\mu - \frac{1}{2}\sigma \leq (predicted\ accuracy - actual\ accuracy) \leq \mu + \frac{1}{2}\sigma$$

where μ is the mean of differences between predicted and actual accuracies, and σ is the standard deviation these differences. The flanking sequences of these well-predicted STR regions were one-hot encoded and converted into an Anndata (v0.10.6) and subjected to UMAP visualization [42]. The following functions and parameters of Scanpy (1.10.0) were used: `sc.pp.neighbors(adata, n_neighbors=15, n_pcs=18)` and `sc.tl.umap(adata, spread=1)` [43].

Identification of 'top' and 'worst' 20 flanking sequences of A-repeat STR loci

Confirming the association of flanking sequences of A-repeat STR loci and sequencing accuracy through CNN prediction and UMAP visualization, we identified 'top' (i.e., demonstrating the best sequencing accuracy) 20 and 'worst' (i.e., demonstrating the worst sequencing accuracy) 20 flanking sequences of A-repeat STR (Ax10-Ax14; 2 nucleotides in each direction, totaling 4 nucleotides) by calculating the average sequencing accuracy of STR loci of each length, with different flanking sequences. To ensure that the impact of a given flanking sequence on the sequencing accuracy was consistent, we only included flanking sequences that were present in more than 10 A-repeats of every length. For example, although CG/CA flanking motif was found in more than 10 Ax10 repeats, it was not found in A-repeat of other lengths, and thus wasn't included in our analyses. The results were visualized using Seaborn's `heatmap` function.

Subset of A-repeat STRs that harbor low-complexity flankings

A subset of STRs that were previously excluded from the main analysis due to low-complexity flanking sequences (see Identification of STR regions in Methods) was reintroduced to validate the association between flanking sequence complexity and STR sequencing accuracy. Specifically, A-repeat STRs were selected if the Levenshtein distance between the STR sequence and either the left or right flanking sequence was below 7. For example, consider the following two A-repeat STR loci:

Loci A: 5' – AAAAAAAAAAAC (A)12 GCAATCCATACT – 3'

Loci B: 5' – TTTTTTTTCCCC (A)12 GCAATCCATACT – 3'

In Locus A, although the Levenshtein distance between the right flanking sequence and the STR sequence is 8, the distance for the left flanking sequence is 1, and therefore excluded. In contrast, Locus B has a Levenshtein distance of 12 between the left flanking sequence and the STR sequence, and therefore included.

Benchmarking NanoMnT, NanoRepeat, and NanoSTR

600 1bp-repeat STRs and 600 2bp-repeat STRs were incorporated. Unlike NanoRepeat and NanoMnT, NanoSTR requires the genomic coordinates of STR regions to be based on the hg38 assembly. Thus, we converted the genomic coordinates of STR regions from HG002 maternal assembly to the hg38 assembly using LiftOff. After filtering out loci that could not be confidently converted, 712 loci remained for 1bp-repeat STRs and 619 loci for 2bp-

repeat STRs. The STR loci used for benchmark are available in Supplementary Table 2. In-house scripts were used to summarize the outputs of each program.

MSI detection of cancer sequencing datasets

Ax10-Ax14 STR loci with coverage above 30 were used for MSI identification, as mononucleotide repeats of these lengths have been shown to be vulnerable to deletion mutations in MMR-deficient cells [44]. For the SG-NEx dataset, loci that were not covered in at least 3 out of 4 samples were discarded, while for the CRC organoid WGS dataset, loci that were not covered in at least 10 out of 15 samples were discarded. For the 'read + loci selection approach' shown in Figure 10a, we filtered out (1) A-repeat STR loci that had guanine nucleotides directly next to the A-repeat tracts, and (2) loci whose allele prominence (calculated by NanoMnT) satisfied the following expression:

$$\mu - \frac{1}{2}\sigma \leq allele\ prominence \leq \mu + \frac{1}{2}\sigma$$

where μ is the mean of allele prominences of all genotyped loci, and σ is the standard deviation these prominences.

Subsequently, we obtained the allele size histogram and calculated the relative allele size of each STR locus using the following expression:

$$\sum_{i=1}^{n}(A_i - R_i)f_i$$

where A_i is the observed allele, R_i is the reference allele (CHM13) and f_i is the frequency of A_i. The STR loci used for MSI detection are available in Supplementary Table 3 and 4.

Implementation of NanoMnT

NanoMnT provides three functions: (1) error-correction of reads, (2) STR allele size estimation, and (3) informative loci identification.

1. Error correction of individual reads

   NanoMnT collects reads that aligned to the user-provided STR loci using Pysam (v0.20.0) [45] and realigns them to a modified STR region that excludes the STR sequence itself, consisting only of the STR-flanking

regions. This approach prevents alignment bias caused by the reference genome, as minimap2 tends to produce slightly different alignments in STR regions, depending on the STR sequence length. For each realigned read, the sequences that aligned to the STR regions are extracted and compared against a list of possible alleles by calculating the Levenshtein distance. When defining the list of possible alleles (i.e., number of repeats), NanoMnT assumes that the allele for a given aligned read falls between 0 repeats (lower bound) and the longest allele among all the reads (determined by counting the number of repeats prior to error correction) plus 5 additional repeats (upper bound). The 5 additional repeats serve as a buffer to account for potential outliers. The allele with the minimum Levenshtein distance is chosen as the most likely allele. If the total Levenshtein distance exceeds a certain threshold, the read is considered excessively erroneous and discarded. This process yields corrected STR alleles for each ONT read, which are then used for subsequent STR allele size estimation.

2. Estimation of STR allele size

Using the corrected reads, NanoMnT creates an allele size histogram for each locus. The user can decide whether to use all reads or forward/reverse strand reads – which is very beneficial when analyzing A-/T-repeats – when creating allele size histogram. To estimate the STR allele size, NanoMnT generates synthetic allele size histograms for each possible allele and calculates the distance between the observed allele size histogram against each synthetic allele size histogram. The synthetic histogram with the minimum distance to the observed histogram is then chosen as the best match. The allele associated with this chosen histogram is selected as the most probable STR allele. Finally, SciPy's `find_peak` function is used to calculate the prominence of the observed allele size histogram.

3. Informative loci identification

Given the outputs of NanoMnT (Allele Table and Locus Table, see Figure 8a) of paired normal and tumor samples, NanoMnT finds STR loci that have been sequenced in both sample (namely, commonly covered loci) and compares the STR allele size histogram by calculating the distance between the two histograms. This distance information tells us about the similarity between the STR allele size histogram of two samples; if the similarity is low, this locus may be an indication of MSI phenotype. Lastly, the 'score' of each STR locus is calculated using the following expression: $locus\ score = distance(H_n, H_t) \times Peak\ prominenc\ of\ H_n$, where H_n and H_t is the allele size histogram of normal and tumor sample, respectively. This score informs the reliability of each result.

Results

Distribution of sequencing errors in STR regions

The STR regions analyzed in this study were carefully selected because many exhibited excessively low-complexity sequences in their flanking regions, which often introduce alignment bias and hampers downstream analyses (Methods). The number and distribution of STRs analyzed in this study are available in Supplementary Figure 2a. Note that GC-rich STR could not be robustly represented in our analyses due to the scarcity of GC-rich STR in the human genome (Supplementary Fig. 2b).

We first measured the abundance of each type of sequencing error (deletions, insertions, and substitutions) by counting the number of errors in ONT reads that aligned to the STR regions (Fig. 1a). Overall, the error rates were higher in STRs with shorter repeat units, with 1bp-repeats exhibiting the highest rate. Indel errors accounted for most of the sequencing errors (96%, 73%, 55% for 1bp-, 2bp-, 3bp-repeats, respectively). Among these, deletion errors were the most prevalent, particularly in 1bp-repeats, where 59% of reads (14,989,206 out of 25,392,291) contained at least one deletion, aligning with previous reports [20, 22, 24].

To provide a more practical analysis of STR error profile, we performed rudimentary polishing of sequencing errors using in-house scripts, as doing so considerably increased the number of reads that can be analyzed. This was achieved by calculating the Levenshtein distance between the observed STR sequence and a list of possible STR sequences, then selecting the STR sequence with the minimum distance. Reads with distances exceeding 4, which constituted approximately 2.8% of the total reads, were discarded. Using these polished reads, we visualized the distribution of STR allele sizes by generating histograms for various STR alleles. We observed that ONT tended to underestimate STR sizes, causing some histograms to shift slightly leftward (Fig. 1b, Supplementary Fig. 3). While many histograms exhibited clear peaks that matched the actual STR alleles, prominent peaks could not be generated for 1bp-repeats.

Sequencing accuracy of STR across different repeat units and lengths

Next, we measured ONT sequencing accuracy for different STR types. In this study, we define *sequencing accuracy* as the percentage of errorless reads (i.e., reads that do not contain any sequencing errors within the STR sequence), terms we will use interchangeably throughout the manuscript. When conducting this analysis, we made an important consideration. Given that only one strand of the double-stranded DNA enters the nanopore, the error profiles generated

by the two different orientations of reads are probably different. For example, the error profile of forward strand reads originating from an A-repeat STR locus (reads that map to the forward strand of the reference genome) may differ from that of reverse strand reads from the same locus (reads that map to the reverse strand of the reference genome). This is because the former set of reads encompasses the sequencing of A-repeats, while the latter encompasses the sequencing of T-repeats. Thus, we calculated the sequencing accuracy for each type of STR, based on their lengths and the repeat units that were actually sequenced by the nanopore. As a result, we found that the sequencing accuracy varied substantially among different types of STR (Fig. 2a). Among 1bp-repeats, A-repeats were generally better sequenced than other 1bp-repeats, whereas in 2bp-repeats, AT/TA-repeats were better sequenced than other 2bp-repeats. However, we emphasize that this trend only applies in a general sense, as there are considerable exceptions (Supplementary Fig. 4). We validated this hierarchy of sequencing accuracy among repeat units using the HG002 R9.4.1 dataset (Fig. 2b). For STR with longer repeat units, most STR displayed much better accuracy compared to 1bp- and 2bp-repeat STR (Supplementary Fig. 5), although it should be noted that their scarce nature limited our analysis to their relatively shorter forms. Moreover, we noticed a substantial variability of sequencing accuracy among STR, even among STR with identical repeat units and lengths (Fig. 2c).

Relationship between STR sequencing accuracy and flanking sequences

To explain the variability in sequencing accuracy among identical STR types (i.e., STRs with same repeat unit and same length) (as shown in Fig. 2c), we hypothesized that the flanking sequences of STRs may influence the sequencing accuracy. We tested this hypothesis by training a convolutional neural network (CNN) machine learning model using the flanking sequences of Ax10 STR regions (6 nucleotides for each direction, totaling 12 nucleotides), to predict the sequencing accuracy of Ax10 STR regions (n=39,594) (Fig. 3, Methods). The model displayed considerable predictive accuracy, as shown by the Pearson correlation value of 0.66 (Fig. 3b). Repeating the same process with either left or right flanking sequences yielded markedly lower Pearson correlation values, suggesting that flanking sequences influence the STR sequencing accuracy in both directions (Supplementary Fig. 6a). The STR accuracy of 2bp-repeats such as ATx8 (n=7,660) and ACx8 (n=5,916) repeats was also moderately predicted by our model, indicating that the association between sequencing accuracy and flanking sequences extends beyond Ax10 to other STR types. However, outliers in the CNN prediction suggest that flanking sequences alone do not fully determine the sequencing accuracy of STR regions. Although STR regions with identical flanking sequences generally exhibit similar sequencing accuracies, noticeable variation remained evident (Supplementary Fig. 6b).

Nonetheless, motivated by this finding, we identified motifs associated with high or low sequencing accuracy within the CNN model. We selected Ax10-Ax15 STR regions whose sequencing accuracies were well-predicted by the CNN model and applied UMAP using the flanking sequences as features (Methods, Fig. 4a). Mapping sequencing accuracy onto the UMAP revealed an interesting pattern: the sequencing accuracy appeared to be primarily with the nucleotides closest to the A-repeats. Notably, A-repeats flanked by two guanine nucleotides consistently exhibited poor sequencing accuracy. Furthermore, the distance between the flanking nucleotides and the A-repeats appeared to be inversely proportional to their influence on sequencing accuracy (Supplementary Fig. 7a). We identified the top 20 motifs and the worst 20 motifs of A-repeats (defined by the 2 flanking nucleotides on each side) whose effects on sequencing accuracy were relatively consistent across varying lengths of A-repeats (Fig. 4b, Methods). Top motifs were enriched with pyrimidine bases, while the worst motifs were enriched with purine bases (Supplementary Fig. 7b). These results were validated using the HG002 R9.4.1 dataset (Supplementary Fig. 7c). Finally, to address the potential bias introduced by filtering STR regions with low-complexity flanking sequences, we repeated the same analysis using these STR regions and obtained similar results (Supplementary Fig. 8, Methods).

The impact of basecaller on STR sequencing accuracy

A unique aspect of ONT data analysis is the basecalling process, which uses machine learning to convert electric signals into nucleotide sequences. Basecallers are regularly updated, allowing users to reanalyze their data using different versions. To explore the impact of basecallers on STR sequencing accuracy, we compared the performance of 4 basecaller versions – Guppy v5.0.7, Guppy v6.0.0, Guppy v6.5.7 (the final Guppy version) and Dorado v5.2.0 – by comparing the sequencing accuracy of Ax10 STR (n=1,552) and ATx10 STR (n=102) located in chromosome 1 of the T2T-CHM13 genome (Fig. 5a). High accuracy (HAC) model was used for all 4 basecaller versions. Guppy v6.5.7 and Dorado v5.2.0 exhibited similar performances and vastly outperformed the other two versions, highlighting the importance of using the most up-to-date basecaller. Next, we compared the influence of HAC model against super accuracy (SUP) model of Guppy v6.5.7 and observed considerable improvements (Fig. 5b). Given that the SUP model is known to offer only marginal improvements over the HAC model, this improvement was unexpectedly significant. However, this improvement was not uniform; while 71.5% of Ax10 STR regions were better resolved using the SUP model, the remaining 28.5% regions were better resolved with the HAC model (Fig. 5c). Notably, UMAP analysis of these loci revealed no segregation based on the basecalling model that best resolved each locus, suggesting that flanking sequences do not determine the better model (Supplementary Fig. 9). Nevertheless, in general, choosing the latest version and model provides significant benefit for STR analysis.

Base quality score of sequencing error in STR regions

Next, we examined whether the elevated error rates in STR regions were reflected in the base quality. First, we compared the average base quality scores of correctly sequenced reads against incorrectly sequenced reads and noted marginal differences (Fig. 6a). However, we observed a significant overestimation of the base quality scores of bases within the STR regions, regardless of the presence of errors (the average base quality score within the entire CHM13 dataset was approximately 20.67). Upon investigating the distribution of base quality scores of bases within and adjacent to STR regions (Supplementary Fig. 10a), we observed bursts of quality scores to abnormally high values. While this phenomenon was observed in 1bp-, 2bp-, and 3bp-repeat STRs, it was most evident in 2bp-repeat STRs. For the majority of 2bp-repeat STRs, the basecaller consistently assigned a fixed value of 90 as the base quality score for bases within the STR regions. Figure 6b shows the base quality score distribution within an ACx12 STR locus (chr10:25491367-25491390, T2T-CHM13v2.0), which demonstrates the typical base quality score distribution within 2bp-repeat STR regions. Notably, such quality score bursts were not observed in 4bp-, 5bp-, and 6bp-repeat STR.

We also examined the base quality scores of sequencing errors to assess their potential utility in sequencing error inference. The overall base quality scores of substitution errors within the STR regions were markedly lower than those of the correct bases (Fig. 6c). In contrast, the differences between base quality scores between correctly sequenced reads and reads harboring indel errors were unnoticeable, which was disappointing, considering that indel errors accounted for most sequencing errors (Supplementary Fig. 10b). We validated these findings by repeating the same analysis on the HG002 R9.4.1 dataset (Supplementary Fig. 11).

STR error profile of R10.4.1 flowcell.

We expanded our analysis by comparing the HG002 R10.4.1 dataset with the HG002 R9.4.1 dataset, incorporating reads that mapped to 1bp-, 2bp-, and 3bp-repeat STRs. The error profile of R10.4.1 resembled that of R9.4.1 (Fig. 7a). Although indel errors still accounted for the majority of sequencing errors (91%), R10.4.1 demonstrated significant improvement over its predecessor across nearly all 3 STR types, particularly for the GC-rich STR (Fig. 7b). We performed similar analyses performed throughout the study on the HG002 R10.4.1 dataset and show that all the topics discussed in this article – sequencing accuracy of various STR types, impact of basecallers, and the association of flanking sequences with sequencing accuracy – are largely maintained in R10.4.1 as well (Supplementary Fig. 12–14).

Notably, unlike with R9.4.1 (Fig. 5c), the advantages of the SUP model over the HAC model (Dorado v8.1.0) were more prominent and consistent, with 92.99% of Ax12 STR loci showing improvements (Supplementary Fig. 12b).

Development of NanoMnT

Although existing STR analysis tools discussed in the Introduction section excel in genotyping 3bp-, 4bp-, 5bp-, and 6bp-repeats, they are not designed for analyzing 2bp- and, especially 1bp-repeats. Thus, we developed NanoMnT, a lightweight Python-based tool that (1) corrects STR sequencing errors for ONT reads, (2) estimate allele sizes of user-specified STR loci using the corrected reads, and (3) searches for informative STR loci given the output files for paired normal and tumor samples (Methods, Fig. 8a). Using the HG002 R9.4.1 dataset, we tested the performance of NanoMnT against NanoRepeat and NanoSTR and confirmed that NanoMnT provides better STR allele size estimation for monoallelic 1bp-repeats and 2bp-repeats (Fig. 8b, Supplementary Fig. 15a). NanoMnT provides the prominence of the allele histogram peak, which can be used as quality measure; high peak prominence generally corresponding to confident allele estimation results (Fig. 8c, Supplementary Fig. 15b). When estimating Ax10 repeats, selectively using reads based on their sequencing orientation (e.g., forward strand reads for A-repeat STRs and reverse strand reads for T-repeat STRs) improves accuracy from 55% (without read selection) to 78% (Supplementary Fig. 15c). This accuracy can be further increased to 85% by excluding loci flanked by guanine nucleotides. These results highlight the practicability of our findings.

Next, we evaluated the impact of sequencing coverage and the flowcell version on NanoMnT performance by using inputs of varying sequencing parameters (Fig. 9). The HG002 R9.4.1 and HG002 R10.4.1 datasets were used to compare the two flowcell versions. STR allele estimation with the R10.4.1 data produced more accurate results for both 1bp-repeat STR (44% more accurate on average) and 2bp-repeat STR (23% more accurate on average), reflecting the advancements introduced by the R10.4.1 flowcell and the V14 chemistry. This improvement was especially pronounced in 2bp-repeat STR regions, where R10.4.1 maintained consistently high accuracy even for longer repeat lengths. Coverage was also an important factor that affected allele estimation results for R10.4.1 data. As expected, higher coverage led to more accurate results.

MSI detection of cancer samples from ONT data using NanoMnT

We integrated our findings into a biological context by identifying MSI status of cancer samples from the bulk RNA sequencing dataset created by SG-NEx [36]. Among the many types of ONT sequencing datasets provided by SG-NEx, we chose the PCR-free direct cDNA sequencing data to ensure the absence of PCR stutter. Conventionally, the MSI status is determined by comparing the STR allele size histograms of the tumor sample with those of the corresponding normal sample. Unfortunately, due to the lack of matched normal sample, we used the CHM13 genome as the substitute normal sample. We calculated the relative allele sizes of STR loci and compared their distribution among samples to identify MSI and microsatellite stable (MSS) cancers (Methods). To showcase the importance of bioinformatics strategies for analyzing ONT data, we compared the MSI identification results derived from 3 distinct versions of FASTQ data obtained from the same sample: (1) raw FASTQ files provided by SG-NEx which were basecalled using Guppy version 3.2.10; (2) data re-basecalled using the latest version of Guppy, version 6.5.7 (HAC); and (3) data re-basecalled using Guppy version 6.5.7 (HAC), while applying read-selection and STR loci-selection process to achieve better accuracy (Fig. 10a, Methods). Overall, the STR allele sizes of the MSI cell line were shorter than those of the MSS cell lines, aligning with previous reports that deletion mutations predominate in mononucleotide repeats within MSI [44]. While simply re-basecalling the data with Guppy v6.5.7 was enough in separating the STR allele profiles of the MSI cell line from those of the MSS cell lines, the read selection and/or loci selection process gave markedly better results (Fig. 10b).

We performed a similar analysis on the CRC organoid dataset created by Pickles et al. [37], who conducted WGS on 15 primary CRC organoids, each labeled with MMR status and consensus molecular subtype (CMS) (Fig. 10c). Although we could not re-basecall this dataset with the latest basecaller because the raw FAST5/POD5 files were unavailable, we still showed that dMMR status could be readily identified, except for sample 064 and 080. Although these two discordant results may be false positives, they could also reflect the intratumoral heterogeneity of CRC. Indeed, several studies have reported coexistence of CMS1 – which is almost exclusively enriched in MSI CRC – and other CMS CRC within individual patients [46-48].

Discussion

The capacity of ONT to generate long reads, along with its portability and versatility, makes it an attractive approach in many research fields. However, the high error rate of ONT in low-complexity regions hinders its application in STR-related fields. This study provides a comprehensive overview of ONT sequencing profiles in STR regions by measuring

the abundance of sequencing errors in various STR types and identifying factors that influence STR sequencing accuracy. Indels were responsible for most sequencing errors, with deletions being more prevalent than insertions. In addition, we observed a substantial overestimation of base quality scores in STR regions, which may suggest that the basecaller machine learning models are not properly tailored for STR regions. While base quality scores of substitution error bases and correct bases differed significantly—suggesting the potential of base quality score in inferring substitution errors—we did not observe such difference between indel errors and correctly sequenced bases, which is unfortunate, considering the abundance of indel errors. We also compared the STR error profiles between the R10.4.1 and R9.4.1 flowcells and observed a significant improvement in the R10.4.1 flowcell. Although the overall frequency of each type of sequencing error was similar between the two flowcells (with indels comprising the majority), the total number of errors was markedly reduced in the R10.4.1 flowcell. Consequently, as shown in Figure 9, allele estimation accuracy for both 1bp-repeat and 2bp-repeat STR loci was substantially enhanced.

In this study, we identified 3 factors that influence the ONT sequencing accuracy of STR. First, the sequencing accuracy of STRs was heavily influenced by the repeat unit of the sequenced STR, specifically the repeat units that entered the nanopores. This finding suggests a strategic approach when analyzing STR from ONT data: preferential usage of reads with specific orientation over reads with opposite orientation may achieve superior accuracy, given that the sequencing depth is sufficiently high. This finding was consistently observed in both versions of flowcells (R9.4.1 and R10.4.1) and basecalling programs, indicating that the electric signals associated with some repeat units may be intrinsically more resolvable for ONT compared to others. Second, flanking sequences were also associated with the sequencing accuracy of STR regions, implying that careful selection of STR loci based on their flanking sequences may help mitigate the high error rate of ONT. For example, A-repeats with purine-rich flanking sequences were linked to worse sequencing accuracy compared to A-repeat with pyrimidine-rich flanking sequences. This could be due to the high similarity of electric signals produced by the A-repeats and the purine-rich flanking sequences. Thirdly, we highlighted the significance of basecaller version, which is possibly the most influential factor of sequencing accuracy, as shown in Figure 5. Therefore, we encourage researchers who have previously generated ONT sequencing data, to re-basecall their data using the latest basecaller for STR related analyses such as MSI identification.

We introduced NanoMnT, a lightweight Python-based tool that performs error correction in STR regions by choosing the most parsimonious allele, i.e., allele with the minimum Levenshtein distance compared to the observed allele, and genotypes STR regions using these corrections. Although there are existing tools that serve similar purposes, none of them have been designed to genotype 1bp- and 2bp-repeat STR. Instead, to the best of our knowledge, most tools are designed to genotype STR with longer repeat units to study areas such as neurological disease [34] and forensics [31]. Benchmarking analyses demonstrate that NanoMnT provides superior STR allele estimation for monoallelic 1bp- and

2bp-repeat STR loci. By applying NanoMnT on two cancer datasets, we were able to identify MSI status of various cancer samples. However, we acknowledge a major caveat of NanoMnT: NanoMnT lacks the capability to phase multiple alleles, making it unsuitable for analyzing polyallelic STR loci. If heterozygosity is expected, we encourage users to examine NanoMnT output metrics (e.g., peak prominence or allele histogram visualizations), or to use a different tool capable of detecting heterozygotic STR alleles, such as NanoRepeat, NanoSTR, or WarpSTR.

We acknowledge several limitations of this study. First, our study was solely focused on perfect tandem repeats, excluding many types of repeats such as compound repeats and imperfect tandem repeats. Future studies will be needed to assess the performance of ONT in analyzing these types of repeats. Second, although we identified certain motifs that are enriched in well-/poorly sequenced A-repeat STR, we failed to provide a comprehensive mechanism that explains the influence of flanking sequences on STR sequencing accuracy. Also, the CNN machine learning model did not exhibit optimal predictive accuracy, indicating the presence of additional factors that we could not detect and/or the stochastic nature of ONT error profile. We note that the lack of diversity of flanking sequences within the human genome – since a major portion of A-/T-repeat STR originates from mobile genetic elements such as Alu elements – may have exacerbated the CNN prediction results. Thus, using a sufficiently diverse set of flanking sequences may improve our understanding of the association between flanking sequences and sequencing accuracy. Lastly, while NanoMnT outperforms existing tools in estimating 1bp-repeat and 2bp-repeat STR loci, its overall accuracy remains suboptimal for 1bp-repeat STRs. This limitation is expected to improve as ONT continues to update its flowcells and enhance sequencing accuracy.

Availability of source code and requirements

Project name: NanoMnT

Project home page: https://github.com/18parkky/NanoMnT

Operating systems: Tested on Ubuntu, CentOS 7 and macOS (Sonoma 14.1.2)

Programming language: Python

Other requirements: Python 3.x, Matplotlib>3.7.1, Numpy>1.20.3, Pysam>0.20.0, Pandas>2.0.0, Scipy>1.7.1, Seaborn>0.13.0

License: MIT

RRID: RRID:SCR_026210

bio.tools ID: nanomnt

Data Availability

The sequencing dataset generate for CHM13 was accessed via the Telomere-to-Telomere consortium CHM13 project GitHub page [51]. Sequencing datasets for HG002 were accessed through the Dataset Releases made available by EPI2ME [52, 53]. SG-NEx RNA-seq dataset was accessed through the SG-NEx GitHub page [54] and WGS dataset of CRC organoids generated by Pickles et al was downloaded from NCBI (PRJNA978372). Other data further supporting this work are openly available in the GigaScience repository, GigaDB [55]. DOME-ML (Data, Optimisation, Model, and Evaluation in Machine Learning) annotations supporting the current study, is available in the DOME Registry [56]. A snapshot of the GitHub repository [49] has also been archived in Software Heritage [50].

List of abbreviations

bp: base pair; CMS: consensus molecular subtype; CNN: convolutional neural network; CRC: colorectal cancer; dMMR: mismatch repair deficiency; HAC: high accuracy; MSI: microsatellite instability; MSS: microsatellite stability; NGS: next-generation sequencing; ONT: Oxford Nanopore Technology; SG-NEx: The Singaporean Nanopore Expression Dataset; STR: short tandem repeat; SUP: super accuracy; WGS: whole-genome sequencing.

Declarations

Consent for publication

N/A

Competing interests

The authors declare that they have no competing interests.

Funding

This work was supported GIST-CNUH Research Collaboration grant and GIST-MIT Research collaboration grant funded by the GIST in 2024; the National Research Foundation of Korea (NRF) and Korea Technology and Information Promotion Agency for SMEs, funded by the Korean government (RS-2024-00335026, RS-2024-00506966); National

Natural Science Foundation of China (82103031, 82272933 , 82422050); and Sichuan Science and Technology Program (2023YFS0098)

Authors' contributions

Gyumin Park (Conceptualization, Software, Visualization, Writing – original draft, Writing – review & editing), Hyunsu An (Data curation, Formal analysis), Han Luo (Conceptualization, Writing – original draft, Writing – review & editing, Supervision), Jihwan Park (Conceptualization, Writing – original draft, Writing – review & editing, Supervision)

References

U Gymrek M. A genomic view of short tandem repeats. Curr Opin Genet Dev. 2017;44:9-16. doi:10.1016/j.gde.2017.01.012.

2. Alonso A, Barrio PA, Muller P, Kocher S, Berger B, Martin P, et al. Current state-of-art of STR sequencing in forensic genetics. Electrophoresis. 2018;39 21:2655-68. doi:10.1002/elps.201800030.
3. Bruford MW and Wayne RK. Microsatellites and their application to population genetic studies. Curr Opin Genet Dev. 1993;3 6:939-43. doi:10.1016/0959-437x(93)90017-j.
4. Gymrek M, Willems T, Guilmatre A, Zeng H, Markus B, Georgiev S, et al. Abundant contribution of short tandem repeats to gene expression variation in humans. Nat Genet. 2016;48 1:22-9. doi:10.1038/ng.3461.
5. Fotsing SF, Margoliash J, Wang C, Saini S, Yanicky R, Shleizer-Burko S, et al. The impact of short tandem repeat variation on gene expression. Nat Genet. 2019;51 11:1652-9. doi:10.1038/s41588-019-0521-9.
6. Horton CA, Alexandari AM, Hayes MGB, Marklund E, Schaepe JM, Aditham AK, et al. Short tandem repeats bind transcription factors to tune eukaryotic gene expression. Science. 2023;381 6664:eadd1250. doi:10.1126/science.add1250.
7. Brinkmann B, Klintschar M, Neuhuber F, Huhne J and Rolf B. Mutation rate in human microsatellites: influence of the structure and length of the tandem repeat. Am J Hum Genet. 1998;62 6:1408-15. doi:10.1086/301869.
8. Malik I, Kelley CP, Wang ET and Todd PK. Molecular mechanisms underlying nucleotide repeat expansion disorders. Nat Rev Mol Cell Biol. 2021;22 9:589-607. doi:10.1038/s41580-021-00382-6.
9. Li K, Luo H, Huang L, Luo H and Zhu X. Microsatellite instability: a review of what the oncologist should know. Cancer Cell Int. 2020;20:16. doi:10.1186/s12935-019-1091-8.
10. Chang L, Chang M, Chang HM and Chang F. Microsatellite Instability: A Predictive Biomarker for Cancer Immunotherapy. Appl Immunohistochem Mol Morphol. 2018;26 2:e15-e21. doi:10.1097/PAI.0000000000000575.
11. Suraweera N, Duval A, Reperant M, Vaury C, Furlan D, Leroy K, et al. Evaluation of tumor microsatellite instability using five quasimonomorphic mononucleotide repeats and pentaplex PCR. Gastroenterology. 2002;123 6:1804-11. doi:10.1053/gast.2002.37070.
12. Kautto EA, Bonneville R, Miya J, Yu L, Krook MA, Reeser JW, et al. Performance evaluation for rapid detection of pan-cancer microsatellite instability with MANTIS. Oncotarget. 2017;8 5:7452-63. doi:10.18632/oncotarget.13918.
13. Tanudisastro HA, Deveson IW, Dashnow H and Macarthur DG. Sequencing and characterizing short tandem repeats in the human genome. Nat Rev Genet. 2024;25 7:460-75. doi:10.1038/s41576-024-00692-3.
14. Liu Q, Tong Y and Wang K. Genome-wide detection of short tandem repeat expansions by long-read sequencing. BMC Bioinformatics. 2020;21 Suppl 21:542. doi:10.1186/s12859-020-03876-w.
15. Wei N, Bemmels JB and Dick CW. The effects of read length, quality and quantity on microsatellite discovery and primer development: from Illumina to PacBio. Mol Ecol Resour. 2014;14 5:953-65. doi:10.1111/1755-0998.12245.
16. Rhoads A and Au KF. PacBio Sequencing and Its Applications. Genomics Proteomics Bioinformatics. 2015;13 5:278-89. doi:10.1016/j.gpb.2015.08.002.

17. Wang Y, Zhao Y, Bollas A, Wang Y and Au KF. Nanopore sequencing technology, bioinformatics and applications. Nat Biotechnol. 2021;39 11:1348-65. doi:10.1038/s41587-021-01108-x.

18. Wenger AM, Peluso P, Rowell WJ, Chang PC, Hall RJ, Concepcion GT, et al. Accurate circular consensus long-read sequencing improves variant detection and assembly of a human genome. Nature Biotechnology. 2019;37 10:1155-+. doi:10.1038/s41587-019-0217-9.

19. Jain M, Koren S, Miga KH, Quick J, Rand AC, Sasani TA, et al. Nanopore sequencing and assembly of a human genome with ultra-long reads. Nat Biotechnol. 2018;36 4:338-45. doi:10.1038/nbt.4060.

20. Gunter HM, Youlten SE, Reis ALM, McCubbin T, Madala BS, Wong T, et al. A universal molecular control for DNA, mRNA and protein expression. Nat Commun. 2024;15 1:2480. doi:10.1038/s41467-024-46456-9.

21. Stevens BM, Creed TB, Reardon CL and Manter DK. Comparison of Oxford Nanopore Technologies and Illumina MiSeq sequencing with mock communities and agricultural soil. Sci Rep-Uk. 2023;13 1 doi:10.1038/s41598-023-36101-8.

22. Delahaye C and Nicolas J. Sequencing DNA with nanopores: Troubles and biases. PLoS One. 2021;16 10:e0257521. doi:10.1371/journal.pone.0257521.

23. Watson M and Warr A. Errors in long-read assemblies can critically affect protein prediction. Nature Biotechnology. 2019;37 2:124-6. doi:10.1038/s41587-018-0004-z.

24. Sereika M, Kirkegaard RH, Karst SM, Michaelsen TY, Sorensen EA, Wollenberg RD, et al. Oxford Nanopore R10.4 long-read sequencing enables the generation of near-finished bacterial genomes from pure cultures and metagenomes without short-read or reference polishing. Nat Methods. 2022;19 7:823-6. doi:10.1038/s41592-022-01539-7.

25. Ni Y, Liu X, Simeneh ZM, Yang M and Li R. Benchmarking of Nanopore R10.4 and R9.4.1 flow cells in single-cell whole-genome amplification and whole-genome shotgun sequencing. Comput Struct Biotechnol J. 2023;21:2352-64. doi:10.1016/j.csbj.2023.03.038.

26. Ahsan MU, Liu Q, Perdomo JE, Fang L and Wang K. A survey of algorithms for the detection of genomic structural variants from long-read sequencing data. Nature Methods. 2023;20 8:1143-58. doi:10.1038/s41592-023-01932-w.

27. Sitarcik J, Vinar T, Brejová B, Krampl W, Budis J, Radvánszky J, et al. WarpSTR: determining tandem repeat lengths using raw nanopore signals. Bioinformatics. 2023;39 6 doi:10.1093/bioinformatics/btad388.

28. Fang L, Liu Q, Monteys AM, Gonzalez-Alegre P, Davidson BL and Wang K. DeepRepeat: direct quantification of short tandem repeats on signal data from nanopore sequencing. Genome Biology. 2022;23 1 doi:10.1186/s13059-022-02670-6.

29. Giesselmann P, Brändl B, Raimondeau E, Bowen R, Rohrandt C, Tandon R, et al. Analysis of short tandem repeat expansions and their methylation state with nanopore sequencing. Nature Biotechnology. 2019;37 12:1478-+. doi:10.1038/s41587-019-0293-x.

30. De Roeck A, De Coster W, Bossaerts L, Cacace R, De Pooter T, Van Dongen J, et al. NanoSatellite: accurate characterization of expanded tandem repeat length and sequence through whole genome long-read sequencing on PromethION. Genome Biology. 2019;20 1 doi:10.1186/s13059-019-1856-3.

31. Lang J, Xu Z, Wang Y, Sun J and Yang Z. NanoSTR: A method for detection of target short tandem repeats based on nanopore sequencing data. Front Mol Biosci. 2023;10:1093519. doi:10.3389/fmolb.2023.1093519.

32. Ummat A and Bashir A. Resolving complex tandem repeats with long reads. Bioinformatics. 2014;30 24:3491-8. doi:10.1093/bioinformatics/btu437.

33. Chiu R, Rajan-Babu IS, Friedman JM and Birol I. Straglr: discovering and genotyping tandem repeat expansions using whole genome long-read sequences. Genome Biology. 2021;22 1 doi:10.1186/s13059-021-02447-3.

34. Fang L, Monteys AM, Durr A, Keiser M, Cheng C, Harapanahalli A, et al. Haplotyping SNPs for allele-specific gene editing of the expanded huntingtin allele using long-read sequencing. HGG Adv. 2023;4 1:100146. doi:10.1016/j.xhgg.2022.100146.

35. Mitsuhashi S, Frith MC, Mizuguchi T, Miyatake S, Toyota T, Adachi H, et al. Tandem-genotypes: robust detection of tandem repeat expansions from long DNA reads. Genome Biology. 2019;20 doi:10.1186/s13059-019-1667-6.

36. Chen Y, Davidson N, Wan YK, Patel H, Yao F, Low HM, et al. A systematic benchmark of Nanopore long read RNA sequencing for transcript level analysis in human cell lines. bioRxiv, 2021.

37. Pickles OJ, Wanigasooriya K, Ptasinska A, Patel AJ, Robbins HL, Bryer C, et al. MHC Class II is Induced by IFNgamma and Follows Three Distinct Patterns of Expression in Colorectal Cancer Organoids. Cancer Res Commun. 2023;3 8:1501-13. doi:10.1158/2767-9764.CRC-23-0091.

38. Du L, Zhang C, Liu Q, Zhang X, Yue B and Hancock J. Krait: an ultrafast tool for genome-wide survey of microsatellites and primer design. Bioinformatics. 2018;34 4:681-3. doi:10.1093/bioinformatics/btx665.

39. Shumate A and Salzberg SL. Liftoff: accurate mapping of gene annotations. Bioinformatics. 2021;37 12:1639-43. doi:10.1093/bioinformatics/btaa1016.

40. Li H. Minimap2: pairwise alignment for nucleotide sequences. Bioinformatics. 2018;34 18:3094-100. doi:10.1093/bioinformatics/bty191.

41. Martín Abadi AA, Paul Barham, Eugene Brevdo,, Zhifeng Chen CC, Greg S. Corrado, Andy Davis,, Jeffrey Dean MD, Sanjay Ghemawat, Ian Goodfellow,, Andrew Harp GI, Michael Isard, Rafal Jozefowicz, Yangqing Jia,, Lukasz Kaiser MK, Josh Levenberg, Dan Mané, Mike Schuster,, Rajat Monga SM, Derek Murray, Chris Olah, Jonathon Shlens,, et al. TensorFlow: Large-scale machine learning on heterogeneous systems. tensorflow2015-whitepaper. 2015.
42. Virshup I, Bredikhin D, Heumos L, Palla G, Sturm G, Gayoso A, et al. The scverse project provides a computational ecosystem for single-cell omics data analysis. Nat Biotechnol. 2023;41 5:604-6. doi:10.1038/s41587-023-01733-8.
43. Wolf FA, Angerer P and Theis FJ. SCANPY: large-scale single-cell gene expression data analysis. Genome Biol. 2018;19 1:15. doi:10.1186/s13059-017-1382-0.
44. Aska EM, Zagidullin B, Pitkanen E and Kauppi L. Single-Cell Mononucleotide Microsatellite Analysis Reveals Differential Insertion-Deletion Dynamics in Mouse T Cells. Front Genet. 2022;13:913163. doi:10.3389/fgene.2022.913163.
45. Bonfield JK, Marshall J, Danecek P, Li H, Ohan V, Whitwham A, et al. HTSlib: C library for reading/writing high-throughput sequencing data. Gigascience. 2021;10 2 doi:10.1093/gigascience/giab007.
46. Valdeolivas A, Amberg B, Giroud N, Richardson M, Galvez EJC, Badillo S, et al. Profiling the heterogeneity of colorectal cancer consensus molecular subtypes using spatial transcriptomics. NPJ Precis Oncol. 2024;8 1:10. doi:10.1038/s41698-023-00488-4.
47. Lee HO, Hong Y, Etlioglu HE, Cho YB, Pomella V, Van den Bosch B, et al. Lineage-dependent gene expression programs influence the immune landscape of colorectal cancer. Nat Genet. 2020;52 6:594-603. doi:10.1038/s41588-020-0636-z.
48. Guinney J, Dienstmann R, Wang X, de Reynies A, Schlicker A, Soneson C, et al. The consensus molecular subtypes of colorectal cancer. Nat Med. 2015;21 11:1350-6. doi:10.1038/nm.3967.
49. NanoMnT. GitHub repository. https://github.com/18parkky/NanoMnT. Accessed 25 Jan 2025.
50. Park G, An H, Luo H and Park J. (2025) NanoMnT: An STR Analysis Tool for Oxford Nanopore Sequencing Data Driven by a Comprehensive Analysis of Error Profile in STR regions (Version 1). [Computer software]. Software Heritage, https://archive.softwareheritage.org/swh:1:snp:ca2f69be469dd1ffb87686cdad2fefd5950e28dc;origin=https://github.com/18parkky/NanoMnT.
51. Telomere-to-telomere consortium CHM13 project. GitHub repository. https://github.com/marbl/CHM13/blob/master/Sequencing_data.md. Accessed 25 Jan 2025.
52. Wright C: Genome in a Bottle Ashkenazi Trio with Ligation Sequencing Kit V14. https://labs.epi2me.io/askenazi-kit14-2022-12/ (2023). Accessed Jan 25 2025.
53. Wright C: GM24385 Dataset Release. https://labs.epi2me.io/gm24385_2020.09/ (2020). Accessed Jan 25 2025.
54. https://github.com/GoekeLab/sg-nex-data (The Singapore Nanopore Expression Project. GitHub Repository). 25 Jan 2025.
55. Park G; An H; Luo H; Park J: Supporting data for "NanoMnT: A STR analysis tool for Oxford Nanopore sequencing data driven by comprehensive analysis of error profile in STR regions" GigaScience Database. 2025. https://doi.org/10.5524/102658.
56. Park G, An H, Luo H and Park J. (2025) NanoMnT: An STR Analysis Tool for Oxford Nanopore Sequencing Data Driven by a Comprehensive Analysis of Error Profile in STR regions. [DOME-ML Annotations]. DOME-ML Registry, https://registry.dome-ml.org/review/hjnyjl40c9.

Figure legends

Figure 1. Distribution of ONT sequencing error in STRs.

(a) Percentage of errorless reads in 1bp-, 2bp- and 3bp-repeat STRs (left) and distribution of sequencing error types (right). 1bp-repeats with 10~30 repeats, 2bp-repeats with 7~24 repeats, 3bp-repeats with 5~15 repeats were analyzed. **(b)** STR allele size histograms of various lengths of 1bp-, 2bp- and 3bp-repeat STRs.

Figure 2. Sequencing accuracy of STRs based on their repeat units.

(a) Sequencing accuracy (measured by calculating the percentage of errorless reads) of varying lengths of 1bp-repeat STRs (left) and 2bp-repeat STRs (right) based on their repeat units. **(b)** Sequencing accuracy of Nx10 and NNx8 STRs of various repeat units, compared between two separate datasets. **(c)** Distribution of sequencing accuracy of Ax12, Ax18 and Ax21 STRs, demonstrating the extreme variability of sequencing accuracy among identical types of STRs.

Figure 3. CNN-based machine learning prediction of STR sequencing accuracy using flanking sequences.

(a) Illustration of CNN-based machine learning prediction workflow. **(b)** Prediction results of Ax10 STRs (left), ATx8 STRs (upper right), and ACx8 STRs (lower right) sequencing accuracy.

Figure 4. Identification of motifs associated with good/bad sequencing accuracy of A-repeat STRs.

(a) UMAP projection of flanking sequences of Ax10-Ax15 repeat STRs. Each dot represents an A-repeat STR locus, colored by its sequencing accuracy (left) and by its most adjacent flanking nucleotides (right). **(b)** Flanking sequences of A-repeat STRs (2 nucleotides in each direction, 4 nucleotides total) associated with good and bad sequencing accuracy. 2 nucleotides in each direction are separated by slash (e.g., CT/CG motif indicates CT-$(A)_n$-CG).

Figure 5. Influence of basecaller on STR sequencing accuracy.

(a) Comparison of 4 ONT basecallers in basecalling Ax10 STRs (left) and ATx10 STRs (right), visualized with kernel density estimate plots. **(b)** Comparison of HAC model and SUP model in basecalling Ax10 STRs, visualized with kernel density estimate plots. Both models are from Guppy v6.5.7. **(c)** Sequencing accuracy of Ax10 STRs obtained by the HAC basecaller model (x-axis) and the SUP basecaller model (y-axis), where each cross represents a single Ax10 STR locus. Loci that exhibited better sequencing accuracy with either SUP basecaller model (71.5%) or HAC basecaller model (28.5%) were marked with different colors.

Figure 6. ONT base quality scores in STR regions.

(a) Base quality score comparison between correctly sequenced reads and reads containing sequencing error across various STR types. The red dashed horizontal line indicates the estimated base quality average across the entire CHM13 dataset (20.7). **(b)** Base quality score distribution in an ACx12 STR locus (chr10:25491367-25491390, T2T-CHM13 v2.0). Each dot represents the base quality score reported by a single read and the line represents the average score of each genomic position. **(c)** Base quality score comparison of correctly sequenced bases and substitution error bases, visualized with kernel density estimate plots.

Figure 7. Comparison of ONT STR sequencing profile between the R9.4.1 and the R10.4.1 flowcell.

(a) Distribution of correctly sequenced reads and various types of sequencing errors in 1bp-, 2bp- and 3bp-repeat STRs, compared between the R9.4.1 (HG002 R9.4.1 dataset) and the R10.4.1 (HG002 R10.4.1 dataset) flowcell. **(b)** Changes in sequencing accuracy from R9.4.1 to R10.4.1 of various types of STR. Accuracy change (y-axis) is measured by dividing the sequencing accuracy observed in R9.4.1 with the sequencing accuracy of R10.4.1 (e.g., 2.0 indicates 200% improvement). The dots in each bar plot represents STRs of different lengths, with longer STR represented by darker colors.

Figure 8. Development of NanoMnT.

(a) Schematic overview of NanoMnT functionality. First, NanoMnT performs rudimentary STR error correction in read level and generates a tab-delimited file (TSV) named Allele Table. The Allele table is then used to estimate STR allele sizes of user-specified STR loci by comparing the observed STR allele size histogram against many synthetic STR allele size histograms, where the synthetic histogram with the most resemblance is chosen as the putative allele histogram. This process generates another TSV file, called the Locus Table. Given the Allele Table and the Locus Table of paired normal and tumor samples, NanoMnT compares the STR loci captured in both samples, to search for loci that may provide useful information regarding the tumor's MSI status. **(b)** Benchmark results of NanoMnT, NanoSTR and NanoRepeat in estimating STR allele sizes of 300 1bp-repeat STR loci. **(c)** Distribution of peak prominence value (which indicates the prominence of the STR allele size histogram of each STR locus, calculated using SciPy *find_peaks* function) reported by NanoMnT (top), and the change in percentage of STR loci whose allele size have been correctly estimated by thresholding peak prominence (bottom). For example, ~90% of STR loci whose peak prominences exceed 50 are correctly estimated. The red dashed horizontal line represents the total percentage of STR loci that have been correctly estimated by NanoMnT, as shown in Figure b.

Figure 9. NanoMnT performance across flowcell versions and coverage.

Percentage of correct allele estimation for 1bp-repeat and 2bp-repeat STRs by NanoMnT across varying sequencing coverages and flowcell types. The HG002 R9.4.1 dataset and the HG002 R10.4.1 dataset were employed for this benchmark. The upper two plots compare NanoMnT performance between the two flowcells, while the lower two plots illustrate the impact of sequencing coverage (10x, 30x, 60x) on allele estimation accuracy.

Figure 10. MSI identification results of SG-NEx and CRC organoid WGS sequencing data.

(a) Distribution of STR allele sizes relative to those of the CHM13 genome in 4 cancer cell lines, visualized by box plots and strip plots. 4 analysis approaches are compared; Naïve (Guppy v3.2.10), Naïve (Guppy v6.5.7), read selection approach, and read + loci selection approach. **(b)** Comparison of STR allele size distributions between the MSS cell lines and the MSI cell line when employing the 4 different analysis approaches, visualized by kernel density estimate plots (left) and the area of intersection between the kernel density estimate plots of MSS and MSI. The red dashed vertical lines in the left figure represents the reference STR allele size (zero). **(c)** Average STR allele sizes of 15 CRC samples visualized by bar plots, with each sample colored by its reported CMS type.

Supplementary Figure 1.

(a) Percentage of chromosomes which reads (of the CHM13 dataset, whose primary alignments aligned to chromosome 21) realigned to. **(b)** Mapping quality distribution of two groups of the realigned reads: reads that mapped back to chromosome 21 and reads that mapped elsewhere. **(c)** Distribution of the number of alignments per read. E.g., 1 indicates that a read aligned to a single region, and 2 indicates that a read aligned to two different regions.

Supplementary Figure 2.

(a) Number of STRs analyzed in this study. **(b)** Percentage of 1bp-/2bp-repeat STRs by repeat units.

Supplementary Figure 3.

STR allele size histograms of various lengths of 4bp-, 5bp- and 6bp-repeat STR.

Supplementary Figure 4.

Distribution of forward strand sequencing accuracy (i.e., sequencing accuracy calculating using only forward strand reads) minus reverse strand sequencing accuracy in Ax12 STRs. 21.7% of Ax12 STR loci exhibited better sequencing accuracy when using reverse strand reads, while the remaining 78.3% of Ax12 STR loci exhibited the opposite.

Supplementary Figure 5.

Sequencing accuracy of 3bp-, 4bp-, 5bp- and 6bp-repeat STRs. Synonymous STR types (e.g., ACG-, CGA-, GAC-repeats) were grouped together and represented by the ‘representative’ repeat unit (e.g., ACG-repeat). STRs with at least 30 observations were included in this plot. The colorbar next to each figure represents the sequencing accuracy.

Supplementary Figure 6.

(a) Prediction of sequencing accuracy of Ax10 STRs using left or right flanking sequences as inputs. **(b)** Standard deviation of sequencing accuracy of Ax10 STRs that share the identical flanking sequences, compared against randomly sampled Ax10 STRs, demonstrating that flanking sequences indeed influence the sequencing accuracy of A-repeat STRs.

Supplementary Figure 7.

(a) Sequencing accuracy of A-repeat STRs that possesses specific pairs of nucleotides in specific distances within their flanking sequences. The influence of nucleotide pair on sequencing accuracy is proportionate to its proximity to A-repeat STR. **(b)** Sequencing accuracy of A-repeat STRs based on the number of purine counts in their flanking sequences of 4 nucleotides (2 nucleotide in each direction) **(c)** Sequencing accuracy of A-repeat STRs measured from the HG002 R9.4.1 dataset. A-repeat STRs that were flanked by the motifs identified in the CHM13 dataset (see the x-axis of Figure 4b) were shown.

Supplementary Figure 8.

(a) Sequencing accuracy (percentage of errorless reads) of A-repeat STRs with low-complex (LC) flanking sequences, that are flanked by motifs shown in Figure 4b. Each column represents a motif that flank the A-repeat STR, and each row represents the sequencing accuracy of A-repeat STRs with different numbers of repeats. **(b)** Average sequencing accuracy of Ax10-Ax13 STRs with LC flanking sequences (converted to percentile), where each dot represents A-repeat STRs with different flanking sequence motifs. **(c)** Relationship between purine counts of A-repeat STRs with LC flanking sequences and sequencing accuracy.

Supplementary Figure 9.

UMAP visualization of A-repeat STRs, created by using flanking sequences as features. Each dot represents a A-repeat locus, colored by sequencing accuracy (upper left), better basecalling model (i.e., model that generated better results for the given locus) (upper right) and the most adjacent flanking sequence (lower left).

Supplementary Figure 10.

(a) Base quality scores of STRs. The two vertical lines represent the start and end of the repeat sequences, while the horizontal lines represent the average base quality of the CHM13 dataset, highlighting the base quality 'burst' observed within the STR regions of some STR types. **(b)** Average base quality score of reads that are presumed to harbor indel errors in STR regions.

Supplementary Figure 11.

(a) The average base quality of reads in various STR types observed in the HG002 R9.4.1 dataset, comparing correctly sequenced reads (i.e., reads with no error within STR region) against incorrectly sequenced reads. The horizontal line represents the average base quality of the HG002 R9.4.1 dataset. **(b)** The base quality 'burst' observed in the HG002 R9.4.1 dataset. **(c)** Distribution of base quality compared between correctly sequenced bases and substitution errors.

Supplementary Figure 12.

(a) Sequencing accuracy of Nx12 and NNx8 STRs by various repeat units, compared between HG002 R9.4.1 dataset and HG002 R10.4.1 dataset. **(b)** Sequencing accuracy of Ax12 STRs observed in HG002 R10.4.1 dataset, comparing HAC basecalling model against SUP basecalling model. Each cross in the scatterplot (left) represents a Ax12 STR locus. Around 92.99% of Ax12 STRs are better resolved using SUP basecaller model, whereas 7.01% of Ax12 STRs are better resolved using HAC basecaller model (right).

Supplementary Figure 13.

(HG002 R10.4.1 dataset) Sequencing accuracy of A-repeat STRs that harbor certain motifs in their flanking sequences, ordered by the 'best' and 'worst' motifs found in the CHM13 dataset (top) and the HG002 R10.4.1 dataset (bottom).

Supplementary Figure 14.

(a) The average base quality of reads in various STR types observed in the HG002 R10.4.1 dataset, comparing correctly sequenced reads (i.e., reads with no error within STR region) against incorrectly sequenced reads. The horizontal line represents the average base quality of the HG002 R10.4.1 dataset. **(b)** The base quality 'burst' observed in the HG002 R10.4.1 dataset. **(c)** Distribution of base quality compared between correctly sequenced bases and substitution errors.

Supplementary Figure 15.

(a) NanoMnT, NanoSTR and NanoRepeat genotyping results of 300 2bp-repeat STR loci. **(b)** STR allele size histograms of 2 example Ax15 loci, one with a highly prominent peak, and the other with a less prominent peak. The dashed line represents the genotyped STR allele for each locus.

Your MS Excel document "Supplementary tables.xlsx" cannot be opened and processed. Please see the common list of problems, and suggested resolutions below.

External Data

If you are submitting an Excel file, please make sure that your document does not have links to external data. If it does, break the links, save the document and resend. To break the links please do the following.
-On the Edit menu, click Links.
-In the Source list, click the link you want to break.
-To select multiple linked objects, hold down CTRL and click each linked object. To select all links, press CTRL+A.
-Click Break Link.

Embedded Macros

Your submission should not contain macros. If they do, an alert box may appear when you open your document (this alert box prevents EM from automatically converting your Excel document into the PDF that Editors and Reviewers will use). You must adjust your Excel document to remove these macros.

Excel 2002/Excel XP files

At the present time, EM supports Excel files in Excel 2000 and earlier formats. If you are using a more recent version of MS Excel, try saving your Excel document in a format compatible with Excel 2000, and resubmit to EM.

Other Problems

If you are able to get your Excel document to open with no alert boxes appearing, and you have submitted it in Excel 2000 (or earlier) format, and you still see an error indication in your PDF file (where your Excel document should be appearing). please contact the journal via the 'Contact Us' button on the Navigation Bar.'

You will need to reformat your Excel document, and then re-submit it.

Main figures in PDF format
Click here to access/download;Figure;Main figures.pdf

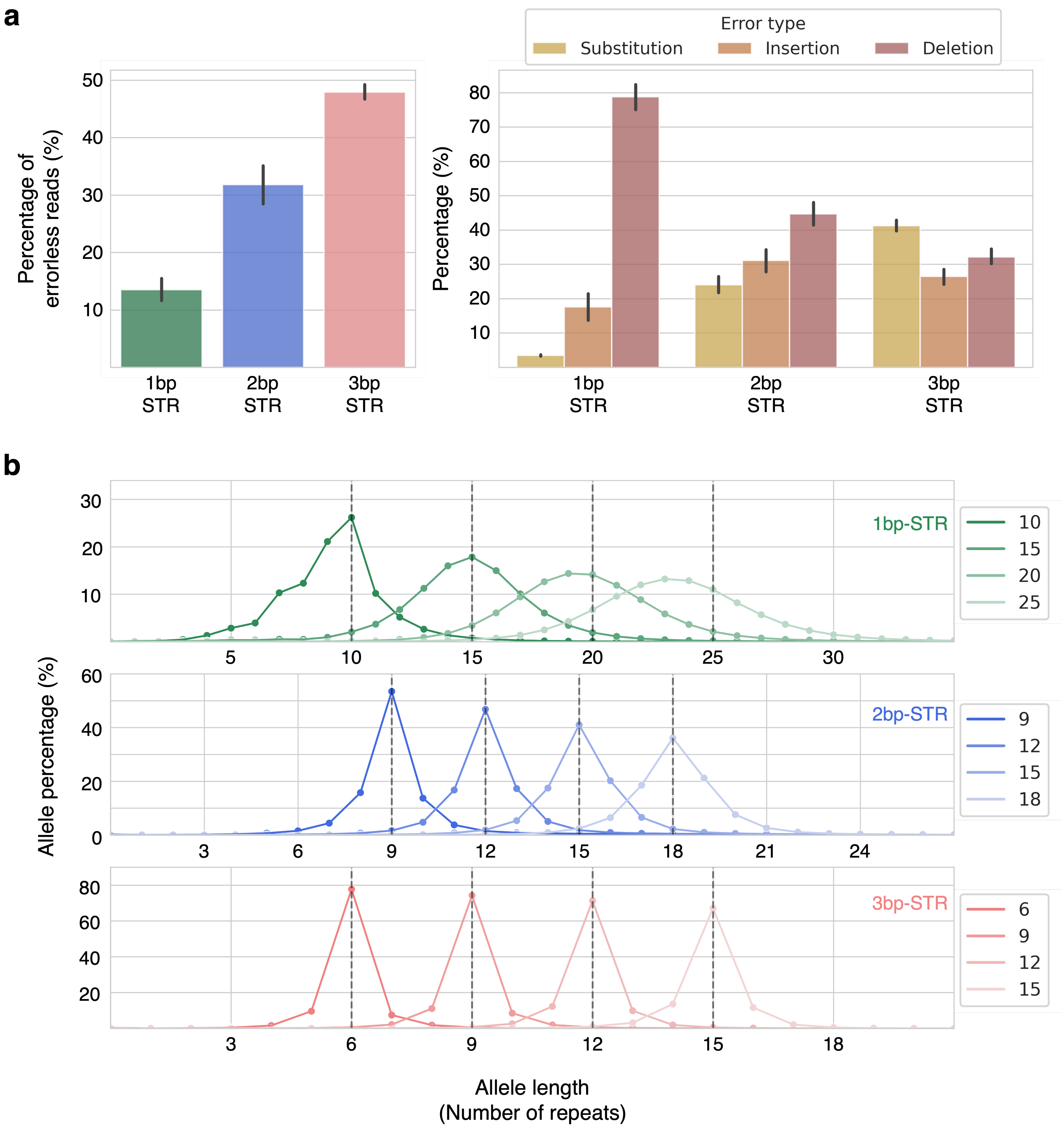

Figure 1. Distribution of ONT sequencing error in STRs.
(a) Percentage of errorless reads in 1bp-, 2bp- and 3bp-repeat STRs (left) and distribution of sequencing error types (right). 1bp-repeats with 10~30 repeats, 2bp-repeats with 7~24 repeats, 3bp-repeats with 5~15 repeats were analyzed.
(b) STR allele size histograms of various lengths of 1bp-, 2bp- and 3bp-repeat STRs.

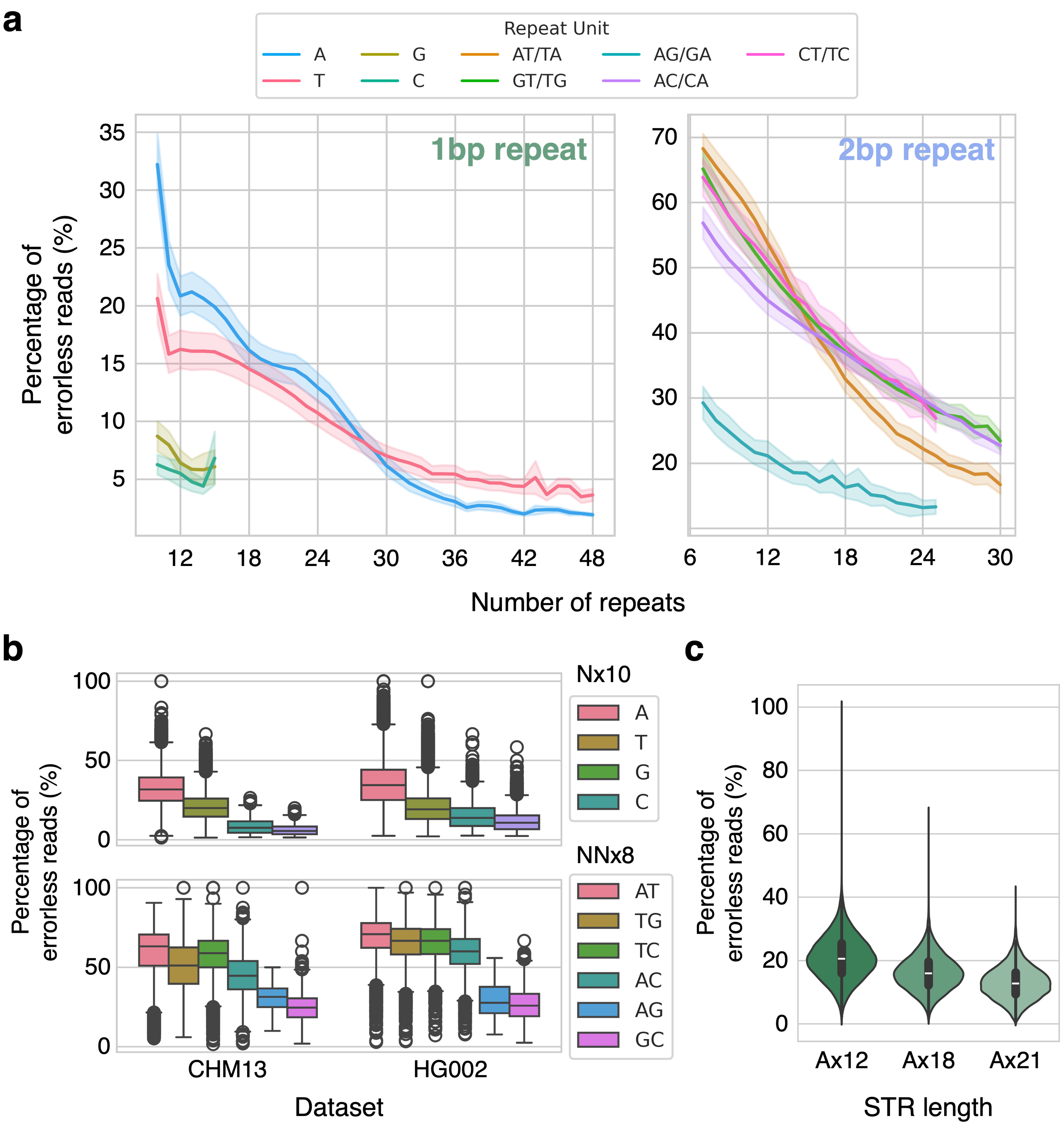

Figure 2. Sequencing accuracy of STRs based on their repeat units.
(a) Sequencing accuracy (measured by calculating the percentage of errorless reads) of varying lengths of 1bp-repeat STRs (left) and 2bp-repeat STRs (right) based on their repeat units. **(b)** Sequencing accuracy of Nx10 and NNx8 STRs of various repeat units, compared between two separate datasets. **(c)** Distribution of sequencing accuracy of Ax12, Ax18 and Ax21 STRs, demonstrating the extreme variability of sequencing accuracy among identical types of STRs.

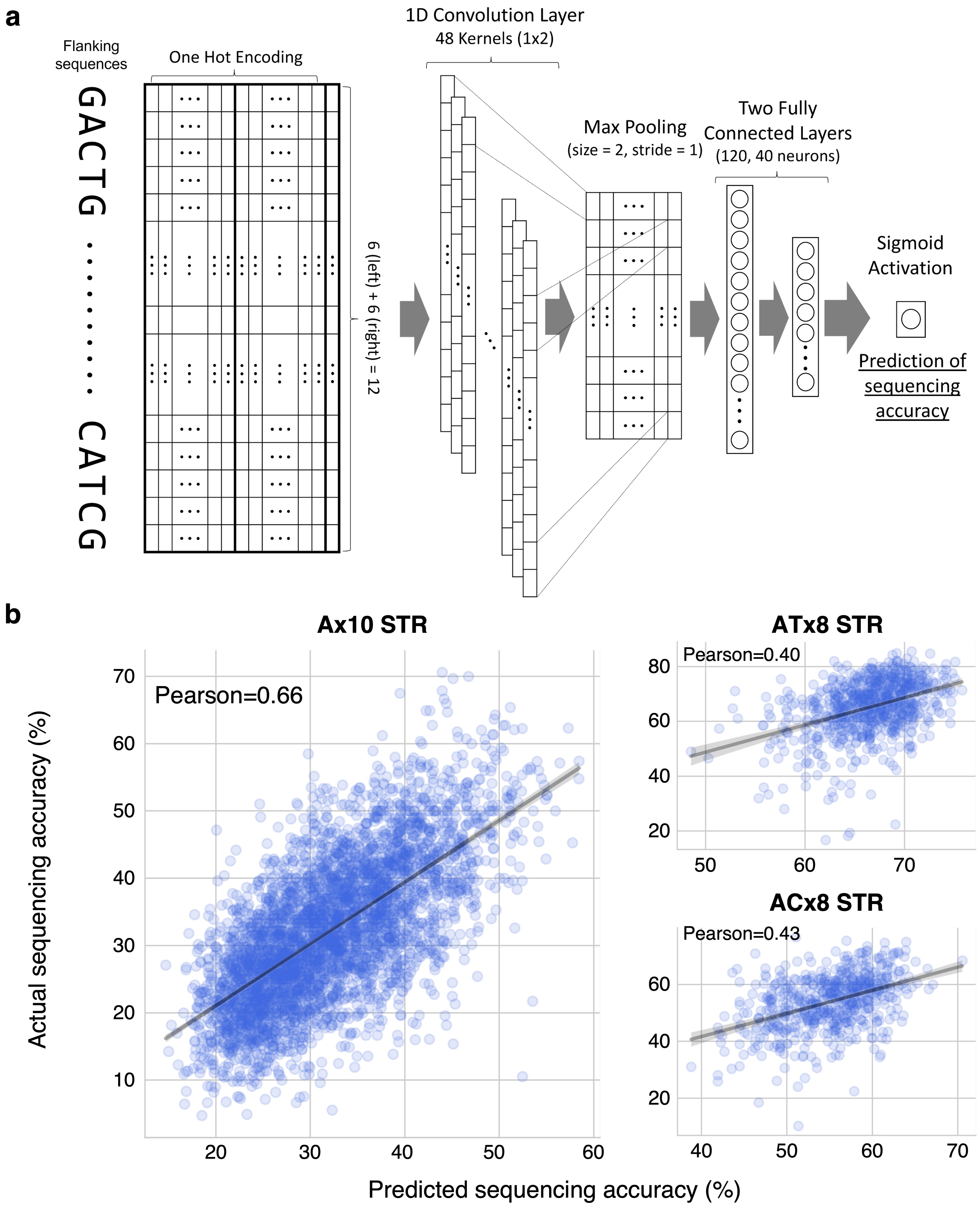

Figure 3. CNN-based machine learning prediction of STR sequencing accuracy using flanking sequences. **(a)** Illustration of CNN-based machine learning prediction workflow. **(b)** Prediction results of Ax10 STRs (left), ATx8 STRs (upper right), and ACx8 STRs (lower right) sequencing accuracy.

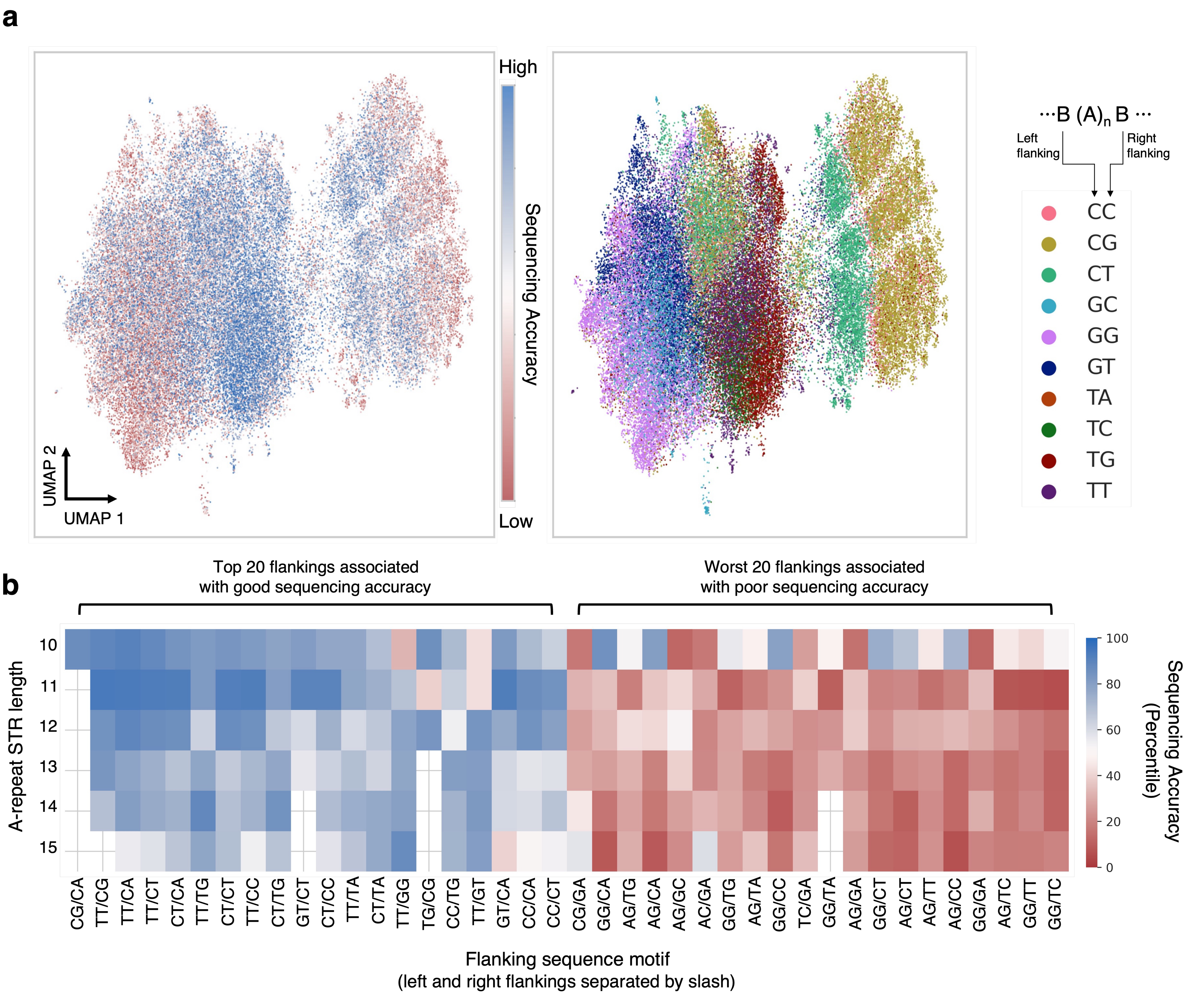

Figure 4. Identification of motifs associated with good/bad sequencing accuracy of A-repeat STRs. **(a)** UMAP projection of flanking sequences of Ax10-Ax15 repeat STRs. Each dot represents an A-repeat STR locus, colored by its sequencing accuracy (left) and by its most adjacent flanking nucleotides (right). **(b)** Flanking sequences of A-repeat STRs (2 nucleotides in each direction, 4 nucleotides total) associated with good and bad sequencing accuracy. 2 nucleotides in each direction are separated by slash (e.g., CT/CG motif indicates CT-$(A)_n$-CG).

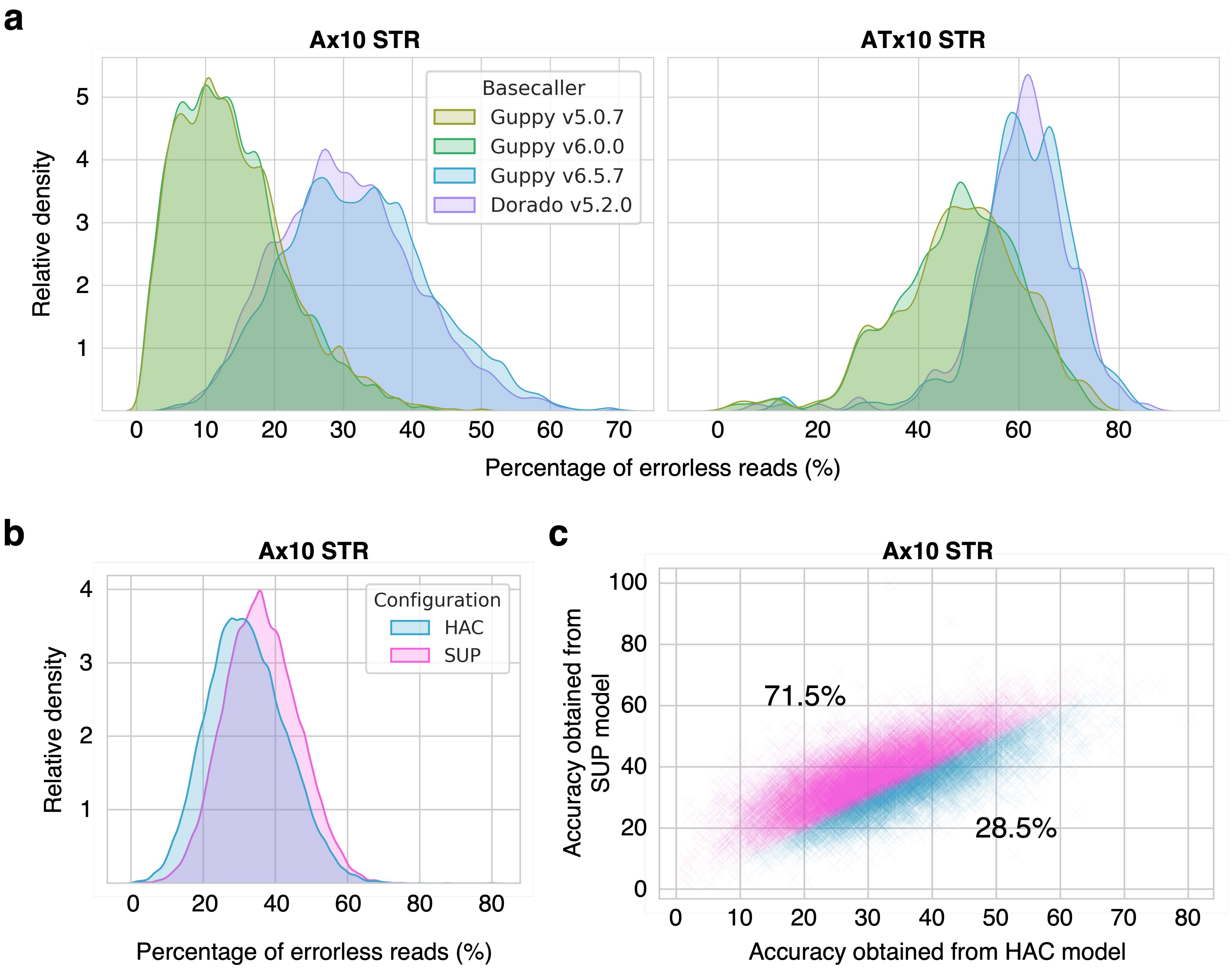

Figure 5. Influence of basecaller on STR sequencing accuracy.
(a) Comparison of 4 ONT basecallers in basecalling Ax10 STRs (left) and ATx10 STRs (right), visualized with kernel density estimate plots. **(b)** Comparison of HAC model and SUP model in basecalling Ax10 STRs, visualized with kernel density estimate plots. Both models are from Guppy v6.5.7. **(c)** Sequencing accuracy of Ax10 STRs obtained by the HAC basecaller model (x-axis) and the SUP basecaller model (y-axis), where each cross represents a single Ax10 STR locus. Loci that exhibited better sequencing accuracy with either SUP basecaller model (71.5%) or HAC basecaller model (28.5%) were marked with different colors.

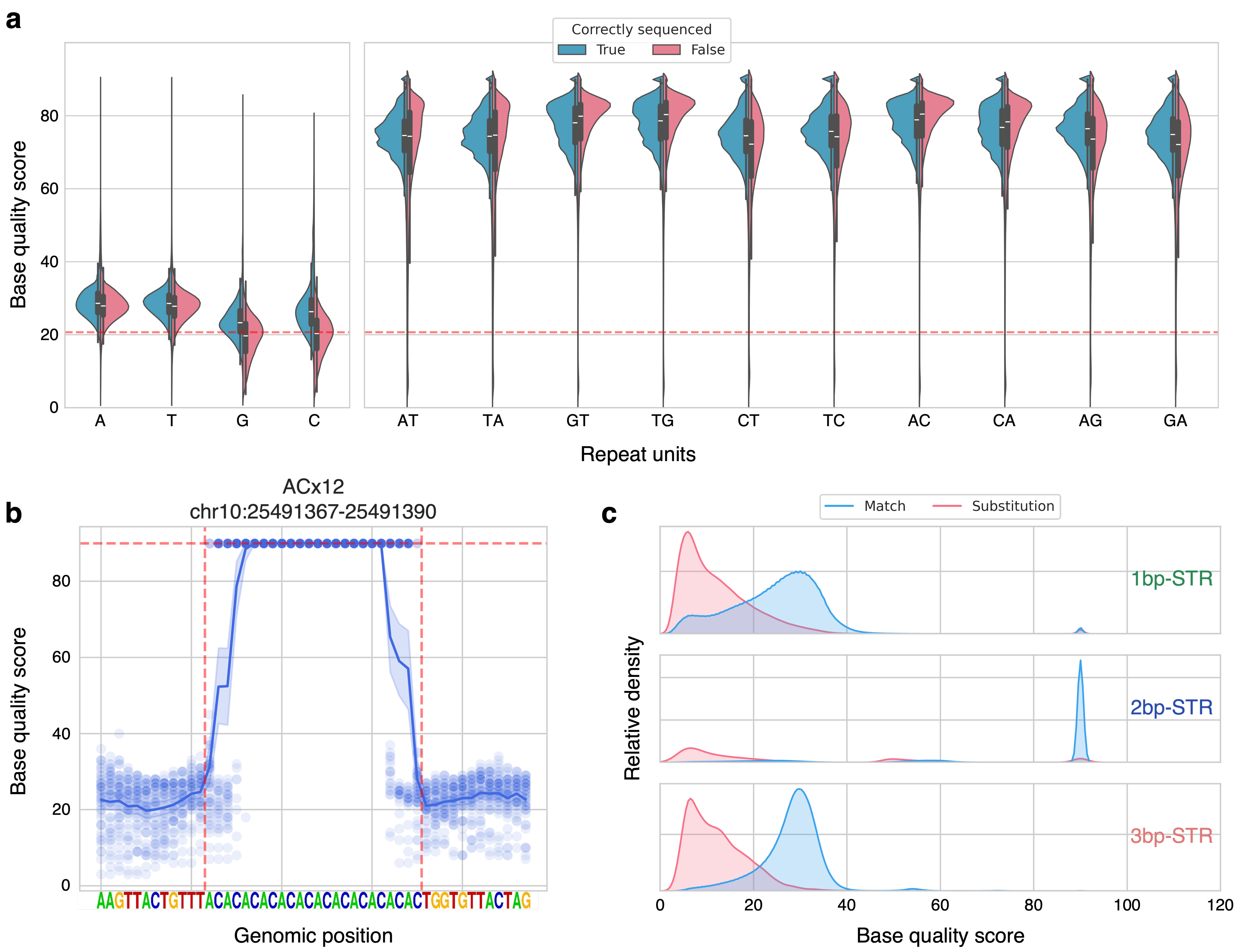

Figure 6. ONT base quality scores in STR regions.
(a) Base quality score comparison between correctly sequenced reads and reads containing sequencing error across various STR types. The red dashed horizontal line indicates the estimated base quality average across the entire CHM13 dataset (20.7). **(b)** Base quality score distribution in an ACx12 STR locus (chr10:25491367-25491390, T2T-CHM13 v2.0). Each dot represents the base quality score reported by a single read and the line represents the average score of each genomic position. **(c)** Base quality score comparison of correctly sequenced bases and substitution error bases, visualized with kernel density estimate plots.

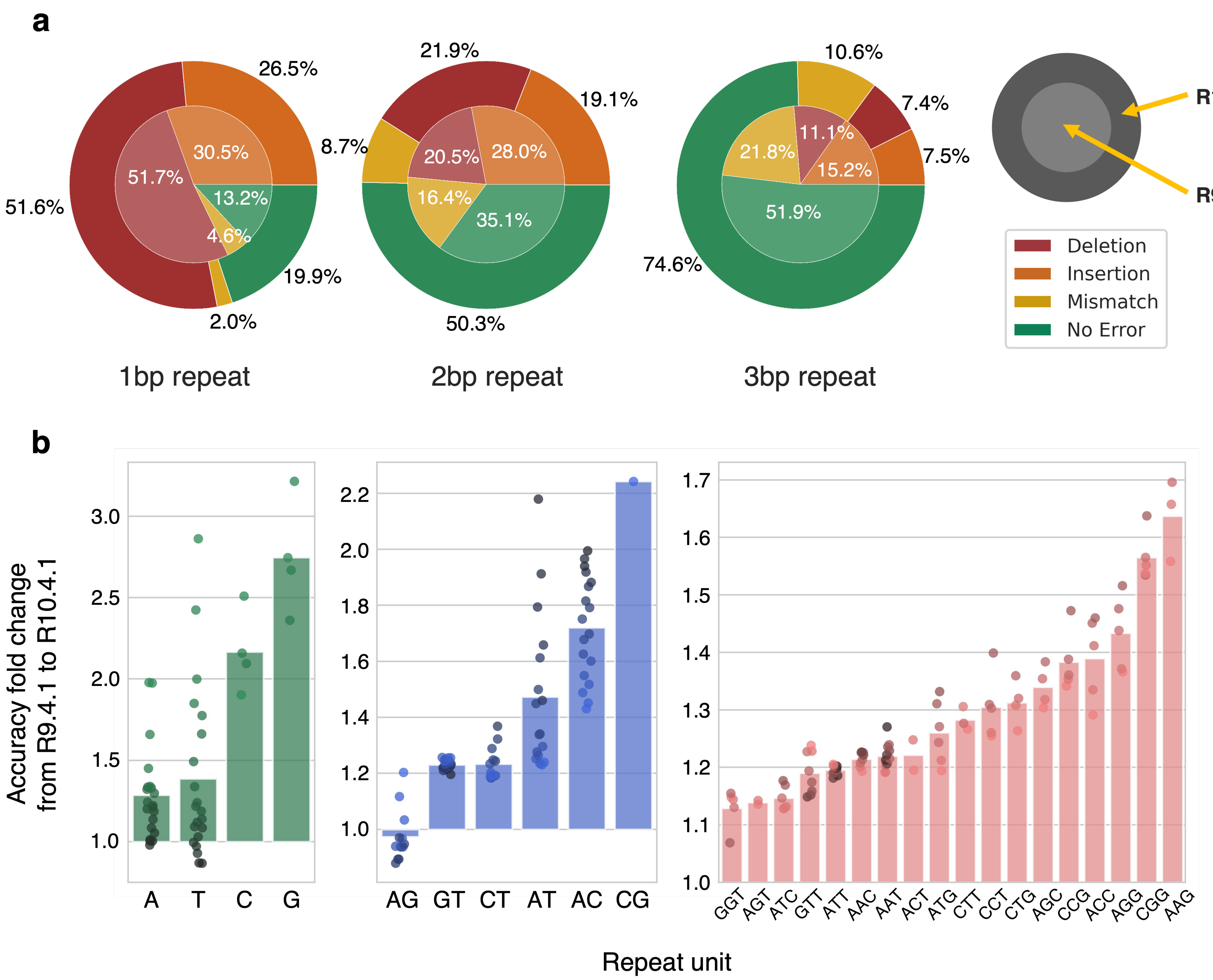

Figure 7. Comparison of ONT STR sequencing profile between the R9.4.1 and the R10.4.1 flowcell. **(a)** Distribution of correctly sequenced reads and various types of sequencing errors in 1bp-, 2bp- and 3bp-repeat STRs, compared between the R9.4.1 (HG002 R9.4.1 dataset) and the R10.4.1 (HG002 R10.4.1 dataset) flowcell. **(b)** Changes in sequencing accuracy from R9.4.1 to R10.4.1 of various types of STR. Accuracy change (y-axis) is measured by dividing the sequencing accuracy observed in R9.4.1 with the sequencing accuracy of R10.4.1 (e.g., 2.0 indicates 200% improvement). The dots in each bar plot represents STRs of different lengths, with longer STR represented by darker colors.

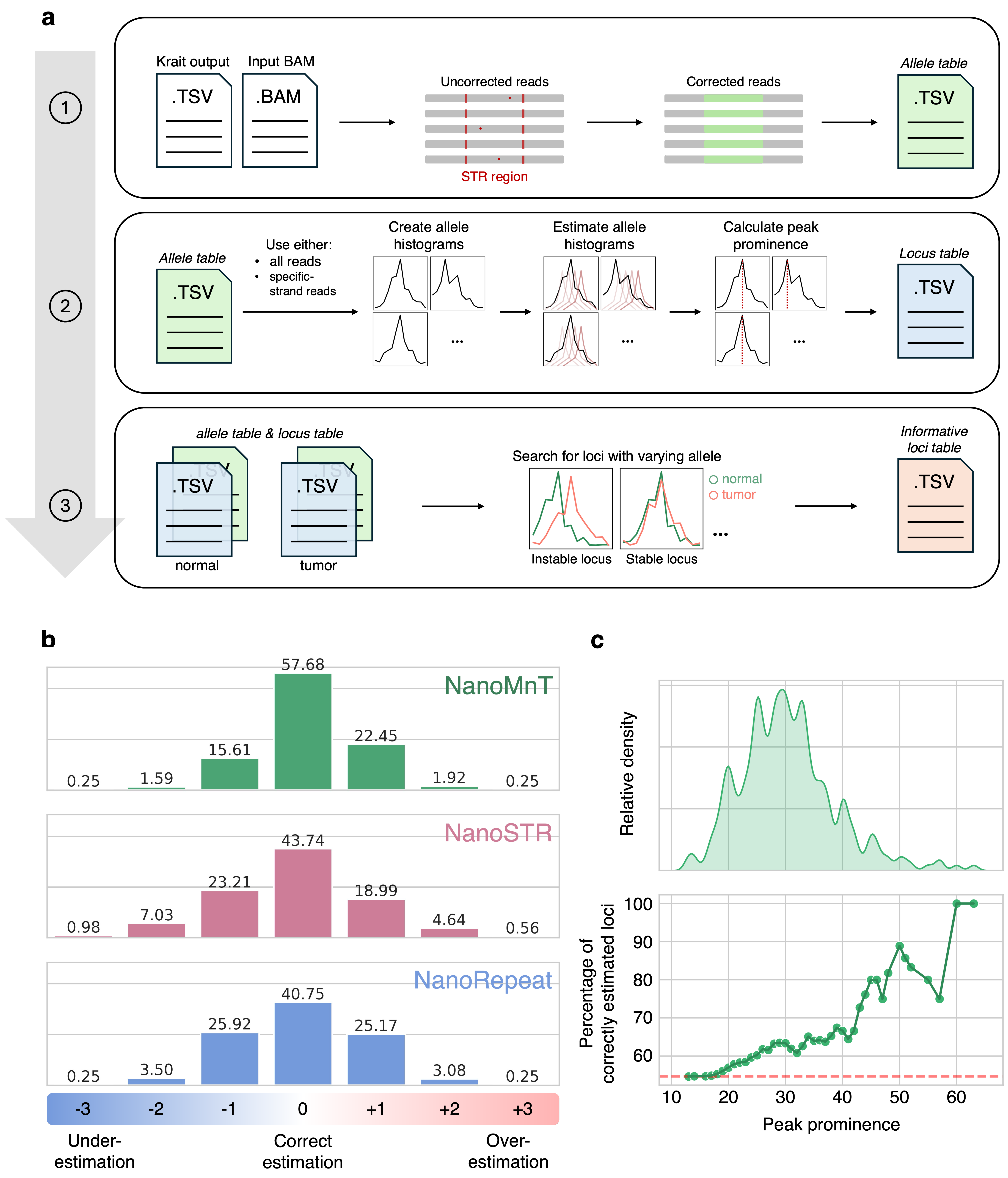
a
1
Krait output
.TSV
Input BAM
.BAM
Uncorrected reads
STR region
Corrected reads
Allele table
.TSV
2
Allele table
.TSV
Use either:
• all reads
• specific-strand reads
Create allele histograms
Estimate allele histograms
Calculate peak prominence
Locus table
.TSV
3
allele table & locus table
.TSV
normal
.TSV
tumor
Search for loci with varying allele
normal
tumor
Instable locus
Stable locus
Informative loci table
.TSV
b
NanoMnT
0.25
1.59
15.61
57.68
22.45
1.92
0.25
NanoSTR
0.98
7.03
23.21
43.74
18.99
4.64
0.56
NanoRepeat
0.25
3.50
25.92
40.75
25.17
3.08
0.25
-3
-2
-1
0
+1
+2
+3
Under-estimation
Correct estimation
Over-estimation
c
Relative density
Percentage of correctly estimated loci
100
90
80
70
60
10
20
30
40
50
60
Peak prominence

Figure 8. Development of NanoMnT.
(a) Schematic overview of NanoMnT functionality. First, NanoMnT performs rudimentary STR error correction in read level and generates a tab-delimited file (TSV) named Allele Table. The Allele table is then used to estimate STR allele sizes of user-specified STR loci by comparing the observed STR allele size histogram against many synthetic STR allele size histograms, where the synthetic histogram with the most resemblance is chosen as the putative allele histogram. This process generates another TSV file, called the Locus Table. Given the Allele Table and the Locus Table of paired normal and tumor samples, NanoMnT compares the STR loci captured in both samples, to search for loci that may provide useful information regarding the tumor's MSI status. **(b)** Benchmark results of NanoMnT, NanoSTR and NanoRepeat in estimating STR allele sizes of 300 1bp-repeat STR loci. **(c)** Distribution of peak prominence value (which indicates the prominence of the STR allele size histogram of each STR locus, calculated using SciPy *find_peaks* function) reported by NanoMnT (top), and the change in percentage of STR loci whose allele size have been correctly estimated by thresholding peak prominence (bottom). For example, ~90% of STR loci whose peak prominences exceed 50 are correctly estimated. The red dashed horizontal line represents the total percentage of STR loci that have been correctly estimated by NanoMnT, as shown in Figure b.

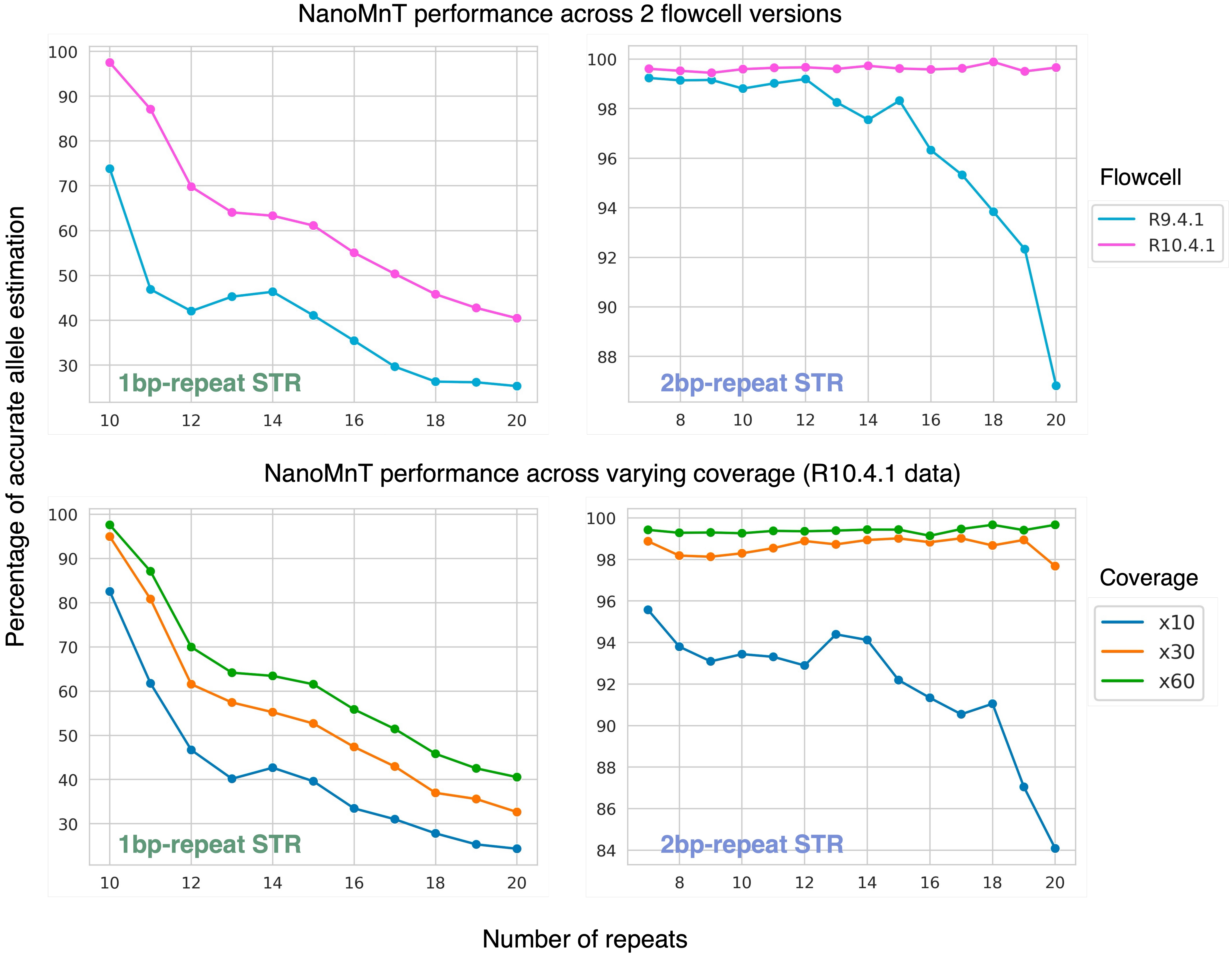

Figure 9. NanoMnT performance across flowcell versions and coverage.
Percentage of correct allele estimation for 1bp-repeat and 2bp-repeat STRs by NanoMnT across varying sequencing coverages and flowcell types. The HG002 R9.4.1 dataset and the HG002 R10.4.1 dataset were employed for this benchmark. The upper two plots compare NanoMnT performance between the two flowcells, while the lower two plots illustrate the impact of sequencing coverage (10x, 30x, 60x) on allele estimation accuracy.

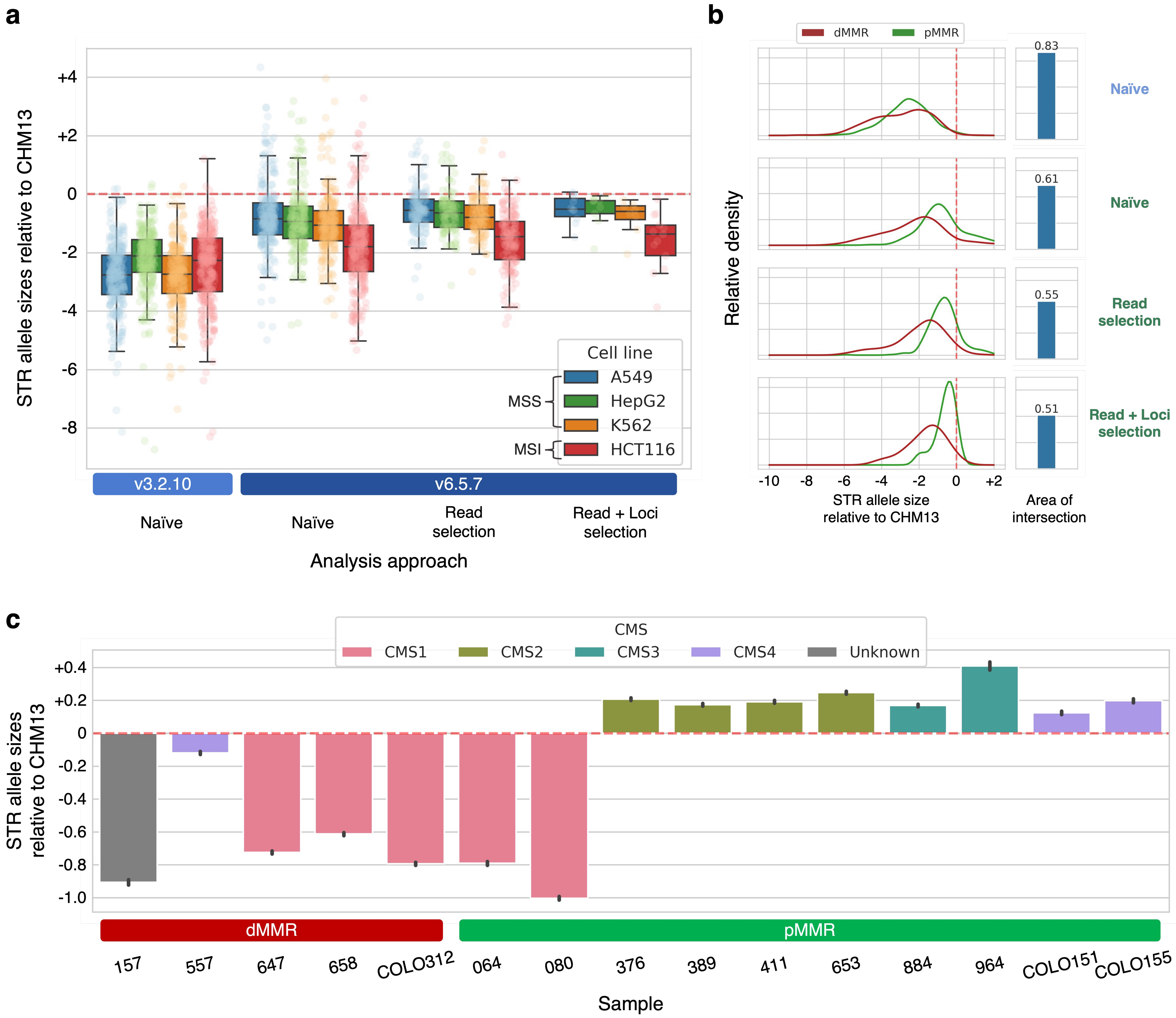

Figure 10. MSI identification results of SG-NEx and CRC organoid WGS sequencing data.
(a) Distribution of STR allele sizes relative to those of the CHM13 genome in 4 cancer cell lines, visualized by box plots and strip plots. 4 analysis approaches are compared; Naïve (Guppy v3.2.10), Naïve (Guppy v6.5.7), read selection approach, and read + loci selection approach. **(b)** Comparison of STR allele size distributions between the MSS cell lines and the MSI cell line when employing the 4 different analysis approaches, visualized by kernel density estimate plots (left) and the area of intersection between the kernel density estimate plots of MSS and MSI. The red dashed vertical lines in the left figure represents the reference STR allele size (zero). **(c)** Average STR allele sizes of 15 CRC samples visualized by bar plots, with each sample colored by its reported CMS type.

Supplementary figures in PDF format
Click here to access/download;Figure;Supplementary Figures.pdf

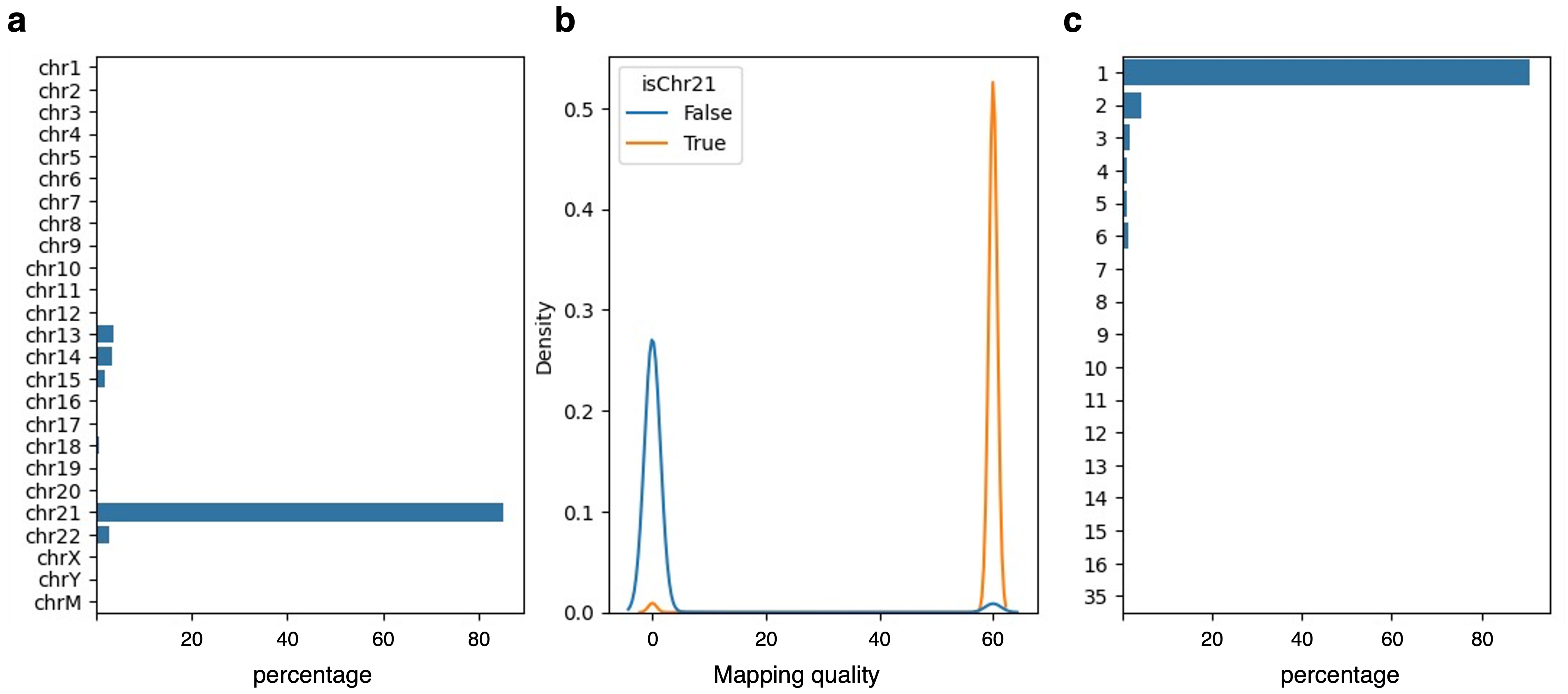

Supplementary Figure 1.
(a) Percentage of chromosomes which reads (of the CHM13 dataset, whose primary alignments aligned to chromosome 21) realigned to. **(b)** Mapping quality distribution of two groups of the realigned reads: reads that mapped back to chromosome 21 and reads that mapped elsewhere. **(c)** Distribution of the number of alignments per read. E.g., 1 indicates that a read aligned to a single region, and 2 indicates that a read aligned to two different regions.

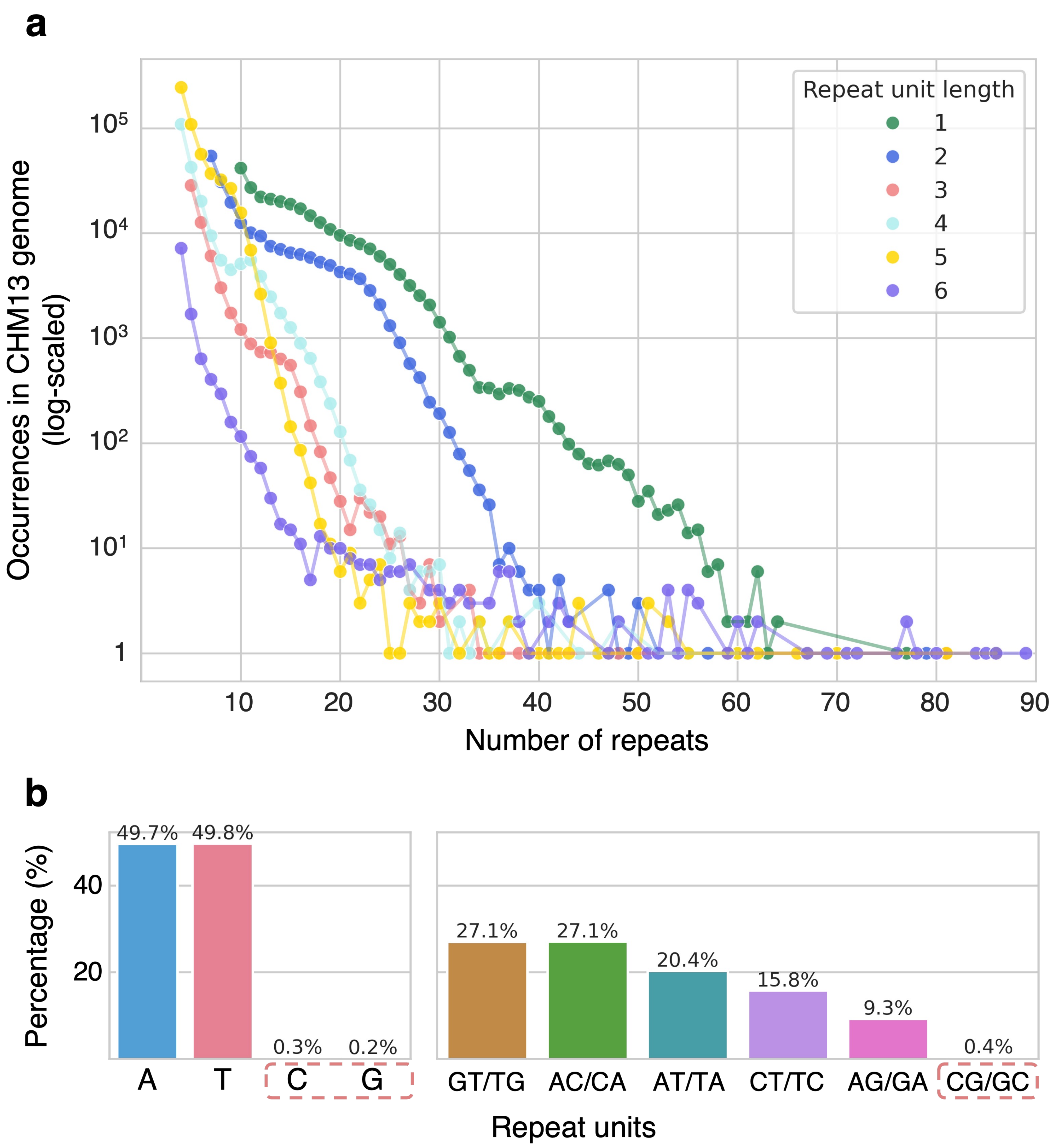

Supplementary Figure 2.
(a) Number of STRs analyzed in this study. **(b)** Percentage of 1bp-/2bp-repeat STRs by repeat units.

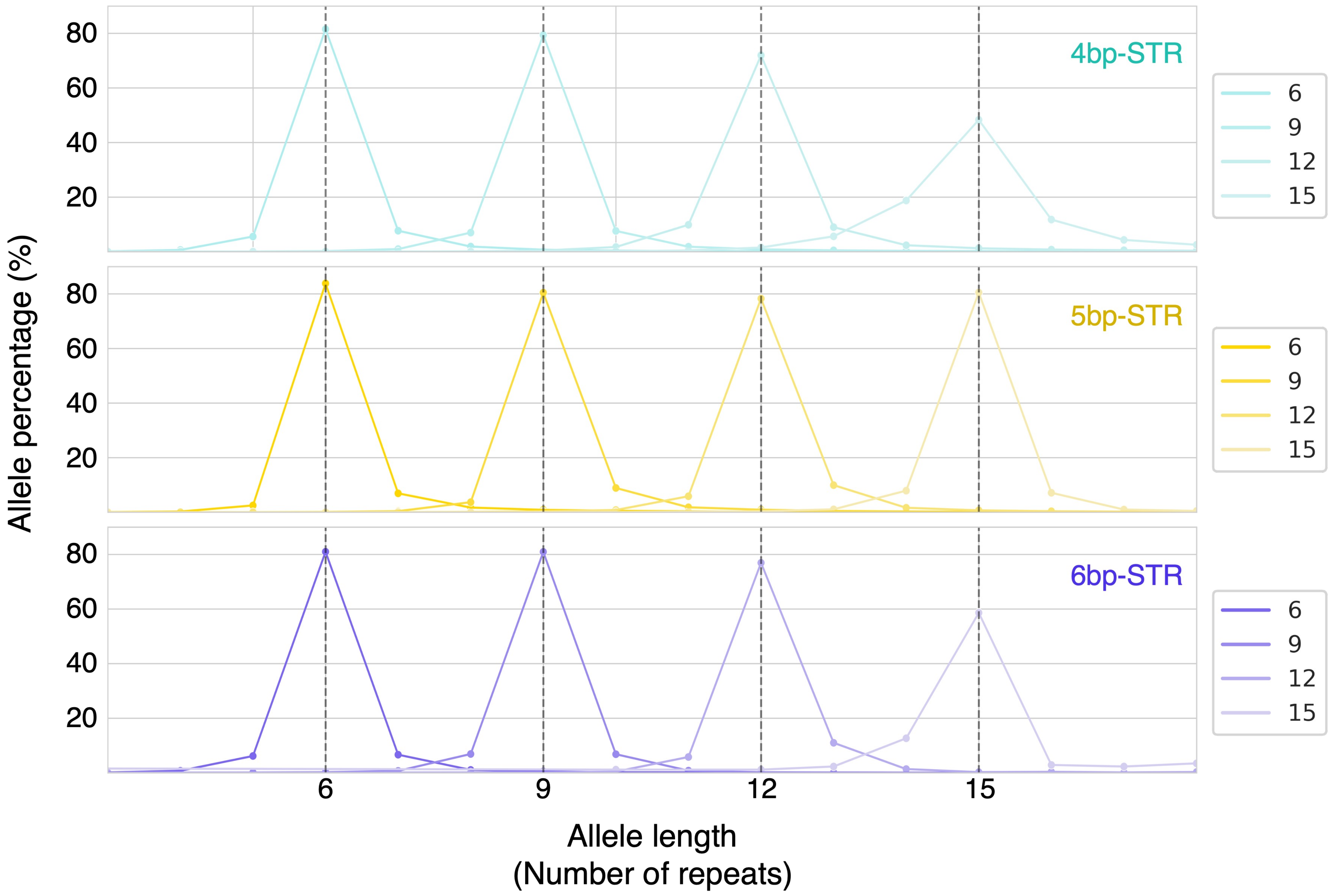

Supplementary Figure 3.
STR allele size histograms of various lengths of 4bp-, 5bp- and 6bp-repeat STR.

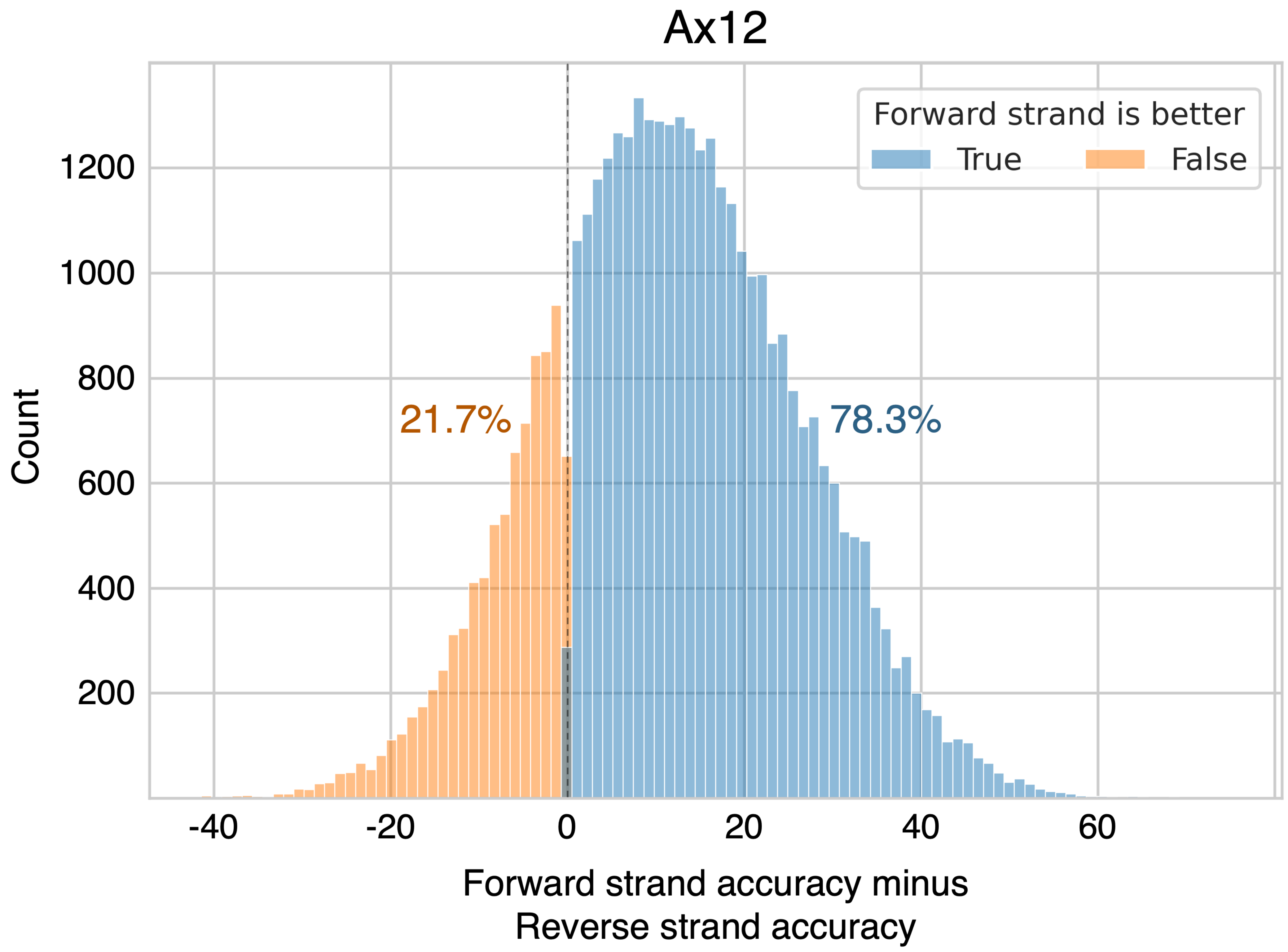

Supplementary Figure 4.
Distribution of forward strand sequencing accuracy (i.e., sequencing accuracy calculating using only forward strand reads) minus reverse strand sequencing accuracy in Ax12 STRs. 21.7% of Ax12 STR loci exhibited better sequencing accuracy when using reverse strand reads, while the remaining 78.3% of Ax12 STR loci exhibited the opposite.

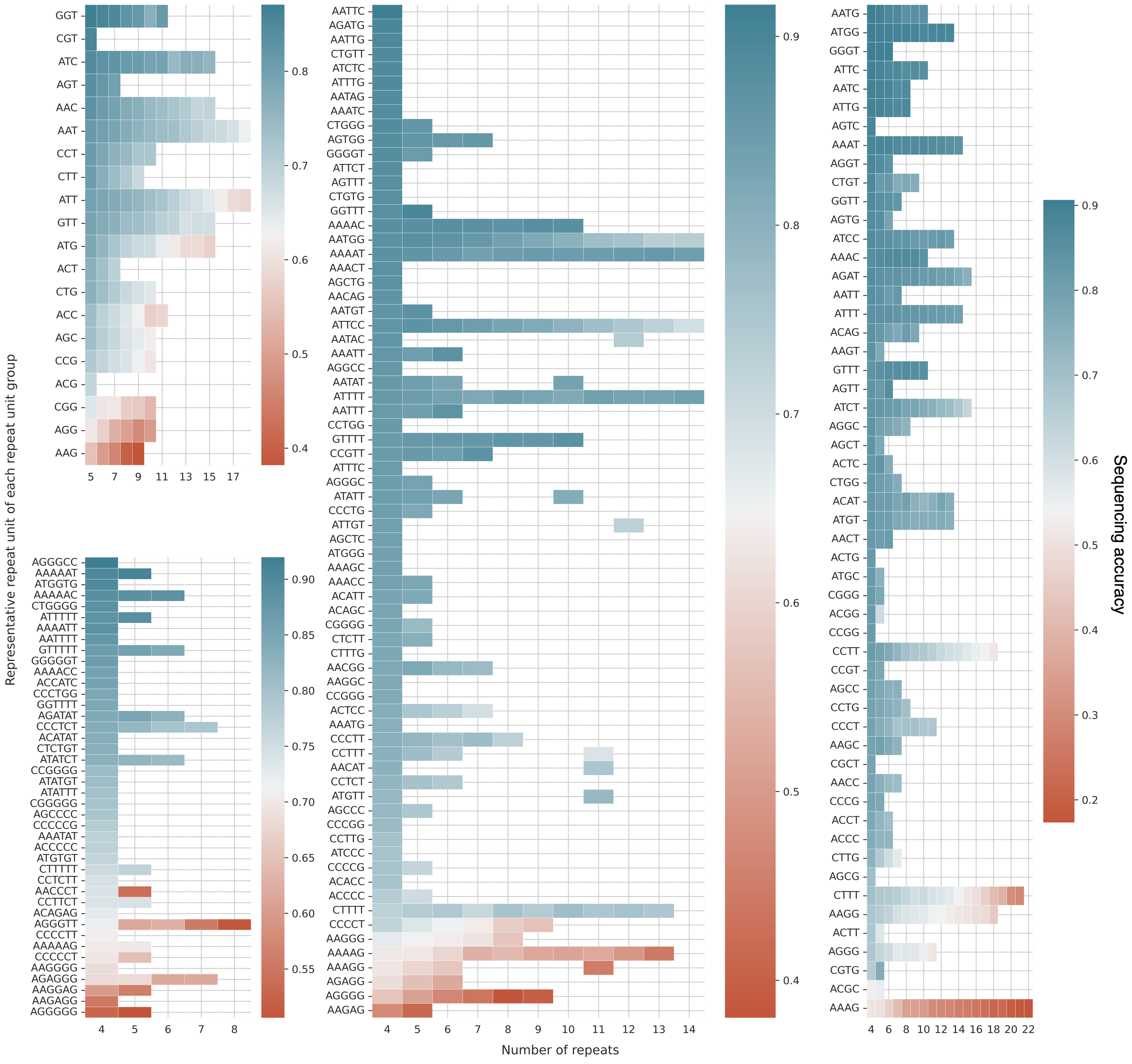

Supplementary Figure 5.
Sequencing accuracy of 3bp-, 4bp-, 5bp- and 6bp-repeat STRs. Synonymous STR types (e.g., ACG-, CGA-, GAC-repeats) were grouped together and represented by the ‘representative’ repeat unit (e.g., ACG-repeat). STRs with at least 30 observations were included in this plot. The colorbar next to each figure represents the sequencing accuracy.

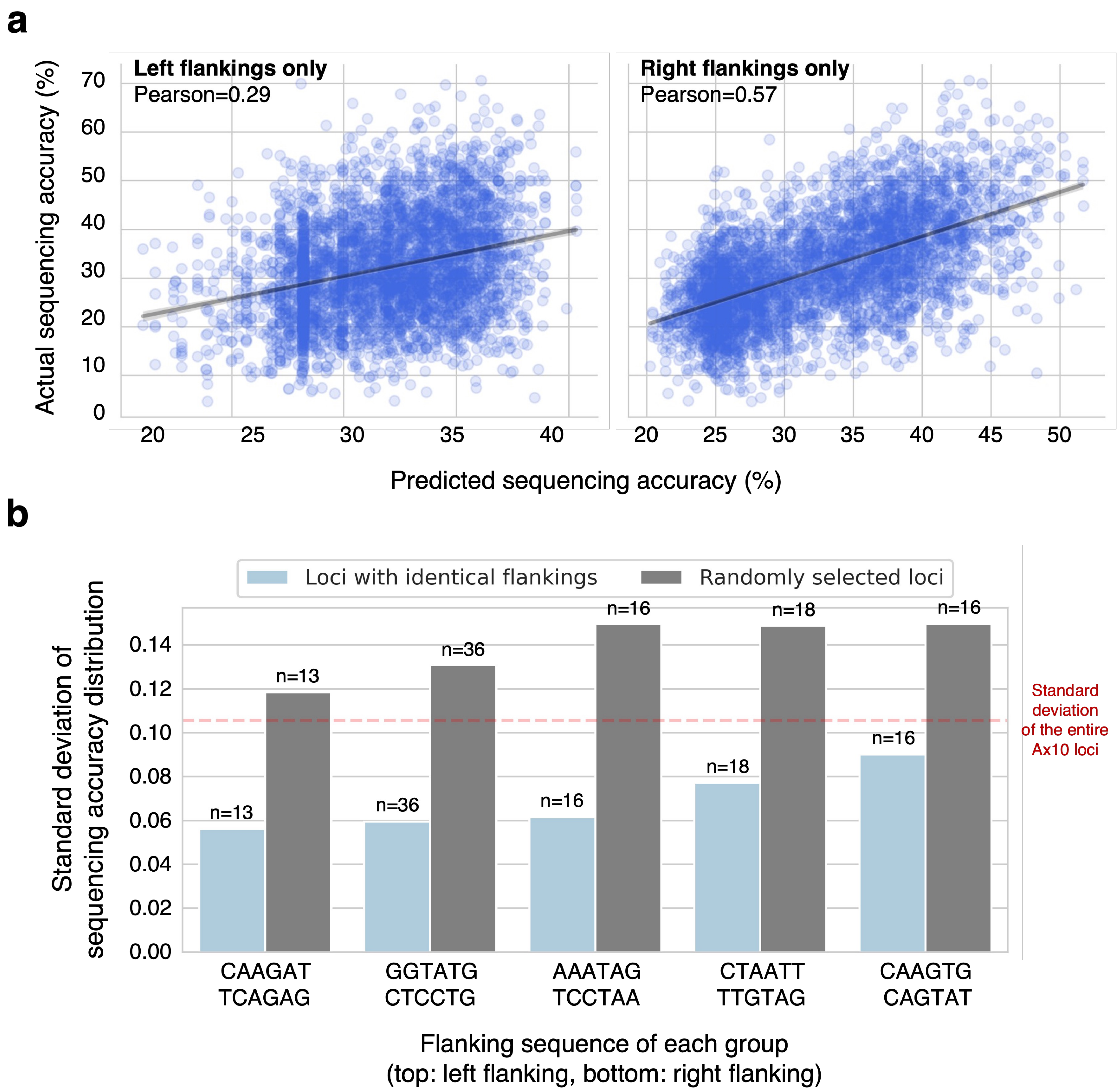

Supplementary Figure 6.
(a) Prediction of sequencing accuracy of Ax10 STRs using left or right flanking sequences as inputs. **(b)** Standard deviation of sequencing accuracy of Ax10 STRs that share the identical flanking sequences, compared against randomly sampled Ax10 STRs, demonstrating that flanking sequences indeed influence the sequencing accuracy of A-repeat STRs.

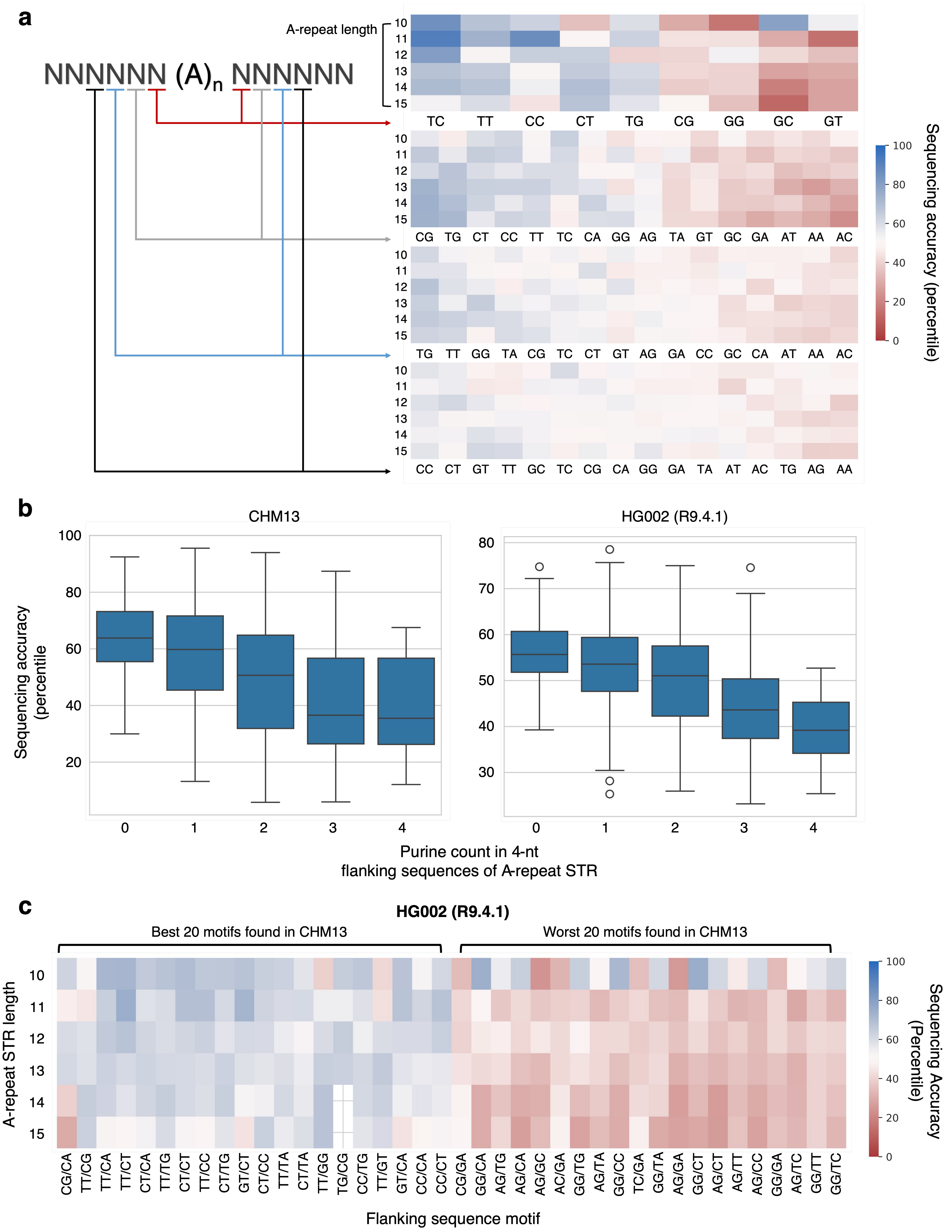
a
A-repeat length
NNNNNN (A)n NNNNNN
TC TT CC CT TG CG GG GC GT
CG TG CT CC TT TC CA GG AG TA GT GC GA AT AA AC
TG TT GG TA CG TC CT GT AG GA CC GC CA AT AA AC
CC CT GT TT GC TC CG CA GG GA TA AT AC TG AG AA
Sequencing accuracy (percentile)
b
CHM13
HG002 (R9.4.1)
Sequencing accuracy (percentile
Purine count in 4-nt flanking sequences of A-repeat STR
c
HG002 (R9.4.1)
Best 20 motifs found in CHM13
Worst 20 motifs found in CHM13
A-repeat STR length
CG/CA TT/CG TT/CA TT/CT CT/CA TT/TG CT/CT TT/CC CT/TG GT/CT CT/CC TT/TA CT/TA TT/GG TG/CG CC/TG TT/GT GT/CA CC/CA CC/CT CG/GA GG/CA AG/TG AG/CA AG/GC AC/GA GG/TG AG/TA GG/CC TC/GA GG/TA AG/GA GG/CT AG/CT AG/TT AG/CC GG/GA AG/TC GG/TT GG/TC
Flanking sequence motif
Sequencing Accuracy (Percentile)

Supplementary Figure 7.
(a) Sequencing accuracy of A-repeat STRs that possesses specific pairs of nucleotides in specific distances within their flanking sequences. The influence of nucleotide pair on sequencing accuracy is proportionate to its proximity to A-repeat STR. **(b)** Sequencing accuracy of A-repeat STRs based on the number of purine counts in their flanking sequences of 4 nucleotides (2 nucleotide in each direction) **(c)** Sequencing accuracy of A-repeat STRs measured from the HG002 R9.4.1 dataset. A-repeat STRs that were flanked by the motifs identified in the CHM13 dataset (see the x-axis of Figure 4b) were shown.

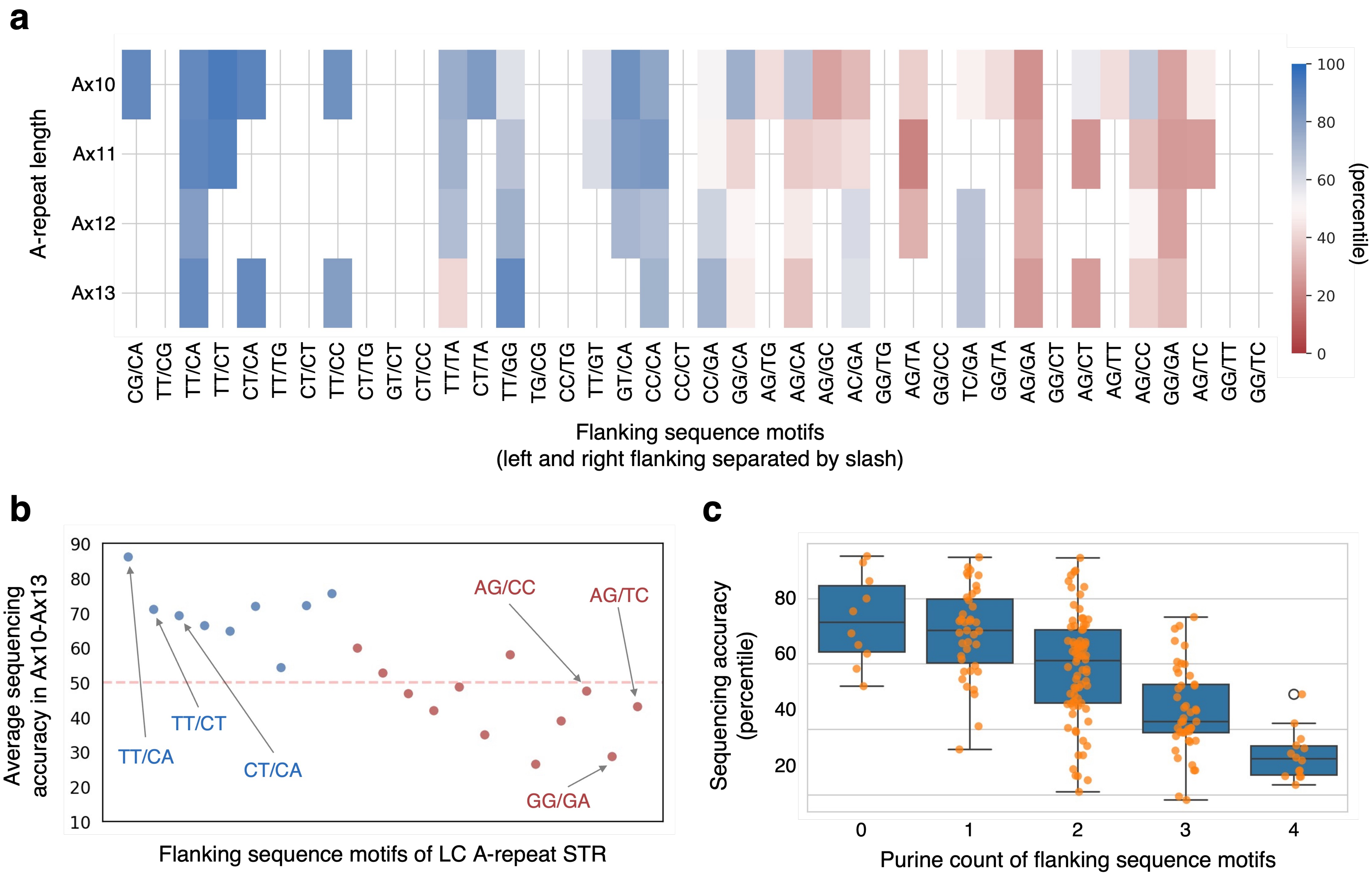

Supplementary Figure 8.

(a) Sequencing accuracy (percentage of errorless reads) of A-repeat STRs with low-complex (LC) flanking sequences, that are flanked by motifs shown in Figure 4b. Each column represents a motif that flank the A-repeat STR, and each row represents the sequencing accuracy of A-repeat STRs with different numbers of repeats. **(b)** Average sequencing accuracy of Ax10-Ax13 STRs with LC flanking sequences (converted to percentile), where each dot represents A-repeat STRs with different flanking sequence motifs. **(c)** Relationship between purine counts of A-repeat STRs with LC flanking sequences and sequencing accuracy.

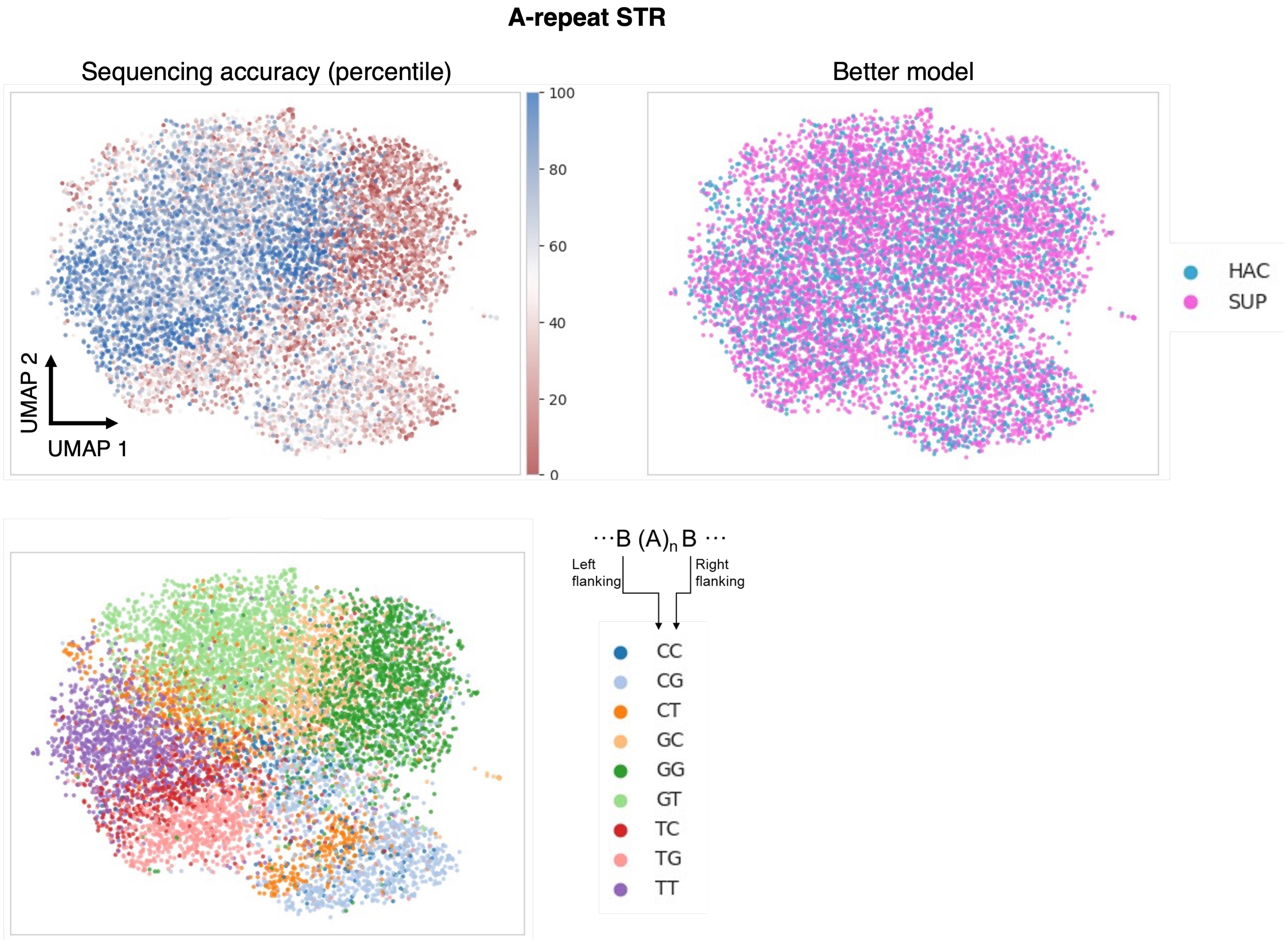

Supplementary Figure 9.
UMAP visualization of A-repeat STRs, created by using flanking sequences as features. Each dot represents a A-repeat locus, colored by sequencing accuracy (upper left), better basecalling model (i.e., model that generated better results for the given locus) (upper right) and the most adjacent flanking sequence (lower left).

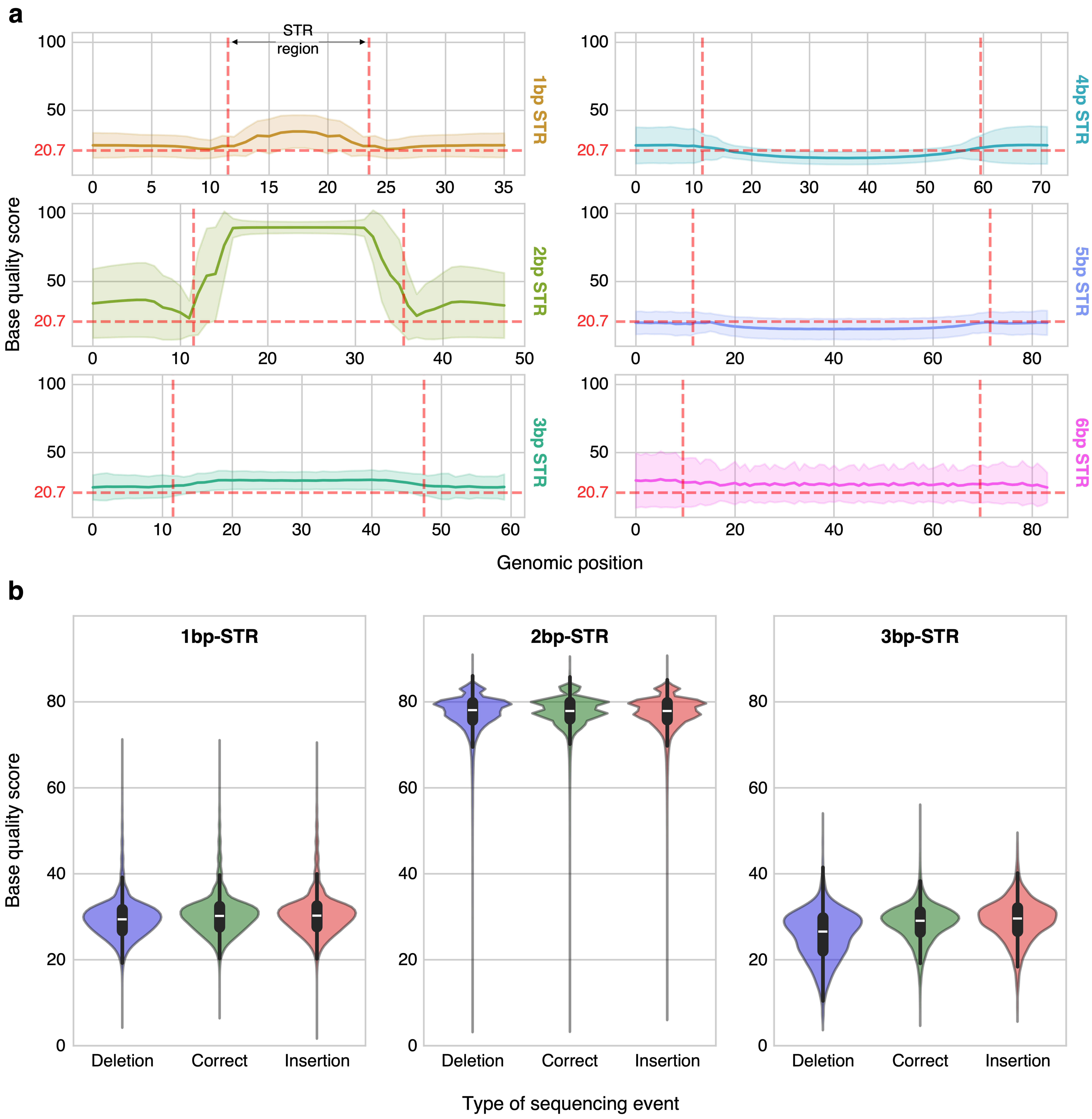

Supplementary Figure 10.
(a) Base quality scores of STRs. The two vertical lines represent the start and end of the repeat sequences, while the horizontal lines represent the average base quality of the CHM13 dataset, highlighting the base quality 'burst' observed within the STR regions of some STR types. **(b)** Average base quality score of reads that are presumed to harbor indel errors in STR regions.

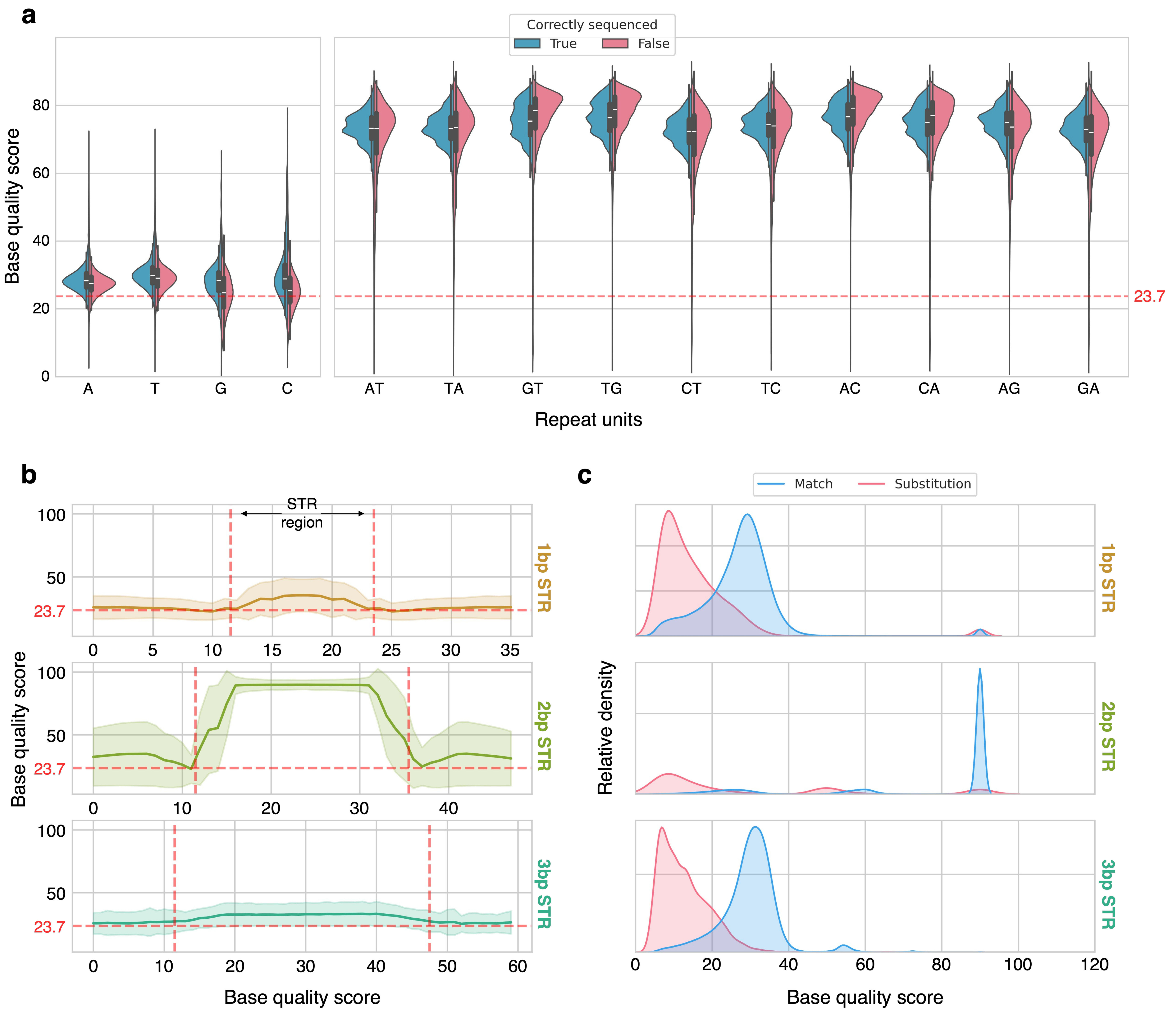

Supplementary Figure 11.
(a) The average base quality of reads in various STR types observed in the HG002 R9.4.1 dataset, comparing correctly sequenced reads (i.e., reads with no error within STR region) against incorrectly sequenced reads. The horizontal line represents the average base quality of the HG002 R9.4.1 dataset. **(b)** The base quality 'burst' observed in the HG002 R9.4.1 dataset. **(c)** Distribution of base quality compared between correctly sequenced bases and substitution errors.

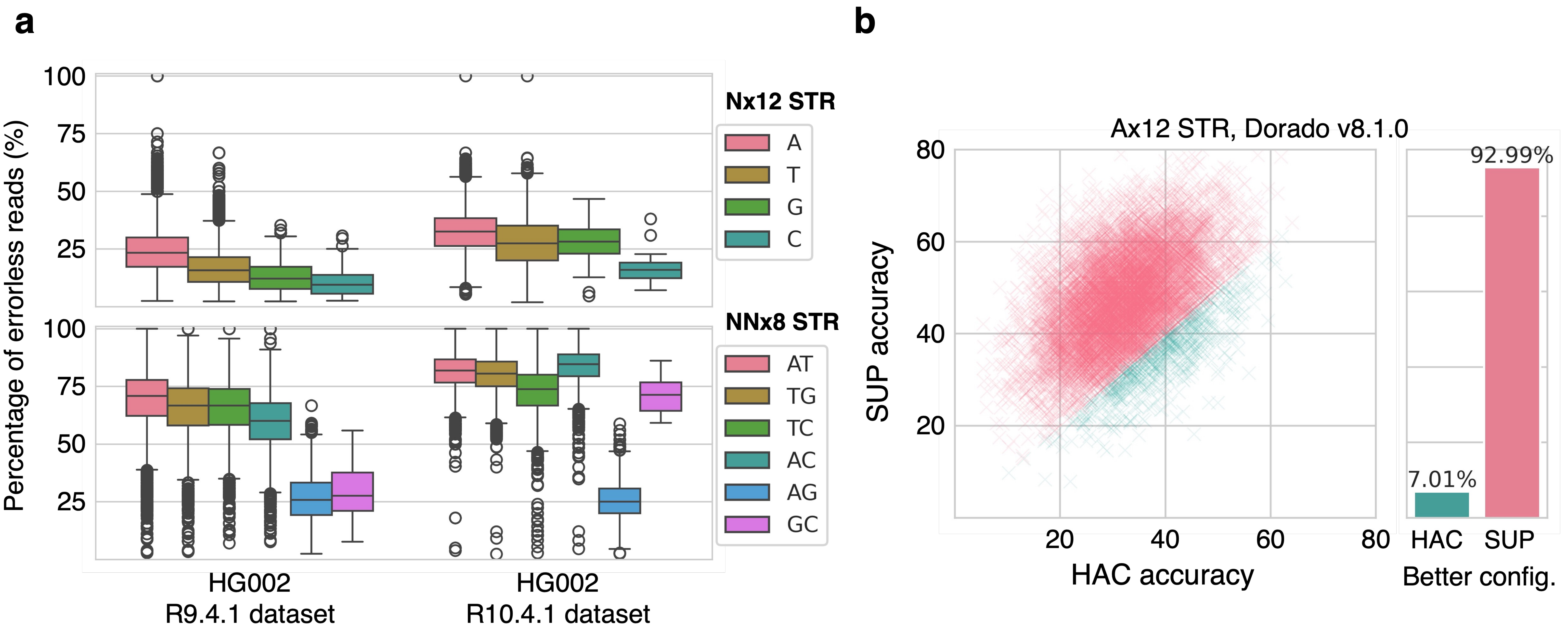

Supplementary Figure 12.
(a) Sequencing accuracy of Nx12 and NNx8 STRs by various repeat units, compared between HG002 R9.4.1 dataset and HG002 R10.4.1 dataset. **(b)** Sequencing accuracy of Ax12 STRs observed in HG002 R10.4.1 dataset, comparing HAC basecalling model against SUP basecalling model. Each cross in the scatterplot (left) represents a Ax12 STR locus. Around 92.99% of Ax12 STRs are better resolved using SUP basecaller model, whereas 7.01% of Ax12 STRs are better resolved using HAC basecaller model (right).

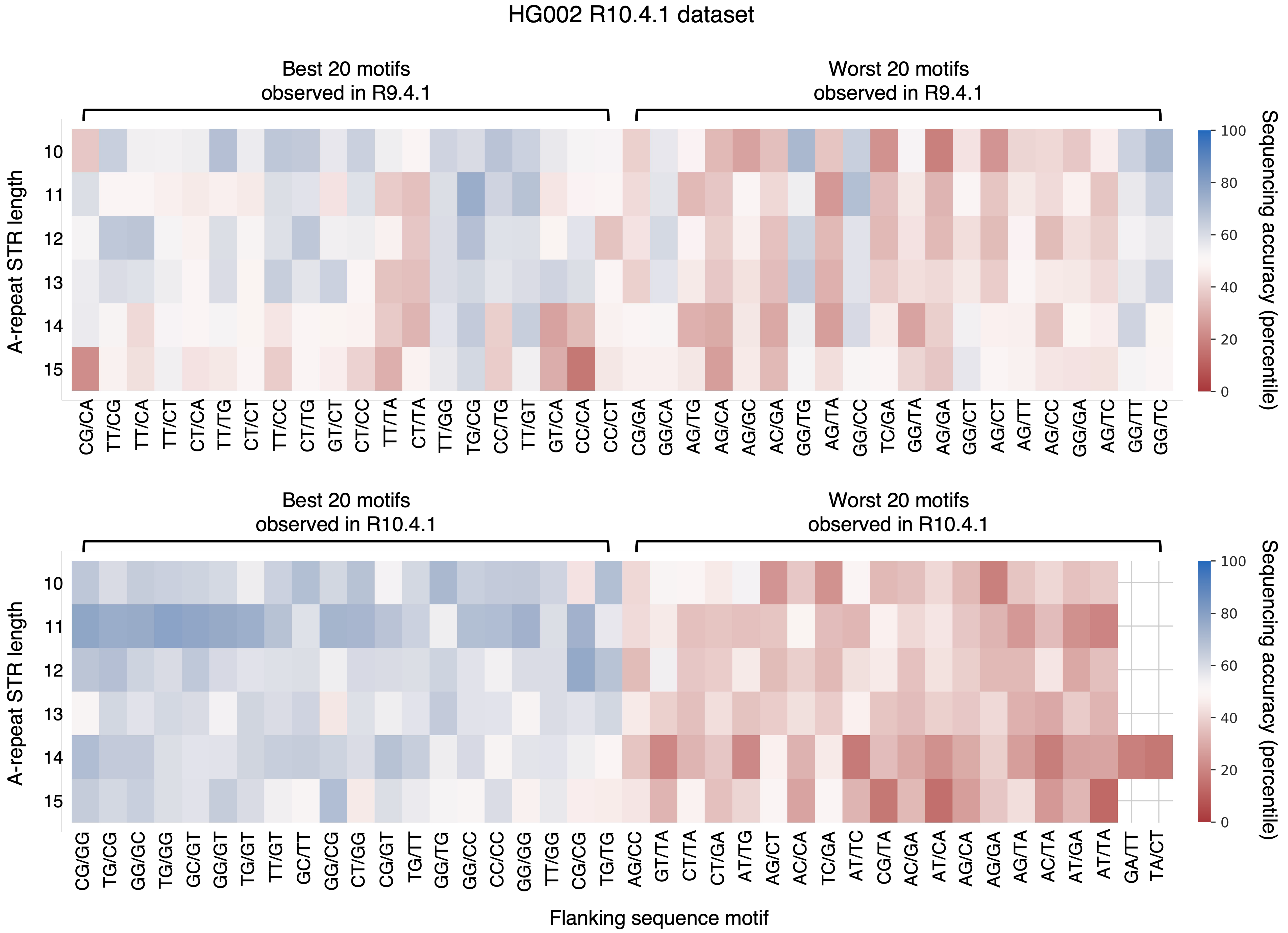

Supplementary Figure 13.

(HG002 R10.4.1 dataset) Sequencing accuracy of A-repeat STRs that harbor certain motifs in their flanking sequences, ordered by the ‘best’ and ‘worst’ motifs found in the CHM13 dataset (top) and the HG002 R10.4.1 dataset (bottom).

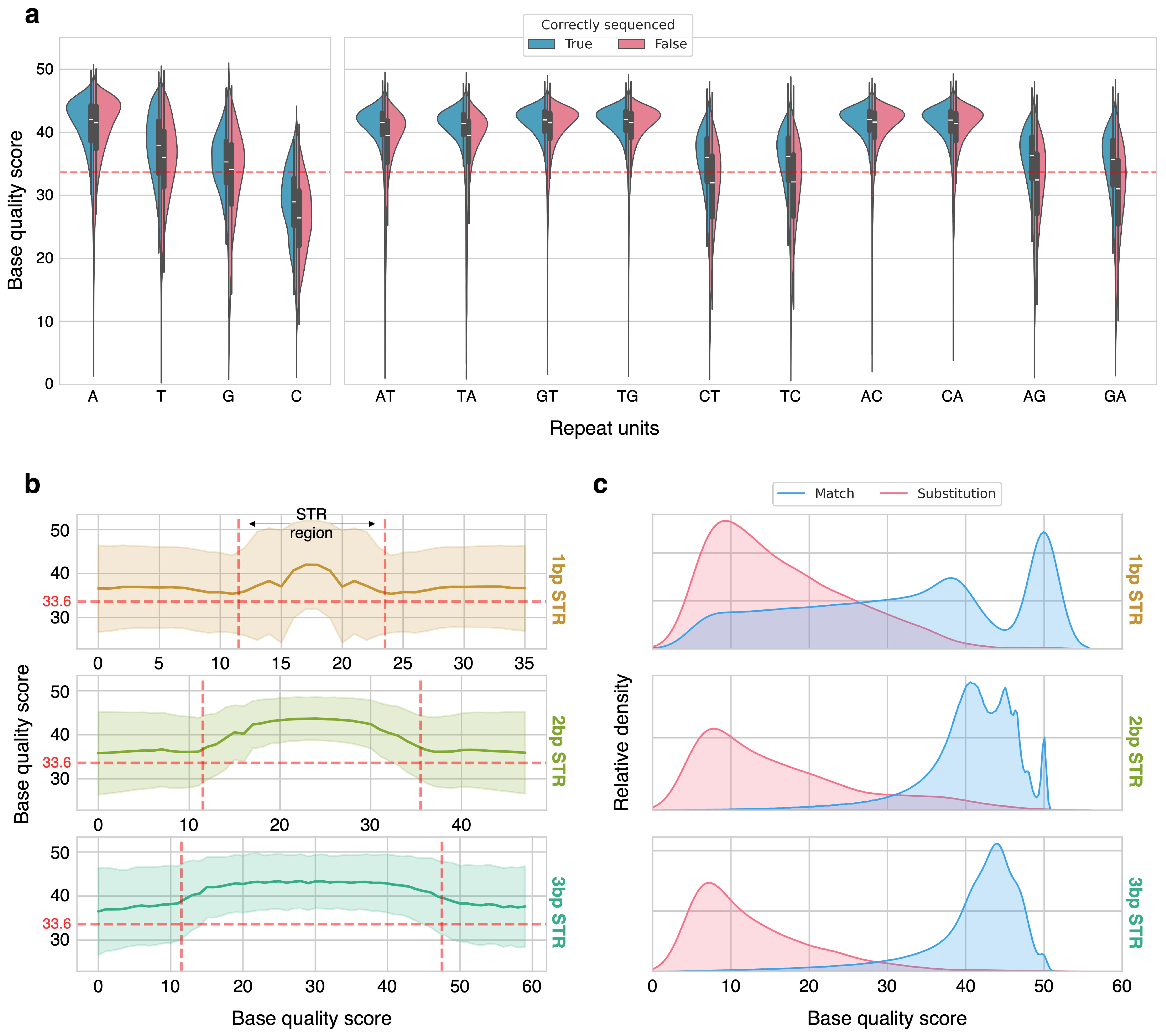

Supplementary Figure 14.
(a) The average base quality of reads in various STR types observed in the HG002 R10.4.1 dataset, comparing correctly sequenced reads (i.e., reads with no error within STR region) against incorrectly sequenced reads. The horizontal line represents the average base quality of the HG002 R10.4.1 dataset. **(b)** The base quality 'burst' observed in the HG002 R10.4.1 dataset. **(c)** Distribution of base quality compared between correctly sequenced bases and substitution errors.

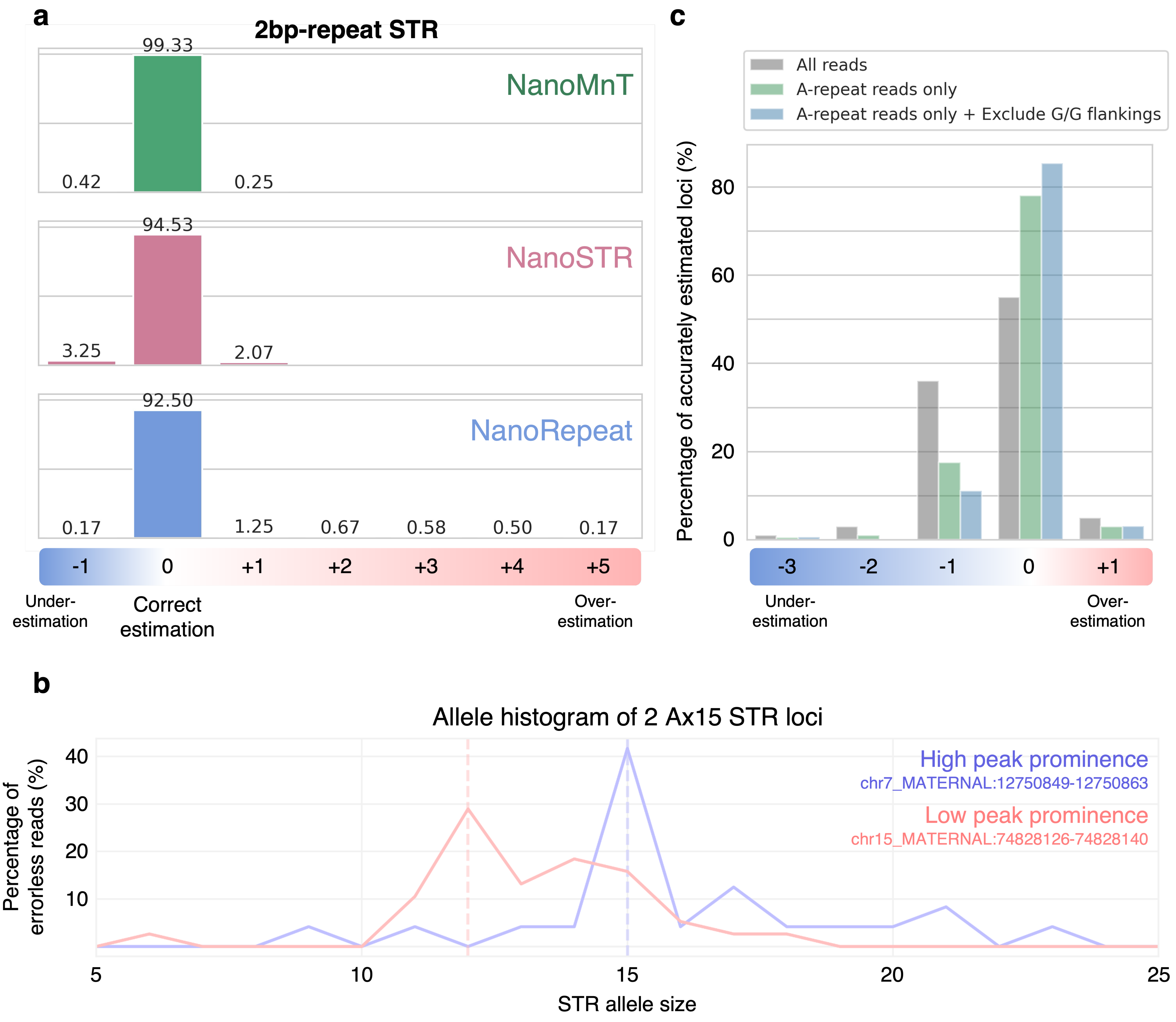

Supplementary Figure 15.
(a) NanoMnT, NanoSTR and NanoRepeat genotyping results of 300 2bp-repeat STR loci. **(b)** STR allele size histograms of 2 example Ax15 loci, one with a highly prominent peak, and the other with a less prominent peak. The dashed line represents the genotyped STR allele for each locus.

SVG format for Supplementary Figure 1

Click here to access/download
Supplementary Material
Supplementary Figure 1.svg

SVG format for Supplementary Figure 2

Click here to access/download
Supplementary Material
Supplementary Figure 2.svg

SVG format for Supplementary Figure 3

Click here to access/download
Supplementary Material
Supplementary Figure 3.svg

SVG format for Supplementary Figure 4

Click here to access/download
Supplementary Material
Supplementary Figure 4.svg

SVG format for Supplementary Figure 5

Click here to access/download
Supplementary Material
Supplementary Figure 5.svg

SVG format for Supplementary Figure 6

Click here to access/download

Supplementary Material

Supplementary Figure 6.svg

SVG format for Supplementary Figure 7

Click here to access/download
Supplementary Material
Supplementary Figure 7.svg

SVG format for Supplementary Figure 8

Click here to access/download
Supplementary Material
Supplementary Figure 8.svg

SVG format for Supplementary Figure 9

Click here to access/download
Supplementary Material
Supplementary Figure 9.svg

SVG format for Supplementary Figure 10

Click here to access/download
Supplementary Material
Supplementary Figure 10.svg

SVG format for Supplementary Figure 11

Click here to access/download
Supplementary Material
Supplementary Figure 11.svg

SVG format for Supplementary Figure 12

Click here to access/download
Supplementary Material
Supplementary Figure 12.svg

SVG format for Supplementary Figure 13

Click here to access/download
Supplementary Material
Supplementary Figure 13.svg

SVG format for Supplementary Figure 14

Click here to access/download
Supplementary Material
Supplementary Figure 14.svg

SVG format for Supplementary Figure 15

Click here to access/download
Supplementary Material
Supplementary Figure 15.svg

SVG format for Figure 1

Click here to access/download
Supplementary Material
Figure 1.svg

SVG format for Figure 10

Click here to access/download
Supplementary Material
Figure 10.svg

SVG format for Figure 2

Click here to access/download
Supplementary Material
Figure 2.svg

SVG format for Figure 3

Click here to access/download
Supplementary Material
Figure 3.svg

SVG format for Figure 4

Click here to access/download
Supplementary Material
Figure 4.svg

SVG format for Figure 5

Click here to access/download
Supplementary Material
Figure 5.svg

SVG format for Figure 6

Click here to access/download
Supplementary Material
Figure 6.svg

SVG format for Figure 7

Click here to access/download
Supplementary Material
Figure 7.svg

SVG format for Figure 8

Click here to access/download
Supplementary Material
Figure 8.svg

SVG format for Figure 9

Click here to access/download
Supplementary Material
Figure 9.svg
