# Supplementary material for: NanoMnT: an STR analysis tool for Oxford Nanopore sequencing data driven by a comprehensive analysis of error profile in STR regions
Source: Gigascience. 2025 Mar 17;14:giaf013. doi: 10.1093/gigascience/giaf013 (PMC11912559; doi:10.1093/gigascience/giaf013)
Supplement: giaf013_Response_to_Reviewer_Comments_Original_Submission [file giaf013_response_to_reviewer_comments_original_submission.pdf]

Author's Response To Reviewer Comments

Dear reviewers

We thank the reviewers very much for their constructive and thoughtful feedbacks. Your comments have been incredibly valuable in helping us improve the quality of our work. We have carefully considered each of your suggestions and have made revisions accordingly. Please see the Revision_Comments.docx file in the attachments for our response for each point. We hope these revisions address your concerns and improve the clarity and quality of our work.

Best regards,
Jihwan Park.
Gwangju Institute of Science and Technology, Associate Professor.

Dear Editor,

Thank you very much for your kind assistance throughout the submission process. We are deeply grateful for your support.

Our manuscript has been thoroughly modified, adhering to the guidelines shown in the Instructions to Authors on the journal homepage. We have also included the Figures and Supplementary figures in SVG file format in the attachments. Furthermore, we have updated the additional files incorporated in our study in the private User DropBox area (files.gigadb.org).

If there are any further steps required, please do not hesitate to let us know. Once again, thank you for your kind guidance.

Best regards,
Jihwan Park.
Gwangju Institute of Science and Technology, Associate Professor.
