# Supplementary material for: NanoMnT: an STR analysis tool for Oxford Nanopore sequencing data driven by a comprehensive analysis of error profile in STR regions
Source: Gigascience. 2025 Mar 17;14:giaf013. doi: 10.1093/gigascience/giaf013 (PMC11912559; doi:10.1093/gigascience/giaf013)
Supplement: giaf013_Response_to_Reviewer_Comments_Revision_1 [file giaf013_response_to_reviewer_comments_revision_1.pdf]

Author's Response To Reviewer Comments

Dear Editor of GigaScience,

We would like to express our sincere thanks and appreciation for your kind words and help.

We have filled in the missing required sections in our manuscript, including various citations, author contributions, citation styles, etc.

We would like to mention that while going through the Dome-ML annotation of our machine learning model used in our study, we found a minor mistake in our manuscript regarding the number of data points used for training/validating the model. During revision, we incorporated additional data points (STR loci), but we did not update the corresponding numbers. The numbers, which are at line 326 and 330, are now corrected.

Thank you for your kind guidance, and please don't hesitate to let us know if there is anything to change.

Best regards,
Jihwan
