# Supplementary material for: NanoMnT: an STR analysis tool for Oxford Nanopore sequencing data driven by a comprehensive analysis of error profile in STR regions
Source: Gigascience. 2025 Mar 17;14:giaf013. doi: 10.1093/gigascience/giaf013 (PMC11912559; doi:10.1093/gigascience/giaf013)
Supplement: giaf013_Reviewer_1_Report_Original_Submission — Qian Chris Liu -- 9/11/2024 [file giaf013_reviewer_1_report_original_submission.pdf]

Reviewer Report

Title: NanoMnT: An STR Analysis Tool for Oxford Nanopore Sequencing Data Driven by a Comprehensive Analysis of Error Profile in STR regions.

Version: Original Submission **Date:** 9/11/2024

Reviewer name: Qian Chris Liu

Reviewer Comments to Author:

Park et. al. investigated short-tandem-repeat regions using two ONT whole genome sequencing data. They examined how flanking regions, repeat motifs, repeat lengths and basecaller choices affect sequencing errors and base qualities in repeat regions. They then design NanoMnT to correct repeat sequencing errors and estimate repeat size and compared their tool against NanoSTR and NanoRepeat to estimate repeat size on 300 1-bp/2-bp repeat regions. The study is interesting, and the analysis is helpful to analyze STR using ONT data. But the research and writing need a big improvement.
1. Introduction: the discussion of existing tools needs to be expanded. This paper (https://www.nature.com/articles/s41592-023-01932-w#Sec16) has investigated 10 tools to estimate repeat size from long-read sequencing data, while this paper only discussed two of them. It is better to show the advantages of their tools compared to the 10 tools.
2. CNN: STR regions might have different lengths but are used as input of CNN. How they make CNN flexible to tolerate sequences with different lengths. What is the definition of sequencing accuracy? The input of the CNN is read sequences or reference sequences?
3. NanoMnT error correction: how to define a list of possible alleles for each aligned read?
4. It is great to share codes on github. But it is helpful to provide simple installations of the dependencies and their tool. It is also useful to provide instructions on how to re-generate their results.
5. It is great to show how to use NanoMnT on cancer samples. However, it is not clear how many loci are used for SG-Nex data and CRC data. Also, there is no comparison between NanoMnT and other tools. Figure 9(c) y-axis: -1 means 1bp difference? If it is true, the average difference is less than 1 bp usually and might not be significant.
6. Many terms are not clearly defined, for example: similar terms like Ax10: a 10 "A" repeat region with "A" is repeat unit? What is pMMR? What is MSS?
7. Many figures are not self-explanatory. Figure 1: in (a), how to define errorless reads? Which datasets are used to generate error profiles? Figure 2(b): figure legend for repeat units? Figure 4(a): "N(A)nN" should be "B(A)nB" based on HGMD nucleotide symbols. (b): what do colors mean? Figure 5(c): what is HAC/ SUP accuracy for a Ax10 STR locus? Figure 6(a): repeat regions have high base quality scores? Supplementary Figure 4: What does color indicate? Supplementary Figure 6: How to define "good" motifs and "bad" motifs? What does color indicate in (c)?

Methods

Are the methods appropriate to the aims of the study, are they well described, and are necessary controls included? Choose an item.

Conclusions

Are the conclusions adequately supported by the data shown? Choose an item.

Reporting Standards

Does the manuscript adhere to the journal's guidelines on minimum standards of reporting? Choose an item.

Choose an item.

Statistics

Are you able to assess all statistics in the manuscript, including the appropriateness of statistical tests used? Choose an item.

Quality of Written English

Please indicate the quality of language in the manuscript: Choose an item.

Declaration of Competing Interests

Please complete a declaration of competing interests, considering the following questions:

- Have you in the past five years received reimbursements, fees, funding, or salary from an organisation that may in any way gain or lose financially from the publication of this manuscript, either now or in the future?
- Do you hold any stocks or shares in an organisation that may in any way gain or lose financially from the publication of this manuscript, either now or in the future?
- Do you hold or are you currently applying for any patents relating to the content of the manuscript?
- Have you received reimbursements, fees, funding, or salary from an organization that holds or has applied for patents relating to the content of the manuscript?
- Do you have any other financial competing interests?
- Do you have any non-financial competing interests in relation to this paper?

If you can answer no to all of the above, write 'I declare that I have no competing interests' below. If your reply is yes to any, please give details below.

I declare that I have no competing interests

I agree to the open peer review policy of the journal. I understand that my name will be included on my report to the authors and, if the manuscript is accepted for publication, my named report including any attachments I upload will be posted on the website along with the authors' responses. I agree for my

report to be made available under an Open Access Creative Commons CC-BY license (http://creativecommons.org/licenses/by/4.0/). I understand that any comments which I do not wish to be included in my named report can be included as confidential comments to the editors, which will not be published.

Choose an item.

To further support our reviewers, we have joined with Publons, where you can gain additional credit to further highlight your hard work (see: https://publons.com/journal/530/gigascience). On publication of this paper, your review will be automatically added to Publons, you can then choose whether or not to claim your Publons credit. I understand this statement.

Yes Choose an item.
