# Supplementary material for: NanoMnT: an STR analysis tool for Oxford Nanopore sequencing data driven by a comprehensive analysis of error profile in STR regions
Source: Gigascience. 2025 Mar 17;14:giaf013. doi: 10.1093/gigascience/giaf013 (PMC11912559; doi:10.1093/gigascience/giaf013)
Supplement: giaf013_Reviewer_2_Report_Original_Submission — Rupesh Kesharwani -- 9/24/2024 [file giaf013_reviewer_2_report_original_submission.pdf]

Reviewer Report

Title: NanoMnT: An STR Analysis Tool for Oxford Nanopore Sequencing Data Driven by a Comprehensive Analysis of Error Profile in STR regions.

Version: Original Submission **Date:** 9/24/2024

Reviewer name: Rupesh Kesharwani

Reviewer Comments to Author:

This manuscript "NanoMnT: A STR analysis tool for Oxford Nanopore sequencing data driven by comprehensive analysis of error profile in STR regions" presents a valuable contribution to the field of STR analysis using long reads such as Oxford Nanopore sequencing data. The authors have conducted a thorough investigation into the error profiles of STR regions, identifying key factors that influence sequencing accuracy. The application of MSI detection in cancer samples demonstrates its practical utility. However, there are several areas where revisions and improvements are necessary. The comments are as follows:

Comments:

1. The abstract provides a clear and comprehensive summary of the study. However, it could be improved by highlighting the novelty of the work, % of accuracy corrected from their results and its potential impact on the field. There are no data showed as claimed.
2. The introduction provides a good overview of the background and significance of STR analysis. However, it could be strengthened by providing more specific examples of how ONT's high error rate limits its use in STR analysis. Additionally, the authors could discuss the limitations of existing tools in more detail, highlighting the need for NanoMnT. Also, report which nanopore sequencing platforms have what level of errors. As best of my knowledge it has been evolved significantly over the years. Also, good to have some numbers from PacBio hifi sequencing too along with the cost comparison as discussed in this section.
3. The methods section is generally well-written and provides sufficient detail for reproducibility. However, some areas could benefit from further clarification. For example, the authors could provide more information on the training and validation of the CNN model. Additionally, the authors could provide more details on the implementation of NanoMnT using the CNN and why they have used this over other machine learning algorithms. Also, would be good idea to brief why the authors choose to work on T2T over hg38. I know that T2T have repetitive sequences that hg38 but little more basics would be a good read.
4. The results section is well-organized and presents the findings clearly. The figures are informative and support the conclusions drawn. However, some additional analyses could be performed to strengthen the results. For example, the authors could compare the performance of NanoMnT to other existing tools (such as NanoSTR (Lang et al., 2023), NanoRepeat (Fang et al., 2023)) on a larger dataset or the study such as using the benchmarking sets fro the recent study i.e. English, A.C., et al. (2024). Analysis and benchmarking of small and large genomic variants across tandem repeats. Additionally, the authors could investigate the impact of different sequencing parameters on the performance of NanoMnT.

5. The discussion is well written, but the results are not discussed properly, must be revised in-depth analysis it with acknowledge the limitations of the study and suggest future directions for research.
6. The overall the manuscript is good, but there are places where clarity and conciseness are needed. For example, in the introduction, the authors state, "STRs are also known to play important roles in the pathogenesis of numerous diseases." This sentence should be made more specific, with examples of the diseases.

Methods

Are the methods appropriate to the aims of the study, are they well described, and are necessary controls included? Choose an item.

Conclusions

Are the conclusions adequately supported by the data shown? Choose an item.

Reporting Standards

Does the manuscript adhere to the journal's guidelines on minimum standards of reporting? Choose an item.

Choose an item.

Statistics

Are you able to assess all statistics in the manuscript, including the appropriateness of statistical tests used? Choose an item.

Quality of Written English

Please indicate the quality of language in the manuscript: Choose an item.

Declaration of Competing Interests

Please complete a declaration of competing interests, considering the following questions:

- Have you in the past five years received reimbursements, fees, funding, or salary from an organisation that may in any way gain or lose financially from the publication of this manuscript, either now or in the future?
- Do you hold any stocks or shares in an organisation that may in any way gain or lose financially from the publication of this manuscript, either now or in the future?
- Do you hold or are you currently applying for any patents relating to the content of the manuscript?

- Have you received reimbursements, fees, funding, or salary from an organization that holds or has applied for patents relating to the content of the manuscript?
- Do you have any other financial competing interests?
- Do you have any non-financial competing interests in relation to this paper?

If you can answer no to all of the above, write 'I declare that I have no competing interests' below. If your reply is yes to any, please give details below.

I declare that I have no competing interests

I agree to the open peer review policy of the journal. I understand that my name will be included on my report to the authors and, if the manuscript is accepted for publication, my named report including any attachments I upload will be posted on the website along with the authors' responses. I agree for my report to be made available under an Open Access Creative Commons CC-BY license (http://creativecommons.org/licenses/by/4.0/). I understand that any comments which I do not wish to be included in my named report can be included as confidential comments to the editors, which will not be published.

Choose an item.

To further support our reviewers, we have joined with Publons, where you can gain additional credit to further highlight your hard work (see: https://publons.com/journal/530/gigascience). On publication of this paper, your review will be automatically added to Publons, you can then choose whether or not to claim your Publons credit. I understand this statement.

Yes Choose an item.
