# Supplementary material for: NanoMnT: an STR analysis tool for Oxford Nanopore sequencing data driven by a comprehensive analysis of error profile in STR regions
Source: Gigascience. 2025 Mar 17;14:giaf013. doi: 10.1093/gigascience/giaf013 (PMC11912559; doi:10.1093/gigascience/giaf013)
Supplement: giaf013_Reviewer_3_Report_Original_Submission — Roxanne Richelle Zascavage, Ph.D. -- 10/1/2024 [file giaf013_reviewer_3_report_original_submission.pdf]

Reviewer Report

Title: NanoMnT: An STR Analysis Tool for Oxford Nanopore Sequencing Data Driven by a Comprehensive Analysis of Error Profile in STR regions.

Version: Original Submission **Date:** 10/1/2024

Reviewer name: Roxanne Richelle Zascavage, Ph.D.

Reviewer Comments to Author:

The authors provided a thorough assessment of nanopore data using different basecallers and chemistries. Although the previous versions of the chemistries have been phased out, there is value in a comparison in performance across them. The manuscript also introduces a novel software package which users of ONT platforms may find useful. However, the authors do not seem up to date on the current status of ONT sequencing, some of the methods in the presented approach seem counterintuitive to the end goal of the proposed project, and a deeper analysis that supports the conclusions drawn could have been performed without the removal of all the "troublesome" data. Please see specific comments below:

Introduction:

-Author states "much previous research has shown that STRs..." Only a single reference was provided here, which is insufficient to count as "much research".

-References are needed to back many claims made in the introduction. A few I noted: 1. NGS has expanded the number of STRs that can be analyzed, 2. WGS can enable analysis of most STR regions, 3. long read sequencing is advantageous for characterization of STR regions compared to short read sequencing (especially given the additional coverage and accuracy provided by MPS), 4. ONT provides superior throughput cost, and 5. ONT has particularly high error rates in low-complexity regions.

-ONT accuracy has increased significantly since their introduction, especially with the R14 chemistries and flow cells, but this paper still treats it like it is the same system that it was a decade ago.

-Is the paper saying Nanopore is no longer supported as of 2018? I do not believe so, as NanoSTR was published in 2023, and their github website was updated as recently as 10 months ago. I think they are referring to Porechop, but this is not clear from the sentence, especially as the next sentence opens with "On the other hand, while Nanorepeat is regularly updated..."

Methods:

-One of the major conclusions drawn is that flanking regions are essential for accurate sequencing. However, the authors eliminated any STRs with low complexity in their flanking regions. An assessment of even a small subset of these loci would go a long way in supporting the conclusions about flank importance. This is also valuable for a tool designed to evaluate STRs, particularly low complexity STRs. It would help to demonstrate the limitations of the software, which cannot be done if these data are removed prior to analysis due to the assumption of a limitation.

-It is unclear why the coverage requirements and loci assessed were different between the T2t-CHM13 dataset and the HG002 dataset. I would presume that ultimate use of an STR analysis software would be working on samples with unknown profiles/background. What would be the required depth of coverage

for accuracy calls? Also, while I understand the loci assessment was different due to variations in heterozygosity, what if future users are wanting to evaluate unknown samples without any knowledge of the degree of heterozygosity? Are there guidelines for standard STR loci that are safe to be evaluated with these methods and software? A standard approach to all datasets, with conclusions being drawn about what would be considered acceptable results/loci (depth of coverage, heterozygosity, etc) that one could expect reliable results from would be more valuable than a presumptive screening based on knowledge of the dataset itself. How will future users know how to replicate these methods on novel dataset?

- Did the original alignment first include alignment to the reference genome? Given the simplicity of the loci being assessed, how did the authors account for/ensure elimination of off-target alignments, as these could result in an increase in observed errors.

Results:

-Sequencing error rates were calculated by counting the number of error-less reads. Accuracy between forward and reverse strands was also compared, which was a nice touch by the authors.

-It would help if the authors reported raw numbers in addition to percentages. Error section does not include numbers for types of errors at all.

-While sequencing errors across the board are interesting to share, most analyses will evaluate a consensus sequence. It is unclear, so I could be wrong, but it does not appear the authors evaluated the consensus to see if the overall call included any errors. Such an analysis would help to allow this platform to be compared to other published studies, and give an overall assessment of the reliability of final results using this program.

-The applications of NanoMnT to cancer screening are valuable and well laid out.

Discussion:

-Were any motifs identified that were more likely to result in Indels?

-Typo: "The excess number of insertions and substitutions..." but authors said most of the errors were deletions.

-More details about what could have caused the differences in those STRs that responded well the the SUP basecaller vs those that performed better using the HAC would be beneficial.

-The authors acknowledged the shortcomings of this study, including the exclusion of many imperfect STRs.

Language: The manuscript could use heavy editing to improve grammar.

Figures/Tables:

-The gradient effects on Figure 1 are negligible and would not be noticed if it were not for the legend.

Methods

Are the methods appropriate to the aims of the study, are they well described, and are necessary controls included? Choose an item.

Conclusions

Are the conclusions adequately supported by the data shown? Choose an item.

Reporting Standards

Does the manuscript adhere to the journal's guidelines on [minimum standards of reporting?]() Choose an item.

Choose an item.

Statistics

Are you able to assess all statistics in the manuscript, including the appropriateness of statistical tests used? Choose an item.

Quality of Written English

Please indicate the quality of language in the manuscript: Choose an item.

Declaration of Competing Interests

Please complete a declaration of competing interests, considering the following questions:

- Have you in the past five years received reimbursements, fees, funding, or salary from an organisation that may in any way gain or lose financially from the publication of this manuscript, either now or in the future?
- Do you hold any stocks or shares in an organisation that may in any way gain or lose financially from the publication of this manuscript, either now or in the future?
- Do you hold or are you currently applying for any patents relating to the content of the manuscript?
- Have you received reimbursements, fees, funding, or salary from an organization that holds or has applied for patents relating to the content of the manuscript?
- Do you have any other financial competing interests?
- Do you have any non-financial competing interests in relation to this paper?

If you can answer no to all of the above, write 'I declare that I have no competing interests' below. If your reply is yes to any, please give details below.

'I declare that I have no competing interests

I agree to the open peer review policy of the journal. I understand that my name will be included on my report to the authors and, if the manuscript is accepted for publication, my named report including any attachments I upload will be posted on the website along with the authors' responses. I agree for my report to be made available under an Open Access Creative Commons CC-BY license (http://creativecommons.org/licenses/by/4.0/). I understand that any comments which I do not wish to be included in my named report can be included as confidential comments to the editors, which will not be published.

Choose an item.

To further support our reviewers, we have joined with Publons, where you can gain additional credit to further highlight your hard work (see: https://publons.com/journal/530/gigascience). On publication of this paper, your review will be automatically added to Publons, you can then choose whether or not to claim your Publons credit. I understand this statement.

Yes Choose an item.
